# Comparative effectiveness of different exercise modality on glycaemic control and lipid profile for prediabetes: systematic review and network meta-analysis

**DOI:** 10.3389/fendo.2025.1518871

**Published:** 2025-07-24

**Authors:** Ruixiang Yan, Lunxin Chen, Gesheng Lin, Yuer Shi, Wenrui Huang, Yuqiang Mai, Jian Sun, Duanying Li

**Affiliations:** ^1^ School of Athletic Training, Guangzhou Sport University, Guangzhou, China; ^2^ School of Physical Education and Sport, Central China Normal University, Wuhan, China; ^3^ The Fourth Clinical Medical College of Guangzhou University of Chinese Medicine, Shenzhen, Guangdong, China; ^4^ School of Wushu, Guangzhou Sport University, Guangzhou, China; ^5^ Guangdong Provincial Key Laboratory of Human Sports Performance Science, Guangzhou, China

**Keywords:** prediabetes, glycaemic, lipid profile, exercise, weight

## Abstract

**Background:**

Prediabetes is the precursor to type 2 diabetes and represents a critical, reversible window for intervention. This study aims to systematically review and conduct a network meta-analysis to evaluate the efficacy of aerobic training (AT), resistance training (RT), combined training (AT+RT), high-intensity interval training (HIIT), and traditional Chinese exercises (TCEs) on glycemic control, lipid profile, and weight management in prediabetic individuals. This marks the first time HIIT and TCEs have been included in such an assessment.

**Methods:**

A systematic search was conducted across PubMed, Web of Science, Cochrane (CENTRAL), Embase, CNKI, and WangFang Data for randomized controlled trials (RCTs) on the effects of different exercise modalities on prediabetic patients published up to August 10, 2024. Network meta-analysis was performed using the “gemtc” package in R software, and the quality of evidence was assessed using the CINeMA framework.

**Results:**

A total of 74 studies involving 5,683 participants were included. The network meta-analysis results showed that HIIT was the most effective for reducing haemoglobin A1c (HbA1c) (-0.44%, 95% CI: -0.55% to -0.32%, SUCRA 93.8%), 2-hour plasma glucose (2hPG) (-1.3, 95% CI: -1.6 to -0.93, SUCRA 84.3%), and increasing high-density lipoprotein (HDL) (0.20, 95% CI: 0.03 to 0.36, SUCRA 87.3%). AT+RT was most effective in reducing total cholesterol (TC) (-0.46, 95% CI: -0.61 to -0.32, SUCRA 98.3%), TG (-0.55, 95% CI: -0.69 to -0.42, SUCRA 99.9%), low-density lipoprotein (LDL) (-0.35, 95% CI: -0.53 to -0.18, SUCRA 82.2%), and body mass index (BMI) (-0.89, 95% CI: -1.6 to -0.14, SUCRA 66.4%). TCEs showed the most significant improvements in reducing 2hPG (-1.3, 95% CI: -1.5 to -1.0, SUCRA 83.5%), body weight (BW) (-3.4, 95% CI: -6.4 to -0.51, SUCRA 79.1%), and wasit circumference (WC) (-4.27, 95% CI: -7.53 to -0.98, SUCRA 84.6%).

**Conclusion:**

Various exercise interventions significantly improved glycemic and lipid profiles in prediabetic patients. HIIT and AT+RT were found to be the most effective interventions. For elderly individuals with limited physical activity or chronic conditions, TCEs can serve as a gentle and safe alternative. These findings provide the latest evidence to support exercise interventions for managing prediabetes.

**Systematic review registration:**

https://www.crd.york.ac.uk/prospero/, identifier CRD42024578405.

## 1 Introduction

Prediabetes is a condition where blood glucose levels are elevated but not high enough for a diabetes diagnosis (1). In 2019, the International Diabetes Federation (IDF) estimated that 7.5% of the global adult population—around 374 million people—were affected by prediabetes, a figure expected to increase to 548 million by 2045, presenting significant challenges for diabetes management (2). As the precursor to type 2 diabetes, prediabetes represents a crucial opportunity for intervention while it remains reversible (1, 3). Without intervention, up to 70% of individuals with prediabetes will develop diabetes, according to the American Diabetes Association (ADA). Additionally, prediabetes carries similar risks to diabetes, including cardiovascular disease, stroke, and kidney complications, affecting individuals of all ages (1, 4, 5). Addressing modifiable risk factors such as obesity, poor diet, and inactivity can help prevent or delay the onset of type 2 diabetes (6, 7). Numerous studies show that both lifestyle changes and pharmacological interventions can reduce the risk of diabetes, with some individuals returning to normal glucose levels (8–10). Notably, the Diabetes Prevention Program (DPP) found that lifestyle interventions were more effective than medication, offering longer-term benefits and broader health advantages (11, 12).

Exercise plays a crucial role in lifestyle interventions, helping to improve blood sugar control, insulin sensitivity, body composition, blood pressure, and lipid levels, while also lowering cardiovascular risk (13, 14). Consequently, international guidelines recommend at least 150 minutes of moderate-to-vigorous aerobic activity and twice-weekly resistance training to prevent and manage type 2 diabetes and cardiovascular conditions (15). Despite these recommendations, only 12% of older adults meet the required activity levels (16). While high-intensity interval training (HIIT) offers a time-efficient way to achieve similar health benefits, its practicality for older adults is often limited by physical challenges (17, 18). By contrast, Traditional Chinese Exercises (TCEs) offer a gentler and safer alternative, particularly for older individuals. Studies have shown that TCEs significantly improve blood glucose and lipid levels in patients with type 2 diabetes, helping to delay disease progression and reduce complications (19, 20). As a result, both the ADA and the American College of Sports Medicine (ACSM) recommend TCEs for individuals with type 2 diabetes, advising 2–3 sessions per week (21).

Previous meta-analyses have generally grouped exercise into three broad categories: aerobic (AT), resistance (RT), and combined training (AT+RT), without fully exploring the specific roles and mechanisms of different exercise types in diabetes prevention (22–26). To address this, we conducted the first network meta-analysis comparing HIIT and TCEs. This study comprehensively evaluates the effectiveness of various exercise types in regulating blood glucose, lipid levels, and body weight in individuals with prediabetes. In addition to exercise type, we examined how different intensities, durations, frequencies, and cycles influence glycemic and lipid regulation. Our findings offer the latest evidence on the impact of exercise interventions for managing prediabetes, providing a foundation for future guidelines.

## 2 Method

### 2.1 Protocol and registration

This systematic review and network meta-analysis is registered with PROSPERO (Registration number: CRD42024578405). The study adheres to the PRISMA 2020 guidelines (Preferred Reporting Items for Systematic Reviews and Meta-Analyses) and the PRISMA extension for network meta-analyses (PRISMA-NMA) (27, 28).

### 2.2 Search strategy and study selection

We performed a comprehensive search across PubMed, Web of Science, Cochrane (CENTRAL), Embase, CNKI, and WangFang Data to identify randomized controlled trials (RCTs) examining the effects of various exercise modalities on individuals with prediabetes. The search included studies published from the databases’ inception to August 10, 2024. To ensure comprehensive coverage of RCTs in the field of prediabetes, we modified the PubMed search strategy to include both “randomized” and “randomised” to account for potential discrepancies between American and British English spelling, thus minimizing the risk of missing relevant studies (29). Three reviewers (GS, BW, and LX) independently screened studies, with any discrepancies resolved by consulting a fourth reviewer (YE). Additionally, reference lists from included studies and relevant systematic reviews were screened for potential eligible studies. The full search strategy is detailed in **Appendix 1**.

### 2.3 Eligibility criteria

Study eligibility was assessed using the PICOS framework (Participants, Interventions, Comparators, Outcomes, and Study design) (30). Studies were included if they met the following criteria:

#### 2.3.1 Population

We included studies with participants aged 18 years or older diagnosed with prediabetes, excluding those with diabetes, severe comorbidities, children, adolescents, or pregnant women. The diagnosis of prediabetes was based on the ADA criteria and met at least one of the following conditions: fasting blood glucose (FBG) between 5.6–6.9 mmol/L, hemoglobin A1c (HbA1c) between 39–47 mmol/mol (5.7–6.4%), or 2-hour plasma glucose levels between 7.8–11.0 mmol/L during an oral glucose tolerance test (OGTT) (31).

#### 2.3.2 Intervention

We categorized exercise into five different types: AT, RT, AT+RT, HIIT, TCEs. The definitions of each exercise intervention and the classification of exercise intensity are detailed in **Appendix 2**.

#### 2.3.3 Comparator

Control groups consisted of either one of the five exercise types, health education, usual care, or a waitlist group.

#### 2.3.4 Outcome

Studies were required to report at least one of the following outcomes: glycemic control (HbA1c, FBG, 2-hour postprandial glucose (2hPG)), lipid control (total cholesterol (TC), triglycerides (TG), high-density lipoprotein (HDL), low-density lipoprotein (LDL)), or weight control (body weight (BW), body mass index (BMI), waist circumference (WC)).

#### 2.3.5 Study design

Only RCTs were eligible. Studies were excluded if they:

- Were non-randomized controlled trials.
- Involved pharmacological treatments or dietary interventions.
- Were conference abstracts, protocols, or systematic reviews.
- Lacked sufficient data for analysis.
- Did not provide full-text access through relevant databases or other sources.

### 2.4 Data extraction

For each eligible study, data were independently extracted using a pre-designed form. Extracted information included study characteristics (first author, publication year, country), population demographics (age, gender, sample size), intervention details (type, duration, frequency, intensity), and outcome measures. In cases where data were unavailable, the corresponding author was contacted up to three times over a three-week period. Two independent reviewers (GS, BW) conducted the data extraction, with a third reviewer (LX) verifying the results and resolving any discrepancies. If data were missing, the corresponding author was contacted up to three times within three weeks.

### 2.5 Measures of treatment effect

This meta-analysis evaluated treatment effects using mean difference (MD) and changes in standard deviation (SD). When SD values were not reported directly in the original studies, we estimated them based on standard error, 95% confidence intervals (CI), p-values, or t-statistics (32). To calculate the difference in SD before and after the intervention, we assumed a correlation coefficient of 0.5, reflecting a commonly accepted level of moderate measurement repeatability in the literature. This value was chosen to account for potential variability while ensuring the robustness and reliability of the results (32).

### 2.6 Quality assessment of evidence

**Figure 1:**
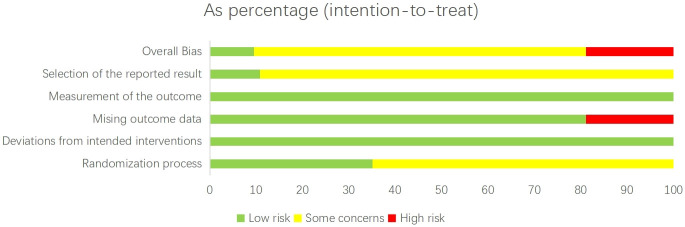
Summary of the risk of bias assessment in the individual domains of the included studies.

We assessed the risk of bias in the included trials using the Cochrane Risk of Bias tool for randomized controlled trials (version 2.0) (**Figure 1**). This evaluation considered vital factors such as random sequence generation, allocation concealment, blinding, incomplete outcome data, and selective reporting (33). A study was classified as having a low overall risk of bias (score 1) if all domains were rated as low risk. If any domain was rated as high risk, the study was classified as high risk of bias (score 3). Studies with intermediate concerns were given a score of 2. Two independent reviewers performed the assessments, and disagreements were resolved through discussion.

We generated funnel plots for each direct comparison to identify minor study effects and publication bias. We also applied the CINeMA (Confidence in Network Meta-Analysis) framework to evaluate the certainty of evidence across six domains: within-study bias, reporting bias, indirectness, imprecision, heterogeneity, and incoherence (34, 35). These domains assess potential systematic errors within studies, the effects of selective reporting and publication bias, the relevance of the evidence, the precision of effect estimates, consistency across studies, and the agreement between direct and indirect evidence.

### 2.7 Statistical analysis

We conducted the network meta-analysis using the gemtc package in R (version 4.3.3) to evaluate the efficacy of various exercise interventions for type 2 diabetes patients. The Bayesian framework within the gemtc package enables simultaneous comparisons of multiple treatments. To visualize the network of comparisons, we used the “networkplot” function in STATA 17, where nodes represent different interventions and edges denote direct head-to-head comparisons.

Treatment effects were estimated using the Markov Chain Monte Carlo (MCMC) method, with a random-effects model applied to account for heterogeneity across studies (36, 37). Outcomes were standardized for comparability, using mean difference (MD) and 95% credible intervals (CrI) as the primary effect measure. Heterogeneity was assessed using τ², following established thresholds (low <0.04; low-moderate 0.04-0.16; moderate-high 0.16-0.36; high >0.36) (38, 39).

To assess local inconsistency, a node-splitting analysis was conducted within a Bayesian hierarchical framework using MCMC sampling in the gemtc package. Direct effects (obtained from head-to-head trials) and indirect effects (derived from the network) were separately estimated. Under the consistency model, direct and indirect evidence were analyzed jointly to estimate the overall effect. Under the inconsistency model, they were estimated separately, allowing for potential differences between the two estimates. The difference between direct and indirect effects was assessed by calculating a Bayesian p-value based on the posterior distribution of their difference, with p < 0.05 indicating significant inconsistency. When inconsistency was detected, the certainty of evidence was downgraded according to the CINeMA framework (40).

To test the transitivity assumption, we compared the distribution of key modifiers across studies, including average age, Baseline HbA1c. This ensured comparability between groups and strengthened the robustness of the results. Intervention efficacy was ranked using Surface Under the Cumulative Ranking (SUCRA), quantifying the likelihood of an intervention being the best treatment option (41).

We further explored potential moderators of treatment effect through meta-regression using the gemtc package, focusing on factors such as age, gender proportion, and intervention duration to explain heterogeneity. Subgroup analyses were also performed based on patient characteristics, intervention intensity, and duration to assess differences in treatment effects.

## 3 Results

### 3.1 Literature selection and study characteristics

**Figure 2:**
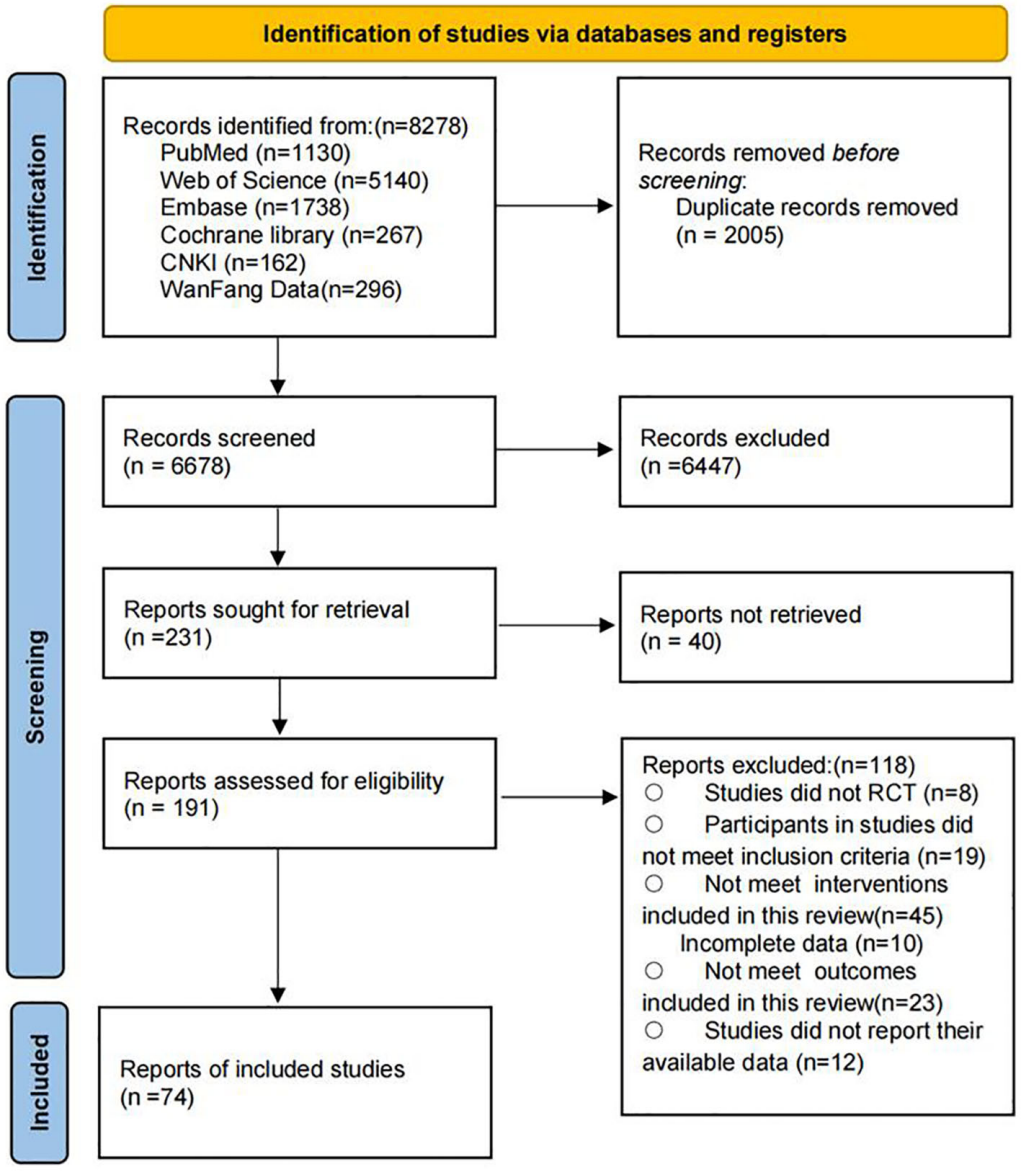
PRISMA flow diagram of the search process for studies.

A total of 8,278 potential records were identified through the systematic search. After removing duplicates, 6,678 articles remained for title and abstract screening. The authors conducted full-text reviews on 191 articles that met the inclusion criteria. Ultimately, 74 studies were included in this systematic review and meta-analysis, involving 5,683 participants, of whom 46.7% were male. The mean age of the participants was 56.25 years (standard deviation 6.6), and the mean body mass index was 26.79 (standard deviation 2.72). The complete screening and selection process is shown in **Figure 2**. A summary of the number of studies and participants for each outcome is presented in **Table 1**.

**Table 1:** Summary of the number of studies and participants for each outcome.

Outcome	Number of studies	Number of participants
HbA1c	43	3895
FBG	66	5404
2hPG	48	3941
TC	32	2868
TG	34	3172
HDL	34	3150
LDL	32	3100
BMI	40	3226
BW	23	1534
WC	18	1155

HbA1c, hemoglobin A1c; FBG, fasting blood glucose; 2hPG, 2-hour postprandial glucose; TC, total cholesterol; TG, triglycerides; HDL, high-density lipoprotein; LDL, low-density lipoprotein; BMI, body mass index; BW, body weight; WC, waist circumference.

### 3.2 Risk of bias, certainty of evidence, and consistency

The risk of bias for each trial is presented in **Appendix 4**. Overall, 7 studies (9.5%) were classified as having a low risk of bias, 53 studies (71.6%) as having an unclear risk of bias, and 14 studies (18.9%) as having a high risk of bias. In the consistency assessment, which evaluates the agreement between direct and indirect evidence, the node-splitting analysis identified inconsistencies in certain comparisons. For example, significant discrepancies were observed in the comparisons AT vs. TCEs (p = 0.006) and TCEs vs. C (p = 0.028) for HbA1c, indicating inconsistency between direct and indirect estimates. Consequently, the certainty of evidence for these outcomes was downgraded. However, no statistically significant local inconsistency was detected in most treatment comparisons (**Appendix 5**). Furthermore, the consistency assessment of HbA1c data (P = 0.0586) suggests that the results should be interpreted cautiously, indicating the need for more rigorous randomized controlled trials in the future. The τ² results did not reveal any high heterogeneity in the network; most outcomes displayed low to moderate heterogeneity, with a few showing moderate to high levels of heterogeneity (**Appendix 5**). Using the CINeMA framework for quality assessment, we found that most pairwise comparisons yielded evidence of very low to moderate quality (**Appendix 9**). All networks met the transitivity assumption, ensuring indirect comparisons’ validity (**Appendix 9**, **Supplementary Figure S9.1**). Additionally, no asymmetry was detected in the funnel plot analysis, indicating no apparent publication bias (**Appendix 10**).

**Figure 3:**
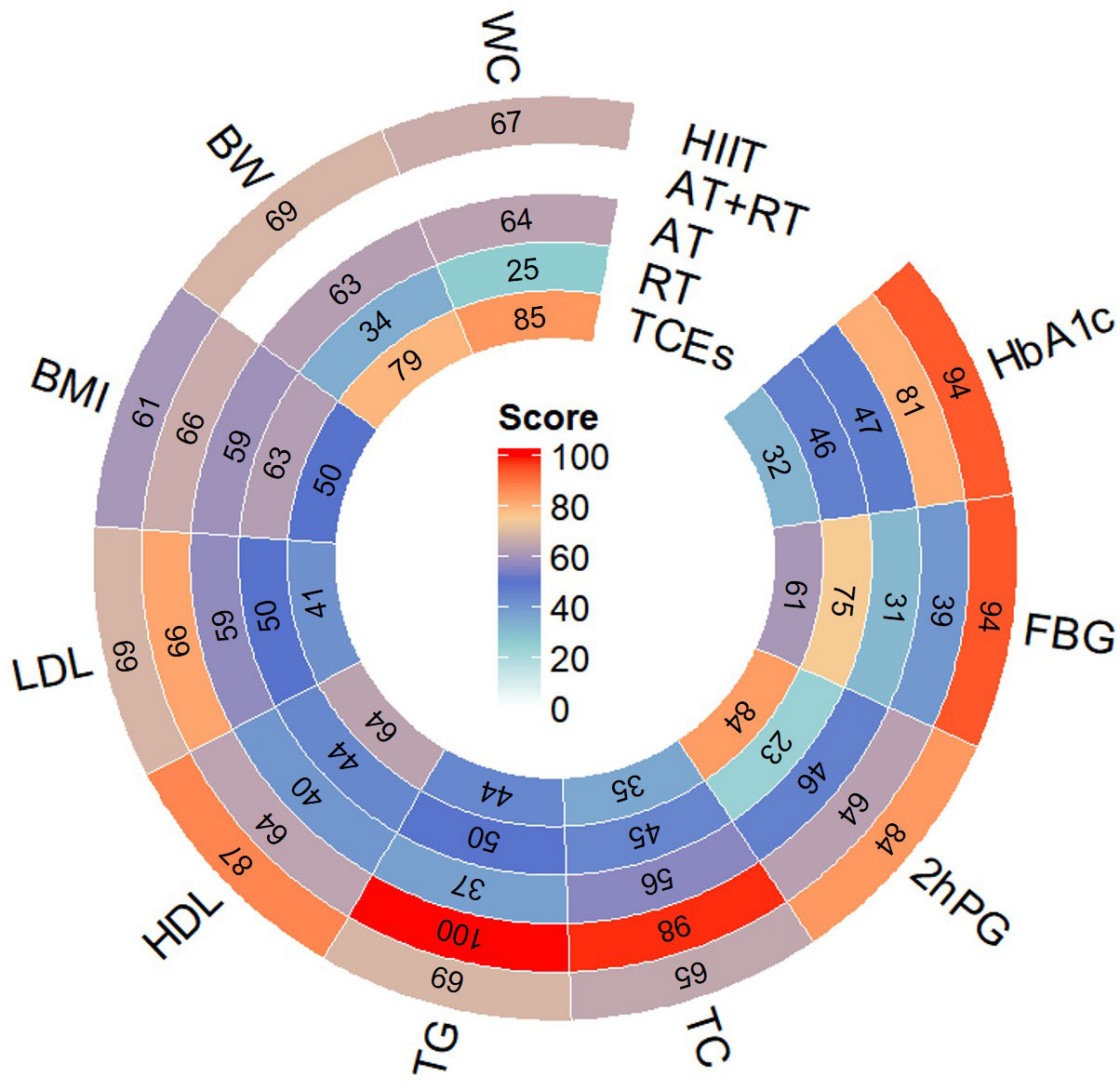
Circular Heatmap of SUCRA Rankings for Exercise Interventions Across All Outcomes.Note: This circular heatmap presents the Surface Under the Cumulative Ranking Curve (SUCRA) scores for five exercise interventions (HIIT, AT+RT, AT, RT, and TCEs) across nine outcomes: glycemic control (HbA1c, FBG, 2hPG), blood lipids (TG, TC, HDL, LDL), and body composition indicators (BMI, BW, WC).The color gradient represents SUCRA rankings, where red indicates the highest ranking, signifying the most effective intervention for improving that outcome, while blue represents lower rankings. The numbers inside each segment denote the SUCRA scores (range: 0–100), with higher values indicating a greater probability of being the most effective intervention.

To enhance clarity and facilitate interpretation, we present a circular heatmap in **Figure 3** summarizing SUCRA rankings. This visualization provides a comparative overview of the effectiveness of each intervention in improving glycaemic control, lipid profiles, and body composition, reinforcing the study’s key conclusions.

### 3.3 Glycaemic control

**Figure 4:**
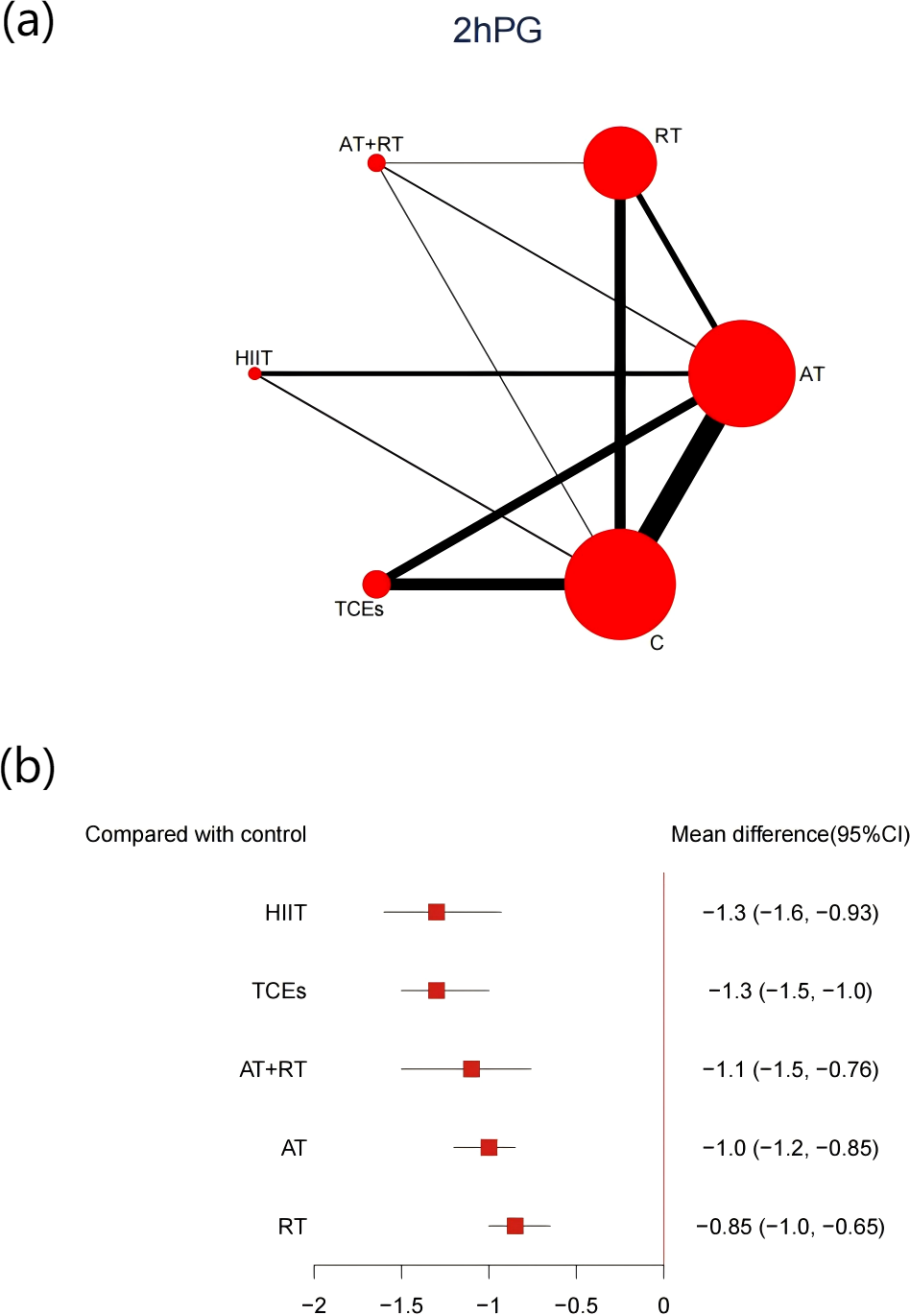
**(a)** Network map of the effect on HbA1c, and forest plot of network effect sizes for compared with control. **(b)** Forest plot of network effect sizes between different exercise interventions and control for HbA1c measured in percentage.

A total of 43 studies, comprising 3,895 participants, reported changes in HbA1c levels. The network meta-analysis indicated that all exercise interventions significantly reduced HbA1c levels in prediabetic individuals compared with control groups (**Figure 4**). HIIT demonstrated the most pronounced reduction (-0.44%, 95% CI: -0.55 to -0.32, SUCRA 93.8%, Moderate confidence of evidence), followed by combined aerobic and AT+RT (-0.39%, 95% CI: -0.53 to -0.25, SUCRA 80.7%, Moderate confidence of evidence), with AT having a weaker effect (-0.3%, 95% CI: -0.34 to -0.24, SUCRA 47.2%, Moderate confidence of evidence). Comparisons further revealed that HIIT significantly outperformed AT, RT, and TCEs in improving HbA1c levels (**Appendix 8**, **Supplementary Table S8.1**).

**Figure 5:**
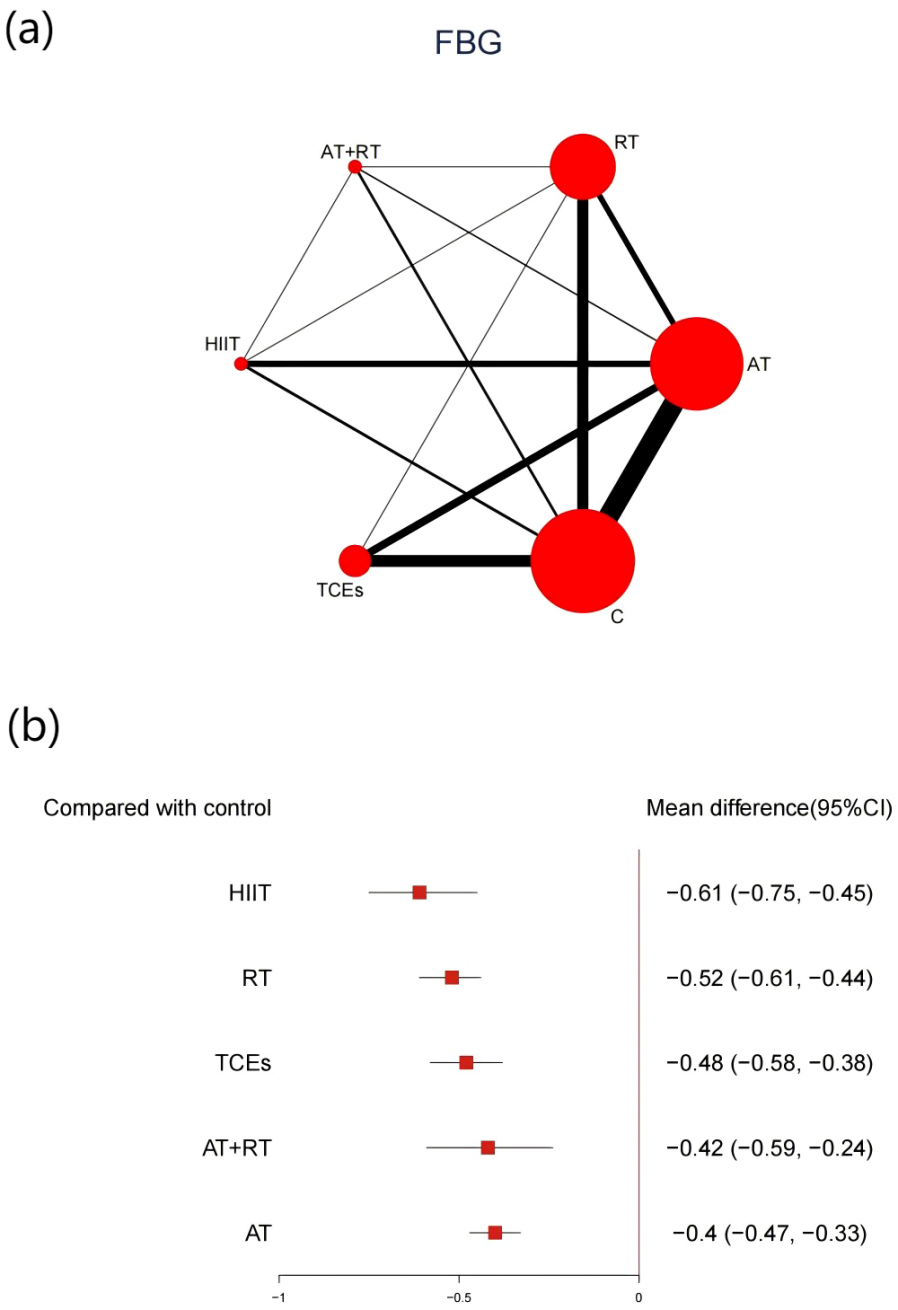
**(a)** Network map of the effect on FBG, and forest plot of network effect sizes for compared with control. **(b)**. Forest plot of network effect sizes between different exercise interventions and control for FBG.

A total of 66 studies involving 5,404 participants reported changes in FBG. Compared with control groups, all exercise modalities yielded significant improvements (**Figure 5**). HIIT was associated with the most substantial reduction in FBG (-0.61, 95% CI: -0.75 to -0.45, SUCRA 94.1%, Very low confidence of evidence), followed by RT (-0.52, 95% CI: -0.61 to -0.44, SUCRA 75.3%, Low confidence of evidence), while TCEs showed a relatively smaller effect (-0.48, 95% CI: -0.58 to -0.38, SUCRA 60.7%, Low confidence of evidence). Further comparisons showed that HIIT and RT were significantly more effective than AT in lowering FBG (**Appendix 8**, **Supplementary Table S8.2**).

**Figure 6:**
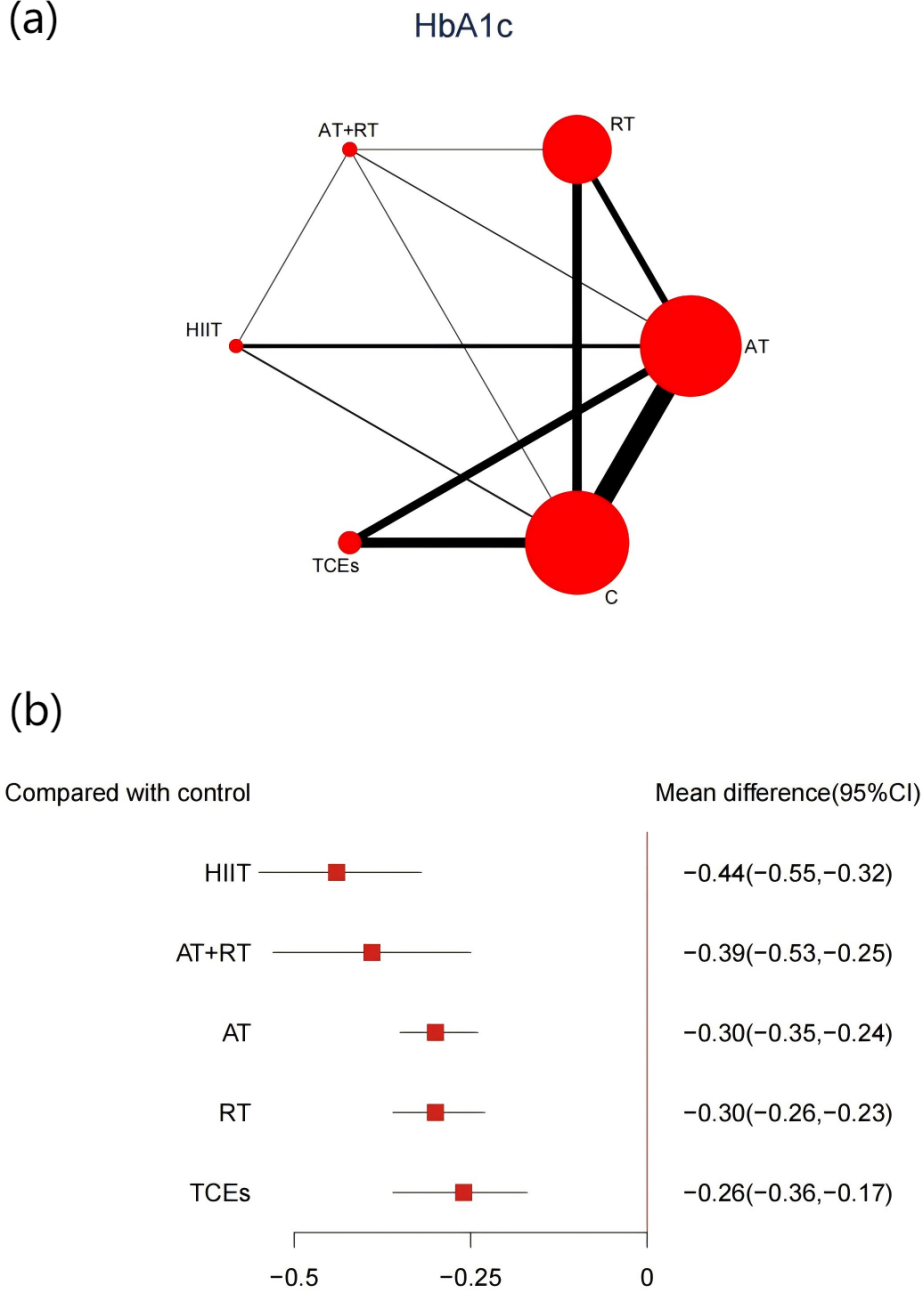
**(a)** Network map of the effect on 2hPG, and forest plot of network effect sizes for compared with control. **(b)**. Forest plot of network effect sizes between different exercise interventions and control for 2hPG.

48 studies, comprising 3,941 participants, reported changes in 2hPG. All exercise interventions produced significant reductions compared with control groups (**Figure 6**). HIIT showed the most significant reduction in 2hPG (-1.3, 95% CI: -1.6 to -0.93, SUCRA 84.3%, Very low confidence of evidence), followed by TCEs (-1.3, 95% CI: -1.5 to -1.0, SUCRA 83.5%, Low confidence of evidence), and AT+RT (-1.1, 95% CI: -1.5 to -1.0, SUCRA 63.5%, Low confidence of evidence). Further analyses indicated that HIIT was significantly more effective than RT in reducing 2hPG (**Appendix 8**, **Supplementary Table S8.3**).

The CINeMA assessment revealed that the overall certainty of the evidence for HbA1c, FBG, and 2hPG ranged from very low to moderate (**Appendix 9**, **Supplementary Figure S9.2**).

### 3.4 Lipid profiles

**Figure 7:**
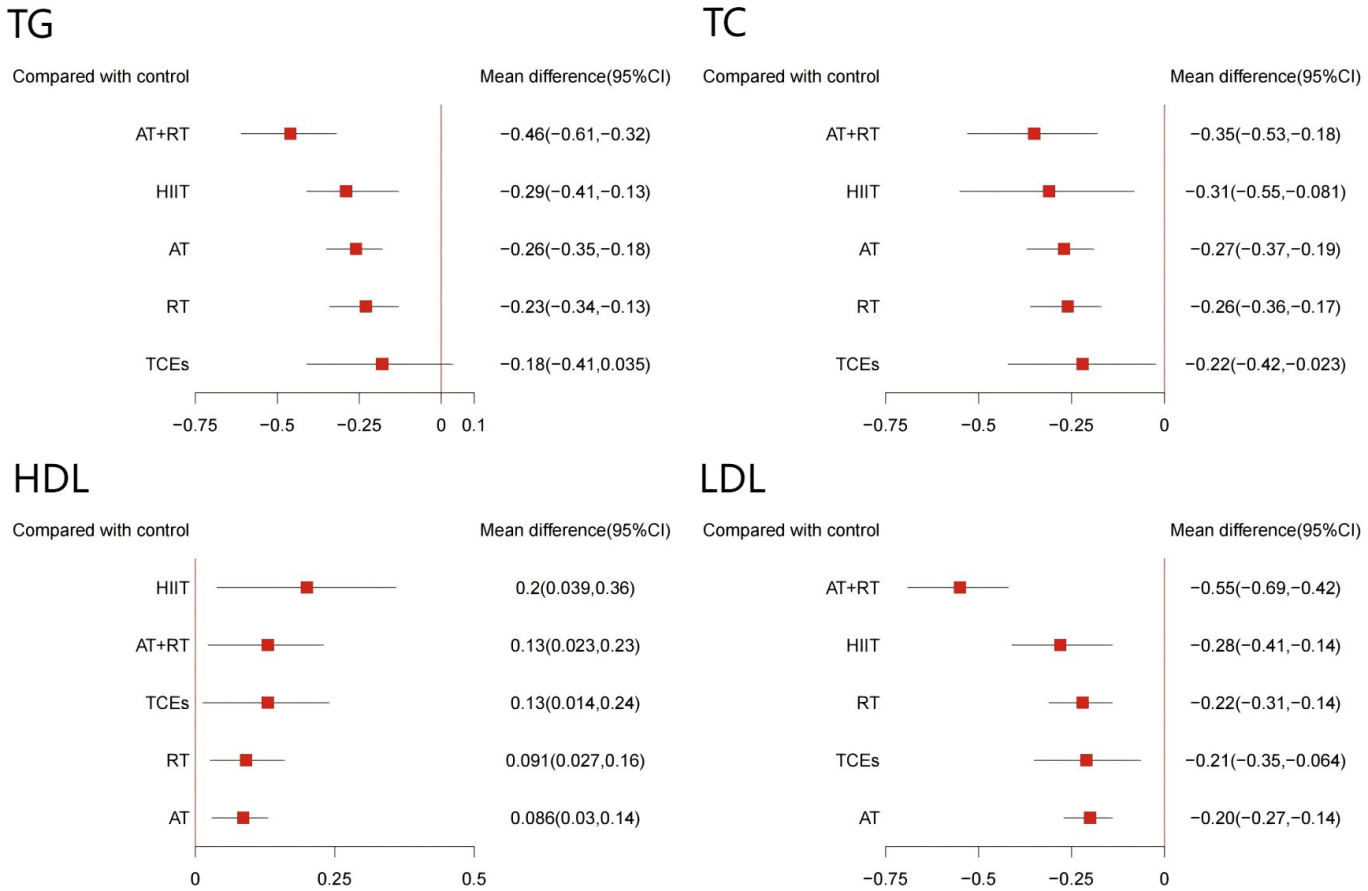
Forest plot of network effect sizes between different exercise interventions and control for blood lipids (TG, TC, HDL, and LDL).

The effects of different exercise interventions on serum lipid concentrations were evaluated using HDL, LDL, TC, and TG levels. A total of 32 studies (involving 2,868 participants) reported changes in TC, 34 studies (3,172 participants) reported changes in TG, 34 studies (3,150 participants) reported changes in HDL, and 32 studies (3,100 participants) reported changes in LDL. The results of the network meta-analysis indicated that, except for TCEs, which had no significant effect on TC, all other exercise interventions significantly improved HDL, LDL, TC, and TG levels (**Figures 7**). Among them, AT+RT was the most effective in reducing TC (-0.46, 95% CI: -0.61 to -0.32, SUCRA 98.3%, Moderate confidence of evidence), TG (-0.55, 95% CI: -0.69 to -0.42, SUCRA 99.9%, Moderate confidence of evidence), and LDL (-0.35, 95% CI: -0.53 to -0.18, SUCRA 82.2%, Low confidence of evidence). HIIT was the most effective intervention for increasing HDL (0.20, 95% CI: 0.03 to 0.36, SUCRA 87.3%, Moderate confidence of evidence). Additionally, HIIT also showed promising results in improving TC (-0.29, 95% CI: -0.41 to -0.13, SUCRA 64.9%, moderate confidence of evidence), TG (-0.28, 95% CI: -0.41 to -0.14, SUCRA 69.0%, Moderate confidence of evidence), and LDL (-0.31, 95% CI: -0.55 to -0.08, SUCRA 68.7%, Moderate confidence of evidence). AT+RT was relatively effective in increasing HDL (-0.13, 95% CI: 0.02 to 0.2, SUCRA 64.2%, low confidence of evidence). Further comparisons showed that AT+RT was significantly more effective than AT, RT, and TCEs in improving TC and TG levels and more effective than AT in improving LDL levels (**Appendix 8**, **Supplementary Tables S8.4**-**S8.7**).

The CINeMA assessment indicated that the overall quality of evidence for TC, TG, HDL, and LDL ranged from very low to moderate (**Appendix 9**, **Supplementary Figures S9.4**-**S9.7**).

### 3.5 Weight loss

A total of 23 studies (involving 1,534 participants) reported changes in BW, and another 18 studies (1,155 participants) reported changes in WC. The network meta-analysis results indicated that TCEs were the most effective intervention for reducing BW (-3.4, 95% CI: -6.4 to -0.51, SUCRA 79.1%, Very low confidence of evidence) and WC (-4.27, 95% CI: -7.53 to -0.98, SUCRA 84.6%, Low confidence of evidence) (**Appendix 6**, **Supplementary Figures S6.8**-**S6.9**).

40 studies (3,226 participants) reported changes in body mass index (BMI). Unlike the results for BW and WC, compared to the control group, AT+RT (-0.89, 95% CI: -1.6 to -0.14, SUCRA 66.4%, Very low confidence of evidence) was the most effective intervention for reducing BMI (**Appendix 6**, **Supplementary Figure S6.8**).

Our CINeMA assessment, a robust tool for evaluating evidence quality, reaffirms the reliability of our findings. The overall quality of evidence for BMI and WC, as shown in our analysis, ranged from very low to moderate, while the quality of evidence for BW was primarily very low to low (**Appendix 8**, **Supplementary Figures S9.8**-**S9.10**).

### 3.6 Subgroup analysis of different intensities of exercise interventions

To further investigate the differential effects of exercise intensity on various metabolic indicators, we conducted a subgroup analysis based on seven exercise intensity classifications. Specifically, aerobic training (AT) and resistance training (RT) were each categorized into three intensity levels: low-intensity aerobic training (LAT) and low-intensity resistance training (LRT), moderate-intensity aerobic training (MAT) and moderate-intensity resistance training (MRT), and high-intensity aerobic training (HAT) and high-intensity resistance training (HRT). Meanwhile, combined aerobic and resistance training (AT+RT) was divided into two subgroups: moderate-intensity aerobic and moderate-intensity resistance training (MAT+MRT) and moderate-intensity aerobic and high-intensity resistance training (MAT+HRT). Additionally, HIIT and TCEs were analyzed as a whole, as they could not be classified by intensity. According to our findings, HIIT demonstrated the best intervention effects in controlling HbA1c, FBG, 2hPG, and HDL levels. MAT+HRT improved TC and TG levels most effectively. HAT yielded the most significant results in lowering LDL and improving BMI. Meanwhile, TCEs showed clear advantages in reducing BW and WC (**Appendix 11**).

### 3.7 Meta-regressions

To further test the robustness of the results, we conducted a meta-regression analysis to explore potential sources of heterogeneity for all outcome measures. The factors included publication year, mean age, percentage of male participants, BMI, sample size, intervention duration, training frequency, session duration, and weekly training time. The regression analysis showed that the reduction in FBG was associated with publication year (β = -0.29; 95% CI: -0.41 to -0.17) and BMI (β = 0.24; 95% CI: 0.07 to 0.41), independent of other confounding factors. The reduction in 2hPG was related to BMI (β = 0.60; 95% CI: 0.22 to 0.98) and training frequency (β = -0.36; 95% CI: -0.63 to 0.09), independent of other confounders. The reduction in TC was associated with BMI (β = 1.58; 95% CI: -0.33 to 0.01), while reductions in LDL (β = 0.14; 95% CI: 0.01 to 0.28) and body weight (BW) (β = -3.2; 95% CI: -5.5 to -0.68) were associated with the proportion of male participants, independent of other confounders (**Appendix 12**).

Based on the regression analysis results, we performed a subgroup analysis. The results indicated that patients with lower BMI experienced more significant improvements in 2hPG and TC after exercise interventions. Similarly, higher training frequency was associated with enhanced improvements in 2hPG. In addition, exercise interventions appeared to have a more pronounced effect on LDL improvement in populations with a lower proportion of male participants. In comparison, a higher proportion of male participants was linked to more significant reductions in BW (**Appendix 14**).

### 3.8 Sensitivity analyses

To assess the robustness of our findings, we performed two types of sensitivity analyses: excluding 14 studies at high risk of bias (**Appendix 12**), and reanalyzing the data after adjusting all potential sources of heterogeneity to the median (**Appendix 13**). In both analyses, the results remained consistent with those of the primary network meta-analysis, supporting the stability of our main conclusions

## 4 Discussion

This study is the first to include HIIT and TCEs in a systematic network meta-analysis, comprehensively evaluating the efficacy of five different exercise interventions on glycemic control, lipid profile, and weight management in prediabetic patients. We found that HIIT was the most effective exercise for controlling blood glucose. HIIT showed a strong trend in improving lipid levels, particularly in increasing HDL levels, which was the most notable effect. These findings suggest that HIIT may have potential advantages in managing and preventing diabetes, warranting further exploration of its specific mechanisms and clinical value in future studies. Moreover, TCEs demonstrated certain benefits in improving glycemic control and weight management. Given the physical capabilities and safety concerns of elderly populations, we recommend traditional Chinese exercises as a moderate-intensity and effective alternative.

Our meta-analysis revealed that HIIT was the best intervention for improving glycemic control. Recent reviews have shown that HIIT is superior to continuous aerobic exercise in improving insulin resistance and blood glucose control, particularly in individuals with type 2 diabetes or those at high risk of developing the disease (42, 43). This further supports HIIT’s advantage in blood glucose regulation, suggesting that exercise intensity may be more crucial than volume in reducing HbA1c (44, 45). From a metabolic perspective, HIIT significantly increases AMP/ATP concentrations within skeletal muscle cells, accelerates glycogen breakdown, and rapidly activates AMP-activated protein kinase (AMPK) and p38 mitogen-activated protein kinase (p38 MAPK), which further enhances the expression and translocation of glucose transporter type 4 (GLUT4) on muscle cell membranes (46). Upregulation and enhancement of GLUT4 function improve glucose uptake in muscle tissues, promoting insulin sensitivity (46). Particularly during the recovery phase after exercise, muscles utilize glucose more efficiently, facilitating effective blood glucose regulation. Additionally, AMPK and p38 MAPK phosphorylate and activate PGC-1α protein, promoting mitochondrial biogenesis and lipid metabolism, further improving muscle cells’ energy metabolism efficiency (47, 48). From a cardiovascular perspective, HIIT shows more significant potential for controlling blood glucose (49). HIIT’s recovery periods allow the body to recover quickly from high-intensity stress, preventing the excessive accumulation of reactive oxygen species (ROS) (50). Compared with moderate-intensity continuous training (MCT), HIIT effectively reduces oxidative stress damage to endothelial cells by shortening the time window of ROS generation (51). Additionally, a study by Martins et al. (52) showed that, for individuals at high risk of type 2 diabetes, HIIT achieved similar improvements in HbA1c as AT+RT within a shorter training period, demonstrating its unique clinical advantages. However, for elderly or low-physical-capacity populations, high-intensity exercise may increase the risk of injury and strain the cardiovascular system, highlighting the need for safer exercise options. To address this, we included TCEs in the network meta-analysis and found that TCEs have the potential to improve glycemic and lipid metabolism in prediabetic patients. Compared with HIIT, TCEs, characterized by low intensity and emphasizing balance and flexibility, are better suited for these populations.

Interestingly, we found that TCEs had a similar effect to HIIT in improving 2hPG levels. Traditional Chinese exercises focus on slow, rhythmic movements, mental regulation, and controlled breathing, helping practitioners meditate during exercise (53). This process effectively alleviates anxiety, improves mental health, and helps reduce negative physiological responses triggered by stress (53). Studies have shown that psychological regulation also positively affects blood glucose control (54). TCEs activate the parasympathetic nervous system while inhibiting excessive sympathetic nervous activity, reducing the secretion of stress hormones such as cortisol and norepinephrine, improving insulin sensitivity, and reducing insulin resistance (55–57). At the same time, controlled breathing improves blood circulation and oxygen transport, promoting aerobic metabolism and enhancing glucose and fatty acid utilization by cells (58). In our study, AT+RT was the most effective intervention for reducing HbA1c besides HIIT. Previous studies have indicated that AT+RT is more effective in reducing HbA1c than AT or RT alone, which aligns with our findings (59, 60). The likely reason is that AT and RT improve HbA1c through different physiological mechanisms. Combining both training methods generates additional metabolic benefits through synergistic effects in different metabolic pathways, leading to more effective glycemic regulation and HbA1c improvement (61).

Most prediabetic patients also exhibit lipid abnormalities and other metabolic disorders, which may increase the risk of progression to type 2 diabetes (62, 63). Therefore, in this study, lipid metabolism indicators were analyzed as secondary outcomes to provide a more comprehensive assessment of metabolic characteristics in prediabetes. Our meta-analysis results indicated that AT+RT was the most effective intervention for improving lipid profiles, particularly in reducing TC, TG, and LDL levels, consistent with the findings of Zhang (24) and Schwingshackl (23). The superiority of AT+RT is biologically plausible. RT improves lipid metabolism primarily through activation of the Akt/mTOR pathway, thereby enhancing protein synthesis and promoting lipid clearance (64). In contrast, AT activates the AMPK–PGC-1α signaling axis, which enhances mitochondrial function and fatty acid oxidation (65). Importantly, evidence indicates that AT+RT does not impair anabolic signaling. Phosphorylation of Akt and the fractional synthesis rate (FSR) of myofibrillar proteins following concurrent training are comparable to RT alone, suggesting preserved hypertrophic responses (66). More critically, a synergistic effect appears to occur in mitochondrial adaptation. Compared to either modality alone, AT+RT may further enhance mitochondrial biogenesis in skeletal muscle, particularly when RT precedes AT. This phenomenon may be influenced by the sequence-dependent activation of mTOR and PGC-1α pathways (67). These findings support the superior metabolic and lipid profile benefits observed with AT+RT.

Regarding HDL, HIIT showed the most significant intervention effect. The superior effectiveness of HIIT may be attributed to exercise intensity as a critical determinant of energy substrate utilization during exercise (68). HIIT, compared to moderate-intensity continuous training, more effectively enhances adipocyte function and improves glycogen breakdown, thereby reducing glycogen conversion into fat. Although HIIT excelled in improving lipid metabolism, we did not observe notable advantages in reducing body weight and waist circumference. This may be because changes in body weight depend primarily on overall energy expenditure rather than solely on fat oxidation (69). Additionally, HIIT offers greater efficiency in the dose-response relationship by shortening the required exercise time to achieve the same effect, making it more sustainable for long-term adherence in prediabetic populations (70). However, one meta-analysis reported adverse events in 34% of the included studies, with most related to high-intensity interval training, primarily musculoskeletal injuries, whereas moderate-intensity exercise resulted in fewer injuries (42). Lee et al (17). found that physical function limitations are common among older adults with prediabetes. Among prediabetic patients aged ≥53 years, 32% had activity limitations, 56% had lower limb restrictions, over one-third reported chronic pain, and one-quarter required treatment for arthritis. Thus, in clinical practice, exercise intervention should be personalized based on the patient’s functional status and comorbidities. Moreover, exercise intensity and total energy expenditure play a crucial role in the lipid-improving effects of aerobic exercise. Moderate-intensity aerobic exercise has been shown to significantly increase HDL levels (71). A previous meta-analysis confirmed that, compared to other exercise modalities, moderate-intensity aerobic training, due to its relatively higher energy expenditure, effectively increases adiponectin levels in both prediabetic and diabetic adults (72). Adiponectin, in turn, promotes the hepatic uptake of HDL cholesterol and facilitates reverse cholesterol transport (73), a mechanism that is considered potentially important for preventing cardiovascular risks associated with diabetes and metabolic disorders (74). Additionally, adiponectin activates the AMPK pathway via its receptors, which further enhances glucose uptake and fatty acid oxidation, thereby improving insulin sensitivity (75). However, the improvement of LDL may require higher-intensity aerobic exercise (71). Our subgroup analysis yielded similar findings, as HAT was superior in reducing LDL levels, while low-to-moderate intensity TCEs demonstrated a better trend in increasing HDL levels. In Ma et al (76).’s study, TCEs and AT had similar overall effects. However, TCEs outperformed AT in regulating HDL levels in prediabetic patients, while AT was more effective in reducing HbA1c, which aligns with our results. However, Ma et al. noted that the glycemic improvement effect of TCEs appeared later than that of AT. This observation was supported by Yu et al. (20), who found that TCEs required more than six months to achieve significant glycemic improvement. However, only one study in the subgroup analysis on HbA1c involved a three-month intervention period, which may not fully capture the short-term effects, and this result should be interpreted with caution. Our meta-regression analysis revealed no significant correlation between exercise intervention duration and improvements in glycemic and lipid outcomes, suggesting that short-term TCEs may also confer significant health benefits. This finding aligns with Dong et al.’s study (19), which similarly found that short-term TCEs significantly improved glycemic and lipid indicators, further validating our conclusions.

Previous meta-analyses (19, 20, 77, 78) on TCEs did not include BW, BMI, and WC as evaluation metrics despite the significant importance of these factors in assessing the overall metabolic health and cardiovascular risk of prediabetic patients (79). Our meta-analysis found that TCEs were the most effective intervention for reducing BW and WC. Unlike other exercise modalities, traditional forms of exercise not only emphasize physical movement but also psychological well-being, which has been linked to improved sleep quality, emotional stability, and stress regulation. These factors may contribute to weight management by lowering cortisol levels and reducing stress-induced overeating (80). However, such traditional exercise practices often occur within a broader lifestyle context, where individuals may gradually adjust their dietary habits or adopt healthier behaviors over time. This is particularly relevant in Eastern cultures, where traditional exercise habits are closely associated with comprehensive lifestyle changes, including dietary improvements. Additionally, the number of AT+RT studies included in our analysis was limited, and none reported WC or BW data. Therefore, while our findings highlight the potential benefits of TCEs in weight management, these results should be interpreted with caution, and future studies should incorporate comprehensive lifestyle assessments. Beyond weight management, the suitability of different exercise modalities for prediabetic individuals is also an important consideration. In clinical trials such as the DPP (10) and the Finnish Diabetes Prevention Study (81), adults with multiple comorbidities and functional limitations were excluded, as these conditions might interfere with their participation in the interventions. Thus, the trial results may not fully represent the needs of these high-risk populations (82). Even though most lifestyle interventions involve moderate-intensity aerobic exercises such as jogging, cycling, or brisk walking, prediabetic individuals may find it challenging to engage in such activities. Additionally, certain types of aerobic exercises, such as jogging, while beneficial for cardiovascular and respiratory health, may be challenging to manage in terms of intensity and volume and are unsuitable for frail elderly individuals or those with chronic illnesses. Therefore, as a gentle, safe, and sustainable exercise intervention, TCEs may offer fitness and health maintenance advantages that other aerobic exercises cannot achieve. From a public health perspective, TCEs also hold significant potential for promoting diabetes care and prevention within community settings (77).

### 4.1 Strengths and limitations

To our knowledge, this study is the most comprehensive and up-to-date systematic review and network meta-analysis evaluating the effects of various exercise modalities, including HIIT and TCEs, on adults with prediabetes. These exercise interventions are recommended by the ADA and the ACSM as part of diabetes management and health risk reduction strategies (21). We employed the CINeMA framework for quality assessment, ensuring the credibility of the study results. Additionally, we conducted regression analyses on several key variables to examine the influence of potential moderating factors on exercise outcomes, further enhancing the generalizability of the findings. Finally, sensitivity analyses were performed, adjusting all potential sources of heterogeneity to the median and reanalyzing the data, thereby verifying the robustness of the conclusions.

However, this study has several limitations. First, the quality of the included studies was often rated as a moderate risk of bias, with the certainty of evidence ranging from very low to moderate. Many of the included RCTs did not report allocation concealment or blinding of participants, making it difficult to assess the risk of bias. This inadequate reporting may have introduced selective bias in the study design and implementation. Although we conducted sensitivity analyses excluding studies at high risk of bias, and found that the main results remained robust, the overall risk of bias across studies was moderate to high, and our findings should therefore be interpreted with caution. Second, the number of studies involving AT+RT, HIIT, and TCEs was limited, which imposes certain restrictions on the reliability and generalizability of our findings. Larger, well-designed randomized controlled trials in high-risk populations are needed to further validate the efficacy and safety of these interventions. Third, most studies on TCEs did not describe exercise intensity in their designs. Although TCEs are generally classified as low- to moderate-intensity exercises, this study’s lack of specific intensity data limits further exploration of the optimal exercise intensity for prediabetic patients undergoing TCEs interventions. Future research should report standardized intensity metrics, such as metabolic equivalents (METs) or heart rate zones, to enhance comparability and reproducibility across studies. Fourth, in this meta-analysis, we found that most of the included studies did not categorize the different subtypes of prediabetes (IGT, IFG, or HbA1c). Additionally, some studies included participants based on only one of these criteria, which could have led to the inclusion of subjects with other undiagnosed glycemic abnormalities. This subtype ambiguity may have contributed to the confounding of results, affecting the precise evaluation of the effects of different exercise interventions. Due to these factors, this study could not conduct a subgroup analysis of different prediabetes subtypes, potentially limiting the understanding of how each subtype responds to exercise interventions. Future studies should ensure transparent classification and detailed reporting of prediabetes subtypes, and may also explore the integration of continuous glucose monitoring with artificial intelligence (CGM-AI) to identify dynamic glycaemic patterns and latent metabolic sub-phenotypes, thereby informing more precise and mechanism-based personalized exercise prescriptions (83).

## 5 Conclusions

In this comprehensive meta-analysis, we confirmed the effectiveness of various exercise interventions in improving glycemic and lipid control in prediabetic patients. Evidence of very low to moderate quality suggests that HIIT and AT+RT are likely the most effective exercise modalities for enhancing glucose and lipid metabolism in prediabetic individuals. For elderly patients with limited physical activity, reduced exercise capacity, or multiple comorbidities, we recommend moderate TCEs as the preferred exercise option. Our study provides the latest evidence to optimize exercise intervention strategies for prediabetic patients, offering a scientific basis for future clinical applications and guideline development.

## Data Availability

The original contributions presented in the study are included in the article/**Supplementary Material**. Further inquiries can be directed to the corresponding author/s.

## References

[1] Tabák AG Herder C Rathmann W Brunner EJ Kivimäki M . Prediabetes: a high-risk state for diabetes development. Lancet Lond Engl. (2012) 379:2279–90. doi: 10.1016/S0140-6736(12)60283-9, PMID: 22683128 PMC3891203

[2] Saeedi P Petersohn I Salpea P Malanda B Karuranga S Unwin N . Global and regional diabetes prevalence estimates for 2019 and projections for 2030 and 2045: Results from the international diabetes federation diabetes atlas, 9th edition. Diabetes Res Clin Pract. (2019) 157:107843. doi: 10.1016/j.diabres.2019.107843, PMID: 31518657

[3] Schlesinger S Neuenschwander M Barbaresko J Lang A Maalmi H Rathmann W . Prediabetes and risk of mortality, diabetes-related complications and comorbidities: Umbrella review of meta-analyses of prospective studies. Diabetologia. (2022) 65:275–85. doi: 10.1007/s00125-021-05592-3, PMID: 34718834 PMC8741660

[4] Hu D Fu P Xie J Chen C-S Yu D Whelton PK . Increasing prevalence and low awareness, treatment and control of diabetes mellitus among Chinese adults: the InterASIA study. Diabetes Res Clin Pract. (2008) 81:250–7. doi: 10.1016/j.diabres.2008.04.008, PMID: 18495287

[5] Echouffo-Tcheugui JB Dagogo-Jack S . Preventing diabetes mellitus in developing countries. Nat Rev Endocrinol. (2012) 8:557–62. doi: 10.1038/nrendo.2012.46, PMID: 22488646

[6] Haw JS Galaviz KI Straus AN Kowalski AJ Magee MJ Weber MB . Long-term sustainability of diabetes prevention approaches. JAMA Intern Med. (2017) 177:1808–17. doi: 10.1001/jamainternmed.2017.6040, PMID: 29114778 PMC5820728

[7] Huang Y Cai X Mai W Li M Hu Y . Association between prediabetes and risk of cardiovascular disease and all cause mortality: systematic review and meta-analysis. BMJ. (2016) 355:i5953. doi: 10.1136/bmj.i5953, PMID: 27881363 PMC5121106

[8] Diabetes Prevention Program Research Group Knowler WC Fowler SE Hamman RF Christophi CA Hoffman HJ . 10-year follow-up of diabetes incidence and weight loss in the Diabetes Prevention Program Outcomes Study. Lancet Lond Engl. (2009) 374:1677–86. doi: 10.1016/S0140-6736(09)61457-4, PMID: 19878986 PMC3135022

[9] DREAM (Diabetes REduction Assessment with ramipril and rosiglitazone Medication) Trial Investigators Gerstein HC Yusuf S Bosch J Pogue J Sheridan P . Effect of rosiglitazone on the frequency of diabetes in patients with impaired glucose tolerance or impaired fasting glucose: a randomised controlled trial. Lancet Lond Engl. (2006) 368:1096–105. doi: 10.1016/S0140-6736(06)69420-8, PMID: 16997664

[10] Knowler WC Barrett-Connor E Fowler SE Hamman RF Lachin JM Walker EA . Reduction in the incidence of type 2 diabetes with lifestyle intervention or metformin. N Engl J Med. (2002) 346:393–403. doi: 10.1056/NEJMoa012512, PMID: 11832527 PMC1370926

[11] Gong Q Zhang P Wang J Ma J An Y Chen Y . Morbidity and mortality after lifestyle intervention for people with impaired glucose tolerance: 30-year results of the Da Qing Diabetes Prevention Outcome Study. Lancet Diabetes Endocrinol. (2019) 7:452–61. doi: 10.1016/S2213-8587(19)30093-2, PMID: 31036503 PMC8172050

[12] Diabetes Prevention Program (DPP) Research Group . The Diabetes Prevention Program (DPP): description of lifestyle intervention. Diabetes Care. (2002) 25:2165–71. doi: 10.2337/diacare.25.12.2165, PMID: 12453955 PMC1282458

[13] International Diabetes Federation Guideline Development Group . Global guideline for type 2 diabetes. Diabetes Res Clin Pract (2014) 104:1–52. doi: 10.1016/j.diabres.2012.10.001, PMID: 24508150

[14] Rydén L Grant PJ Anker SD Berne C Cosentino F Danchin N . ESC Guidelines on diabetes, pre-diabetes, and cardiovascular diseases developed in collaboration with the EASD: the Task Force on diabetes, pre-diabetes, and cardiovascular diseases of the European Society of Cardiology (ESC) and developed in collaboration with the European Association for the Study of Diabetes (EASD). Eur Heart J. (2013) 34:3035–87. doi: 10.1093/eurheartj/eht108, PMID: 23996285

[15] Mendes R Sousa N Almeida A Subtil P Guedes-Marques F Reis VM . Exercise prescription for patients with type 2 diabetes—a synthesis of international recommendations: narrative review. Br J Sports Med. (2016) 50:1379–81. doi: 10.1136/bjsports-2015-094895, PMID: 26719499

[16] Conn VS Minor MA Burks KJ Rantz MJ Pomeroy SH . Integrative review of physical activity intervention research with aging adults. J Am Geriatr. Soc. (2003) 51:1159–68. doi: 10.1046/j.1532-5415.2003.51365.x, PMID: 12890083

[17] Lee PG Cigolle CT Ha J Min L Murphy SL Blaum CS . Physical function limitations among middle-aged and older adults with prediabetes: one exercise prescription may not fit all. Diabetes Care. (2013) 36:3076–83. doi: 10.2337/dc13-0412, PMID: 23757432 PMC3781567

[18] Gibala MJ Little JP Macdonald MJ Hawley JA . Physiological adaptations to low-volume, high-intensity interval training in health and disease. J Physiol. (2012) 590:1077–84. doi: 10.1113/jphysiol.2011.224725, PMID: 22289907 PMC3381816

[19] Dong C Liu R Li R Huang Z Sun S . Effects of traditional chinese exercises on glycemic control in patients with type 2 diabetes mellitus: A systematic review and meta-Analysis of randomized controlled trials. Sports Med. (2024) 54:2327–55. doi: 10.1007/s40279-024-02046-9, PMID: 38874898

[20] Yu D You L Huang W Cao H Wang F Tang X . Effects of traditional Chinese exercises on blood glucose and hemoglobin A1c levels in patients with prediabetes: A systematic review and meta-analysis. J Integr Med. (2020) 18:292–302. doi: 10.1016/j.joim.2020.04.003, PMID: 32534937

[21] Kanaley JA Colberg SR Corcoran MH Malin SK Rodriguez NR Crespo CJ . Exercise/physical activity in individuals with type 2 diabetes: A consensus statement from the american college of sports medicine. Med Sci Sports Exerc. (2022) 54:353–68. doi: 10.1249/MSS.0000000000002800, PMID: 35029593 PMC8802999

[22] Pan B Ge L Xun Y Chen Y Gao C Han X . Exercise training modalities in patients with type 2 diabetes mellitus: a systematic review and network meta-analysis. Int J Behav Nutr Phys Act. (2018) 15:72. doi: 10.1186/s12966-018-0703-3, PMID: 30045740 PMC6060544

[23] Schwingshackl L Missbach B Dias S König J Hoffmann G . Impact of different training modalities on glycaemic control and blood lipids in patients with type 2 diabetes: A systematic review and network meta-analysis. Diabetologia. (2014) 57:1789–97. doi: 10.1007/s00125-014-3303-z, PMID: 24996616

[24] Zhang H Guo Y Hua G Guo C Gong S Li M . Exercise training modalities in prediabetes: a systematic review and network meta-analysis. Front Endocrinol. (2024) 15:1308959. doi: 10.3389/fendo.2024.1308959, PMID: 38440785 PMC10911289

[25] Huang L Fang Y Tang L . Comparisons of different exercise interventions on glycemic control and insulin resistance in prediabetes: a network meta-analysis. BMC Endocr. Disord. (2021) 21:181. doi: 10.1186/s12902-021-00846-y, PMID: 34488728 PMC8422751

[26] Bennasar-Veny M Malih N Galmes-Panades AM Hernandez-Bermudez IC Garcia-Coll N Ricci-Cabello I . Effect of physical activity and different exercise modalities on glycemic control in people with prediabetes: a systematic review and meta-analysis of randomized controlled trials. Front Endocrinol. (2023) 14:1233312. doi: 10.3389/fendo.2023.1233312, PMID: 37842295 PMC10569497

[27] Page MJ McKenzie JE Bossuyt PM Boutron I Hoffmann TC Mulrow CD . The PRISMA 2020 statement: An updated guideline for reporting systematic reviews. BMJ. (2021) 372:n71. doi: 10.1136/bmj.n71, PMID: 33782057 PMC8005924

[28] Hutton B Salanti G Caldwell DM Chaimani A Schmid CH Cameron C . The PRISMA extension statement for reporting of systematic reviews incorporating network meta-analyses of health care interventions: checklist and explanations. Ann Intern Med. (2015) 162:777–84. doi: 10.7326/M14-2385, PMID: 26030634

[29] Corrao S Colomba D Arnone S Argano C Di Chiara T Scaglione R . Improving efficacy of PubMed clinical queries for retrieving scientifically strong studies on treatment. J Am Med Inform Assoc JAMIA. (2006) 13:485–7. doi: 10.1197/jamia.M2084, PMID: 16799123 PMC1561795

[30] Liberati A Altman DG Tetzlaff J Mulrow C Gøtzsche PC Ioannidis JPA . The PRISMA statement for reporting systematic reviews and meta-analyses of studies that evaluate healthcare interventions: explanation and elaboration. BMJ. (2009) 339:b2700. doi: 10.1136/bmj.b2700, PMID: 19622552 PMC2714672

[31] Expert Committee on the Diagnosis and Classification of Diabetes Mellitus . Report of the expert committee on the diagnosis and classification of diabetes mellitus. Diabetes Care. (2003) 26 Suppl 1:S5–20. doi: 10.2337/diacare.26.2007.s5, PMID: 12502614

[32] Editor HJ . Cochrane handbook for systematic reviews of interventions. Httpwwwcochrane-Handbookorg. (2008). Available online at: http://www.cochrane-Handbookorg. (Accessed November 17, 2024).

[33] Sterne JAC Savović J Page MJ Elbers RG Blencowe NS Boutron I . RoB 2: A revised tool for assessing risk of bias in randomised trials. BMJ. (2019) 366:l4898. doi: 10.1136/bmj.l4898, PMID: 31462531

[34] Nikolakopoulou A Higgins JPT Papakonstantinou T Chaimani A Del Giovane C Egger M . CINeMA: An approach for assessing confidence in the results of a network meta-analysis. PloS Med. (2020) 17:e1003082. doi: 10.1371/journal.pmed.1003082, PMID: 32243458 PMC7122720

[35] Papakonstantinou T Nikolakopoulou A Higgins JPT Egger M Salanti G . CINeMA: Software for semiautomated assessment of the confidence in the results of network meta-analysis. Campbell Syst Rev. (2020) 16:e1080. doi: 10.1002/cl2.v16.1, PMID: 37131978 PMC8356302

[36] Welton NJ Sutton AJ Cooper N Abrams KR Ades AE . Evidence synthesis for decision making in healthcare Vol. 132. Chichester, UK: John Wiley & Sons (2012).

[37] Mavridis D Salanti G . A practical introduction to multivariate meta-analysis. Stat Methods Med Res. (2013) 22:133–58. doi: 10.1177/0962280211432219, PMID: 22275379

[38] Turner RM Davey J Clarke MJ Thompson SG Higgins JP . Predicting the extent of heterogeneity in meta-analysis, using empirical data from the cochrane database of systematic reviews. Int J Epidemiol. (2012) 41:818–27. doi: 10.1093/ije/dys041, PMID: 22461129 PMC3396310

[39] da Costa BR . & Juni, P. Systematic reviews and meta-analyses of randomized trials: Principles and pitfalls. Eur Heart J. (2014) 35:3336–45. doi: 10.1093/eurheartj/ehu424, PMID: 25416325

[40] Dias S Welton NJ Sutton AJ Caldwell DM Lu G Ades AE . Evidence synthesis for decision making 4: Inconsistency in networks of evidence based on randomized controlled trials. Med Decis. Mak. Int J Soc Med Decis. Mak. (2013) 33:641–56. doi: 10.1177/0272989X12455847, PMID: 23804508 PMC3704208

[41] Mbuagbaw L Rochwerg B Jaeschke R Heels-Andsell D Alhazzani W Thabane L . Approaches to interpreting and choosing the best treatments in network meta-analyses. Syst Rev. (2017) 6:79. doi: 10.1186/s13643-017-0473-z, PMID: 28403893 PMC5389085

[42] Jelleyman C Yates T O’Donovan G Gray LJ King JA Khunti K . The effects of high-intensity interval training on glucose regulation and insulin resistance: a meta-analysis. Obes Rev Off J Int Assoc Study Obes. (2015) 16:942–61. doi: 10.1111/obr.2015.16.issue-11, PMID: 26481101

[43] Grace A Chan E Giallauria F Graham PL Smart NA . Clinical outcomes and glycaemic responses to different aerobic exercise training intensities in type II diabetes: a systematic review and meta-analysis. Cardiovasc Diabetol. (2017) 16:37. doi: 10.1186/s12933-017-0518-6, PMID: 28292300 PMC5351065

[44] Boulé NG Kenny GP Haddad E Wells GA Sigal RJ . Meta-analysis of the effect of structured exercise training on cardiorespiratory fitness in type 2 diabetes mellitus. Diabetologia. (2003) 46:1071–81. doi: 10.1007/s00125-003-1160-2, PMID: 12856082

[45] Safarimosavi S Mohebbi H Rohani H . High-Intensity interval vs. Continuous endurance training: preventive effects on hormonal changes and physiological adaptations in prediabetes patients. J Strength Cond. Res. (2021) 35:731–8. doi: 10.1519/JSC.0000000000002709, PMID: 29939900

[46] Hood MS Little JP Tarnopolsky MA Myslik F Gibala MJ . Low-volume interval training improves muscle oxidative capacity in sedentary adults. Med Sci Sports Exerc. (2011) 43:1849–56. doi: 10.1249/MSS.0b013e3182199834, PMID: 21448086

[47] Little JP Safdar A Bishop D Tarnopolsky MA Gibala MJ . An acute bout of high-intensity interval training increases the nuclear abundance of PGC-1α and activates mitochondrial biogenesis in human skeletal muscle. Am J Physiol Regul Integr Comp Physiol. (2011) 300:R1303–1310. doi: 10.1152/ajpregu.00538.2010, PMID: 21451146

[48] Valle I Alvarez-Barrientos A Arza E Lamas S Monsalve M . PGC-1alpha regulates the mitochondrial antioxidant defense system in vascular endothelial cells. Cardiovasc Res. (2005) 66:562–73. doi: 10.1016/j.cardiores.2005.01.026, PMID: 15914121

[49] Magalhães JP Melo X Correia IR Ribeiro RT Raposo J Dores H . Effects of combined training with different intensities on vascular health in patients with type 2 diabetes: a 1-year randomized controlled trial. Cardiovasc Diabetol. (2019) 18:34. doi: 10.1186/s12933-019-0840-2, PMID: 30885194 PMC6423850

[50] Green DJ Hopman MTE Padilla J Laughlin MH Thijssen DHJ . Vascular adaptation to exercise in humans: role of hemodynamic stimuli. Physiol Rev. (2017) 97:495–528. doi: 10.1152/physrev.00014.2016, PMID: 28151424 PMC5539408

[51] Ramos JS Dalleck LC Tjonna AE Beetham KS Coombes JS . The impact of high-intensity interval training versus moderate-intensity continuous training on vascular function: a systematic review and meta-analysis. Sports Med Auckl. NZ. (2015) 45:679–92. doi: 10.1007/s40279-015-0321-z, PMID: 25771785

[52] Martins F Souza A Nunes P Michelin M Murta E Resende E . High-intensity body weight training is comparable to combined training in changes in muscle mass, physical performance, inflammatory markers and metabolic health in postmenopausal women at high risk for type 2 diabetes mellitus: A randomized controlled clinical trial. Exp Gerontol. (2018) 107:108–15. doi: 10.1016/j.exger.2018.02.016, PMID: 29471132

[53] Jahnke R Larkey L Rogers C Etnier J Lin F . A comprehensive review of health benefits of qigong and tai chi. Am J Health Promot. AJHP. (2010) 24:e1–e25. doi: 10.4278/ajhp.081013-LIT-248, PMID: 20594090 PMC3085832

[54] American Diabetes Association . 5. Lifestyle management: standards of medical care in diabetes-2019. Diabetes Care. (2019) 42:S46–60. doi: 10.2337/dc19-S005, PMID: 30559231

[55] Paul-Labrador M Polk D Dwyer JH Velasquez I Nidich S Rainforth M . Effects of a randomized controlled trial of transcendental meditation on components of the metabolic syndrome in subjects with coronary heart disease. Arch Intern Med. (2006) 166:1218–24. doi: 10.1001/archinte.166.11.1218, PMID: 16772250

[56] Chen XY . The dose-effect relationship of baduanjin assisted interventions in type 2 diabetes and explore the mechanism of inquiry. Nanjing Univ Chin Med. (2014).

[57] Zhang S WJ . Network meta-analysis of the effects of different ChineseTR aditional fitness exercises on blood lipid. Cap. Instit Phys Educ. (2022) 34:545–54. doi: 10.14036/j.cnki.cn11-4513.2022.05.010

[58] Li Q Ni Q Wu R Xie B Xu H Zhang Q . Application of traditional Chinese medicine exercise in diabetes prevention and treatment. World Chin Med. (2020) 5:3355–8.

[59] Church TS Blair SN Cocreham S Johannsen N Johnson W Kramer K . Effects of aerobic and resistance training on hemoglobin A1c levels in patients with type 2 diabetes: a randomized controlled trial. JAMA. (2010) 304:2253–62. doi: 10.1001/jama.2010.1710, PMID: 21098771 PMC3174102

[60] Sigal RJ Kenny GP Boulé NG Wells GA Prud’homme D Fortier M . Effects of aerobic training, resistance training, or both on glycemic control in type 2 diabetes: a randomized trial. Ann Intern Med. (2007) 147:357–69. doi: 10.7326/0003-4819-147-6-200709180-00005, PMID: 17876019

[61] Jorge MLMP de Oliveira VN Resende NM Paraiso LF Calixto A Diniz ALD . The effects of aerobic, resistance, and combined exercise on metabolic control, inflammatory markers, adipocytokines, and muscle insulin signaling in patients with type 2 diabetes mellitus. Metabolism. (2011) 60:1244–52. doi: 10.1016/j.metabol.2011.01.006, PMID: 21377179

[62] Neves JS Newman C Bostrom JA Buysschaert M Newman JD Medina JL . Management of dyslipidemia and atherosclerotic cardiovascular risk in prediabetes. Diabetes Res Clin Pract. (2022) 190. doi: 10.1016/j.diabres.2022.109980, PMID: 35787415

[63] Bloomgarden Z Chilton R . Lipids as risk markers for type 2 diabetes. J Diabetes. (2019) 11:176–8. doi: 10.1111/jdb.2019.11.issue-3 30479008

[64] Dreyer HC Fujita S Cadenas JG Chinkes DL Volpi E Rasmussen BB . Resistance exercise increases AMPK activity and reduces 4E-BP1 phosphorylation and protein synthesis in human skeletal muscle. J Physiol. (2006) 576:613–24. doi: 10.1113/jphysiol.2006.113175, PMID: 16873412 PMC1890364

[65] Mendham AE Goedecke JH Zeng Y Larsen S George C Hauksson J . Exercise training improves mitochondrial respiration and is associated with an altered intramuscular phospholipid signature in women with obesity. Diabetologia. (2021) 64:1642–59. doi: 10.1007/s00125-021-05430-6, PMID: 33770195 PMC8187207

[66] Donges CE Burd NA Duffield R Smith GC West DWD Short MJ . Concurrent resistance and aerobic exercise stimulates both myofibrillar and mitochondrial protein synthesis in sedentary middle-aged men. J Appl Physiol. (2012) 112:1992–2001. doi: 10.1152/japplphysiol.00166.2012, PMID: 22492939

[67] Zhao Y-C Gao B . Integrative effects of resistance training and endurance training on mitochondrial remodeling in skeletal muscle. Eur J Appl Physiol. (2024) 124:2851–65. doi: 10.1007/s00421-024-05549-5, PMID: 38981937

[68] Støa EM Meling S Nyhus L-K Strømstad G Mangerud KM Helgerud J . High-intensity aerobic interval training improves aerobic fitness and HbA1c among persons diagnosed with type 2 diabetes. Eur J Appl Physiol. (2017) 117:455–67. doi: 10.1007/s00421-017-3540-1, PMID: 28160083

[69] Cowan TE Brennan AM Stotz PJ Clarke J Lamarche B Ross R . Separate effects of exercise amount and intensity on adipose tissue and skeletal muscle mass in adults with abdominal obesity. Obes Silver Spring Md. (2018) 26:1696–703. doi: 10.1002/oby.2018.26.issue-11, PMID: 30261125

[70] Buchheit M High-intensity interval training PB . solutions to the programming puzzle. Sports Med. (2013) 43:313–38. doi: 10.1007/s40279-013-0029-x, PMID: 23539308

[71] Mann S Beedie C Jimenez A . Differential effects of aerobic exercise, resistance training and combined exercise modalities on cholesterol and the lipid profile: review, synthesis and recommendations. Sports Med Auckl. NZ. (2014) 44:211–21. doi: 10.1007/s40279-013-0110-5, PMID: 24174305 PMC3906547

[72] Becic T Studenik C Hoffmann G . Exercise increases adiponectin and reduces leptin levels in prediabetic and diabetic individuals: Systematic review and meta-analysis of randomized controlled trials. Med Sci. (2018) 6:97. doi: 10.3390/medsci6040097, PMID: 30380802 PMC6318757

[73] Abdalla MMI . Therapeutic potential of adiponectin in prediabetes: Strategies, challenges, and future directions. Ther Adv Endocrinol Metab. (2024) 15:20420188231222371. doi: 10.1177/20420188231222371, PMID: 38250316 PMC10798122

[74] Di Chiara T Licata A Argano C Duro G Corrao S Scaglione R . Plasma adiponectin: A contributing factor for cardiac changes in visceral obesity-associated hypertension. Blood Press. (2014) 23:147–53. doi: 10.3109/08037051.2013.823767, PMID: 24011171

[75] Krause MP Milne KJ Hawke TJ . Adiponectin—consideration for its role in skeletal muscle health. Int J Mol Sci. (2019) 20:1528. doi: 10.3390/ijms20071528, PMID: 30934678 PMC6480271

[76] Ma X Li M Liu L Lei F Wang L Xiao W . A randomized controlled trial of Baduanjin exercise to reduce the risk of atherosclerotic cardiovascular disease in patients with prediabetes. Sci Rep. (2022) 12:19338. doi: 10.1038/s41598-022-22896-5, PMID: 36369247 PMC9651897

[77] Song G Chen C Zhang J Chang L Zhu D Wang X . Association of traditional Chinese exercises with glycemic responses in people with type 2 diabetes: A systematic review and meta-analysis of randomized controlled trials. J Sport Health Sci. (2018) 7:442–52. doi: 10.1016/j.jshs.2018.08.004, PMID: 30450253 PMC6226554

[78] Gao Y Yu L Li X Yang C Wang A Huang H . The effect of different traditional chinese exercises on blood lipid in middle-aged and elderly individuals: A systematic review and network meta-analysis. Life. (2021) 11:714. doi: 10.3390/life11070714, PMID: 34357085 PMC8305451

[79] Lavie CJ Ozemek C Carbone S Katzmarzyk PT Blair SN . Sedentary behavior, exercise, and cardiovascular health. Circ Res. (2019) 124:799–815. doi: 10.1161/CIRCRESAHA.118.312669, PMID: 30817262

[80] Xiong X Wang P Li S Zhang Y Li X . Effect of baduanjin exercise for hypertension: A systematic review and meta-analysis of randomized controlled trials. Maturitas. (2015) 80:370–8. doi: 10.1016/j.maturitas.2015.01.002, PMID: 25636242

[81] Tuomilehto J Lindström J Eriksson JG Valle TT Hamäläinen H Ianne-Parikka P . Prevention of type 2 diabetes mellitus by changes in lifestyle among subjects with impaired glucose tolerance. N Engl J Med. (2001) 344:1343–50. doi: 10.1056/NEJM200105033441801, PMID: 11333990

[82] Orozco LJ Buchleitner AM Gimenez-Perez G Roquéi Figuls M Richter B Mauricio D . Exercise or exercise and diet for preventing type 2 diabetes mellitus. Cochrane Database Syst Rev. (2008) CD003054. doi: 10.1002/14651858.CD003054.pub3, PMID: 18646086

[83] Ji C Jiang T Liu L Zhang J You L . Continuous glucose monitoring combined with artificial intelligence: Redefining the pathway for prediabetes management. Front Endocrinol. (2025) 16. doi: 10.3389/fendo.2025.1571362, PMID: 40491592 PMC12146165

