# Supplementary material for: Comparative effectiveness of different exercise modality on glycaemic control and lipid profile for prediabetes: systematic review and network meta-analysis
Source: Front Endocrinol (Lausanne). 2025 Jul 24;16:1518871. doi: 10.3389/fendo.2025.1518871 (PMC12328182; doi:10.3389/fendo.2025.1518871)
Supplement: Supplementary file 1 [file DataSheet1.docx]

Supplementary Appendix

**Comparative effectiveness of different exercise modality on glycaemic control and lipid profile for prediabetes: systematic review and network meta-analysis**

**Table of contents**

*[Appendix 1: Search strategy 2](#_Toc177311842)*

*[Appendix 2: Definitions of physical activity types and non-exercise training control 8](#_Toc177311843)*

*[Appendix 3: Characteristics of included studies 9](#_Toc177311844)*

*[Appendix 4: Risk of bias of randomized clinical trials 17](#_Toc177311845)*

*[Appendix 5: Evaluation of inconsistency and heterogeneity 20](#_Toc177311846)*

*[Appendix 6: Network maps and forest plots of secondary outcomes 24](#_Toc177311847)*

*[Appendix 7: SUCRA and cumulative probability plots 33](#_Toc177311848)*

*[Appendix 8: league table of Summary Estimates for different exercise modality on prediabetes Derived from Network Meta-analysis 42](#_Toc177311849)*

*[Appendix 9: CINeMA Assessment 47](#_Toc177311850)*

*[Appendix 10: Funnel plots 61](#_Toc177311851)*

*[Appendix 11: Subgroup analysis of different intensities of exercise interventions on prediabetes 66](#_Toc177311852)*

*[Appendix12: Network Meta-Regression--changes in heterogeneity 84](#_Toc177311853)*

*[Appendix 13: subgroups analysis 130](#_Toc177311854)*

# Appendix 1: Search strategy

**Table S1.** Search strategy of Pubmed

| **#** | **Searches** |
| --- | --- |
| 1 | (((((((((((((((((((((((((Exercises[Title/Abstract]) OR (Exercise,Physical[Title/Abstract])) OR (Exercises, Physical[Title/Abstract])) OR (Physical Exercise[Title/Abstract])) OR (Physical Exercises[Title/Abstract])) OR (Physical Activity[Title/Abstract])) OR (Activities, Physical[Title/Abstract])) OR (Activity, Physical[Title/Abstract])) OR (Physical Activities[Title/Abstract])) OR (Exercise, Aerobic[Title/Abstract])) OR (Aerobic Exercise[Title/Abstract])) OR (Aerobic Exercises[Title/Abstract])) OR (Exercises, Aerobic[Title/Abstract])) OR (Exercise, Isometric[Title/Abstract])) OR (Exercises, Isometric[Title/Abstract])) OR (Isometric Exercises[Title/Abstract])) OR (Isometric Exercise[Title/Abstract])) OR (Acute Exercise[Title/Abstract])) OR (Acute Exercises[Title/Abstract])) OR (Exercise, Acute[Title/Abstract])) OR (Exercises, Acute[Title/Abstract])) OR (Exercise Training[Title/Abstract])) OR (Exercise Trainings[Title/Abstract])) OR (Training, Exercise[Title/Abstract])) OR (Trainings, Exercise[Title/Abstract])) OR (Exercise[MeSH Terms]) |
| 2 | (((((((((((((((((((((((Training, Resistance[Title/Abstract]) OR (Strength Training[Title/Abstract])) OR (Training, Strength[Title/Abstract])) OR (Weight-Lifting Strengthening Program[Title/Abstract])) OR (Strengthening Programs, Weight-Lifting[Title/Abstract])) OR (Strengthening Program, Weight-Lifting[Title/Abstract])) OR (Weight Lifting Strengthening Program[Title/Abstract])) OR (Weight-Lifting Strengthening Programs[Title/Abstract])) OR (Weight-Lifting Exercise Program[Title/Abstract])) OR (Exercise Programs, Weight-Lifting[Title/Abstract])) OR (Exercise Program, Weight-Lifting[Title/Abstract])) OR (Weight Lifting Exercise Program[Title/Abstract])) OR (Weight-Lifting Exercise Programs[Title/Abstract])) OR (Weight-Bearing Strengthening Program[Title/Abstract])) OR (Strengthening Programs, Weight-Bearing[Title/Abstract])) OR (Strengthening Program, Weight-Bearing[Title/Abstract])) OR (Weight Bearing Strengthening Program[Title/Abstract])) OR (Weight-Bearing Strengthening Programs[Title/Abstract])) OR (Weight-Bearing Exercise Program[Title/Abstract])) OR (Exercise Programs, Weight-Bearing[Title/Abstract])) OR (Exercise Program, Weight-Bearing[Title/Abstract])) OR (Weight Bearing Exercise Program[Title/Abstract])) OR (Weight-Bearing Exercise Programs[Title/Abstract])) OR (Resistance training[MeSH Terms]) |
| 3 | ((((((((Cardiovascular Exercise[Title/Abstract]) OR (Endurance Training[Title/Abstract])) OR (Walking[Title/Abstract])) OR (Running[Title/Abstract])) OR (Cycling[Title/Abstract])) OR (Swimming[Title/Abstract])) OR (Dancing[Title/Abstract])) OR (combined training[Title/Abstract])) OR (Concurrent Training[Title/Abstract]) |
| 4 | ((((((((((((High Intensity Interval Training[Title/Abstract]) OR (High-Intensity Interval Trainings[Title/Abstract])) OR (Interval Training, High-Intensity[Title/Abstract])) OR (Interval Trainings, High-Intensity[Title/Abstract])) OR (Training, High-Intensity Interval[Title/Abstract])) OR (Trainings, High-Intensity Interval[Title/Abstract])) OR (High-Intensity Intermittent Exercise[Title/Abstract])) OR (Exercise, High-Intensity Intermittent[Title/Abstract])) OR (Exercises, High-Intensity Intermittent[Title/Abstract])) OR (High-Intensity Intermittent Exercises[Title/Abstract])) OR (Sprint Interval Training[Title/Abstract])) OR (Sprint Interval Trainings[Title/Abstract])) OR (High-Intensity Interval Training[MeSH Terms]) |
| 5 | ((((((Circuit-Based Exercise[MeSH Terms]) OR (Circuit Based Exercise[Title/Abstract])) OR (Circuit-Based Exercises[Title/Abstract])) OR (Exercise, Circuit-Based[Title/Abstract])) OR (Exercises, Circuit-Based[Title/Abstract])) OR (Circuit Training[Title/Abstract])) OR (Training, Circuit[Title/Abstract]) |
| 6 | (((((((((((((((((Chinese exercise[Title/Abstract]) OR (traditional exercise[Title/Abstract])) OR (traditional chinese medicine[Title/Abstract])) OR (chinese traditional exercise[Title/Abstract])) OR (traditional chinese exercise[Title/Abstract])) OR (traditional exercise[Title/Abstract])) OR (traditional Kungfu[Title/Abstract])) OR (Kungfu[Title/Abstract])) OR (Shadowboxing[Title/Abstract])) OR (Five-animal exercises[Title/Abstract])) OR (Wuqinxi[Title/Abstract])) OR (Five animal frolics[Title/Abstract])) OR (Five-animal boxing[Title/Abstract])) OR (Baduanjin[Title/Abstract])) OR (Yijinjing[Title/Abstract])) OR (liuzijue[Title/Abstract])) OR (((Qigong[MeSH Terms]) OR (Ch'i Kung[Title/Abstract])) OR (Qi Gong[Title/Abstract]))) OR ((((((((((taiji[MeSH Terms]) OR (Tai-ji[Title/Abstract])) OR (Tai Chi[Title/Abstract])) OR (Chi, Tai[Title/Abstract])) OR (Tai Chi Chuan[Title/Abstract])) OR (Taijiquan[Title/Abstract])) OR (T'ai Chi[Title/Abstract])) OR (Tai Ji Quan[Title/Abstract])) OR (Ji Quan, Tai[Title/Abstract])) OR (Quan, Tai Ji[Title/Abstract])) |
| 7 | (((Mind-body exercises[Title/Abstract]) OR (yoga[Title/Abstract])) OR (dance[Title/Abstract])) OR (Pilates[Title/Abstract]) |
| 8 | (((((((((((((Prediabetic State[MeSH Terms]) OR (Prediabetic States[Title/Abstract])) OR (State, Prediabetic[Title/Abstract])) OR (States, Prediabetic[Title/Abstract])) OR (Prediabetes[Title/Abstract])) OR (Pre-diabetes[Title/Abstract])) OR (impaired glucose[Title/Abstract])) OR (impaired fasting glucose[Title/Abstract])) OR (impaired glucose intolerance[Title/Abstract])) OR (impaired glucose tolerance[Title/Abstract])) OR (impaired glucose regulation[Title/Abstract])) OR (borderline diabetes[Title/Abstract])) OR (higher risk of diabetes[Title/Abstract])) OR (high risk of diabetes[Title/Abstract]) |
| 9 | (((((randomized controlled trial[Publication Type]) OR (randomized)) OR (clinical trials as topic[MeSH Terms])) OR (placebo)) OR (randomly)) OR (trial) OR (randomised) |
| 10 | #1 OR #2 OR #3 OR #4 OR #5 OR #6 OR #7 |
| 11 | #8 AND #9 AND #10 |

**Table S2.** Search strategy of Web of Science

| **#** | **Searches** |
| --- | --- |
| 1 | TS=("Prediabetic State" OR "Prediabetic States" OR "States, Prediabetic" OR "Prediabetes" OR "impaired glucose" OR "impaired fasting glucose" OR "impaired glucose intolerance" OR "impaired glucose tolerance" OR "impaired glucose regulation" OR "borderline diabetes" OR "higher risk of diabetes" OR "high risk of diabetes") |
| 2 | TS=("Exercise" OR "Exercises" OR "Exercise, Physical" OR "Exercises, Physical" OR "Physical Exercise" OR "Physical Exercises" OR "Physical Activity" OR "Activities, Physical" OR "Activity, Physical" OR "Physical Activities" OR "Exercise, Aerobic" OR "Aerobic Exercise" OR "Aerobic Exercises" OR "Exercises, Aerobic" OR "Exercise, Isometric" OR "Exercises, Isometric" OR "Isometric Exercises" OR "Isometric Exercise" OR "Acute Exercise" OR "Acute Exercises" OR "Exercise, Acute" OR "Exercises, Acute" OR "Exercise Training" OR "Exercise Trainings" OR "Training, Exercise" OR "Trainings, Exercise") |
| 3 | TS=("Resistance training" OR "Training, Resistance" OR "Strength Training" OR "Training, Strength" OR "Weight-Lifting Strengthening Program" OR "Strengthening Programs, Weight-Lifting" OR "Strengthening Program, Weight-Lifting" OR "Weight Lifting Strengthening Program" OR "Weight-Lifting Strengthening Programs" OR "Weight-Lifting Exercise Program" OR "Exercise Programs, Weight-Lifting" OR "Exercise Program, Weight-Lifting" OR "Weight Lifting Exercise Program" OR "Weight-Lifting Exercise Programs" OR "Weight-Bearing Strengthening Program" OR "Strengthening Programs, Weight-Bearing" OR "Strengthening Program, Weight-Bearing" OR "Weight Bearing Strengthening Program" OR "Weight-Bearing Strengthening Programs" OR "Weight-Bearing Exercise Program" OR "Exercise Programs, Weight-Bearing" OR "Exercise Program, Weight-Bearing" OR "Weight Bearing Exercise Program" OR "Weight-Bearing Exercise Programs") |
| 4 | TS=("Cardiovascular Exercise" OR "Endurance Training" OR "Walking" OR "Running" OR "Cycling" OR "Swimming" OR "Dancing" OR "combined training" OR "Concurrent Training") |
| 5 | TS=("High-Intensity Interval Training" OR "High Intensity Interval Training" OR "High-Intensity Interval Trainings" OR "Interval Training, High-Intensity" OR "Interval Trainings, High-Intensity" OR "Training, High-Intensity Interval" OR "Trainings, High-Intensity Interval" OR "High-Intensity Intermittent Exercise" OR "Exercise, High-Intensity Intermittent" OR "Exercises, High-Intensity Intermittent" OR "High-Intensity Intermittent Exercises" OR "Sprint Interval Training" OR "Sprint Interval Trainings") |
| 6 | TS=("Circuit-Based Exercise" OR "Circuit Based Exercise" OR "Circuit-Based Exercises" OR "Exercise, Circuit-Based" OR "Exercises, Circuit-Based" OR "Circuit Training" OR "Training, Circuit") |
| 7 | TS=("Chinese exercise" OR "traditional exercise" OR "traditional chinese medicine" OR "chinese traditional exercise" OR "traditional chinese exercise" OR "traditional Kungfu" OR "Kungfu" OR "Shadowboxing" OR "Five-animal exercises" OR "Wuqinxi" OR "Five animal frolics" OR "Five-animal boxing" OR "Baduanjin" OR "Yijinjing" OR "liuzijue" OR "Qigong" OR "Ch'i Kung" OR "Qi Gong" OR "taiji" OR "Tai-ji" OR "Tai Chi" OR "Chi, Tai" OR "Tai Chi Chuan" OR "Taijiquan" OR "T'ai Chi" OR "Tai Ji Quan" OR "Ji Quan, Tai" OR "Quan, Tai Ji") |
| 8 | TS=("Mind-body exercises" OR "yoga" OR "dance" OR "Pilates") |
| 9 | TS=("randomized controlled trial" OR "randomized" OR "clinical trials" OR "placebo" OR "randomly" OR "trial" OR "randomised") |
| 10 | #2 OR #3 OR #4 OR #5 OR #6 OR #7 OR #8 |
| 11 | #1 AND #9 AND #10 |

**Table S3.** Search strategy of Cochrane Central Register of Controlled Trials

| **#** | **Searches** |
| --- | --- |
| 1 | MeSH descriptor: [Prediabetic State] explode all trees |
| 2 | MeSH descriptor: [High-Intensity Interval Training] explode all trees |
| 3 | MeSH descriptor: [Exercise] explode all trees |
| 4 | MeSH descriptor: [Circuit-Based Exercise] explode all trees |
| 5 | MeSH descriptor: [Resistance Training] explode all trees |
| 6 | MeSH descriptor: [Tai Ji] explode all trees |
| 7 | MeSH descriptor: [Qigong] explode all trees |
| 8 | MeSH descriptor: [Yoga] explode all trees |
| 9 | MeSH descriptor: [Exercise Movement Techniques] explode all trees |
| 10 | ("aerobic exercise":ti,ab,kw OR "aerobic training":ti,ab,kw OR "Cardiovascular Exercise":ti,ab,kw OR "Endurance Training":ti,ab,kw OR "Walking":ti,ab,kw OR "Running":ti,ab,kw OR "Cycling":ti,ab,kw OR "Swimming":ti,ab,kw OR "Dancing":ti,ab,kw OR "combined training":ti,ab,kw OR "Concurrent Training":ti,ab,kw OR "Mind-body exercises":ti,ab,kw OR "yoga":ti,ab,kw OR "dance":ti,ab,kw OR "pilates":ti,ab,kw OR "traditional chinese medicine":ti,ab,kw OR "chinese traditional exercise":ti,ab,kw OR "traditional exercise":ti,ab,kw OR "traditional Kungfu":ti,ab,kw OR "Kungfu":ti,ab,kw OR "Shadowboxing":ti,ab,kw OR "Wuqinxi":ti,ab,kw OR "Five-animal exercises":ti,ab,kw OR "Five animal frolics":ti,ab,kw OR "Five-animal boxing":ti,ab,kw OR "Baduanjin":ti,ab,kw OR "Yijinjing":ti,ab,kw OR "liuzijue":ti,ab,kw) |
| 11 | #2 OR #3 OR #4 OR #5 OR #6 OR #7 OR #8 OR #9 OR #10 |
| 12 | #1 AND #11 |

**Table S4.** Search strategy of Embase

| **#** | **Searches** |
| --- | --- |
| 1 | 'Prediabetic State'/exp OR 'Prediabetic State':ab,ti OR 'Prediabetic States':ab,ti OR 'States, Prediabetic':ab,ti OR 'Prediabetes':ab,ti OR 'impaired glucose':ab,ti OR 'impaired fasting glucose':ab,ti OR 'impaired glucose intolerance':ab,ti OR 'impaired glucose tolerance':ab,ti OR 'impaired glucose regulation':ab,ti OR 'borderline diabetes':ab,ti OR 'higher risk of diabetes':ab,ti OR 'high risk of diabetes':ab,ti |
| 2 | 'Exercise'/exp OR 'Exercise':ab,ti OR 'Exercises':ab,ti OR 'Exercise, Physical':ab,ti OR 'Exercises, Physical':ab,ti OR 'Physical Exercise':ab,ti OR 'Physical Exercises':ab,ti OR 'Physical Activity':ab,ti OR 'Activities, Physical':ab,ti OR 'Activity, Physical':ab,ti OR 'Physical Activities':ab,ti OR 'Exercise, Aerobic':ab,ti OR 'Aerobic Exercise':ab,ti OR 'Aerobic Exercises':ab,ti OR 'Exercises, Aerobic':ab,ti OR 'Exercise, Isometric':ab,ti OR 'Exercises, Isometric':ab,ti OR 'Isometric Exercises':ab,ti OR 'Isometric Exercise':ab,ti OR 'Acute Exercise':ab,ti OR 'Acute Exercises':ab,ti OR 'Exercise, Acute':ab,ti OR 'Exercises, Acute':ab,ti OR 'Exercise Training':ab,ti OR 'Exercise Trainings':ab,ti OR 'Training, Exercise':ab,ti OR 'Trainings, Exercise':ab,ti |
| 3 | 'Resistance training'/exp OR 'Resistance training':ab,ti OR 'Training, Resistance':ab,ti OR 'Strength Training':ab,ti OR 'Training, Strength':ab,ti OR 'Weight-Lifting Strengthening Program':ab,ti OR 'Strengthening Programs, Weight-Lifting':ab,ti OR 'Strengthening Program, Weight-Lifting':ab,ti OR 'Weight Lifting Strengthening Program':ab,ti OR 'Weight-Lifting Strengthening Programs':ab,ti OR 'Weight-Lifting Exercise Program':ab,ti OR 'Exercise Programs, Weight-Lifting':ab,ti OR 'Exercise Program, Weight-Lifting':ab,ti OR 'Weight Lifting Exercise Program':ab,ti OR 'Weight-Lifting Exercise Programs':ab,ti OR 'Weight-Bearing Strengthening Program':ab,ti OR 'Strengthening Programs, Weight-Bearing':ab,ti OR 'Strengthening Program, Weight-Bearing':ab,ti OR 'Weight Bearing Strengthening Program':ab,ti OR 'Weight-Bearing Strengthening Programs':ab,ti OR 'Weight-Bearing Exercise Program':ab,ti OR 'Exercise Programs, Weight-Bearing':ab,ti OR 'Exercise Program, Weight-Bearing':ab,ti OR 'Weight Bearing Exercise Program':ab,ti OR 'Weight-Bearing Exercise Programs':ab,ti |
| 4 | 'Cardiovascular Exercise':ab,ti OR 'Endurance Training':ab,ti OR 'Walking':ab,ti OR 'Running':ab,ti OR 'Cycling':ab,ti OR 'Swimming':ab,ti OR 'Dancing':ab,ti OR 'combined training':ab,ti OR 'Concurrent Training':ab,ti |
| 5 | 'High-Intensity Interval Training'/exp OR 'High Intensity Interval Training':ab,ti OR 'High-Intensity Interval Trainings':ab,ti OR 'Interval Training, High-Intensity':ab,ti OR 'Interval Trainings, High-Intensity':ab,ti OR 'Training, High-Intensity Interval':ab,ti OR 'Trainings, High-Intensity Interval':ab,ti OR 'High-Intensity Intermittent Exercise':ab,ti OR 'Exercise, High-Intensity Intermittent':ab,ti OR 'Exercises, High-Intensity Intermittent':ab,ti OR 'High-Intensity Intermittent Exercises':ab,ti OR 'Sprint Interval Training':ab,ti OR 'Sprint Interval Trainings':ab,ti |
| 6 | 'Circuit-Based Exercise'/exp OR 'Circuit Based Exercise':ab,ti OR 'Circuit-Based Exercises':ab,ti OR 'Exercise, Circuit-Based':ab,ti OR 'Exercises, Circuit-Based':ab,ti OR 'Circuit Training'/exp OR 'Training, Circuit':ab,ti |
| 7 | 'chinese exercise':ab,ti OR 'traditional exercise':ab,ti OR 'traditional chinese medicine':ab,ti OR 'chinese traditional exercise':ab,ti OR 'traditional chinese exercise':ab,ti OR 'traditional kungfu':ab,ti OR 'kungfu':ab,ti OR 'shadowboxing':ab,ti OR 'five-animal exercises':ab,ti OR 'wuqinxi':ab,ti OR 'five animal frolics':ab,ti OR 'five-animal boxing':ab,ti OR 'baduanjin':ab,ti OR 'yijinjing':ab,ti OR 'liuzijue':ab,ti OR 'qigong'/exp OR 'chi kung':ab,ti OR 'qi gong':ab,ti OR 'taiji' OR 'tai-ji':ab,ti OR 'chi, tai':ab,ti OR 'tai chi chuan':ab,ti OR 'taijiquan':ab,ti OR 'tai chi':ab,ti OR 'tai ji quan':ab,ti OR 'ji quan, tai':ab,ti OR 'quan, tai ji':ab,ti |
| 8 | 'Mind-body exercises':ab,ti OR 'yoga'/exp OR 'dance':ab,ti OR 'Pilates'/exp |
| 9 | 'randomized controlled trial'/exp OR 'randomized':ab,ti OR 'clinical trials':ab,ti OR 'placebo':ab,ti OR 'randomly':ab,ti OR 'trial':ab,ti OR 'randomised':ab,ti |
| 10 | #2 OR #3 OR #4 OR #5 OR #6 OR #7 OR #8 |
| 11 | #1 AND #9 AND #10 |

**Table S5.** Search strategy of CNKI

| **#** | **Searches** |
| --- | --- |
| 1 | TKA%=(中医运动+传统功法+中国传统运动+五禽戏+太极+太极拳+八段锦+易筋经+功夫+气功+六字诀+身心运动+瑜伽+舞蹈+普拉提+抗阻训练＋阻力训练＋抗阻运动+力量训练+有氧运动+耐力训练+心肺锻炼+跳绳+有氧训练+跑步+游泳+长跑+骑行+广场舞+健身操+慢跑+自行车+步行+健步走+联合运动+同期训练+循环训练) *(糖尿病前期+空腹血糖受损+糖耐量受损+血糖受损调节＋糖调节受损) |

**Table S6.** Search strategy of WanFang Data

| **#** | **Searches** |
| --- | --- |
| 1 | 主题:(中医运动 OR 传统功法 OR 中国传统运动 OR 五禽戏 OR 太极 OR 太极拳 OR 八段锦 OR 易筋经 OR 功夫 OR 气功 OR 六字诀 OR 身心运动 OR 瑜伽 OR 舞蹈 OR 普拉提 OR 抗阻训练 OR 阻力训练 OR 抗阻运动 OR 力量训练 OR 有氧运动 OR 耐力训练 OR 心肺锻炼 OR 跳绳 OR 有氧训练 OR 跑步 OR 游泳 OR 长跑 OR 骑行 OR 广场舞 OR 健身操 OR 慢跑 OR 自行车 OR 步行 OR 健步走 OR 联合运动 OR 同期训练 OR 循环训练) |
|  | 主题:(糖尿病前期 OR 空腹血糖受损 OR 糖耐量受损 OR 血糖受损调节 OR 糖调节受损) |
|  | #1 AND #2 |

# Appendix 2: Definitions of physical activity types and non-exercise training control

**2.1** Definitions of Exercise Interventions

**Aerobic Exercise(AT)** ^4^: Characterized by prolonged activities that increase heart rate and respiration, primarily targeting cardiovascular endurance. Common forms include walking, running, cycling, and swimming.

**Resistance Exercise(RT)** ^4^: Activities designed to enhance muscular strength, endurance, and hypertrophy by applying resistance. Typical examples include weightlifting, bodyweight exercises, and resistance band workouts.

**Combined Exercise(AT+RT)** ^4^: Refers to combining aerobic and resistance exercises within a single session or training program. Its purpose is to provide the benefits of both modalities, such as improved cardiovascular health and muscle strength.

**Traditional Chinese Exercises (TCEs)** ^5^: These exercises, rooted in Chinese culture, emphasize not only physical training but also breathing regulation, mental health improvement, and overall balance. Examples include Tai Chi, Qigong, Baduanjin, Yijinjing, and Five-Animal Frolics.

**High-Intensity Interval Training (HIIT)** ^6,7^: Defined as exercising at “near maximal” intensity, characterized by alternating between high-intensity exercise and low-intensity recovery or rest phases. The intensity is ≥75% of VO2max, VO2peak, peak power output (PPO), HRmax, HRpeak, or HRR.

**2.2** Classification of Exercise Intensity

Based on the recommendations from the American College of Sports Medicine (ACSM)^1–3^, aerobic and resistance exercises are further classified according to exercise intensity and training duration.

**2.2.1 Aerobic Exercise:**

**Low-Intensity Aerobic Training (LAT)**: Exercises performed at 30-40% HRR or VO2R, 37-45% VO₂max, 57-63% HRmax, RPE of 9-11, or 2-3 METs.

**Moderate-Intensity Aerobic Training (MAT)**: Exercises performed at 40-59% HRR or VO2R, 46-63% VO₂max, 64-75% HRmax, RPE of 12-13, or 3-6 METs.

**High-Intensity Aerobic Training (HAT)**: Exercises performed at 60-90% HRR or VO2R, 64-91% VO₂max, 76-95% HRmax, RPE of 14-17, or 6-8.8 METs. Unlike HAT, which involves continuous high-intensity aerobic exercise, HIIT consists of alternating periods of high- and low-intensity efforts.

**Note**: Unlike HAT, which involves continuous high-intensity aerobic exercise, HIIT alternates between short bursts of maximal effort and recovery periods. HAT is classified based on sustained intensity, whereas HIIT is defined by its interval-based structure and fluctuating intensity.

**2.2.2 Resistance Training**:

**Low-Intensity Resistance Training (LRT)**: Exercises performed at <50% of one-repetition maximum (1RM) or 8–12 repetitions.

**Moderate-Intensity Resistance Training (MRT)**: Exercises performed at 50–69% 1RM or 10–15 repetitions.

**High-Intensity Resistance Training (HRT)**: Exercises performed at >70% 1RM or 15–20 repetitions.

**2.2.3 Combined Exercise**

**Moderate-intensity aerobic combined with moderate -intensity resistance training(MAT+MRT):** Refers to a combined exercise regimen involving moderate-intensity aerobic training (MAT) and moderate-intensity resistance training (MRT). MAT is performed at 40-59% HRR or VO2R, 46-63% VO₂max, or 64-75% HRmax, while MRT is conducted at 50-69% of one-repetition maximum (1RM) or 10-15 repetitions.

**Moderate-intensity aerobic combined with high-intensity resistance training (MAT+HRT):** Refers to a combined exercise regimen involving moderate-intensity aerobic training (MAT) and high-intensity resistance training (HRT). MAT is performed at 40-59% HRR or VO2R, 46-63% VO₂max, or 64-75% HRmax, while HRT is conducted at >70% 1RM or 15-20 repetitions.

**References**

1. American College of Sports Medicine Position Stand. Exercise and physical activity for older adults. *Med. Sci. Sports Exerc.* **30**, 992–1008 (1998).

2. Garber, C. E. *et al.* Quantity and Quality of Exercise for Developing and Maintaining Cardiorespiratory, Musculoskeletal, and Neuromotor Fitness in Apparently Healthy Adults: Guidance for Prescribing Exercise. *Med. Sci. Sports Exerc.* **43**, 1334 (2011).

3. Riebe, D. *et al.* Updating ACSM’s Recommendations for Exercise Preparticipation Health Screening. *Med. Sci. Sports Exerc.* **47**, 2473–2479 (2015).

4. Pescatello, L. S. *ACSM’s Guidelines for Exercise Testing and Prescription*. (Lippincott Williams & Wilkins, 2014).

5. Kong, L. *et al.* Traditional Chinese Exercises on Pain and Disability in Middle-Aged and Elderly Patients With Neck Pain: A Systematic Review and Meta-Analysis of Randomized Controlled Trials. *Front. Aging Neurosci.* **14**, 912945 (2022).

6. MacInnis, M. J. & Gibala, M. J. Physiological adaptations to interval training and the role of exercise intensity. *J. Physiol.* **595**, 2915–2930 (2017).

7. Atakan, M. M. *et al.* Effects of high-intensity interval training (HIIT) and sprint interval training (SIT) on fat oxidation during exercise: a systematic review and meta-analysis. *Br. J. Sports Med.* **56**, 988–996 (2022).

# Appendix 3: Characteristics of included studies

**Table S3.1:** Baseline of characteristics of included studies

| Author  Year | Country | Group | Sample Size  (F/M) | Age  (Mean ± SD) | Exercise Prescription | | | Results |
| --- | --- | --- | --- | --- | --- | --- | --- | --- |
|  |  |  |  |  | Exercise intensity | Exercise Frequency | Exercise Period |  |
| Alvarez et al. 2012 | Chile | HIIT | 12/0 | 39.2±9.5 | 85% HRmax | 3 | 12 | c,h,i,j |
|  |  | RT | 8/0 | 33.9 ± 9.3 | 75%~85%1RM | 2 | 12 |  |
|  |  | C | 13/0 | 43.3 ± 8.1 |  |  |  |  |
| Badaam et al.2021 | India | HIIT | 0/74 | 31± 3.4 | all-out run effort | 3 | 12 | a,c,h |
|  |  | AT | 0/72 | 30.7 ± 3.3 | 70% HRmax | 5 | 12 |  |
| Burtscher et al. 2009 | Austria | AT+RT | 10/8 | 59.1 ± 7.8 | 70%HRmax+70%1RM | 2 | 48 | c,d,f,h,i |
|  |  | C | 10/8 | 55.8 ± 5.5 |  |  | 24 |  |
| Cai et al. 2023 | China | TCEs | 9/8 | 63.41 ± 5.06 |  | 5 | 24 | a, b, c, d, e, f, g, h, i |
|  |  | C | 12/5 | 61.82 ± 4.33 |  |  |  |  |
| Chen et al. 2021 | China | AT | 24/59 | 60.93 ± 5.71 | 60%-70% HRmax | 3 | 96 | a, b, c, d, e, f, h |
|  |  | RT | 30/52 | 59.91 ± 5.92 | 50%-60%1RM | 3 | 96 |  |
|  |  | C | 33/50 | 60.73 ± 5.83 |  |  |  |  |
| Cheng et al. 2017 | China | AT | 23/6 | 46.9 ± 7.3 | 60%-75% VO2max | 3 | 24 | a, b, c, e, i |
|  |  | C | 22/7 | 48.1 ± 6.8 |  |  |  |  |
| Desch et al.2010 | Germany | AT | 3/11 | 62.3 ± 6.2 | 75% HRmax | 3 | 24 | a, b, c, h |
|  |  | C | 4/8 | 62.3 ± 6.5 |  |  |  |  |
| Eriksson et al.1998 | Finland | AT | 4/3 | 60 ± 5 | 60% HRmax | 3 | 24 | c, d, f, e, h |
|  |  | C | 3/4 | 60 ± 5 |  |  |  |  |
| Fritz et al.2013 | Sweden | AT | 9/5 | 59.1± 6.2 | 60%~70%HRmax | 5 | 16 | a, b, c, d, e, f, g, h, i, j |
|  |  | C | 11/10 | 61.8±3.4 |  |  |  |  |
| Gaitán et al.2019 | USA | HIIT | 9/2 | 60.1 ± 2.3 | 90% HRpeak | 2 | 6 | a, b, c, h, i, j |
|  |  | AT | 8/3 | 65.6 ± 1.9 | 70% HRpeak | 2 | 6 |  |
| Gidlund e al.2016 | Spain | RT | 0/20 | 54 ± 6.2 | 50-85% 1RM |  |  | a, b |
|  |  | AT | 0/18 | 56 ± 5.6 | 55%-75%HRR | 12 | 3 |  |
|  |  | C | 0/17 | 54 ± 6.9 |  |  |  |  |
| Gilbertson et al.2019 | USA | HIIT | 17 | 45.7 ± 4.4 | 80% HRmax | 16 | 3 | a, c, h, i |
|  |  | AT | 12 | 50.8 ± 4.4 | 45%-55% HRR | 16 | 3 |  |
| Hansen et al.2012 | Norway | RT(H) | 7/2 | 59.1 ± 6.2 | 60%-85% 1RM | 16 | 3 | b, c |
|  |  | RT(L) | 7/2 | 61.8 ± 3.4 | 45%-65% 1RM | 16 | 3 |  |
|  |  | C | 7/2 | 56.1 ± 4.4 |  |  |  |  |

| Herzig et al.2014 | Finland | AT | 24/9 | 58.1 ± 9.9 | 60%~70%HRmax | 12 | 3 | b, c, d, e, f, g, h, i, j |
| --- | --- | --- | --- | --- | --- | --- | --- | --- |
|  |  | C | 26/9 | 59.5 ± 10.8 |  |  |  |  |
| Hu et al.2022 | China | TCEs | 11/3 | 62.68±7.33 |  | 12 | 4 | a, b, c, d, e, f, g, h, i |
|  |  | C | 11/3 | 61.58±6.62 |  |  |  |  |
| Jung et al.2015 | Canada | HIIT | 11/4 | 51±11 | 90% HRpeak | 4 | 3 | h, i, j |
|  |  | AT | 16/1 | 51±10 | 50%-75% HRR | 4 | 3 |  |
| Kargarfard et al. 2022 | Iran | AT | 0/22 | 44.73 ± 3.30 | 50%-75% HRR | 12 | 3 | a, c, h, i |
|  |  | C | 0/20 | 44.73 ± 3.30 |  |  |  |  |
| Kim et al. 2022 | Korea | AT | 15/0 | 70.47 ± 5.57 | 55%-65% HRR | 12 | 3 | a, h, i |
|  |  | RT | 12/0 | 72.25 ± 5.07 | 55%-65% 1RM | 12 | 3 |  |
|  |  | C | 9/0 | 67.78 ± 2.33 |  |  |  |  |
| Kramer et al. 2018 | USA | AT | 30/58 | 62.8 ± 12.1 | 60%~70%HRmax | 24 | 3 | a, c, d, e, f, g, h, i, j |
|  |  | C | 14/32 | 61.9 ± 11.9 |  |  |  |  |
| Liao et al.2015 | China | AT | 24/28 | 43.7 ± 5.2 | 60%~70%HRmax | 12 | 5 | a, c, d, e, f, g, h, j |
|  |  | C | 25/31 | 44.9 ± 6.0 |  |  |  |  |
| Liu et al. 2013 | China | AT | 20 | 49.8 ± 4.8 | 60%-70% HRmax | 24 | 4 | b, c, h, j |
|  |  | AT+RT | 20 | 49.8 ± 4.8 | 60%-70% HRmax+15-20 reps | 24 | 4 |  |
|  |  | C | 21 | 49.8 ± 4.8 |  |  |  |  |
| Liu et al.2021 | China | AT | 40/3 | 60.35±4.29 | 60%-70% HRmax | 24 | 3 | a, b, c |
|  |  | RT | 38/4 | 60.12±3.97 | 60%-80%1RM | 24 | 3 |  |
|  |  | C | 39/4 | 59.94±4.40 |  |  |  |  |
| Luo et al.2023 | China | AT | 11/15 | 51±9.5 | 40%-60% VO2R | 12 | 3 | b, c, h, j |
|  |  | RT | 10/13 | 52±8.2 | 60%-80% 1RM | 12 | 3 |  |
|  |  | C | 10/11 | 50±6.5 |  |  |  |  |
| Ma et al. 2022 | China | TCEs | 18/16 | 59.18 ± 3.93 |  | 12 | 2 | a, b, c, d, e, f, g, i, j |
|  |  | AT | 21/11 | 59.81 ± 4.54 | 40%-60% HRR | 12 | 2 |  |
|  |  | C | 18/14 | 59.09 ± 5.25 |  |  |  |  |
| Malin et al. 2012 | USA | AT+RT | 5/3 | 45.4 ± 8.0 | 70% HRpeak＋70% 1RM | 12 | 3 | c |
|  |  | C | 6/2 | 49.8 ± 10.9 |  |  |  |  |
| Malin et al. 2013 | USA | AT+RT | 5/3 | 45.4 ± 8.0 | 70% HRpeak＋70% 1RM | 12 | 3 | d, g, f, e |
|  |  | C | 6/2 | 49.8 ± 10.9 |  |  |  |  |
| Marcus et al. 2009 | USA | RT | 10/0 | 56.3±6.4 | 85%1RM | 12 | 3 | c |
|  |  | C | 6/0 | 53..2±6.5 |  |  |  |  |
| Martins et al.2018 | Brazil | HIIT | 8/0 | 64.3±6.7 | 85% HRmax | 12 | 3 | a, c |
|  |  | AT+RT | 8/0 | 65.0±6.3 | 70% HRmax+70% 1RM | 12 | 3 |  |

| Malin et al. 2019 | USA | HIIT | 9/3 | 59.9±2.2 | 90% HRpeak | 2 | 6 | b, c, h, i |
| --- | --- | --- | --- | --- | --- | --- | --- | --- |
|  |  | AT | 11/3 | 60.4±2.3 | 70% HRpeak | 2 | 6 |  |
| Rafiei et al. 2019 | Canada | HIIT | 8/0 | 51.8 ± 8.5 | 90% HRpeak | 2 | 5 | c, h, i, j |
|  |  | AT | 7/0 | 46 ± 12.9 | 65% HRpeak | 2 | 5 |  |
| Robinson et al. 2015 | Canada | HIIT | 17/3 | 52±10 | 90% HRpeak | 2 | 5 | c |
|  |  | AT | 14/4 | 52±10 | 65% HRpeak | 2 | 5 |  |
| Roumen et al.2008 | Netherlands | AT | 24/28 | 54.2 ± 5.8 | 70% VO2max | 144 | 5 | a, b, c, d, e, f, g, h, i, j |
|  |  | C | 24/30 | 58.4 ± 6.8 |  |  |  |  |
| Rowan et al.2017 | Canada | HIIT | 8/3 | 53.6±8.21 | 90% HRR | 12 | 3 | a, b, c, h, i, j |
|  |  | AT | 7/3 | 47.7±6.93 | 60%-70% HRR | 12 | 3 |  |
| Safarimosavietal.2021 | Iran | HIIT | 0/8 | 38.6±4.5 | 90% VO2peak | 12 | 4 | a, b, c |
|  |  | AT(AT) | 0/8 | 39.1±4.0 | 80%-90% HRmax | 12 | 4 |  |
|  |  | AT(FAT) | 0/8 | 39.8±3.9 | 60%-70% HRmax | 12 | 4 |  |
|  |  | C | 0/8 | 37.4±3.2 |  |  |  |  |
| Skoradal et al. 2018 | Faroe Islands | AT | 14/13 | 60 ± 6 | 79%-96% HRmax | 16 | 2 | d, e, f, g |
|  |  | C | 11/12 | 62 ± 6 |  |  |  |  |
| Shamizadeh et al. 2019 | Iran | AT | 77/59 | 56.9 ±7.8 | 60%~70%VO2max | 16 | 3 | c,h,i |
|  |  | C | 85/51 | 60.4 ±7.0 |  |  |  |  |
| Venojärvi et al.2013 | Finland | AT | 0/39 | 55 ± 6.2 | 55%-75% HRR | 12 | 3 | a, b, c, d, e, f |
|  |  | RT | 0/36 | 54 ± 6.1 | 85% 1RM | 12 | 3 |  |
|  |  | C | 0/40 | 54 ± 7.2 |  |  |  |  |
| Viskochil et al.2017 | USA | AT+RT | 5/4 | 46.2±2.6 | 65% VO2peak+60%-70% 1RM | 12 | 3 | c |
|  |  | C | 6/2 | 49.8 ± 3.9 |  |  |  |  |
| Yan et al.2019 | China | AT | 25/15 | 64.23 ± 5.75 | 60%-70% HRmax | 48 | 3 | a, b, c, d, e, f, g, h |
|  |  | RT | 25/10 | 62.06 ± 8.11 | 50%-60% 1RM | 48 | 3 |  |
|  |  | C | 20/15 | 60.31 ± 7.56 |  |  |  |  |
| Yuan et al.2020 | China | AT | 59/24 | 60.93±5.71 | 60%-70%HRmax | 24 | 3 | a, b, c, d, e, f, g, h, i |
|  |  | RT | 52/30 | 59.91±5.92 | 50%-60% 1RM | 24 | 3 |  |
|  |  | C | 50/30 | 60.73±5.83 |  |  |  |  |
| Zhang et al.2023 | China | TCEs | 18/7 | 61.12 ± 6.57 | | 24 | 5 | a, b, c, d, e, f, g |
|  |  | C | 17/8 | 63.00 ± 4.72 |  |  |  |  |
| Chen et al.2019 | China | HIIT | 0/40 | 50. 95 ± 6. 06 | 90%HRpeak | 2 | 5 | a, b, c, d, e, f, g |
|  |  | AT | 0/40 | 51. 80 ± 5. 78 | 65% HRpeak | 2 | 5 |  |

| Cui et al.2020 | China | AT | 21/34 | 59.84±4.52 | 60%-70% HRmax | 48 | 3 | b, c |
| --- | --- | --- | --- | --- | --- | --- | --- | --- |
|  |  | RT | 22/33 | 59.35±4.32 | 18-22 reps | 48 | 3 |  |
| Chen et al.2011 | China | AT | 6/7 | 54.67±5.26 | 60% VO2max | 16 | 7 | b, c, d, e, f, g, h, i |
|  |  | C | 6/7 | 55.73±7.78 |  |  |  |  |
| Dai et al.2015 | China | AT+RT | 42 | 45-65 | 60%-70% HRmax+60%-80% 1RM | 12 | 3 | d, e, f, g |
|  |  | RT | 41 | 45-65 | 60%-80% 1RM | 12 | 3 |  |
|  |  | AT | 42 | 45-65 | 60%-70% HRmax | 12 | 3 |  |
|  |  | C | 43 | 45-65 |  |  |  |  |
| Fu et al.2023 | China | TCEs | 8/4 | 52.1±10.2 |  | 2 | 7 | b,c |
|  |  | C | 5/5 | 58.4±3.9 |  |  |  |  |
| Ji et al.2017 | China | RT | 20/14 | 61.17 ± 10.93 | 50%-65% 1RM | 24 | 3 | a, b, c, d, e, f, g, h, j |
|  |  | C | 21/18 | 56.90±8.58 |  |  |  |  |
| Li et al.2018a | China | AT+RT | 33/27 | 57.42±5.55 | 50%-80% HRmax+60%~80 1RM | 12 | 3 | a, b, c, h |
|  |  | AT | 32/23 | 58.89±5.22 | 50%-80% HRmax | 12 | 3 |  |
| Li et al.2018b | China | HIIT | 24/22 | 50.6±4.2 | 80% ~90% HRmax | 12 | 3 | a, b, c, d, e, f, g, h |
|  |  | AT | 23/23 | 51.1± 3.9 | 40%~60%HRmax | 12 | 3 |  |
| Li et al.2017 | China | TCEs | 7/3 | 60.50±11.37 |  | 24 | 5 | a, b, c |
|  |  | AT | 8/2 | 59.10±9.69 | 60% HRmax | 24 | 5 |  |
|  |  | C | 5/5 | 57.10±12.59 |  |  |  |  |
| Li et al.2019a | China | TCEs | 31/12 | 55.72±3.42 | | 16 | 5 | c |
|  |  | C | 29/15 | 55.25±3.38 |  |  |  |  |
| Li et al.2019b | China | TCEs | 34/15 | 56.90 ± 3.94 |  | 36 | 3 | c |
|  |  | RT | 27/18 | 56.20 ± 4.67 | 60%-70% 1RM | 36 | 3 |  |
|  |  | C | 33/14 | 56.34 ± 4.03 |  |  |  |  |
| Lu et al.2014 | China | AT+RT | 26/16 | 57.43±5.56 | 50%-80% HRmax+60%-80% 1RM | 24 | 3 | a, b, c, d, e, f, g |
|  |  | RT | 24/17 | 59.24±4.03 | 60%-80%1RM | 24 | 3 |  |
|  |  | AT | 28/14 | 58.90±5.23 | 50%-80%HRmax | 24 | 3 |  |
|  |  | C | 24/19 | 58.60±5.27 | |  |  |  |
| Luo et al.2014 | China | AT | 11/15 | 51 ± 9.48 | 40%-59% VO2R | 12 | 3 | b, c |
|  |  | RT | 10/13 | 52 ± 8.19 | 60%-80% 1RM | 12 | 3 |  |
|  |  | C | 10/13 | 50 ± 6.55 |  |  |  |  |
| Luo et al.2017 | China | AT | 27/12 | 59.95±4.46 | 50%-80%HRmax | 24 | 3 | a, b, c |
|  |  | RT | 29/9 | 58.82±3.85 | 60%-80%1RM | 24 | 3 |  |
|  |  | C | 23/15 | 59.53±5.11 | |  |  |  |

| Ma et al.2017a | China | AT | 17/5 | 57.955±3.684 | 40%-59%VO2R | 48 | 3 | a |
| --- | --- | --- | --- | --- | --- | --- | --- | --- |
|  |  | RT | 19/4 | 60.348±4.376 | 60%-70%1RM | 48 | 3 |  |
|  |  | C | 14/8 | 59.727±4.474 | |  |  |  |
| Ma et al.2017b | China | AT | 17/6 | 59.61±4.27 | 40%-59% VO2R | 48 | 3 | d, e, f, g, h |
|  |  | RT | 15/14 | 58.55±3.23 | 60%-70% 1RM | 48 | 3 |  |
|  |  | C | 13/9 | 59.82±5.26 | |  |  |  |
| Ma et al.2017c | China | AT | 27/12 | 57.4±5.4 | 40%-59% VO2R | 48 | 3 | c |
|  |  | RT | 30/8 | 59.5±4.5 | 60%-70% 1RM | 48 | 3 |  |
|  |  | C | 28/10 | 59.3±4.1 |  |  |  |  |
| Ruan et al.2024 | China | AT | 17/23 | 56. 37±5. 55 | 40%-59%VO2R | 24 | 3 | a, b, c, d, e, f, g |
|  |  | RT | 21/18 | 57. 25±5. 47 | 60%-70% 1RM | 24 | 3 |  |
| Shang et al.2024 | China | TCEs | 22/21 | 54 |  | 24 | 5 | b, c, d, e, f, g |
|  |  | AT | 21/22 | 55 | 50%-60% HRmax | 24 | 5 |  |
| Song et al.2013 | China | TCEs | 21/19 | 54 |  | 12 | 7 | a, b, c |
|  |  | C | 21/19 | 54 |  |  |  |  |
| Wei et al.2017 | China | RT | 17/17 | 53.91±4.19 | 70%-80 1RM | 48 | 3 | a, b, c, d, e, f, g |
|  |  | C | 18/17 | 51.94±4.42 | |  |  |  |
| Wei et al.2024 | China | RT | 11/9 | 52.96±4.63 | 70%-801RM | 48 | 3 | a, b, d, e, f, g |
|  |  | C | 10./10 | 52.98±4.63 | |  |  |  |
| Wang et al.2021 | China | AT | 59/24 | 60.93±5.71 | 60-70% HRmax | 48 | 4 | a, b, c, e, f, g, h |
|  |  | RT | 52/30 | 59.91±5.92 | 60% 1RM | 48 | 3 |  |
|  |  | C | 50/33 | 60.73±5.83 | |  |  |  |
| Wu et al.2017 | China | TCEs | 11/8 | 54.50±12.15 | | 12 | 5 | a, b, c |
|  |  | AT | 11/9 | 55.86±9.26 | 60%-70% HRmax | 12 | 5 |  |
|  |  | C | 12/9 | 56.25±12.42 | |  |  |  |
| Wu et al.2016 | China | TCEs | 13/7 | 60. 38 ± 4. 81 | | 12 | 5 | a, b, c, h |
|  |  | AT | 13/7 | 63. 12 ± 7. 20 | 60%-70% HRmax | 12 | 5 |  |
|  |  | C | 12/8 | 60. 38 ± 5. 81 | |  |  |  |
| Wang et al.2015 | China | AT | 10/10 | 58.73±6.99 | 50% HRmax | 12 | 3 | a, b, c, d, e, f, g, h, j |
|  |  | RT | 11/9 | 61.53±7.25 | 70%-80 1RM | 12 | 3 |  |
|  |  | C | 13/7 | 63.05±6.73 | |  |  |  |
| Wang et al.2023 | China | AT+RT | 18/26 | 50.52 ± 4.04 | 50%-60% HRmax+18-22 reps | 12 | 3 | a, b, c, d, e, f, g, h |
|  |  | AT | 21/23 | 51.33 ± 4.46 | 50%-60%HRmax | 12 | 3 |  |

| Ye et al.2019 | China | AT(Fatmax) | 30 | 62.34±6.87 | 60%-70% HRmax | 24 | 3 | b, c, h |
| --- | --- | --- | --- | --- | --- | --- | --- | --- |
|  |  | AT(AT) | 30 | 62.34±6.87 | 80%-90% HRmax | 24 | 3 |  |
|  |  | C | 30 | 62.34±6.87 | 60%-70% HRmax |  |  |  |
| Zhang et al.2018 | China | RT(H) | 20 | 60.3±5.5 | 70% 1RM | 36 | 4 | b, c, h |
|  |  | RT(M) | 20 | 61.3±4.4 | 50% 1RM | 36 | 4 |  |
|  |  | C | 20 | 61.5±4.2 |  |  |  |  |
| Zhang et al.2019 | China | HIIT | 36/64 | 57.42±9.16 | 80%-90%HRmax | 12 | 4 | a, b, c, d, e, f, g |
|  |  | C | 41/59 | 56.16±8.25 | |  |  |  |
| Zheng et al.2017 | China | RT | 18/22 | 70.28±6.64 | 50%-70% 1RM | 8 | 7 | a, b, c, d, e, f, g, h, j |
|  |  | C | 21/19 | 70.56±7.66 | |  |  |  |
| Li et al.2018c | China | AT | 4/7 | 54.41 ± 10.34 | 40%-60% VO2R | 12 | 3 | a, b, c, d, e, f, g, h, i, j |
|  |  | C | 4/8 | 54.41 ± 10.34 | |  |  |  |

AT, Aerobic exercise;MAT, Moderate intensity aerobic exercise; HAT,High-intensity aerobic exercise;RT, Resistance training; AT+RT, Combined aerobic exercise with resistance training;HIIT, High-intensity interval training; TCEs, Traditional Chinese exercises; C, Control; VO2R,Oxygen Uptake Reserve; HRpeak, Peak Heart Rate; VO2max, Maximal Oxygen Consumption; HRmax, Maximal Heart Rate; HRR, Heart rate reserve; 1RM, one-repetition maximum; Reps, Repetitions; a, HbA1c; b, 2hPG; c, FBG; d, TC; e, TG; f, HDL; g, LDL; h, BMI; i, BW; j, WC.

# Appendix 4: Risk of bias of randomized clinical trials

**Figure S4:** Overall risk of bias presented as percentage of each risk of bias item across all included studies. Green = Low risk, Red = High risk, Yellow = Some concerns.

**Table S4:** Study level risk of bias assessment using Cochrane risk of bias tool 2.0 for assessing risk of bias of randomized clinical trials.

| Unique ID | Study ID | Randomization  process | Deviations from  intended interventions | Missing outcome  data | Measurement of  the outcome | Selection of  the reported result | Overall Bias |
| --- | --- | --- | --- | --- | --- | --- | --- |
| Álvarez2012 | 1 | Some concerns | Low | Low | Low | Some concerns | Some concerns |
| Badaam2021 | 2 | Low | Low | Low | Low | Low | Low |
| Burtscher2009 | 3 | Some concerns | Low | Low | Low | Some concerns | Some concerns |
| Roumen2008 | 4 | Some concerns | Low | Low | Low | Some concerns | Some concerns |
| Cai2023 | 5 | Low | Low | Low | Low | Some concerns | High |
| Chen2021 | 6 | Low | Low | Low | Low | Some concerns | Some concerns |
| Cheng2017 | 7 | Low | Low | Low | Low | Low | Low |
| Desch2010 | 8 | Some concerns | Low | Low | Low | Some concerns | Some concerns |
| Eriksson2007 | 9 | Some concerns | Low | Low | Low | Some concerns | Some concerns |
| Fritz2013 | 10 | Low | Low | Low | Low | Some concerns | Some concerns |
| Gaitán2019 | 11 | Some concerns | Low | Low | Low | Some concerns | Some concerns |
| Gidlund2016 | 12 | Some concerns | Low | Low | Low | Some concerns | Some concerns |
| Gilbertson2019 | 13 | Some concerns | Low | High | Low | Some concerns | High |
| Hansen2012 | 14 | Some concerns | Low | Low | Low | Some concerns | Some concerns |
| Heiston2019 | 15 | Some concerns | Low | Low | Low | Some concerns | Some concerns |
| Herzig2014 | 16 | Low | Low | Low | Low | Some concerns | Some concerns |
| Hu2022 | 17 | Some concerns | Low | Low | Low | Some concerns | Some concerns |
| Jung2015 | 18 | Some concerns | Low | High | Low | Some concerns | High |
| Kargarfard2022 | 19 | Some concerns | Low | High | Low | Some concerns | High |
| Kimi2022 | 20 | Some concerns | Low | High | Low | Some concerns | High |
| Kramer2018 | 21 | Low | Low | Low | Low | Some concerns | Some concerns |
| Liao2015 | 22 | Some concerns | Low | Low | Low | Some concerns | Some concerns |
| Liu2021 | 23 | Low | Low | Low | Low | Low | Low |
| Liu2013 | 24 | Some concerns | Low | High | Low | Some concerns | High |
| Luo202 | 25 | Low | Low | Low | Low | Low | Low |
| Ma2022 | 26 | Low | Low | High | Low | Some concerns | High |
| Malin2012 | 27 | Low | Low | Low | Low | Some concerns | Some concerns |
| Malin2019 | 28 | Low | Low | Low | Low | Low | Some concerns |
| Malin2013 | 29 | Low | Low | Low | Low | Some concerns | Some concerns |
| Marcus2009 | 30 | Some concerns | Low | Low | Low | Some concerns | Some concerns |
| Martins2018 | 31 | Low | Low | High | Low | Low | Low |
| Rafiei2019 | 32 | Low | Low | Low | Low | Some concerns | Some concerns |
| Robinson2015 | 33 | Low | Low | Low | Low | Some concerns | Some concerns |
| Rowan2017 | 34 | Low | Low | Low | Low | Some concerns | Some concerns |
| Safarimosavi2021 | 35 | Low | Low | Low | Low | Some concerns | Some concerns |
| Shamizadeh2019 | 36 | Low | Low | Low | Low | Some concerns | Some concerns |
| Skoradal2018 | 37 | Low | Low | Low | Low | Some concerns | Some concerns |
| Venojärvi2013 | 38 | Some concerns | Low | Low | Low | Some concerns | Some concerns |
| Viskochil2017 | 39 | Some concerns | Low | Low | Low | Some concerns | Some concerns |
| Yan2019 | 40 | Low | Low | Low | Low | Low | Low |
| Yuan2020 | 41 | Low | Low | Low | Low | Low | Low |
| Zhang2023 | 42 | Some concerns | Low | High | Low | Some concerns | High |
| Chen2019 | 43 | Some concerns | Low | Low | Low | Some concerns | Some concerns |
| Chen2011 | 44 | Some concerns | Low | Low | Low | Some concerns | Some concerns |
| Dai2015 | 45 | Some concerns | Low | Low | Low | Some concerns | Some concerns |
| Fu2023 | 46 | Some concerns | Low | Low | Low | Some concerns | Some concerns |
| Ji2019 | 47 | Low | Low | Low | Low | Some concerns | Some concerns |
| Li2018a | 48 | Some concerns | Low | Low | Low | Some concerns | Some concerns |
| Li2019c | 49 | Some concerns | Low | Low | Low | Some concerns | Some concerns |
| Li2017 | 50 | Some concerns | Low | Low | Low | Some concerns | Some concerns |
| Li2019a | 51 | Some concerns | Low | Low | Low | Some concerns | Some concerns |
| Li2019b | 52 | Some concerns | Low | Low | Low | Some concerns | Some concerns |
| Lu2014 | 53 | Some concerns | Low | Low | Low | Some concerns | Some concerns |
| Luo2014 | 54 | Low | Low | Low | Low | Some concerns | Some concerns |
| Luo2017 | 55 | Low | Low | Low | Low | Some concerns | Some concerns |
| Ma2017a | 56 | Some concerns | Low | High | Low | Some concerns | High |
| Ma2017b | 57 | Some concerns | Low | High | Low | Some concerns | High |
| Ruan2024 | 58 | Some concerns | Low | Low | Low | Some concerns | Some concerns |
| Shang2017 | 59 | Some concerns | Low | Low | Low | Some concerns | Some concerns |
| Song2013 | 60 | Some concerns | Low | Low | Low | Some concerns | Some concerns |
| Wei2017 | 61 | Some concerns | Low | Low | Low | Some concerns | Some concerns |
| Wu2017 | 62 | Some concerns | Low | Low | Low | Some concerns | Some concerns |
| Wu2016 | 63 | Some concerns | Low | High | Low | Some concerns | High |
| Ye2019 | 64 | Some concerns | Low | High | Low | Some concerns | High |
| Zhang2018 | 65 | Some concerns | Low | High | Low | Some concerns | High |
| Zhang2019 | 66 | Some concerns | Low | Low | Low | Some concerns | Some concerns |
| Zheng2017 | 67 | Some concerns | Low | Low | Low | Some concerns | Some concerns |
| Li2018b | 68 | Low | Low | Low | Low | Some concerns | Some concerns |
| Cui2020 | 69 | Some concerns | Low | Low | Low | Some concerns | Some concerns |
| Ma2017c | 70 | Some concerns | Low | High | Low | Some concerns | High |
| Wang2015 | 71 | Some concerns | Low | Low | Low | Some concerns | Some concerns |
| Wang2021 | 72 | Some concerns | Low | Low | Low | Some concerns | Some concerns |
| Wang2023 | 73 | Some concerns | Low | Low | Low | Some concerns | Some concerns |
| Wei2024 | 74 | Some concerns | Low | Low | Low | Some concerns | Some concerns |

# Appendix 5: Evaluation of inconsistency and heterogeneity

**Table S5.1:** Global consistency

| **Clinical outcome** | **Chi square** | **P value** | **τ ^2^** |
| --- | --- | --- | --- |
| FBG | 21.13 | 0.0623 | 0.272 |
| 2hPG | 15.25 | 0.3615 | 0.1 |
| HbA1c | 20.47 | 0.0586 | 0.115 |
| TC | 9.82 | 0.5471 | 0 |
| TG | 16.78 | 0.2097 | 0.013 |
| HDL | 16.37 | 0.1280 | 0.1 |
| LDL | 13.82 | 0.2430 | 0.211 |
| BW | 1.8 | 0.8766 | 0.037 |
| BMI | 6.29 | 0.7103 | 0.017 |
| WC | 6.18 | 0.2891 | 0.077 |

**Table S5.2:** Side-splitting of HbA1c. Inconsistency test between direct and indirect treatment comparisons in mixed treatment comparison.

| Comparison | Direct | | Indirect | | Difference | | |
| --- | --- | --- | --- | --- | --- | --- | --- |
|  | Coef. | Std.Err. | Coef. | Std.Err. | Coef. | Std.Err. | P>\|z\| |
| AT v.s. RT | 0.0730631 | 0.0946583 | -0.2084333 | 0.1681246 | 0.2814963 | 0.1927359 | 0.144 |
| AT v.s. AT+RT | -0.2686363 | 0.2008147 | -0.1681935 | 0.3547 | -0.1004427 | 0.4073431 | 0.805 |
| AT v.s. HIIT | -0.3008493 | 0.1626484 | -1.022185 | 0.2996337 | 0.7213354 | 0.3400371 | 0.134 |
| AT v.s. TCEs | -0.0218181 | 0.1262244 | 0.6100992 | 0.1906117 | -0.6319173 | 0.2284465 | 0.006 |
| AT v.s. C | 0.7064722 | 0.06928 | 0.7441352 | 0.2164319 | -0.037663 | 0.2276009 | 0.869 |
| RT v.s. AT+RT | -0.0238118 | 0.2848337 | -0.4077108 | 0.238794 | 0.383899 | 0.3718457 | 0.302 |
| RT v.s. C | 0.745233 | 0.0858448 | 0.424327 | 0.2275916 | 0.3209059 | 0.2441369 | 0.189 |
| AT+RT v.s. HIIT | -0.2365759 | 0.6072857 | -0.2142163 | 0.2358047 | -0.0223595 | 0.6514597 | 0.973 |
| AT+RT v.s. C | 1.047715 | 0.2894592 | 0.8951092 | 0.2301439 | 0.1526056 | 0.3705966 | 0.68 |
| HIIT v.s. C | 1.691491 | 0.2829836 | 0.9685215 | 0.1776513 | 0.722969 | 0.3341864 | 0.061 |
| TCEs v.s. C | 0.444007 | 0.1121844 | 1.08184 | 0.2679135 | -0.6378325 | 0.2908065 | 0.028 |

**Table S5.3:** Side-splitting of FBG. Inconsistency test between direct and indirect treatment comparisons in mixed treatment comparison.

| Comparison | Direct | | Indirect | | Difference | | |
| --- | --- | --- | --- | --- | --- | --- | --- |
|  | Coef. | Std.Err. | Coef. | Std.Err. | Coef. | Std.Err. | P>\|z\| |
| AT v.s. RT | -0.17194 | 0.127254 | -0.24438 | 0.175439 | 0.072447 | 0.216636 | 0.738 |
| AT v.s. AT+RT | -0.30852 | 0.253336 | 0.397195 | 0.29807 | -0.70571 | 0.391031 | 0.071 |
| AT v.s. HIIT | -0.06496 | 0.175462 | -1.04872 | 0.341999 | 0.98376 | 0.382677 | 0.01 |
| AT v.s. TCEs | -0.04409 | 0.151842 | -0.45702 | 0.180422 | 0.412932 | 0.235786 | 0.08 |
| AT v.s. C | 0.60872 | 0.083436 | 1.101372 | 0.207395 | -0.49265 | 0.223287 | 0.072 |
| RT v.s. AT+RT | 0.015615 | 0.401988 | 0.248006 | 0.242472 | -0.23239 | 0.46954 | 0.621 |
| RT v.s. HIIT | 0.066416 | 0.693879 | -0.08166 | 0.191442 | 0.148072 | 0.720742 | 0.837 |
| RT v.s. TCEs | 0.220774 | 0.324626 | -0.06781 | 0.148867 | 0.288585 | 0.357178 | 0.419 |
| RT v.s. C | 0.936212 | 0.103326 | 0.532477 | 0.243973 | 0.403735 | 0.265347 | 0.128 |
| AT+RT v.s. HIIT | -0.2937 | 0.726678 | -0.25234 | 0.257473 | -0.04136 | 0.770943 | 0.957 |
| AT+RT v.s. C | 0.559495 | 0.256541 | 0.867994 | 0.303038 | -0.3085 | 0.397575 | 0.438 |
| HIIT v.s. C | 2.084295 | 0.352937 | 0.597929 | 0.19208 | 1.486366 | 0.405147 | 0.02 |
| TCEs v.s. C | 0.983099 | 0.118437 | 0.425048 | 0.271206 | 0.558051 | 0.297056 | 0.06 |

**Table S5.4:** Side-splitting of 2hPG. Inconsistency test between direct and indirect treatment comparisons in mixed treatment comparison.

| Comparison | Direct | | Indirect | | Difference | | |
| --- | --- | --- | --- | --- | --- | --- | --- |
|  | Coef. | Std.Err. | Coef. | Std.Err. | Coef. | Std.Err. | P>\|z\| |
| AT v.s. RT | 0.100971 | 0.094794 | 0.1326698 | 0.1499595 | -0.0316987 | 0.1774137 | 0.858 |
| AT v.s. AT+RT | -0.1983163 | 0.1754081 | -0.1964198 | 0.3705824 | -0.0018965 | 0.4095192 | 0.996 |
| AT v.s. HIIT | -0.1934737 | 0.1648778 | -1.120257 | 0.316537 | 0.9267835 | 0.355672 | 0.009 |
| AT v.s. TCEs | -0.1379089 | 0.1190201 | 0.0106338 | 0.1839853 | -0.1485427 | 0.2189055 | 0.497 |
| AT v.s. C | 0.6657787 | 0.0674899 | 0.8250388 | 0.1772339 | -0.15926 | 0.1900361 | 0.402 |
| RT v.s. AT+RT | -0.0658626 | 0.2707195 | -0.4563607 | 0.2121118 | 0.3904981 | 0.34404 | 0.256 |
| RT v.s. C | 0.5420547 | 0.0807139 | 0.7858061 | 0.2009309 | -0.2437515 | 0.2166521 | 0.261 |
| AT+RT v.s. C | 0.97027 | 0.2352743 | 0.8051145 | 0.2257301 | 0.1651555 | 0.3267033 | 0.613 |
| HIIT v.s. C | 1.671792 | 0.2679908 | 0.7842606 | 0.1858554 | 0.8875311 | 0.3265559 | 0.072 |
| TCEs v.s. C | 0.7774239 | 0.1088264 | 0.7940433 | 0.2292568 | -0.0166193 | 0.2548189 | 0.948 |

**Table S5.5:** Side-splitting of TC. Inconsistency test between direct and indirect treatment comparisons in mixed treatment comparison.

| Comparison | Direct | | Indirect | | Difference | | |
| --- | --- | --- | --- | --- | --- | --- | --- |
|  | Coef. | Std.Err. | Coef. | Std.Err. | Coef. | Std.Err. | P>\|z\| |
| AT v.s. RT | 0.131226 | 0.056679 | -0.0574345 | 0.1113403 | 0.1886605 | 0.1249581 | 0.131 |
| AT v.s. AT+RT | -0.2061073 | 0.0975194 | -0.2754027 | 0.1575752 | 0.0692954 | 0.1850643 | 0.708 |
| AT v.s. HIIT | -0.2364095 | 0.1534633 | -0.5256506 | 0.1544895 | 0.289241 | 0.2177567 | 0.184 |
| AT v.s. TCEs | 0.0711918 | 0.1355411 | 0.2347974 | 0.170941 | -0.1636056 | 0.2189737 | 0.455 |
| AT v.s. C | 0.3490557 | 0.0461913 | 0.397342 | 0.1189584 | -0.0482863 | 0.1276386 | 0.705 |
| RT v.s. AT+RT | -0.2427409 | 0.1099785 | -0.4359797 | 0.1382929 | 0.1932387 | 0.1768158 | 0.274 |
| RT v.s. C | 0.2734253 | 0.0502532 | 0.1737582 | 0.147852 | 0.0996671 | 0.1563418 | 0.524 |
| AT+RT v.s. C | 0.6080985 | 0.1026348 | 0.5274882 | 0.1428253 | 0.0806103 | 0.1758854 | 0.647 |
| HIIT v.s. C | 0.8692553 | 0.1481047 | 0.5800143 | 0.159634 | 0.289241 | 0.2177567 | 0.184 |
| TCEs v.s. C | 0.1778787 | 0.1284525 | 0.3104882 | 0.1860011 | -0.1326095 | 0.2270311 | 0.559 |

**Table S5.6:** Side-splitting of TG. Inconsistency test between direct and indirect treatment comparisons in mixed treatment comparison.

| Comparison | Direct | | Indirect | | Difference | | |
| --- | --- | --- | --- | --- | --- | --- | --- |
|  | Coef. | Std.Err. | Coef. | Std.Err. | Coef. | Std.Err. | P>\|z\| |
| AT v.s. RT | 0.1062798 | 0.1056931 | -0.0152409 | 0.1889557 | 0.1215207 | 0.2166663 | 0.575 |
| AT v.s. AT+RT | -0.4684866 | 0.1802597 | -0.3318962 | 0.3050654 | -0.1365904 | 0.3538562 | 0.699 |
| AT v.s. HIIT | -0.1122877 | 0.2790292 | -0.6867896 | 0.3710498 | 0.5745019 | 0.4642531 | 0.216 |
| AT v.s. TCEs | 0.0976325 | 0.2077213 | 0.1172918 | 0.2541146 | -0.0196592 | 0.3287242 | 0.952 |
| AT v.s. C | 0.3854284 | 0.0819494 | 0.4374491 | 0.2207405 | -0.0520207 | 0.2353804 | 0.825 |
| RT v.s. AT+RT | -0.2672069 | 0.1949517 | -0.9214916 | 0.2561562 | 0.6542847 | 0.321768 | 0.042 |
| RT v.s. C | 0.3186478 | 0.0905171 | 0.2778668 | 0.2629436 | 0.040781 | 0.2782018 | 0.883 |
| AT+RT v.s. C | 0.9134347 | 0.1951354 | 0.6655341 | 0.260583 | 0.2479006 | 0.3252657 | 0.446 |
| HIIT v.s. C | 1.062007 | 0.3630852 | 0.487505 | 0.2893103 | 0.5745019 | 0.4642535 | 0.216 |
| TCEs v.s. C | 0.3414693 | 0.1819068 | 0.1231719 | 0.3137346 | 0.2182974 | 0.3639308 | 0.549 |

**Table S5.7:** Side-splitting of HDL. Inconsistency test between direct and indirect treatment comparisons in mixed treatment comparison.

| Comparison | Direct | | Indirect | | Difference | | |
| --- | --- | --- | --- | --- | --- | --- | --- |
|  | Coef. | Std.Err. | Coef. | Std.Err. | Coef. | Std.Err. | P>\|z\| |
| AT v.s. RT | 0.0252461 | 0.1001061 | -0.1450155 | 0.1845056 | 0.1702617 | 0.2099181 | 0.417 |
| AT v.s. AT+RT | 0.1344458 | 0.1728732 | -0.0030496 | 0.25714 | 0.1374954 | 0.3098087 | 0.657 |
| AT v.s. HIIT | 0.2344187 | 0.264573 | 0.9370485 | 0.3487249 | -0.7026299 | 0.4377121 | 0.108 |
| AT v.s. TCEs | 0.0771616 | 0.1995807 | 0.1810092 | 0.246743 | -0.1038477 | 0.3173072 | 0.743 |
| AT v.s. C | -0.2376051 | 0.0807992 | -0.2322063 | 0.2074869 | -0.0053988 | 0.2230981 | 0.981 |
| RT v.s. AT+RT | 0.0374671 | 0.1942925 | 0.2010983 | 0.2315063 | -0.1636312 | 0.302447 | 0.588 |
| RT v.s. C | -0.1834024 | 0.0854427 | -0.5439856 | 0.2470883 | 0.3605832 | 0.2615427 | 0.168 |
| AT+RT v.s. C | -0.2500962 | 0.172862 | -0.4924646 | 0.2510188 | 0.2423685 | 0.3042671 | 0.426 |
| HIIT v.s. C | -1.151878 | 0.3408356 | -0.4492483 | 0.2746322 | -0.7026301 | 0.437712 | 0.108 |
| TCEs v.s. C | -0.4883631 | 0.1739166 | 0.0271394 | 0.2953822 | -0.5155025 | 0.3442891 | 0.134 |

**Table S5.8:** Side-splitting of LDL. Inconsistency test between direct and indirect treatment comparisons in mixed treatment comparison.

| Comparison | Direct | | Indirect | | Difference | | |
| --- | --- | --- | --- | --- | --- | --- | --- |
|  | Coef. | Std.Err. | Coef. | Std.Err. | Coef. | Std.Err. | P>\|z\| |
| AT v.s. RT | 0.2584276 | 0.1319789 | -0.2190673 | 0.2368442 | 0.4774949 | 0.271459 | 0.079 |
| AT v.s. AT+RT | -0.1790155 | 0.2285875 | 0.0835406 | 0.3741743 | -0.2625561 | 0.4384495 | 0.549 |
| AT v.s. HIIT | -0.1555494 | 0.3592881 | -0.7107725 | 0.4948094 | 0.5552231 | 0.6114902 | 0.364 |
| AT v.s. TCEs | -0.015677 | 0.2579161 | 0.4867574 | 0.3076663 | -0.5024344 | 0.4017926 | 0.211 |
| AT v.s. C | 0.4887125 | 0.1062005 | 0.5467907 | 0.2773198 | -0.0580782 | 0.29669 | 0.845 |
| RT v.s. AT+RT | 0.0772717 | 0.2457196 | -0.7887649 | 0.3147672 | 0.8660366 | 0.3996517 | 0.053 |
| RT v.s. C | 0.3913606 | 0.1129696 | -0.0293887 | 0.3350147 | 0.4207493 | 0.3536018 | 0.234 |
| AT+RT v.s. C | 0.6848955 | 0.2428791 | 0.4506899 | 0.3330342 | 0.2342056 | 0.4122559 | 0.57 |
| HIIT v.s. C | 1.191598 | 0.4846823 | 0.6363752 | 0.3728316 | 0.5552231 | 0.6114902 | 0.364 |
| TCEs v.s. C | 0.208033 | 0.2232538 | 0.6042298 | 0.3979625 | -0.3961968 | 0.4570391 | 0.386 |

**Table S5.9:** Side-splitting of BMI. Inconsistency test between direct and indirect treatment comparisons in mixed treatment comparison.

| Comparison | Direct | | Indirect | | Difference | | |
| --- | --- | --- | --- | --- | --- | --- | --- |
|  | Coef. | Std.Err. | Coef. | Std.Err. | Coef. | Std.Err. | P>\|z\| |
| AT v.s. RT | 0.0189414 | 0.0786008 | -0.0150739 | 0.1383924 | 0.0340153 | 0.1591713 | 0.831 |
| AT v.s. AT+RT | -0.0640721 | 0.1527764 | -0.0503655 | 0.3158435 | -0.0137066 | 0.351039 | 0.969 |
| AT v.s. HIIT | 0.0020871 | 0.1099515 | 0.1164003 | 0.383449 | -0.1143131 | 0.3989181 | 0.774 |
| AT v.s. TCEs | -0.0440048 | 0.171028 | 0.2551405 | 0.2156393 | -0.2991453 | 0.2752344 | 0.277 |
| AT v.s. C | 0.3189058 | 0.0531114 | 0.229633 | 0.2032037 | 0.0892728 | 0.2102153 | 0.671 |
| RT v.s. HIIT | -0.0168107 | 0.4724824 | 0.0012184 | 0.1289349 | -0.0180292 | 0.4912912 | 0.971 |
| RT v.s. C | 0.3142053 | 0.0702651 | 0.2151436 | 0.1956427 | 0.0990617 | 0.2090449 | 0.636 |
| AT+RT v.s. C | 0.3195193 | 0.2484852 | 0.4013797 | 0.1733263 | -0.0818604 | 0.3029211 | 0.787 |
| HIIT v.s. C | 0.0972182 | 0.4219784 | 0.3189728 | 0.1204833 | -0.2217545 | 0.4385961 | 0.613 |
| TCEs v.s. C | 0.1300559 | 0.144283 | 0.7650292 | 0.312846 | -0.6349732 | 0.3444652 | 0.065 |

**Table S5.10:** Side-splitting of BW. Inconsistency test between direct and indirect treatment comparisons in mixed treatment comparison.

| Comparison | Direct | | Indirect | | Difference | | |
| --- | --- | --- | --- | --- | --- | --- | --- |
|  | Coef. | Std.Err. | Coef. | Std.Err. | Coef. | Std.Err. | P>\|z\| |
| AT v.s. RT | 0.0791538 | 0.1701343 | 0.4054455 | 0.2805372 | -0.3262917 | 0.3271602 | 0.319 |
| AT v.s. HIIT | -0.0302711 | 0.1569708 | 0.2025812 | 0.4069456 | -0.2328523 | 0.4361782 | 0.593 |
| AT v.s. TCEs | 0.0240712 | 0.1800901 | -0.0510792 | 0.2485783 | 0.0751505 | 0.3077992 | 0.807 |
| AT v.s. C | 0.3401229 | 0.0809141 | 0.0972219 | 0.3809752 | 0.242901 | 0.3895654 | 0.533 |
| RT v.s. HIIT | -0.0257243 | 0.4914144 | -0.1961875 | 0.2216495 | 0.1704632 | 0.5397516 | 0.752 |
| RT v.s. C | 0.0677088 | 0.1620739 | 0.5584008 | 0.3285039 | -0.490692 | 0.3655357 | 0.179 |
| HIIT v.s. C | 0.0850983 | 0.4423507 | 0.3674843 | 0.1742998 | -0.2823859 | 0.4750764 | 0.552 |
| TCEs v.s. C | 0.3593684 | 0.1533487 | 0.1814583 | 0.3612976 | 0.1779101 | 0.3943002 | 0.652 |

**Table S5.11:** Side-splitting of WC. Inconsistency test between direct and indirect treatment comparisons in mixed treatment comparison.

| Comparison | Direct | | Indirect | | Difference | | |
| --- | --- | --- | --- | --- | --- | --- | --- |
|  | Coef. | Std.Err. | Coef. | Std.Err. | Coef. | Std.Err. | P>\|z\| |
| AT v.s. RT | 0.3485681 | 0.0886658 | 0.0547179 | 0.3551851 | 0.2938501 | 0.366881 | 0.423 |
| AT v.s. HIIT | -0.0231064 | 0.229355 | 0.0812596 | 0.4041522 | -0.104366 | 0.4646787 | 0.822 |
| AT v.s. TCEs | 0.0333034 | 0.1732303 | 0.5020589 | 0.1828135 | -0.4687555 | 0.2513217 | 0.062 |
| AT v.s. C | 0.069473 | 0.2064333 | -0.6966157 | 0.4120217 | 0.7660886 | 0.4634624 | 0.098 |
| RT v.s. HIIT | -0.3391665 | 0.4389382 | -0.3248403 | 0.2387112 | -0.0143262 | 0.4990135 | 0.977 |
| RT v.s. C | 0.0056224 | 0.1215689 | -0.7322283 | 0.3352325 | 0.7378507 | 0.3558378 | 0.078 |
| HIIT v.s. C | -0.5856538 | 0.2093936 | 0.1804348 | 0.4073174 | -0.7660886 | 0.4634627 | 0.098 |
| TCEs v.s. C | 0.0602921 | 0.4852661 | 0.3037629 | 0.2638524 | -0.2434707 | 0.5569674 | 0.662 |

# Appendix 6: Network maps and forest plots of secondary outcomes

**Figure S6.1:** Network map of the effect on HbA1c, and forest plot of network effect sizes for compared with control. The size of the nodes was proportional to the number of participants included in the trial, and the thickness of lines between the interventions relates to the number of studies for that comparison.


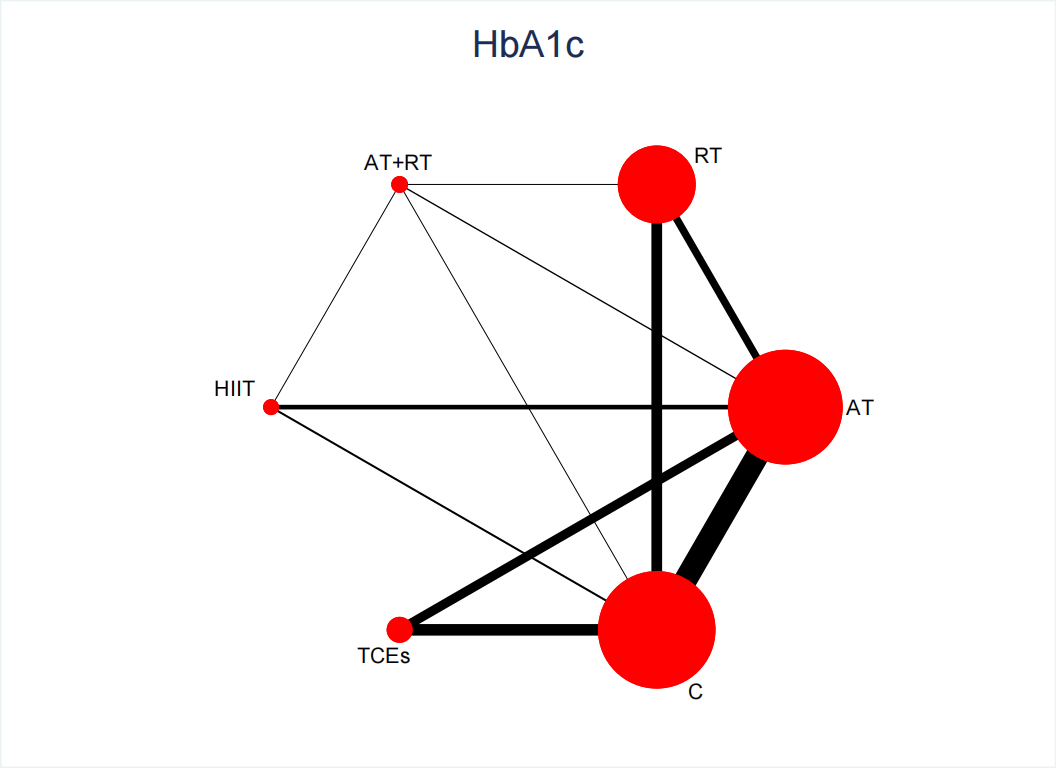


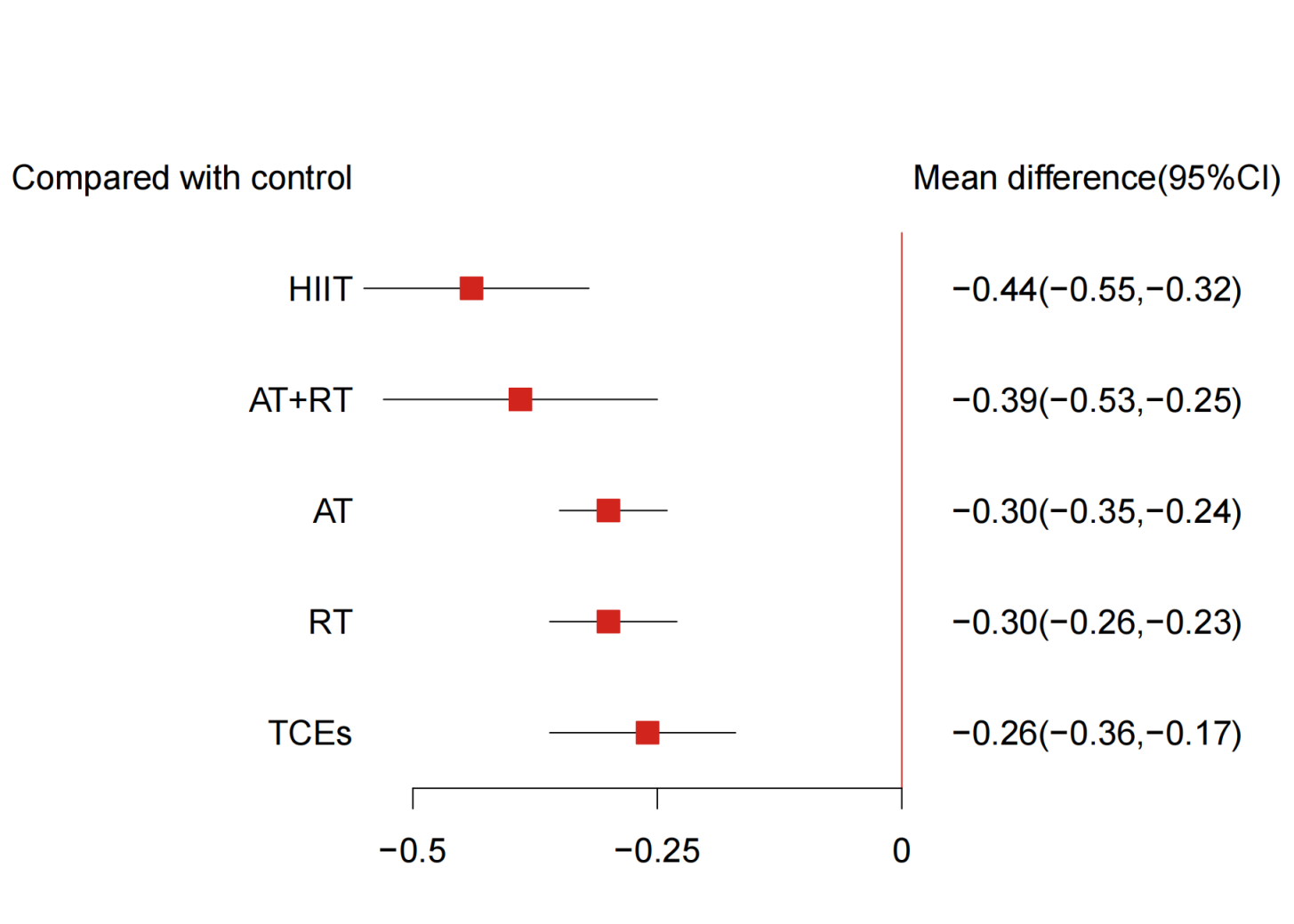


**Figure S6.2:** Network map of the effect on FBG, and forest plot of network effect sizes for compared with control. The size of the nodes was proportional to the number of participants included in the trial, and the thickness of lines between the interventions relates to the number of studies for that comparison.

**
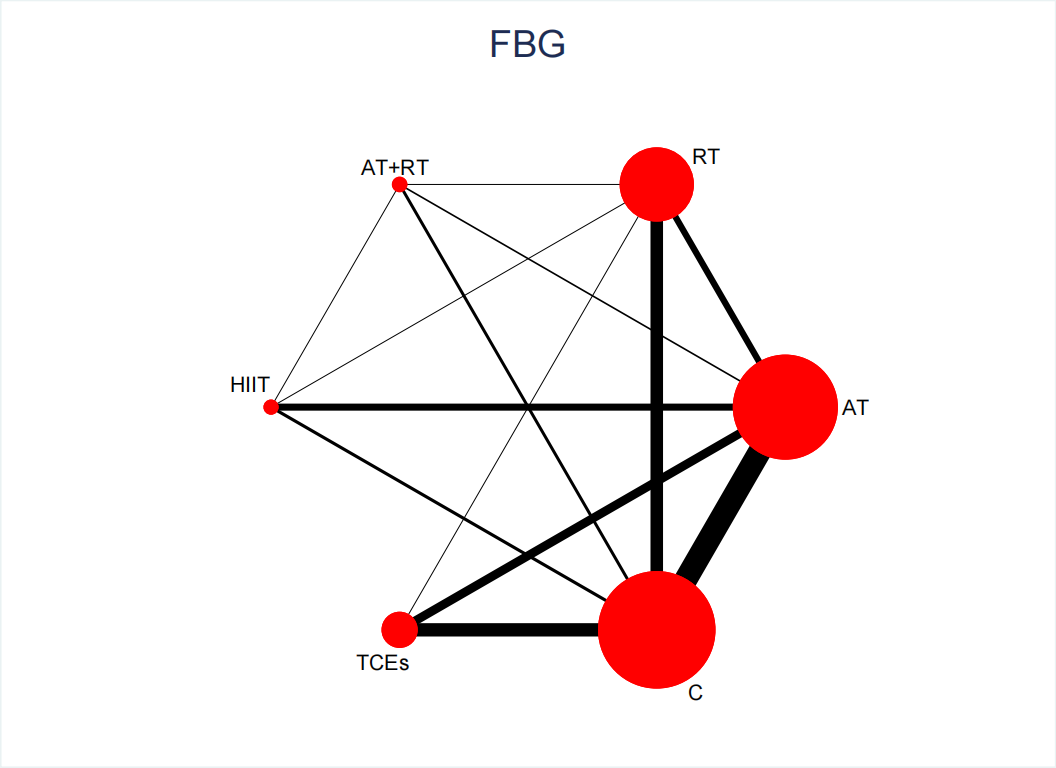
**

**
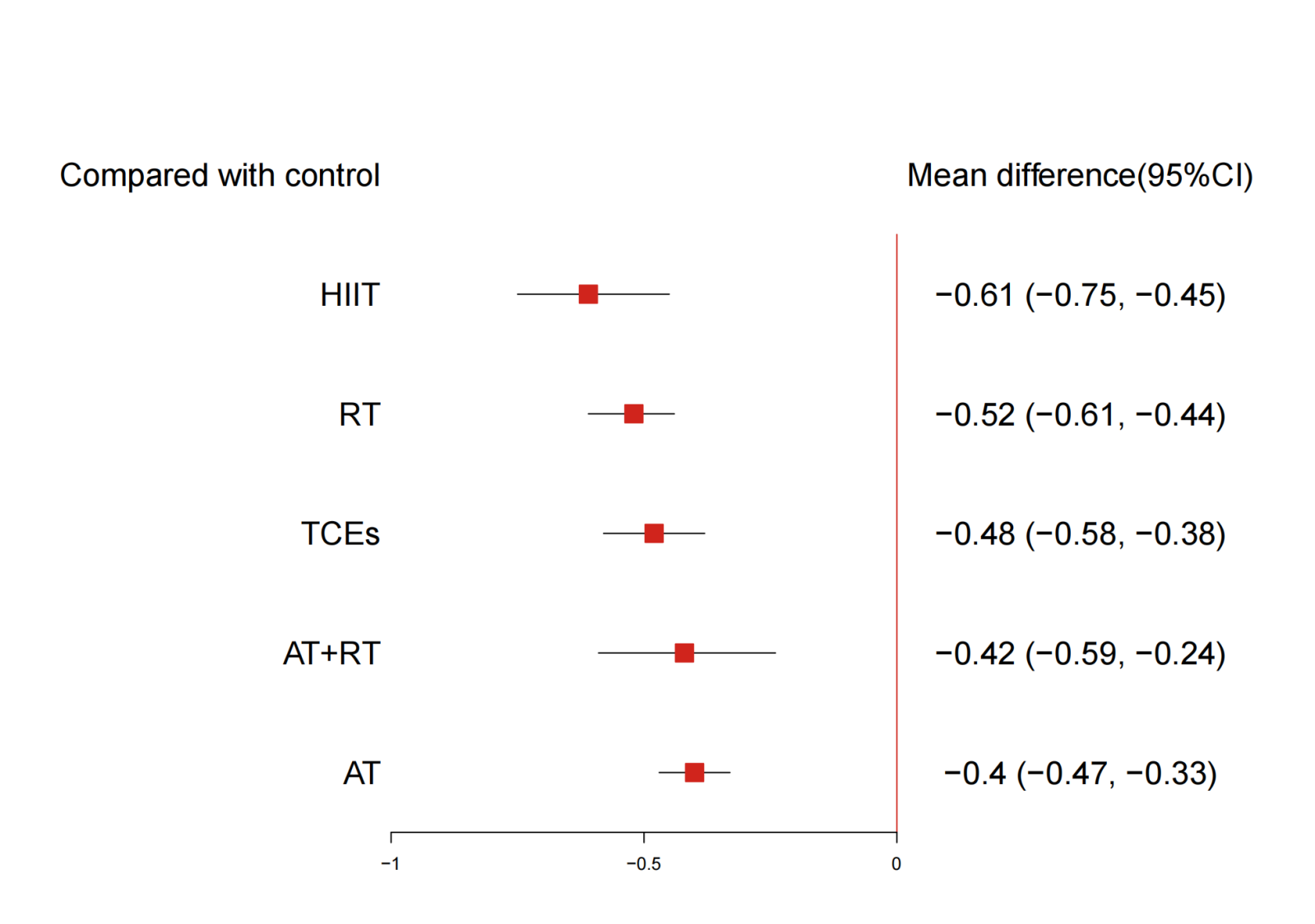
**

**Figure S6.3:** Network map of the effect on 2hPG, and forest plot of network effect sizes for compared with control. The size of the nodes was proportional to the number of participants included in the trial, and the thickness of lines between the interventions relates to the number of studies for that comparison.


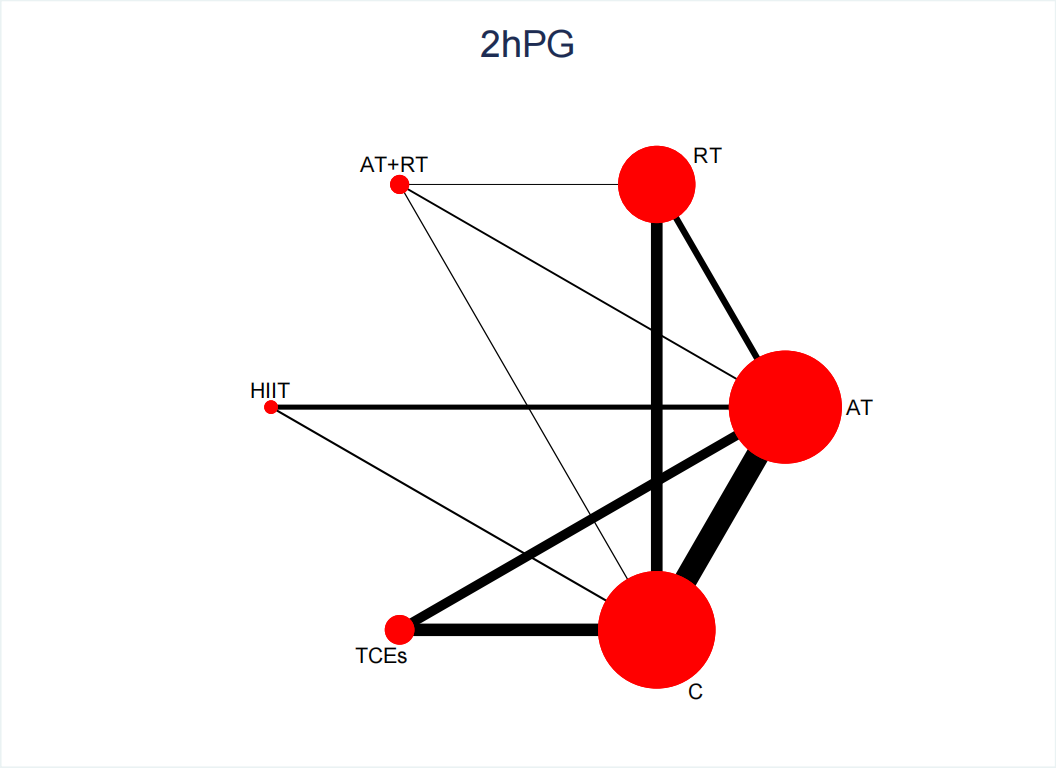


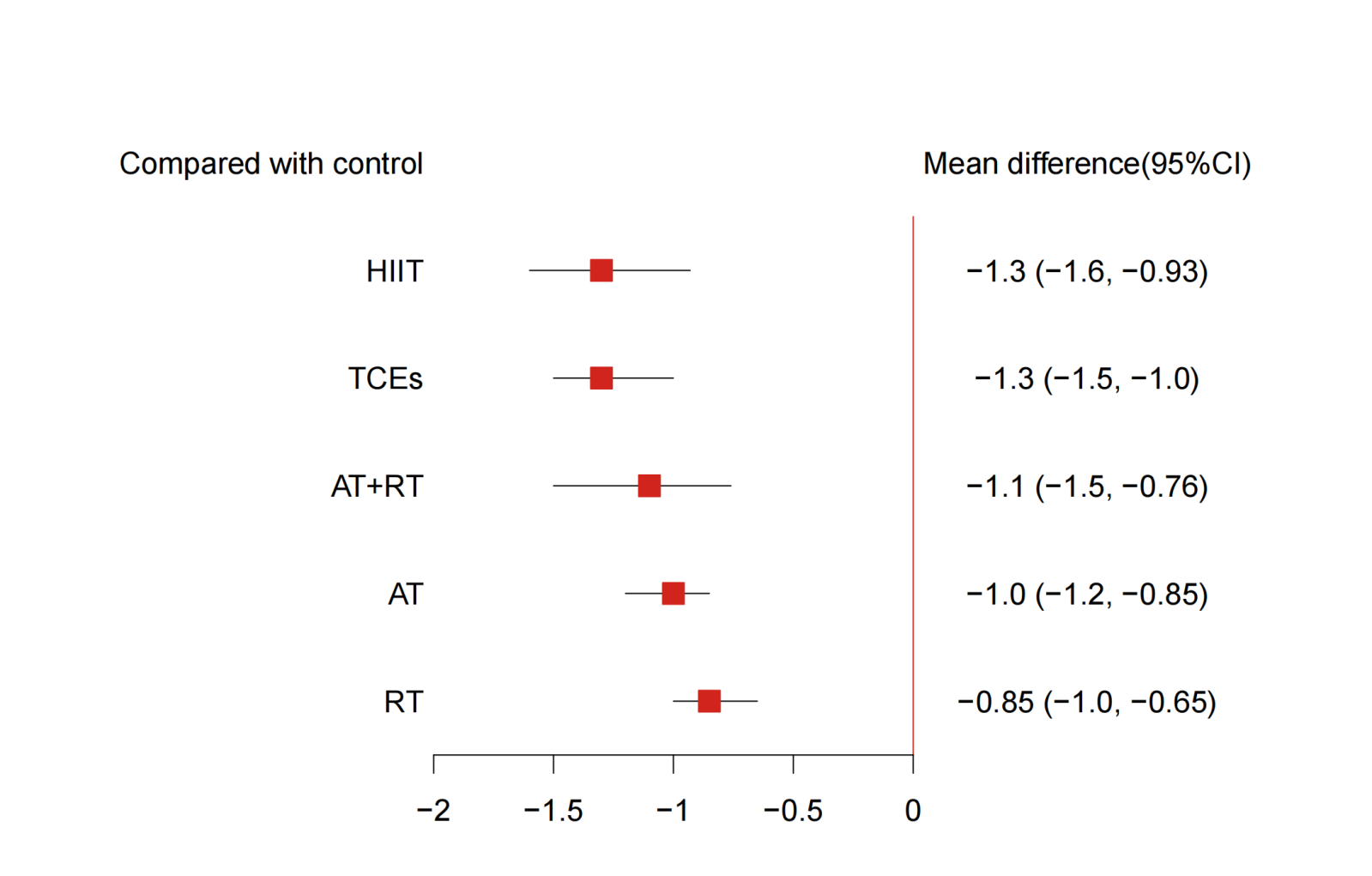


**Figure S6.4:** Network map of the effect on TC, and forest plot of network effect sizes for compared with control. The size of the nodes was proportional to the number of participants included in the trial, and the thickness of lines between the interventions relates to the number of studies for that comparison.


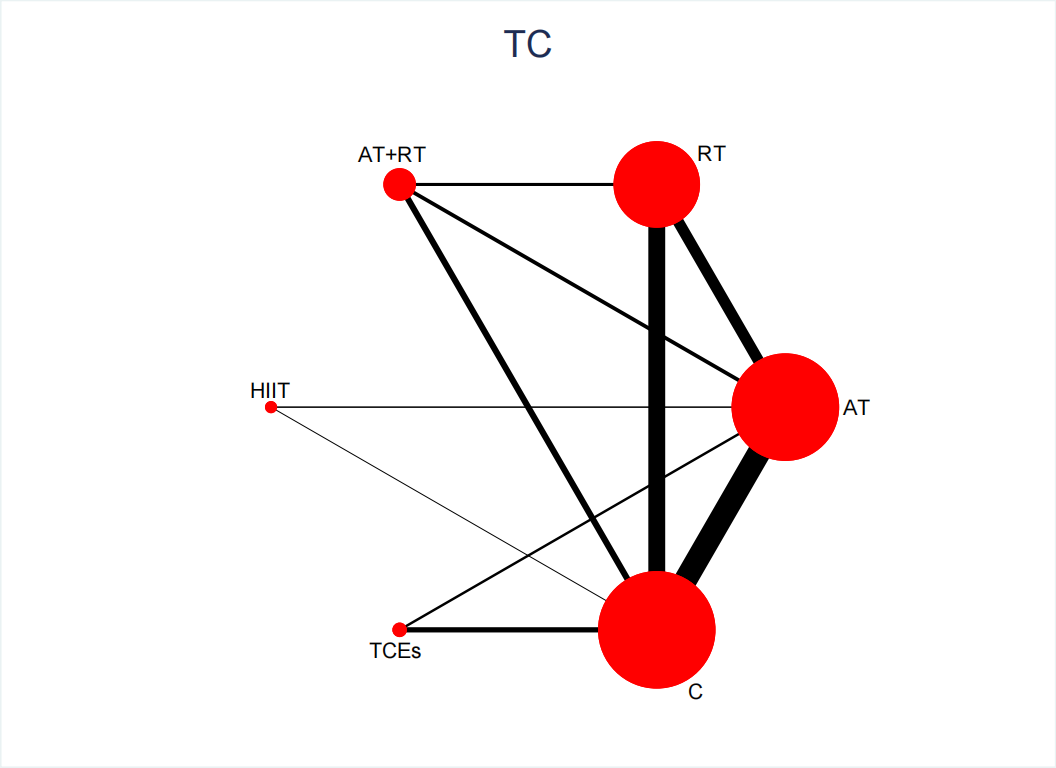


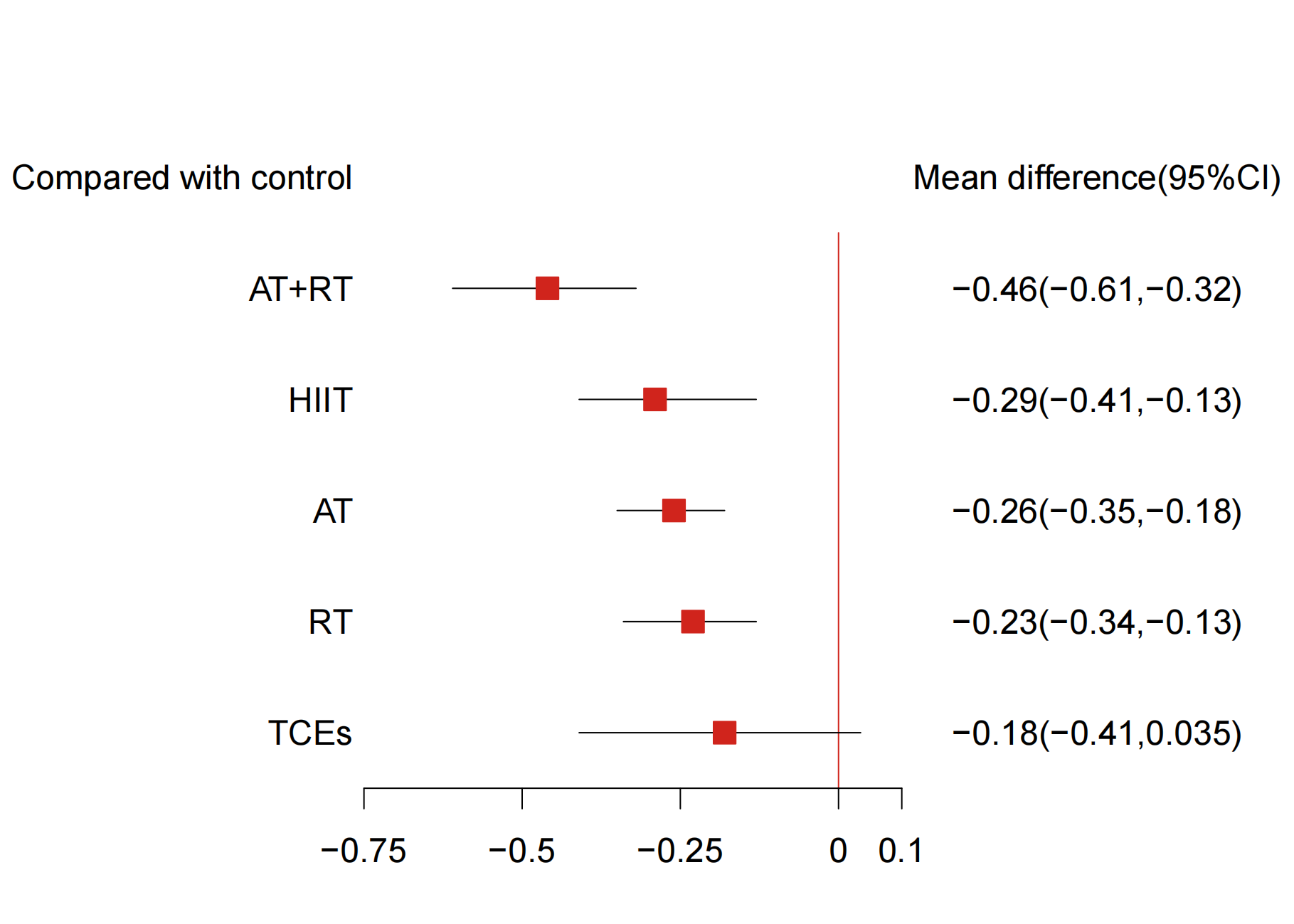


**Figure S6.5:** Network map of the effect on TG, and forest plot of network effect sizes for compared with control. The size of the nodes was proportional to the number of participants included in the trial, and the thickness of lines between the interventions relates to the number of studies for that comparison.


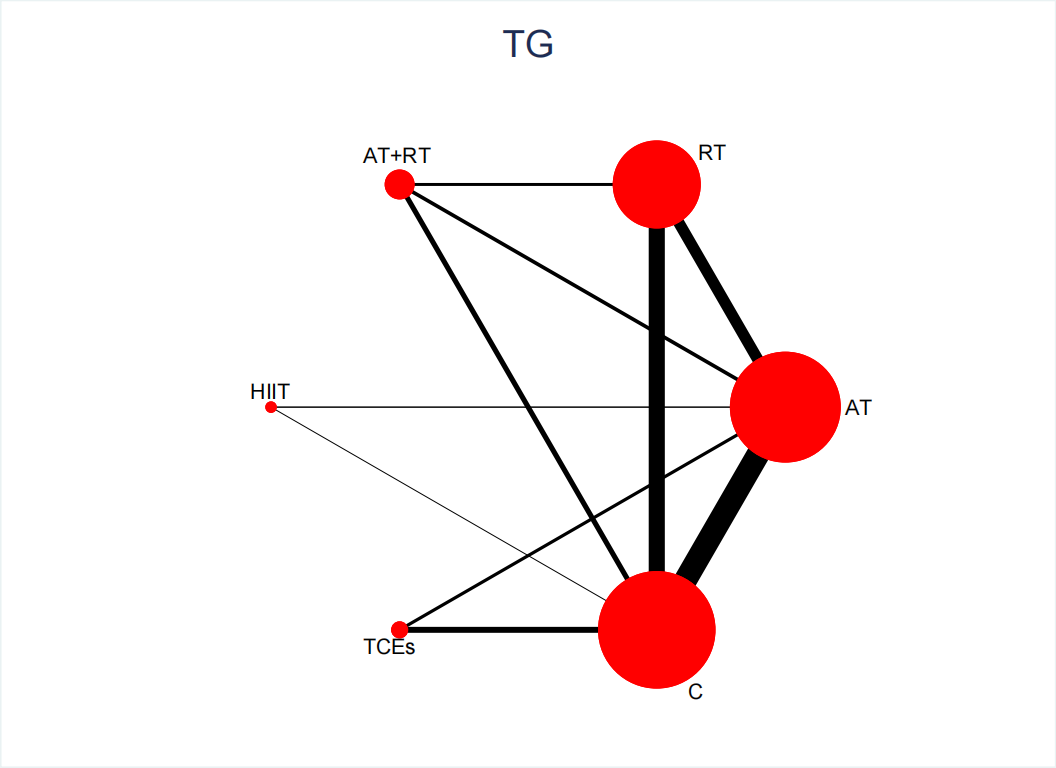


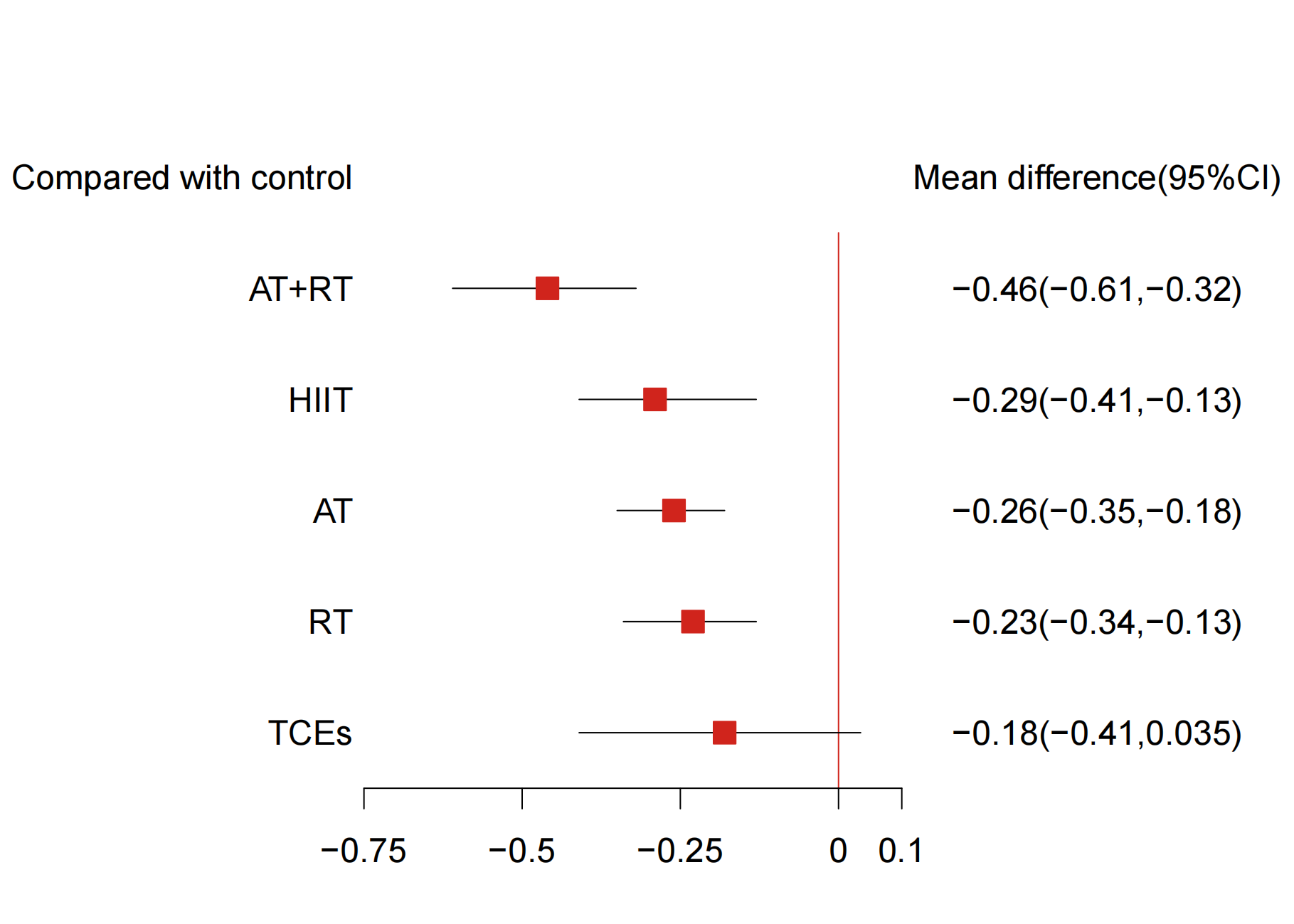


**Figure S6.6:** Network map of the effect on HDL, and forest plot of network effect sizes for compared with control. The size of the nodes was proportional to the number of participants included in the trial, and the thickness of lines between the interventions relates to the number of studies for that comparison.


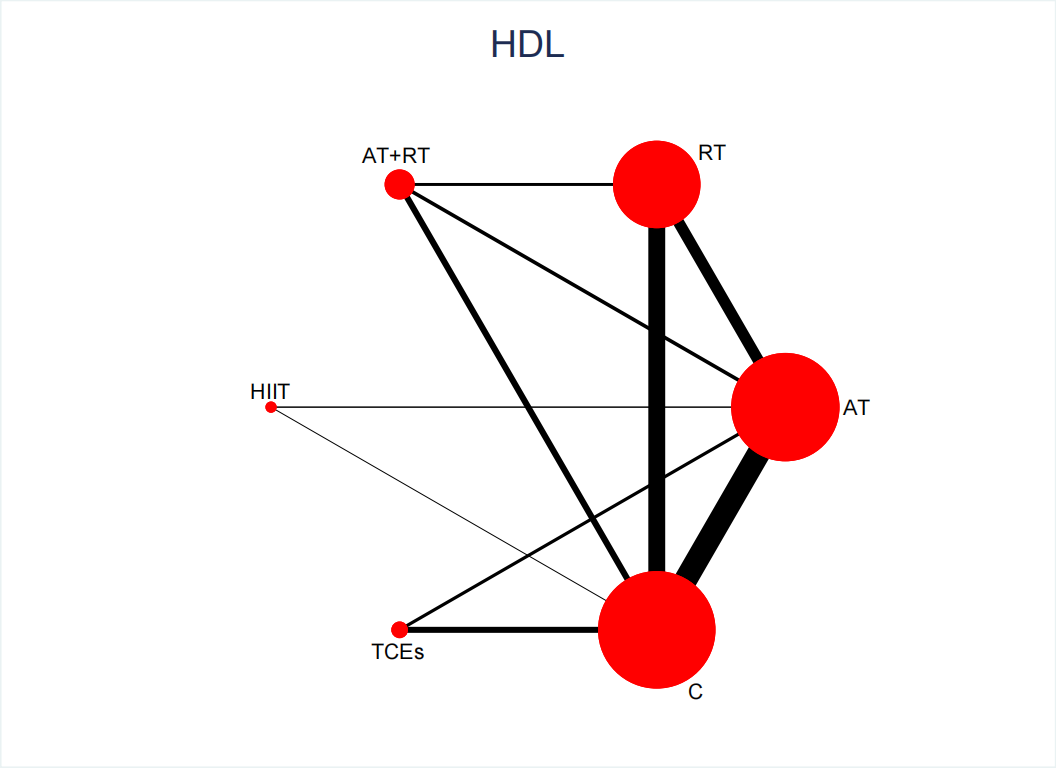


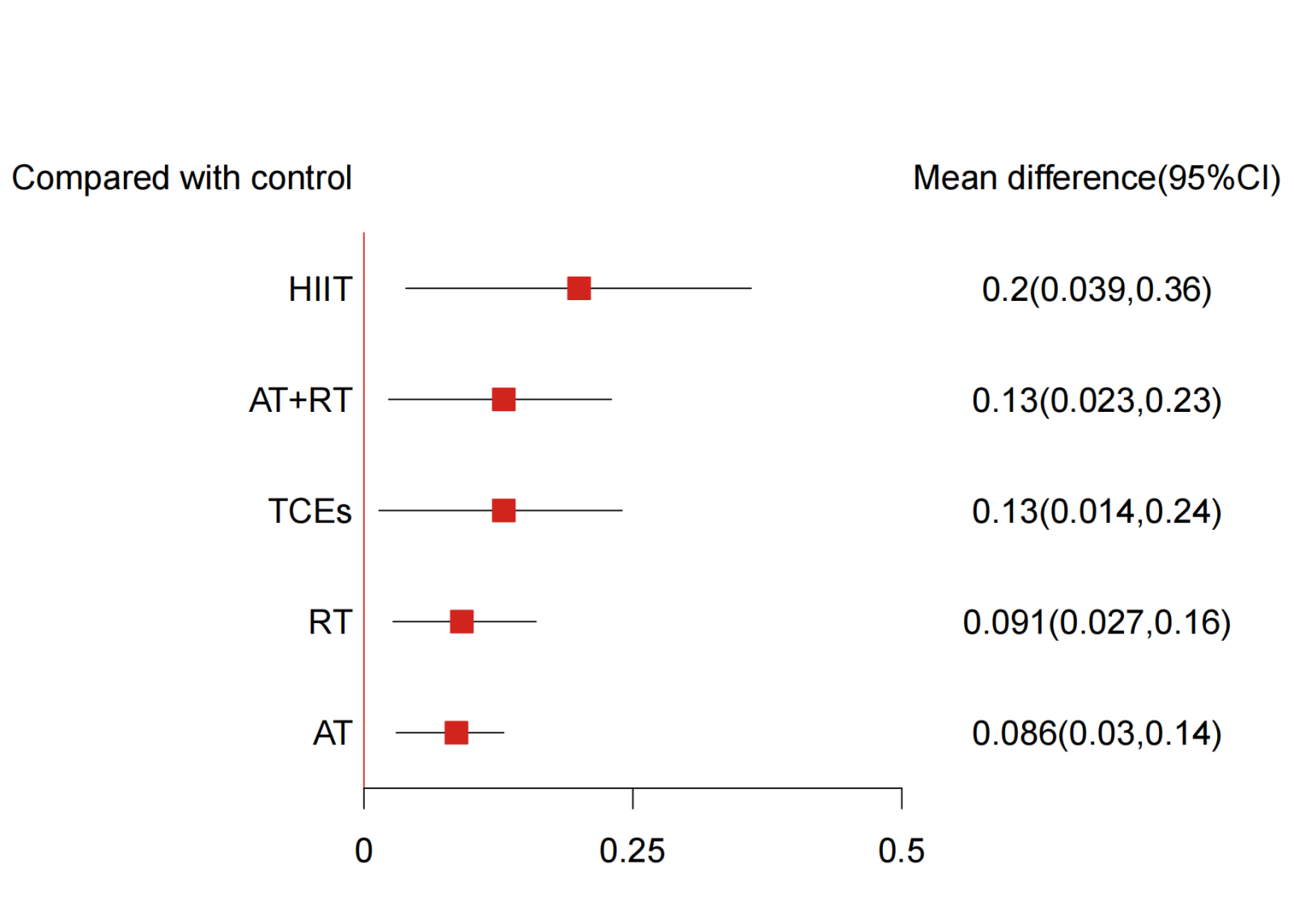


**Figure S6.7:** Network map of the effect on LDL, and forest plot of network effect sizes for compared with control. The size of the nodes was proportional to the number of participants included in the trial, and the thickness of lines between the interventions relates to the number of studies for that comparison.


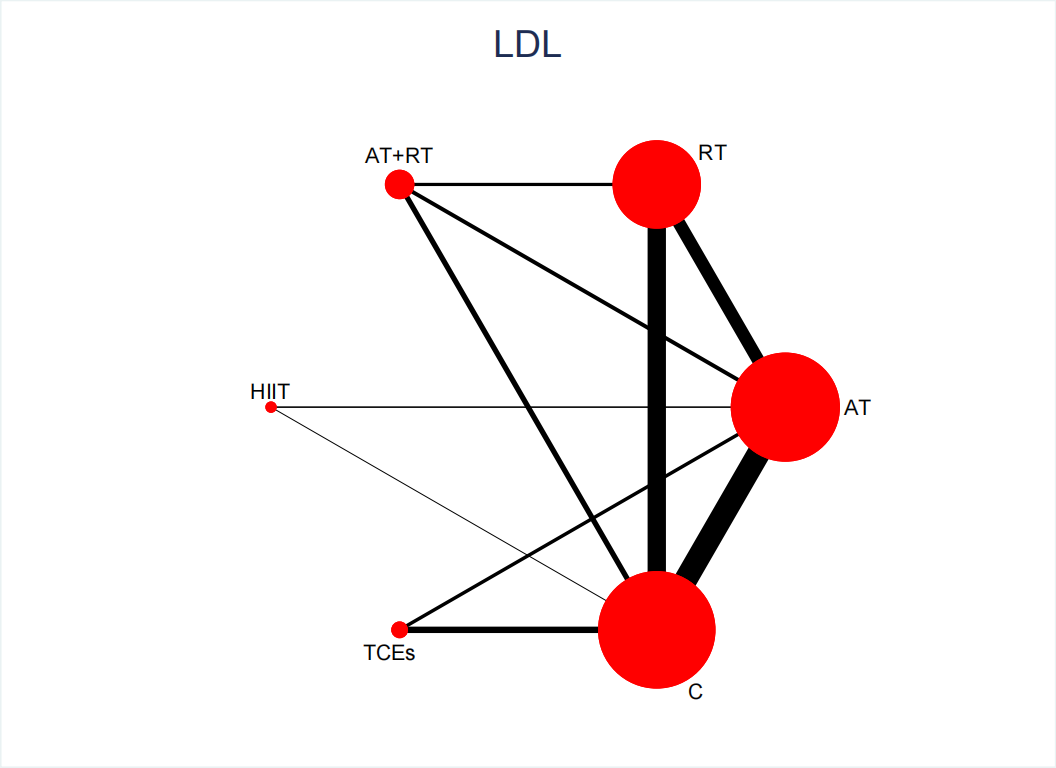


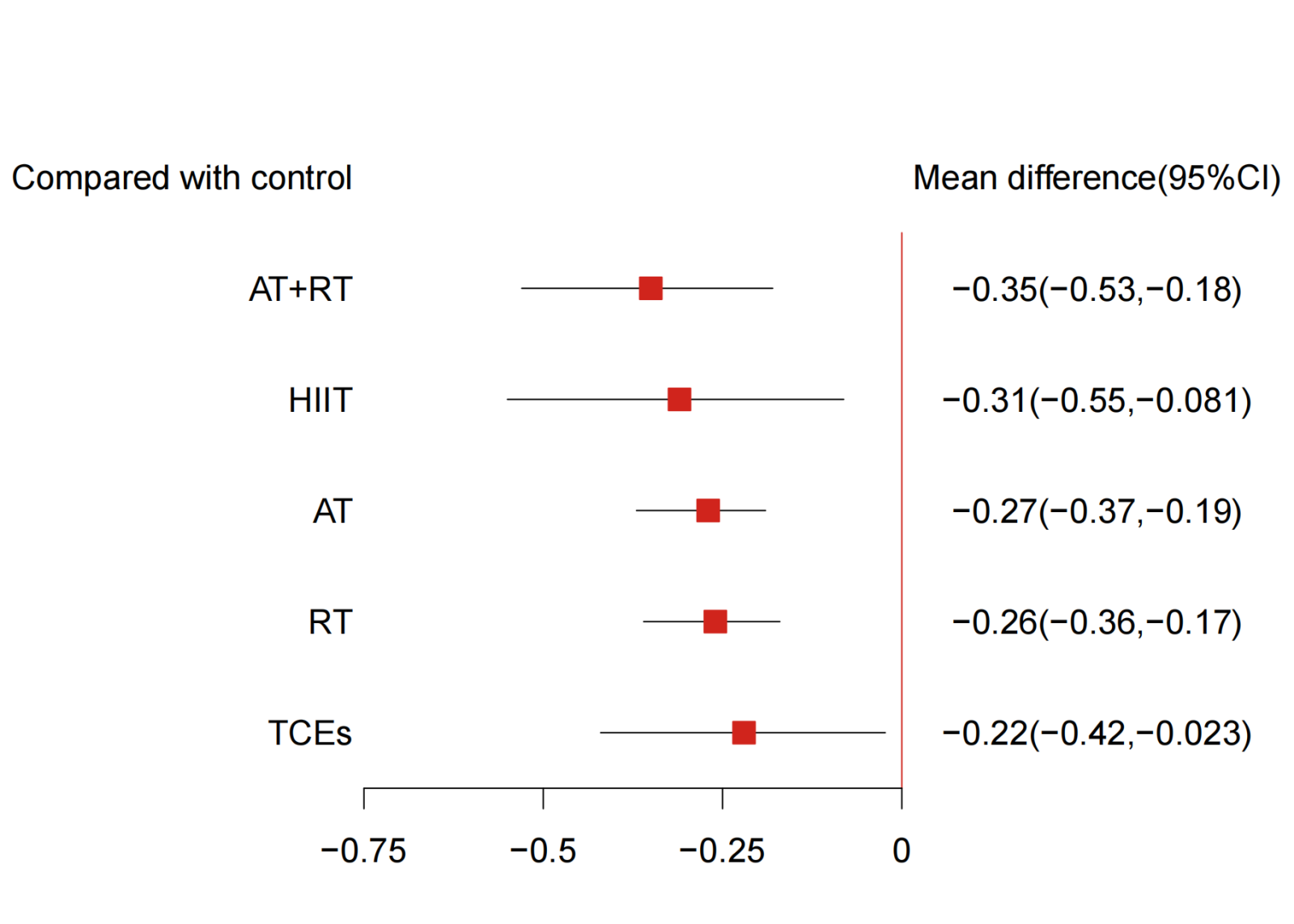


**Figure S6.8:** Network map of the effect on BMI, and forest plot of network effect sizes for compared with control. The size of the nodes was proportional to the number of participants included in the trial, and the thickness of lines between the interventions relates to the number of studies for that comparison.


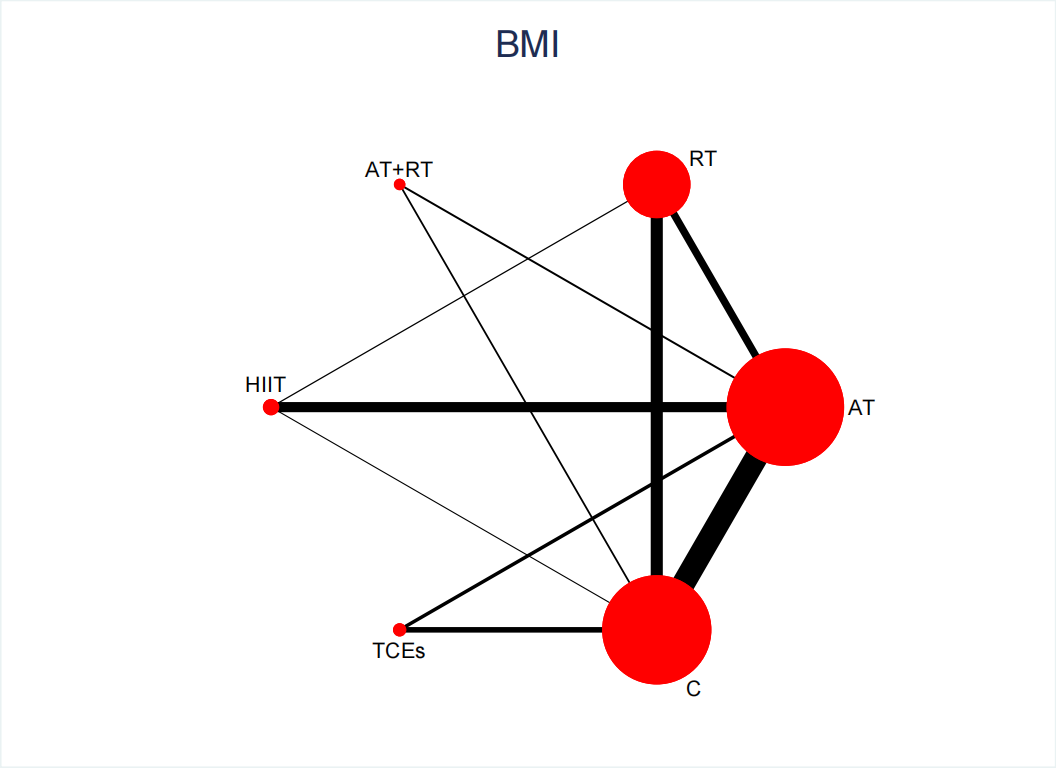


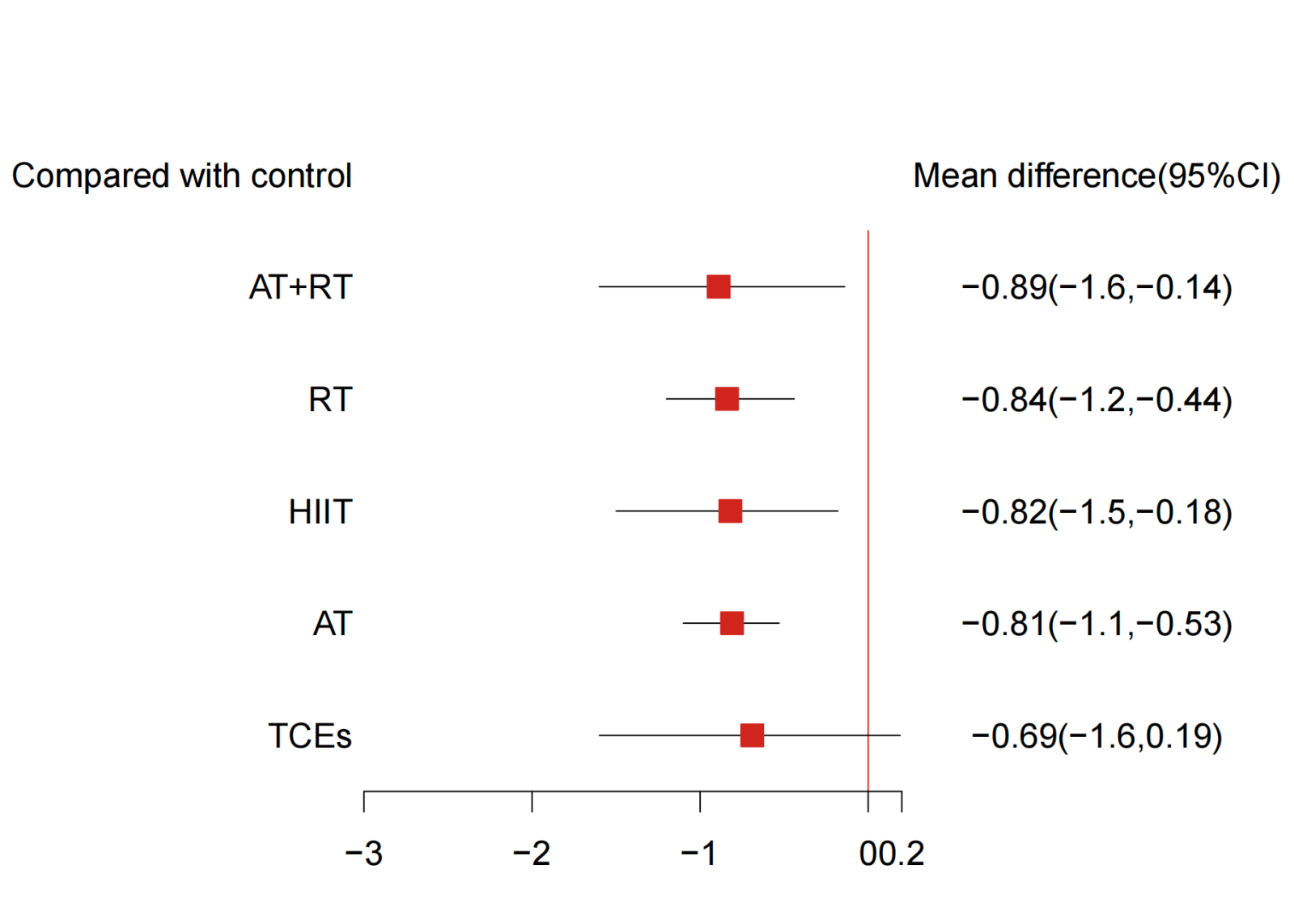


**Figure S6.9:** Network map of the effect on BW, and forest plot of network effect sizes for compared with control. The size of the nodes was proportional to the number of participants included in the trial, and the thickness of lines between the interventions relates to the number of studies for that comparison.


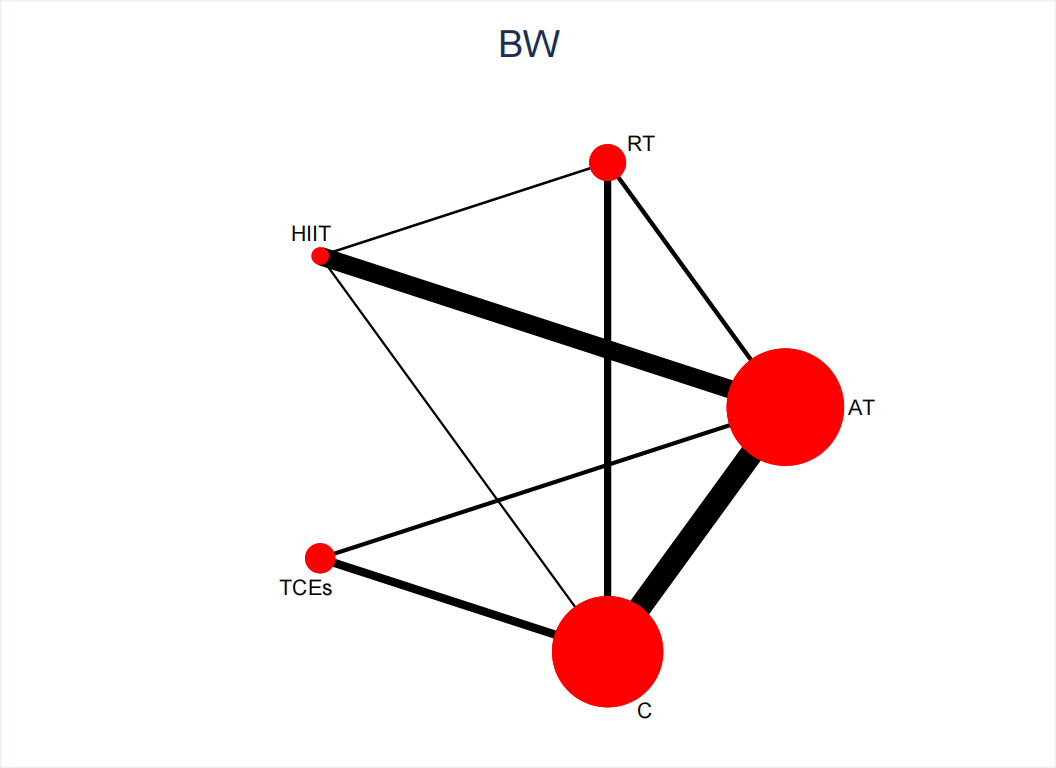


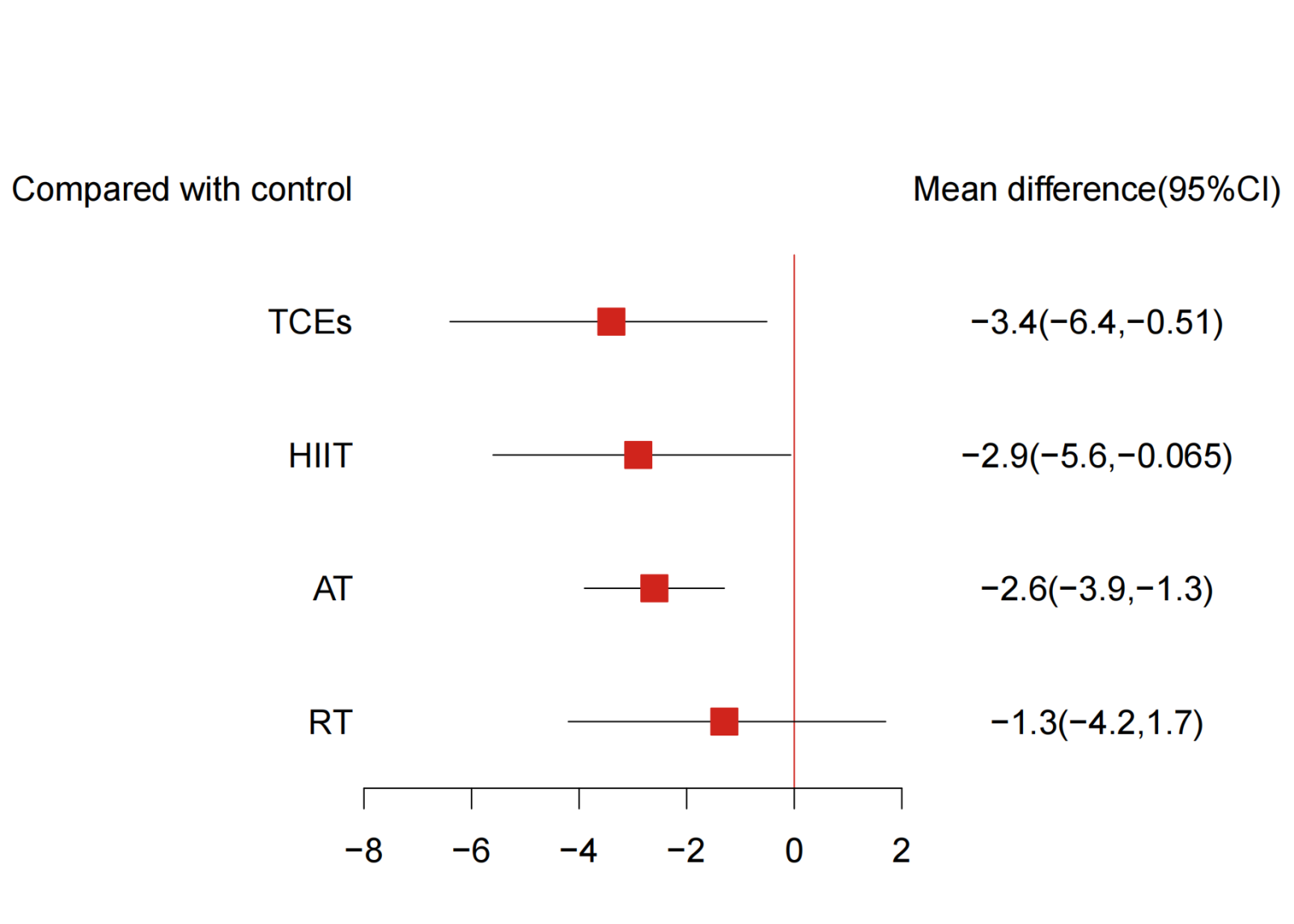


**Figure S6.10:** Network map of the effect on WC, and forest plot of network effect sizes for compared with control. The size of the nodes was proportional to the number of participants included in the trial, and the thickness of lines between the interventions relates to the number of studies for that comparison.


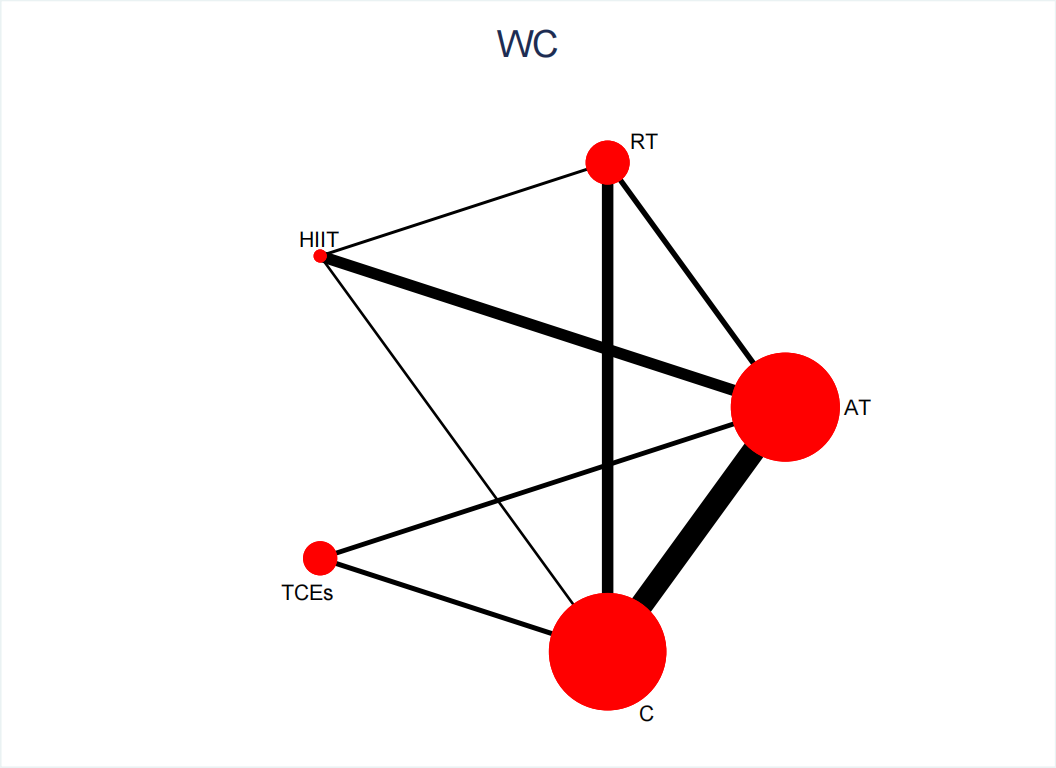

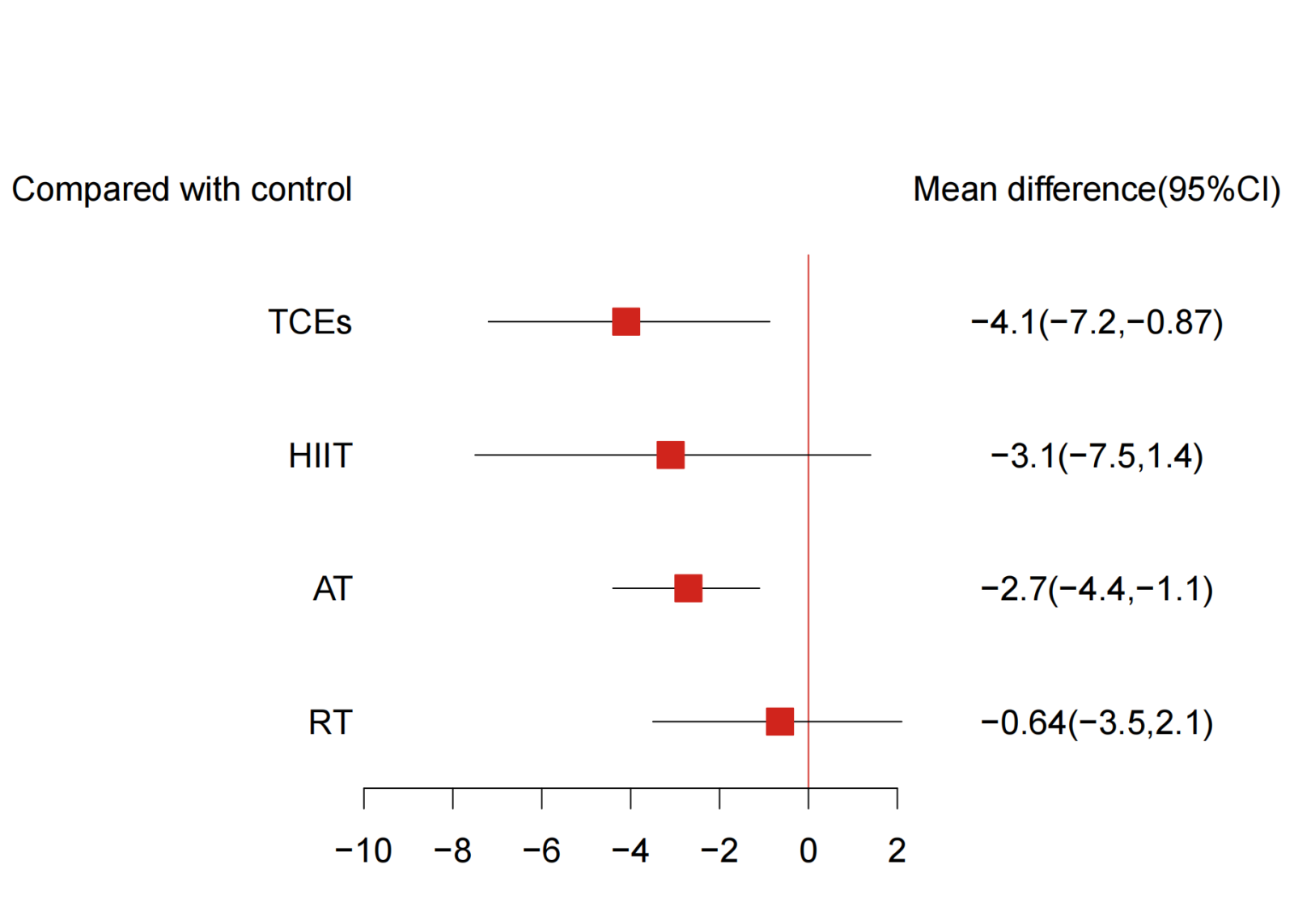


# Appendix 7: SUCRA and cumulative probability plots

**Figure S7.1:** SUCRA plots and cumulative probability plots of different exercise modalities for HbA1c in range network. Higher SUCRA values indicate a greater likelihood of the intervention being ranked higher, and cumulative probability plots show the probability distribution of each rank across interventions.


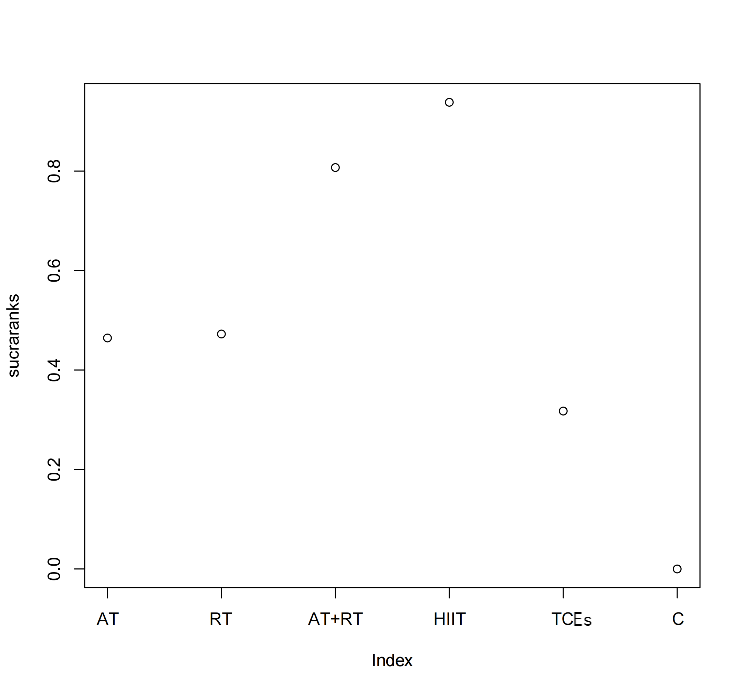


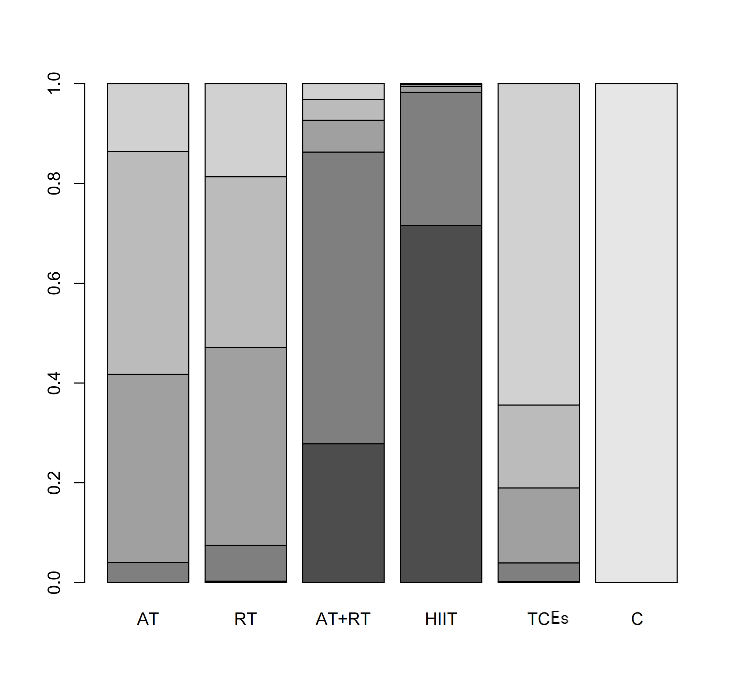


**Table S7.1:** SUCRA of the effects of different exercise modality on HbA1c.

| **Treatment** | **SUCRA** | **PrBest** |
| --- | --- | --- |
| HIIT | 93.8 | 71.9 |
| AT+RT | 80.7 | 27.4 |
| RT | 47.2 | 0.3 |
| AT | 46.4 | 0 |
| TCEs | 31.8 | 0.2 |
| C | 0 | 0 |

**Figure S7.2:** SUCRA plots and cumulative probability plots of different exercise modalities for 2hPG in range network. Higher SUCRA values indicate a greater likelihood of the intervention being ranked higher, and cumulative probability plots show the probability distribution of each rank across interventions.


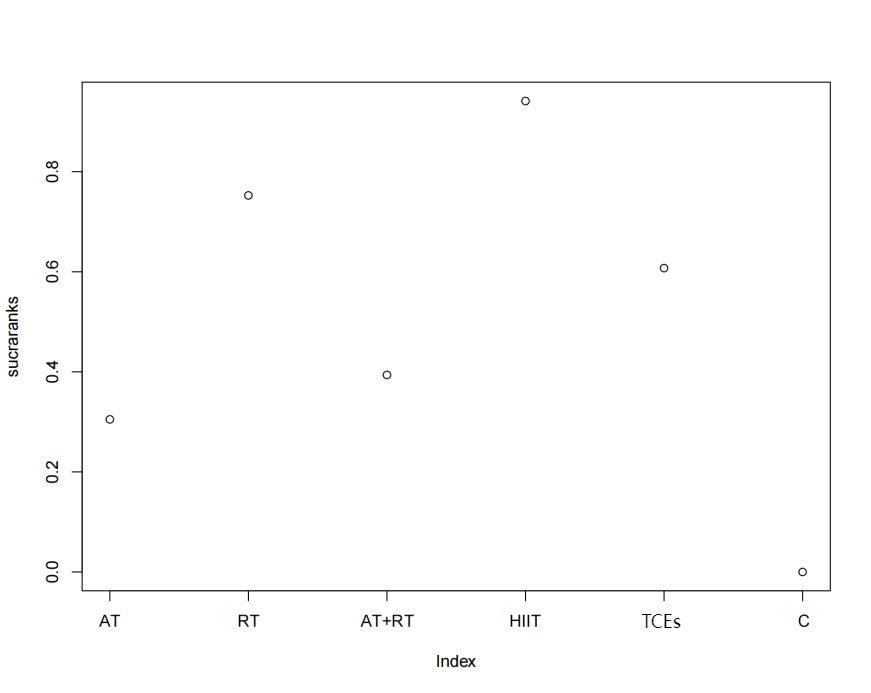


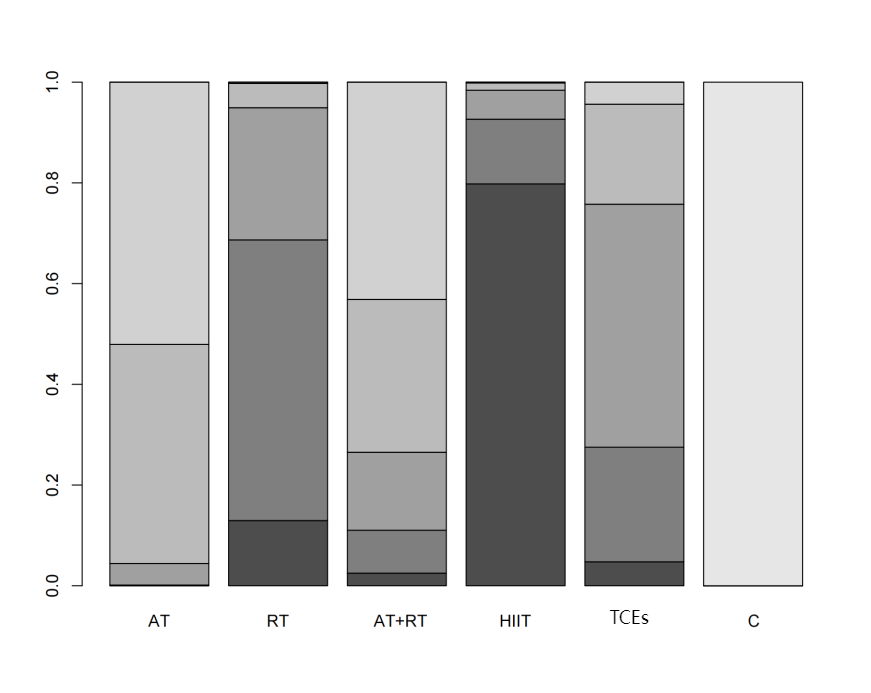


**Table S7.2:** SUCRA of the effects of different exercise modality on FBG.

| **Treatment** | **SUCRA** | **PrBest** |
| --- | --- | --- |
| HIIT | 94.1 | 79.8 |
| RT | 75.3 | 12.9 |
| TCEs | 60.7 | 4.8 |
| AT+RT | 39.4 | 2.5 |
| AT | 30.5 | 0 |
| C | 0 | 0 |

**Figure S7.3:** SUCRA plots and cumulative probability plots of different exercise modalities for 2hPG in range network. Higher SUCRA values indicate a greater likelihood of the intervention being ranked higher, and cumulative probability plots show the probability distribution of each rank across interventions.


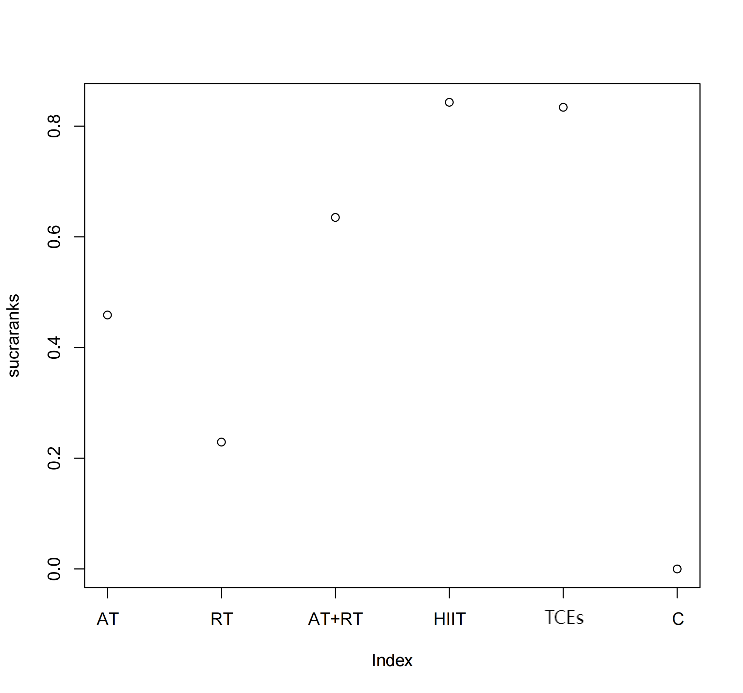


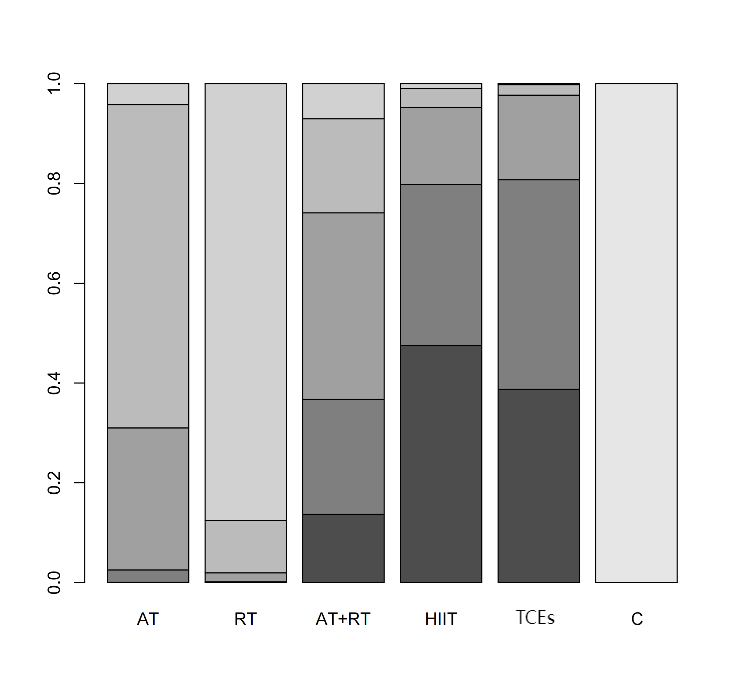


**Table S7.3:** SUCRA of the effects of different exercise modality on 2hPG.

| **Treatment** | **SUCRA** | **PrBest** |
| --- | --- | --- |
| HIIT | 84.3 | 48.2 |
| TCEs | 83.5 | 38.4 |
| AT+RT | 63.5 | 13.3 |
| AT | 45.9 | 0 |
| RT | 22.9 | 0 |
| C | 0 | 0 |

**Figure S7.4:** SUCRA plots and cumulative probability plots of different exercise modalities for TC in range network. Higher SUCRA values indicate a greater likelihood of the intervention being ranked higher, and cumulative probability plots show the probability distribution of each rank across interventions.


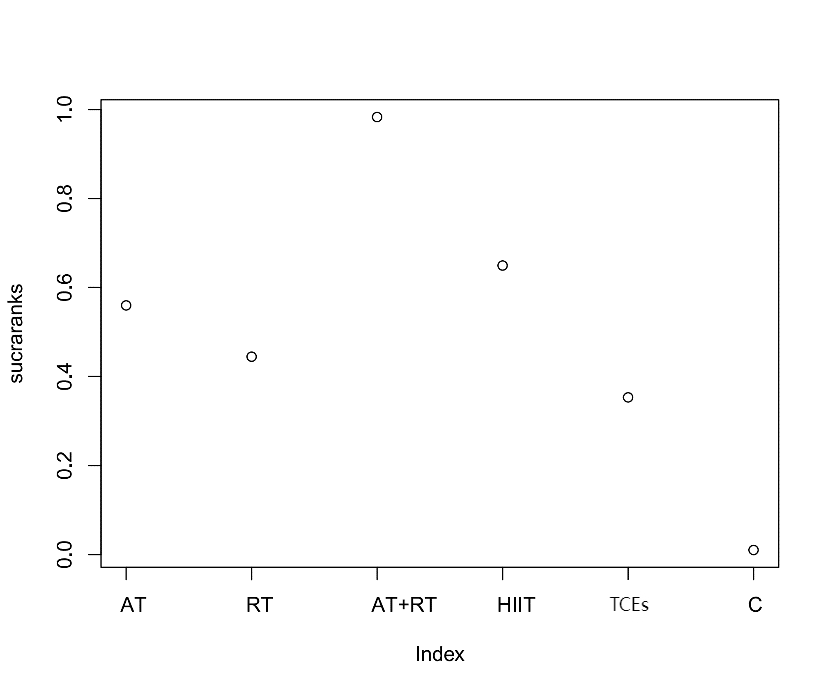
.
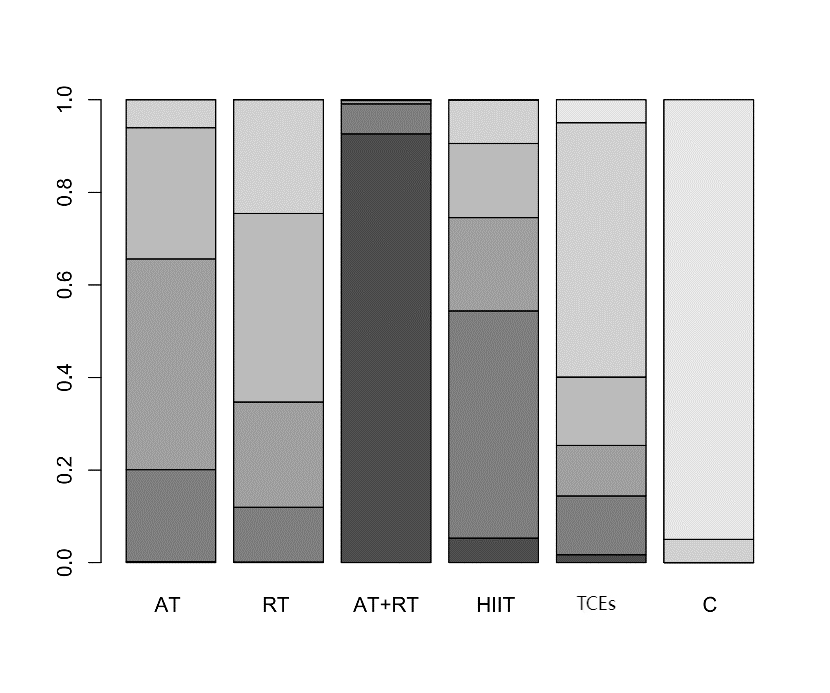


**Table S7.4:** SUCRA of the effects of different exercise modality on TC.

| **Treatment** | **SUCRA** | **PrBest** |
| --- | --- | --- |
| AT+RT | 98.3 | 92.7 |
| HIIT | 64.9 | 5.3 |
| AT | 56.0 | 0.2 |
| RT | 44.5 | 0.1 |
| TCEs | 35.3 | 1.5 |
| C | 1.01 | 0 |

**Figure S7.5:** SUCRA plots and cumulative probability plots of different exercise modalities for TG in range network. Higher SUCRA values indicate a greater likelihood of the intervention being ranked higher, and cumulative probability plots show the probability distribution of each rank across interventions.


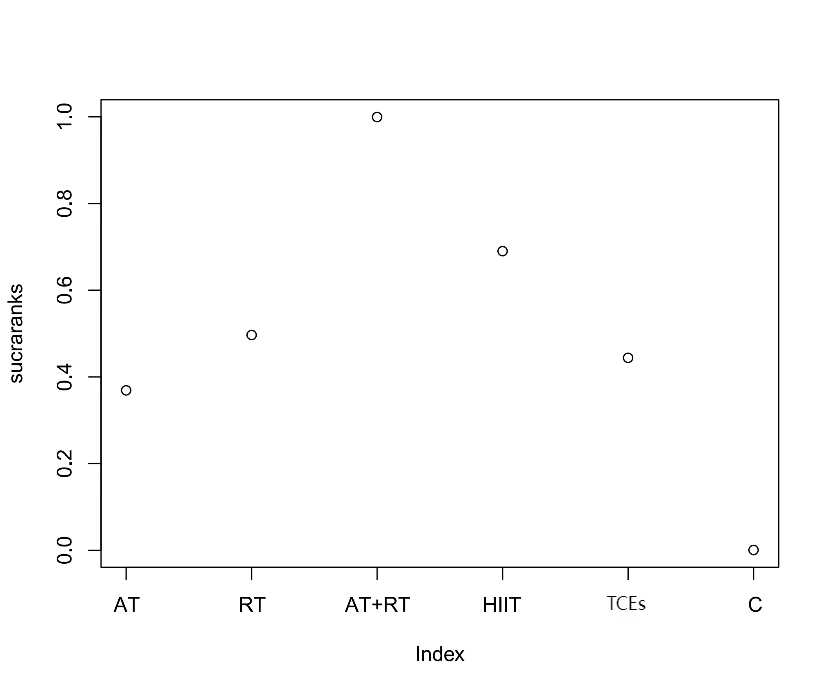

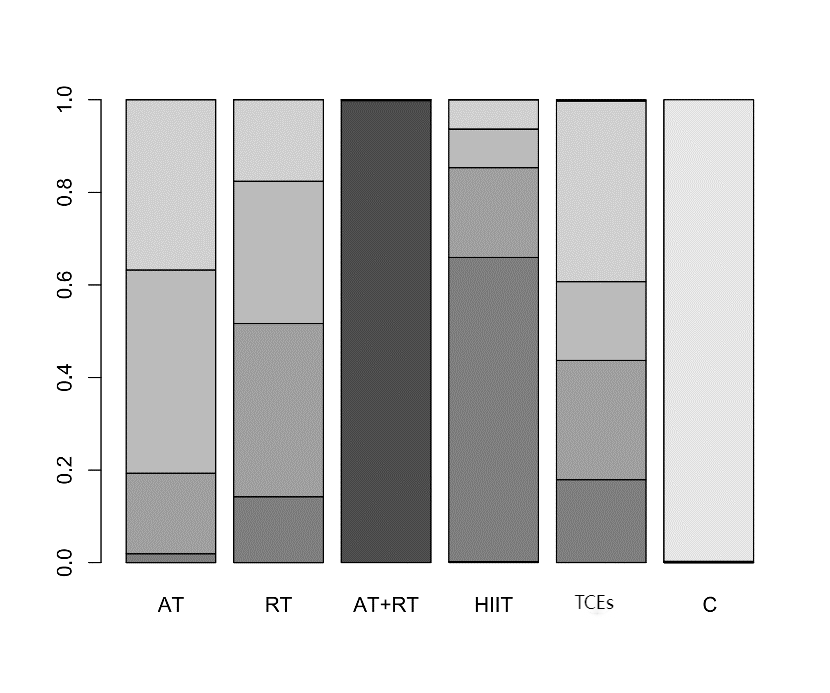


**Table S7.5:** SUCRA of the effects of different exercise modality on TG.

| **Treatment** | **SUCRA** | **PrBest** |
| --- | --- | --- |
| AT+RT | 99.9 | 99.7 |
| HIIT | 69.0 | 0.2 |
| RT | 49.7 | 0 |
| TCEs | 44.4 | 0 |
| AT | 36.9 | 0 |
| C | 0 | 0 |

**Figure S7.6:** SUCRA plots and cumulative probability plots of different exercise modalities for HDL in range network. Higher SUCRA values indicate a greater likelihood of the intervention being ranked higher, and cumulative probability plots show the probability distribution of each rank across interventions.


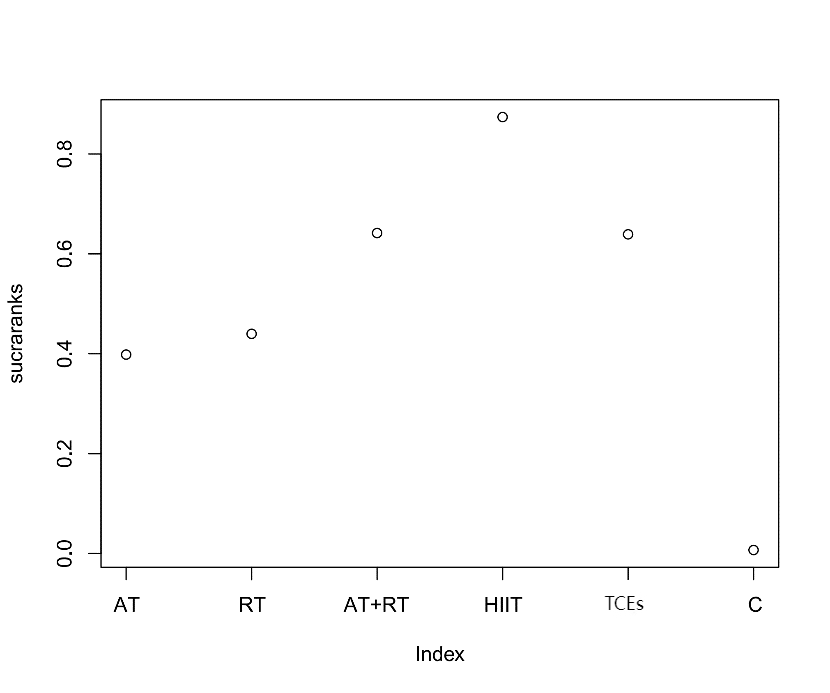

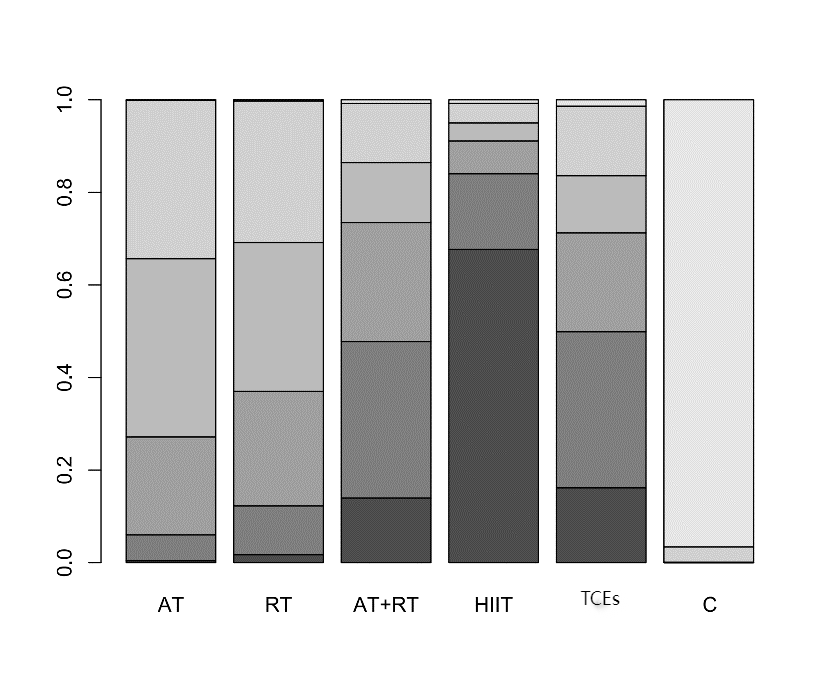


**Table S7.6:** SUCRA of the effects of different exercise modality on HDL.

| **Treatment** | **SUCRA** | **PrBest** |
| --- | --- | --- |
| HIIT | 87.3 | 67.6 |
| AT+RT | 64.2 | 13.9 |
| TCEs | 63.9 | 16.4 |
| RT | 44.0 | 1.7 |
| AT | 39.8 | 0.4 |
| C | 0.7 | 0 |

**Figure S7.7:** SUCRA plots and cumulative probability plots of different exercise modalities for LDL in range network. Higher SUCRA values indicate a greater likelihood of the intervention being ranked higher, and cumulative probability plots show the probability distribution of each rank across interventions.


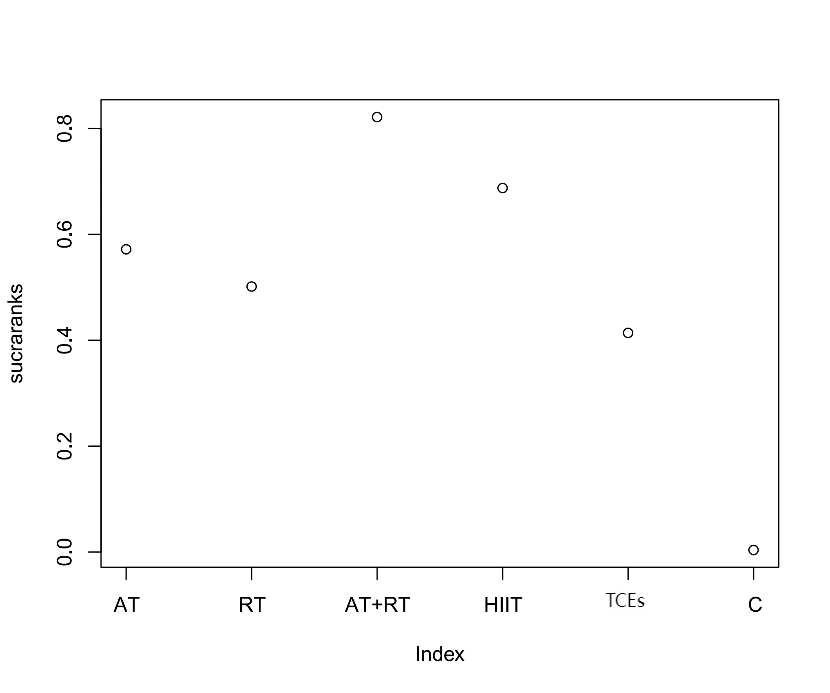

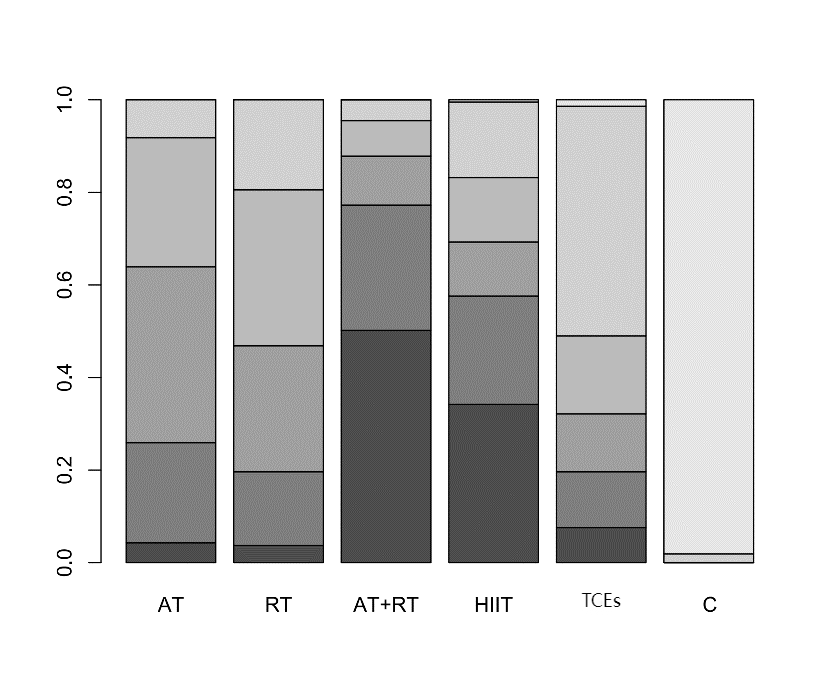


**Table S7.7:** SUCRA of the effects of different exercise modality on LDL.

| **Treatment** | **SUCRA** | **PrBest** |
| --- | --- | --- |
| AT+RT | 82.2 | 50.1 |
| HIIT | 68.7 | 34.2 |
| AT | 57.2 | 4.3 |
| RT | 50.1 | 3.7 |
| TCEs | 41.4 | 7.7 |
| C | 0.3 | 0 |

**Figure S7.8:** SUCRA plots and cumulative probability plots of different exercise modalities for BMI in range network. Higher SUCRA values indicate a greater likelihood of the intervention being ranked higher, and cumulative probability plots show the probability distribution of each rank across interventions.


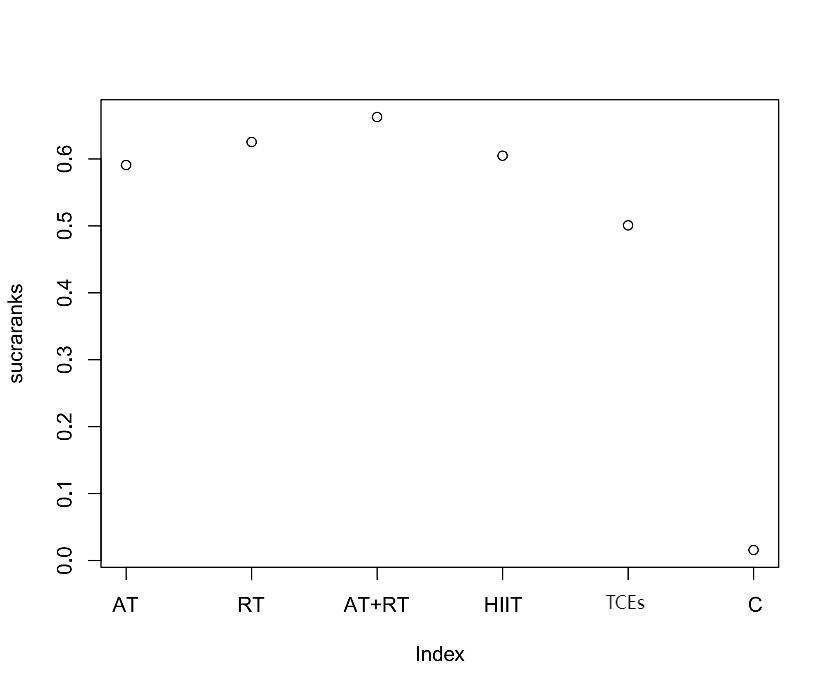

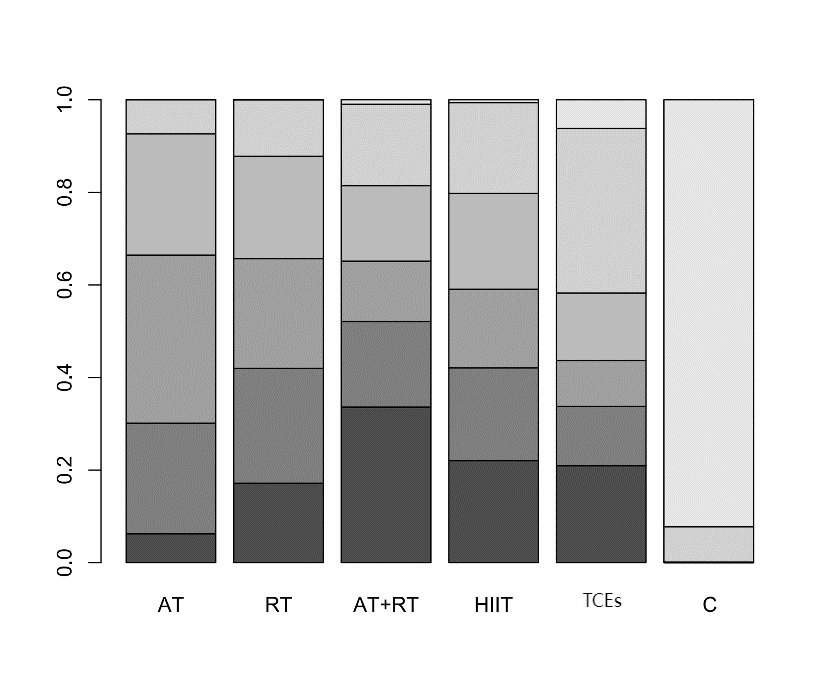


**Table S7.8:** SUCRA of the effects of different exercise modality on BMI.

| **Treatment** | **SUCRA** | **PrBest** |
| --- | --- | --- |
| AT+RT | 66.4 | 33.8 |
| RT | 62.5 | 17.1 |
| HIIT | 60.5 | 21.9 |
| AT | 59.1 | 6.1 |
| TCEs | 50.1 | 21.3 |
| C | 1.6 | 0 |

**Figure S7.9:** SUCRA plots and cumulative probability plots of different exercise modalities for BW in range network. Higher SUCRA values indicate a greater likelihood of the intervention being ranked higher, and cumulative probability plots show the probability distribution of each rank across interventions.


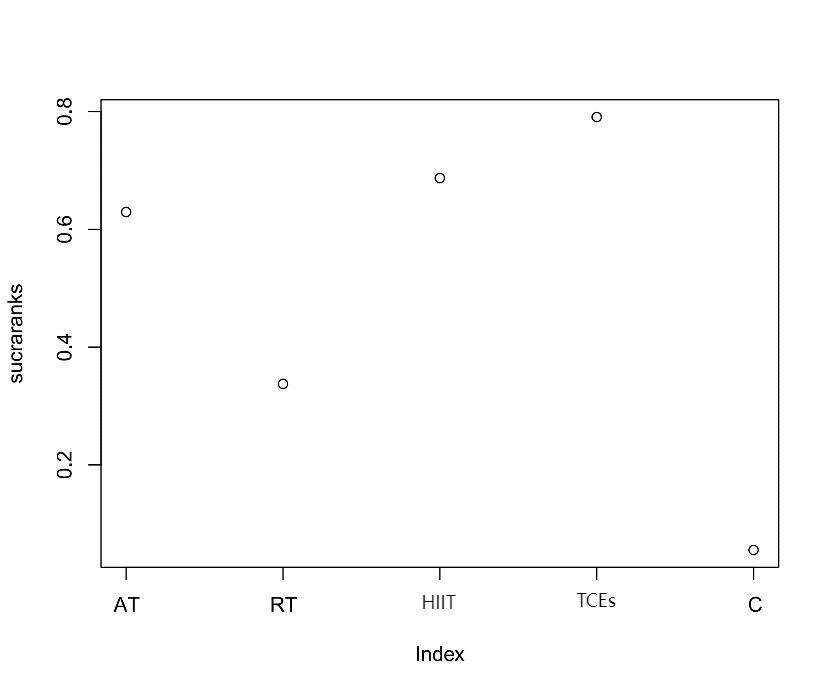

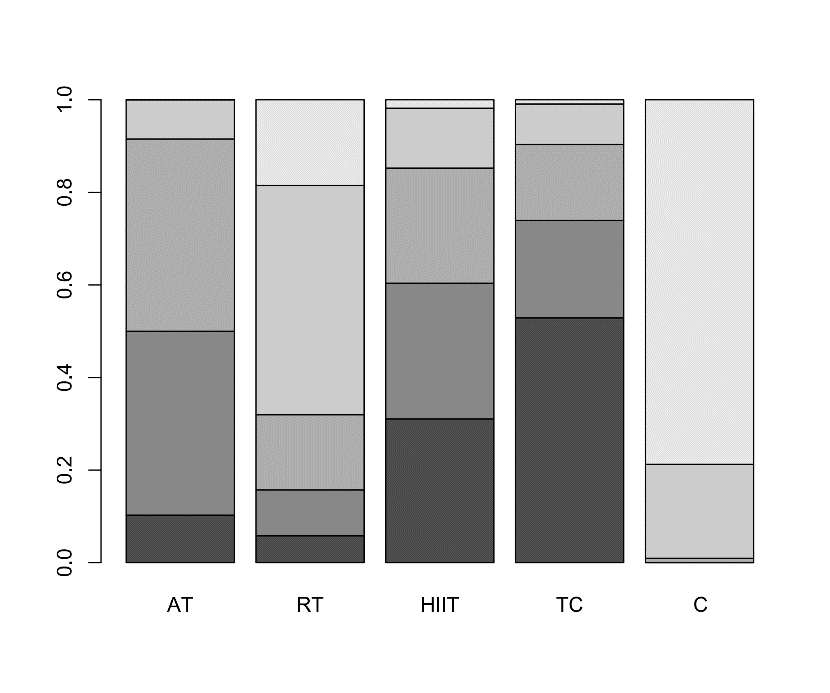


**Table S7.9:** SUCRA of the effects of different exercise modality on BW.

| **Treatment** | **SUCRA** | **PrBest** |
| --- | --- | --- |
| TCEs | 79.1 | 53.0 |
| HIIT | 68.7 | 30.7 |
| AT | 62.9 | 10.6 |
| RT | 33.8 | 5.7 |
| C | 5.6 | 0 |

**Figure S7.10:** SUCRA plots and cumulative probability plots of different exercise modalities for WC in range network. Higher SUCRA values indicate a greater likelihood of the intervention being ranked higher, and cumulative probability plots show the probability distribution of each rank across interventions.


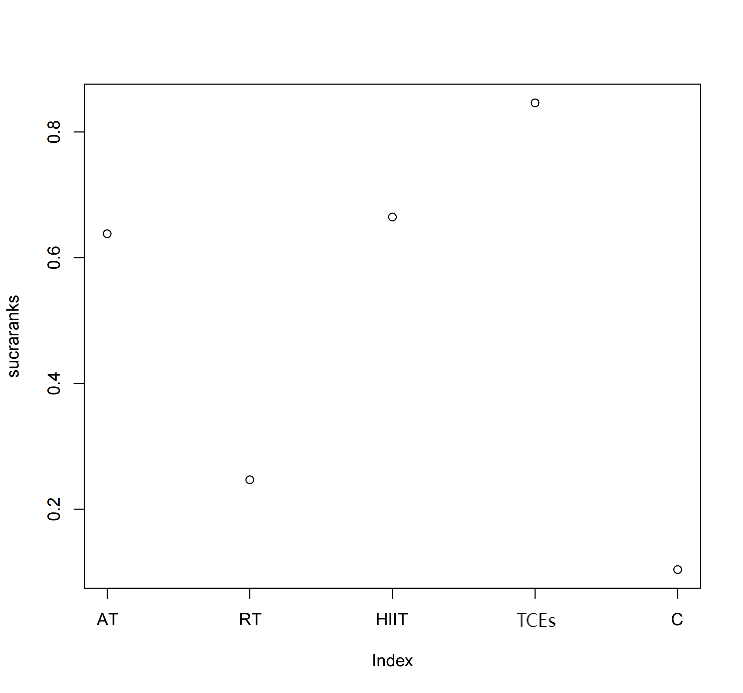


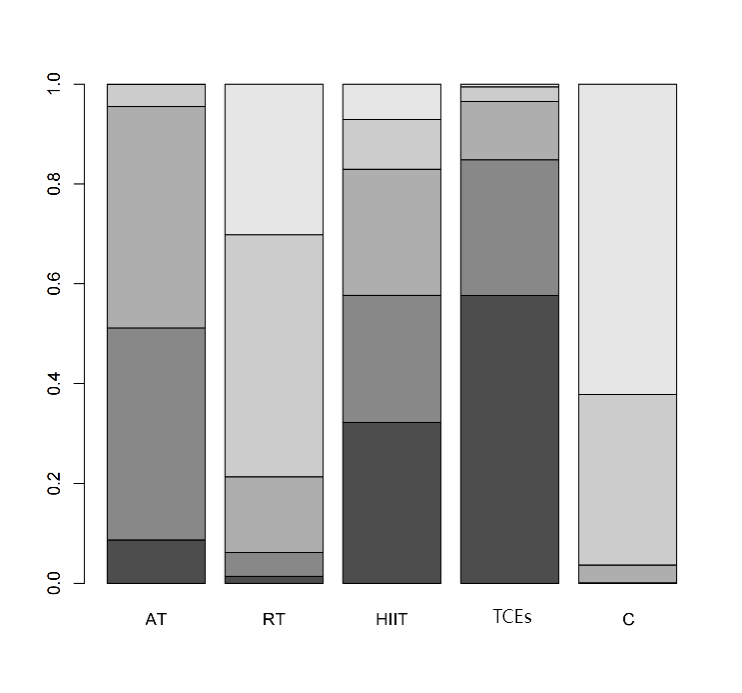


**Table S7.9:** SUCRA of the effects of different exercise modality on WC.

| **Treatment** | **SUCRA** | **PrBest** |
| --- | --- | --- |
| TCEs | 84.6 | 57.7 |
| HIIT | 66.5 | 32.2 |
| AT | 63.8 | 8.7 |
| RT | 24.9 | 1.4 |
| C | 10.4 | 0 |

# Appendix 8: league table of Summary Estimates for different exercise modality on prediabetes Derived from Network Meta-analysis

**Table S8.1**: league table of HbA1c

The columns represent the comparison of the row exercise modality to the column exercise modality. The rows represent the comparison of the row exercise modality to the column exercise modality. The effect estimates are expressed as mean difference and 95% confidence interval. For example, the mean difference in HbA1c for AT compared to RT is 0 (95% confidence interval -0.06 to 0.07). Mean difference <0 favors the exercise modality in the column, and mean difference >0 favors the exercise modality in the row. According to the network meta-analysis (CINeMA) framework, the certainty of the evidence for our comparisons of interest is included in the league table, with * indicating low confidence, † indicating moderate confidence, and ‡ indicating high confidence in the evidence. The full CINeMA assessment is shown in Appendix 9.

| AT |  |  |  |  |  |
| --- | --- | --- | --- | --- | --- |
| 0*  (-0.06, 0.07) | RT |  |  |  |  |
| 0.09*  (-0.04, 0.23) | 0.09*  (-0.05, 0.23) | AT+RT |  |  |  |
| 0.14*  (0.03, 0.25) | 0.14*  (0.02, 0.26) | 0.05*  (-0.12, 0.21) | HIIT |  |  |
| -0.03*  (-0.13, 0.06) | -0.03*  (-0.14, 0.07) | -0.13*  (-0.29, 0.03) | -0.17*  (-0.31, -0.03) | TCEs |  |
| -0.3†  (-0.35, -0.24) | -0.3†  (-0.36, -0.23) | -0.39†  (-0.53, -0.25) | -0.44†  (-0.55, -0.32) | -0.26*  (-0.36, -0.17) | C |

**Table S8.2**: league table of FBG

The columns represent the comparison of the row exercise modality to the column exercise modality. The rows represent the comparison of the row exercise modality to the column exercise modality. The effect estimates are expressed as mean difference and 95% confidence interval. For example, the mean difference in FBG for AT compared to RT is 0.12 (95% confidence interval -0.03 to 0.21). Mean difference <0 favors the exercise modality in the column, and mean difference >0 favors the exercise modality in the row. According to the network meta-analysis (CINeMA) framework, the certainty of the evidence for our comparisons of interest is included in the league table, with * indicating low confidence, † indicating moderate confidence, and ‡ indicating high confidence in the evidence. The full CINeMA assessment is shown in Appendix 9.

| AT |  |  |  |  |  |
| --- | --- | --- | --- | --- | --- |
| 0.12*  (0.03, 0.21) | RT |  |  |  |  |
| 0.01*  (-0.16, 0.18) | -0.11*  (-0.29, 0.07) | AT+RT |  |  |  |
| 0.2*  (0.06, 0.34) | 0.08*  (-0.08, 0.25) | 0.19*  (-0.03, 0.4) | HIIT |  |  |
| 0.08*  (-0.03, 0.19) | -0.04*  (-0.16, 0.08) | 0.07*  (-0.13, 0.26) | -0.12*  (-0.29, 0.06) | TCEs |  |
| -0.4*  (-0.47, -0.33) | -0.52*  (-0.61, -0.44) | -0.42*  (-0.59, -0.24) | -0.61*  (-0.75, -0.45) | -0.48*  (-0.58, -0.38) | C |

**Table S8.3**: league table of 2hPG

The columns represent the comparison of the row exercise modality to the column exercise modality. The rows represent the comparison of the row exercise modality to the column exercise modality. The effect estimates are expressed as mean difference and 95% confidence interval. For example, the mean difference in 2hPG for AT compared to RT is -0.17 (95% confidence interval -0.38 to 0.04). Mean difference <0 favors the exercise modality in the column, and mean difference >0 favors the exercise modality in the row. According to the network meta-analysis (CINeMA) framework, the certainty of the evidence for our comparisons of interest is included in the league table, with * indicating low confidence, † indicating moderate confidence, and ‡ indicating high confidence in the evidence. The full CINeMA assessment is shown in Appendix 9.

| AT |  |  |  |  |  |
| --- | --- | --- | --- | --- | --- |
| -0.17*  (-0.38, 0.04) | RT |  |  |  |  |
| 0.11*  (-0.24, 0.47) | 0.28*  (-0.11, 0.67) | AT+RT |  |  |  |
| 0.28*  (-0.07, 0.62) | 0.44*  (0.06, 0.83) | 0.16*  (-0.32, 0.65) | HIIT |  |  |
| 0.25*  (-0.01, 0.53) | 0.42*  (0.11, 0.74) | 0.14*  (-0.29, 0.58) | -0.02*  (-0.44, 0.41) | TCEs |  |
| -1.01†  (-1.18, -0.85) | -0.85*  (-1.04, -0.65) | -1.13†  (-1.49, -0.76) | -1.29†  (-1.65, -0.93) | -1.27*  (-1.54, -1.01) | C |

**Table S8.4**: league table of TC

The columns represent the comparison of the row exercise modality to the column exercise modality. The rows represent the comparison of the row exercise modality to the column exercise modality. The effect estimates are expressed as mean difference and 95% confidence interval. For example, the mean difference in TC for AT compared to RT is -0.03 (95% confidence interval -0.14 to 0.8). Mean difference <0 favors the exercise modality in the column, and mean difference >0 favors the exercise modality in the row. According to the network meta-analysis (CINeMA) framework, the certainty of the evidence for our comparisons of interest is included in the league table, with * indicating low confidence, † indicating moderate confidence, and ‡ indicating high confidence in the evidence. The full CINeMA assessment is shown in Appendix 9.

| AT |  |  |  |  |  |
| --- | --- | --- | --- | --- | --- |
| -0.03*  (-0.14, 0.08) | RT |  |  |  |  |
| 0.19†  (0.04, 0.34) | 0.22†  (0.07, 0.38) | AT+RT |  |  |  |
| 0.03*  (-0.14, 0.19) | 0.06†  (-0.13, 0.24) | -0.17†  (-0.38, 0.04) | HIIT |  |  |
| -0.08*  (-0.3, 0.14) | -0.05*  (-0.29, 0.19) | -0.27†  (-0.53, -0.01) | -0.11†  (-0.38, 0.16) | TCEs |  |
| -0.26†  (-0.35, -0.18) | -0.23†  (-0.34, -0.13) | -0.46†  (-0.61, -0.32) | -0.29*  (-0.46, -0.13) | -0.18*  (-0.41, 0.04) | C |

**Table S8.5**: league table of TG

The columns represent the comparison of the row exercise modality to the column exercise modality. The rows represent the comparison of the row exercise modality to the column exercise modality. The effect estimates are expressed as mean difference and 95% confidence interval. For example, the mean difference in TG for AT compared to RT is 0.02 (95% confidence interval -0.06 to 0.1). Mean difference <0 favors the exercise modality in the column, and mean difference >0 favors the exercise modality in the row. According to the network meta-analysis (CINeMA) framework, the certainty of the evidence for our comparisons of interest is included in the league table, with * indicating low confidence, † indicating moderate confidence, and ‡ indicating high confidence in the evidence. The full CINeMA assessment is shown in Appendix 9.

| AT |  |  |  |  |  |
| --- | --- | --- | --- | --- | --- |
| 0.02*  (-0.06, 0.1) | RT |  |  |  |  |
| 0.36†  (0.23, 0.5) | 0.33*  (0.19, 0.48) | AT+RT |  |  |  |
| 0.08*  (-0.06, 0.21) | 0.06*  (-0.1, 0.2) | -0.28*  (-0.46, -0.1) | HIIT |  |  |
| 0.01*  (-0.14, 0.16) | -0.01*  (-0.18, 0.15) | -0.35†  (-0.54, -0.16) | -0.07*  (-0.26, 0.12) | TCEs |  |
| -0.2†  (-0.27, -0.14) | -0.22†  (-0.31, -0.14) | -0.55†  (-0.69, -0.42) | -0.28†  (-0.41, -0.14) | -0.21*  (-0.35, -0.06) | C |

**Table S8.6**: league table of HDL

The columns represent the comparison of the row exercise modality to the column exercise modality. The rows represent the comparison of the row exercise modality to the column exercise modality. The effect estimates are expressed as mean difference and 95% confidence interval. For example, the mean difference in HDL for AT compared to RT is 0 (95% confidence interval -0.7 to 0.06). Mean difference <0 favors the exercise modality in the column, and mean difference >0 favors the exercise modality in the row. According to the network meta-analysis (CINeMA) framework, the certainty of the evidence for our comparisons of interest is included in the league table, with * indicating low confidence, † indicating moderate confidence, and ‡ indicating high confidence in the evidence. The full CINeMA assessment is shown in Appendix 9.

| AT |  |  |  |  |  |
| --- | --- | --- | --- | --- | --- |
| 0*  (-0.07, 0.06) | RT |  |  |  |  |
| -0.04*  (-0.14, 0.07) | -0.03*  (-0.14, 0.07) | AT+RT |  |  |  |
| -0.11*  (-0.27, 0.05) | -0.11*  (-0.27, 0.06) | -0.07*  (-0.26, 0.11) | HIIT |  |  |
| -0.04*  (-0.16, 0.07) | -0.04*  (-0.16, 0.09) | 0*  (-0.15, 0.15) | 0.07*  (-0.12, 0.26) | TCEs |  |
| 0.09*  (0.03, 0.14) | 0.09*  (0.03, 0.16) | 0.13*  (0.02, 0.23) | 0.2†  (0.04, 0.36) | 0.13*  (0.01, 0.24) | C |

**Table S8.7**: league table of LDL

The columns represent the comparison of the row exercise modality to the column exercise modality. The rows represent the comparison of the row exercise modality to the column exercise modality. The effect estimates are expressed as mean difference and 95% confidence interval. For example, the mean difference in LDL for AT compared to RT is -0.01 (95% confidence interval -0.12 to 0.09). Mean difference <0 favors the exercise modality in the column, and mean difference >0 favors the exercise modality in the row. According to the network meta-analysis (CINeMA) framework, the certainty of the evidence for our comparisons of interest is included in the league table, with * indicating low confidence, † indicating moderate confidence, and ‡ indicating high confidence in the evidence. The full CINeMA assessment is shown in Appendix 9.

| AT |  |  |  |  |  |
| --- | --- | --- | --- | --- | --- |
| -0.01*  (-0.12, 0.09) | RT |  |  |  |  |
| 0.08*  (-0.1, 0.25) | 0.09*  (-0.09, 0.27) | AT+RT |  |  |  |
| 0.04*  (-0.19, 0.27) | 0.06*  (-0.19, 0.3) | -0.04*  (-0.32, 0.24) | HIIT |  |  |
| -0.05*  (-0.26, 0.15) | -0.04*  (-0.26, 0.17) | -0.13*  (-0.39, 0.12) | -0.09*  (-0.4, 0.21) | TCEs |  |
| -0.27*  (-0.37, -0.19) | -0.26*  (-0.36, -0.17) | -0.35*  (-0.53, -0.18) | -0.31†  (-0.55, -0.08) | -0.22*  (-0.42, -0.02) | C |

**Table S8.8**: league table of BMI

The columns represent the comparison of the row exercise modality to the column exercise modality. The rows represent the comparison of the row exercise modality to the column exercise modality. The effect estimates are expressed as mean difference and 95% confidence interval. For example, the mean difference in BMI for AT compared to RT is -0.02 (95% confidence interval -0.39 to 0.44). Mean difference <0 favors the exercise modality in the column, and mean difference >0 favors the exercise modality in the row. According to the network meta-analysis (CINeMA) framework, the certainty of the evidence for our comparisons of interest is included in the league table, with * indicating low confidence, † indicating moderate confidence, and ‡ indicating high confidence in the evidence. The full CINeMA assessment is shown in Appendix 9.

| AT |  |  |  |  |  |
| --- | --- | --- | --- | --- | --- |
| 0.02*  (-0.39, 0.44) | RT |  |  |  |  |
| 0.08*  (-0.65, 0.81) | 0.05*  (-0.77, 0.88) | AT+RT |  |  |  |
| 0.01*  (-0.58, 0.59) | -0.02*  (-0.72, 0.69) | -0.07*  (-1.01, 0.86) | HIIT |  |  |
| -0.12*  (-1.03, 0.79) | -0.14*  (-1.11, 0.83) | -0.19*  (-1.35, 0.96) | -0.13*  (-1.21, 0.95) | TCEs |  |
| -0.81†  (-1.1, -0.53) | -0.84†  (-1.24, -0.44) | -0.89*  (-1.64, -0.14) | -0.82*  (-1.47, -0.18) | -0.69*  (-1.59, 0.19) | C |

**Table S8.9**: league table of BW

The columns represent the comparison of the row exercise modality to the column exercise modality. The rows represent the comparison of the row exercise modality to the column exercise modality. The effect estimates are expressed as mean difference and 95% confidence interval. For example, the mean difference in BW for AT compared to RT is -1.3 (95% confidence interval -4.3 to 1.62). Mean difference <0 favors the exercise modality in the column, and mean difference >0 favors the exercise modality in the row. According to the network meta-analysis (CINeMA) framework, the certainty of the evidence for our comparisons of interest is included in the league table, with * indicating low confidence, † indicating moderate confidence, and ‡ indicating high confidence in the evidence. The full CINeMA assessment is shown in Appendix 9.

| AT |  |  |  |  |
| --- | --- | --- | --- | --- |
| -1.3*  (-4.3, 1.62) | RT |  |  |  |
| 0.28*  (-2.3, 2.77) | 1.58*  (-2.21, 5.38) | HIIT |  |  |
| 0.83*  (-2.17, 3.83) | 2.13*  (-1.9, 6.23) | 0.56*  (-3.3, 4.49) | TCEs |  |
| -2.61*  (-3.94, -1.28) | -1.31*  (-4.22, 1.67) | -2.89*  (-5.63, -0.06) | -3.44*  (-6.35, -0.51) | C |

**Table S8.10**: league table of WC

The columns represent the comparison of the row exercise modality to the column exercise modality. The rows represent the comparison of the row exercise modality to the column exercise modality. The effect estimates are expressed as mean difference and 95% confidence interval. For example, the mean difference in BW for AT compared to RT is -1.3 (95% confidence interval -4.3 to 1.62). Mean difference <0 favors the exercise modality in the column, and mean difference >0 favors the exercise modality in the row. According to the network meta-analysis (CINeMA) framework, the certainty of the evidence for our comparisons of interest is included in the league table, with * indicating low confidence, † indicating moderate confidence, and ‡ indicating high confidence in the evidence. The full CINeMA assessment is shown in Appendix 9.

| AT |  |  |  |  |
| --- | --- | --- | --- | --- |
| -2.1*  (-5.06, 0.98) | RT |  |  |  |
| 0.33*  (-4, 4.63) | 2.43*  (-2.66, 7.38) | HIIT |  |  |
| 1.32*  (-1.91, 4.47) | 3.42*  (-0.83, 7.44) | 0.98*  (-4.3, 6.25) | TCEs |  |
| -2.74*  (-4.43, -1.06) | -0.64*  (-3.49, 2.11) | -3.08*  (-7.5, 1.39) | -4.06*  (-7.18, -0.87) | C |

# Appendix 9: CINeMA Assessment

We use the CINeMA framework to assess evidence certainty, evaluating each network estimate based on the following criteria:

- **Within study bias:** We classified the overall risk of bias for each study as low risk of bias, the risk of bias as moderate when none of the four assessed risk of bias items were rated as high risk, and the risk of bias as high when one or both items were rated as high risk. See **Appendix 4** for the bias assessment.
- **Reporting bias:** We judged it visually by a funnel plot **(Appendix 9)**.
- **Indirectness:** Transferability assumptions were assessed by reporting baseline glycated hemoglobin levels in the included study population and by comparing age and BMI at baseline concordance between groups.
- **Imprecision:** We use the CINeMA website to grade the accuracy of each comparison.
- **Heterogeneity:** We assessed the degree of worry by comparing clinical reasoning based on 95% confidence intervals (CIs) while applying the same clinical reasoning framework as for inaccuracy. In particular, we judged the consistency of our findings based on the confidence and prediction intervals associated with clinically important effect sizes. And we used the same thresholds of clinical significance as described above and followed the recommendations automatically provided by CINeMA (https://cinema.ispm.unibe.ch/).
- **Inconsistency:** For inconsistency, we looked at the results for node splitting **(Appendix 5)** and we saw major problems when p<0.10, but otherwise no problems.

**Figure S9.1:** Risk of bias contribution by intervention group in HbA1c


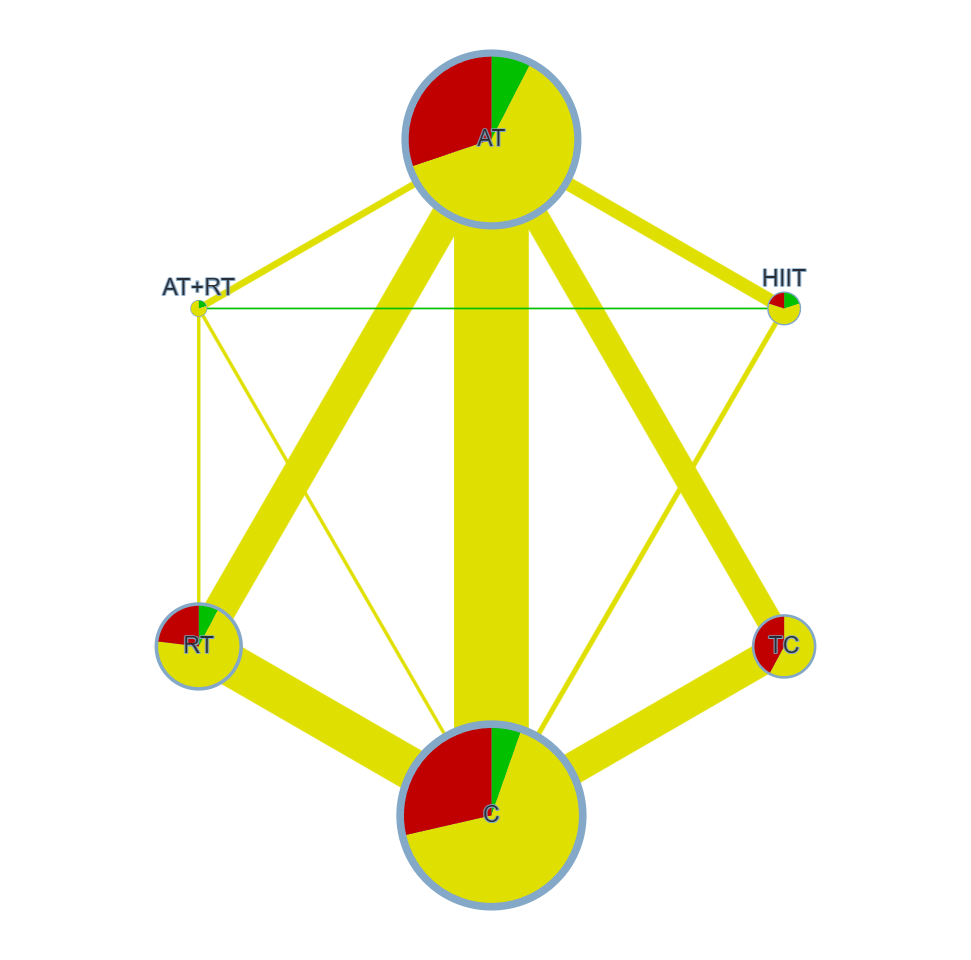


**Figure S9.2:** Overall risk of bias by treatment comparison in HbA1c


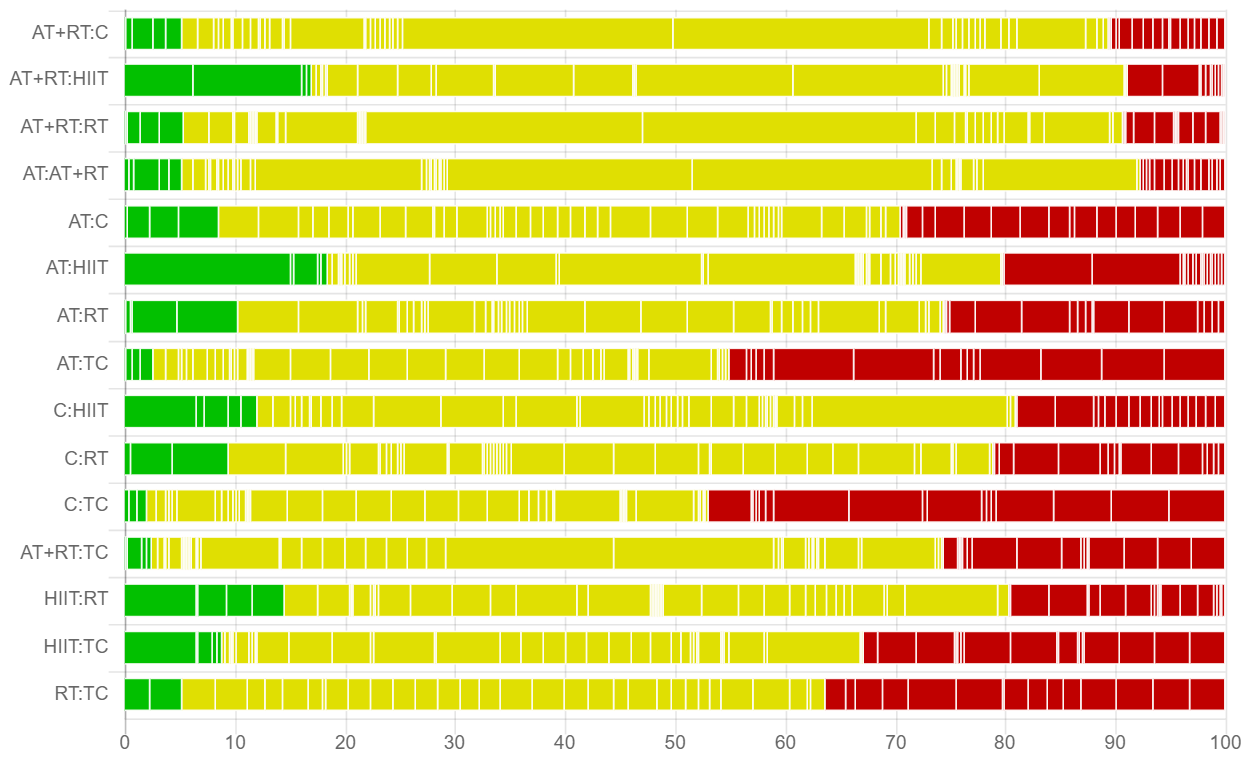


**Figure S9.3**: Risk of bias contribution by intervention group in FBG


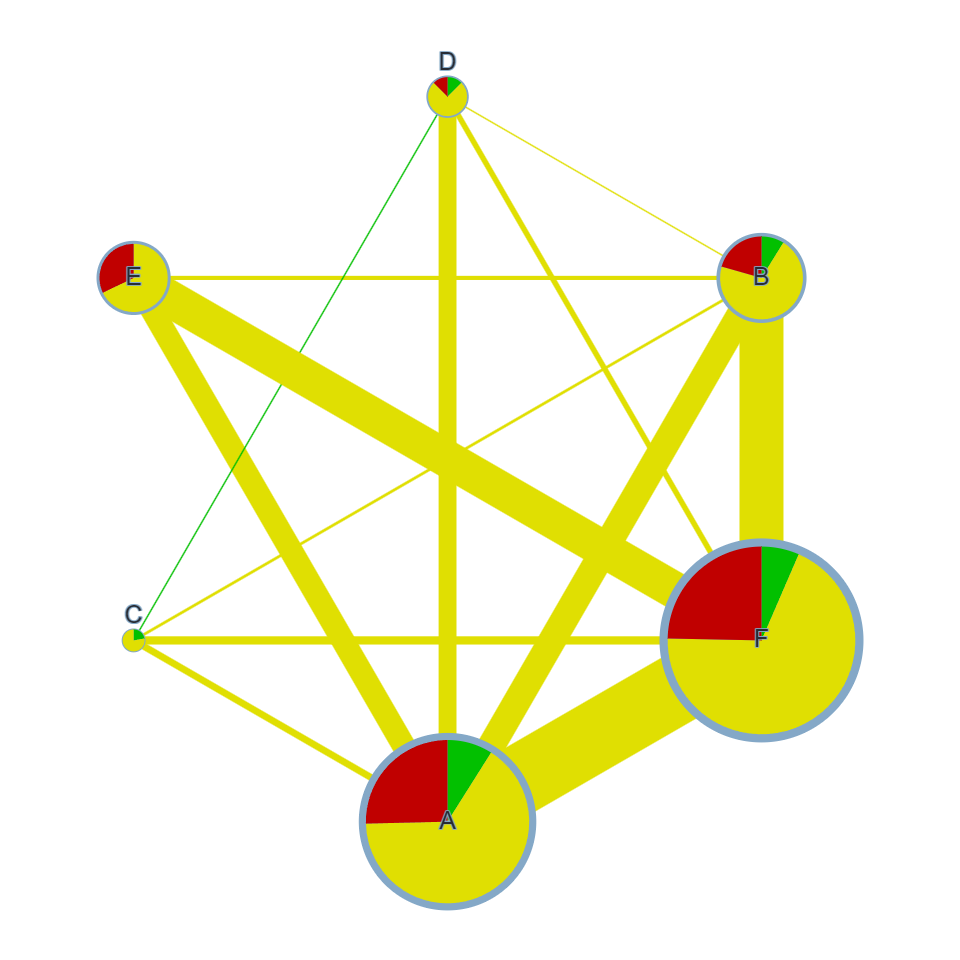


**Figure S9.4:** Overall risk of bias by treatment comparison in FBG


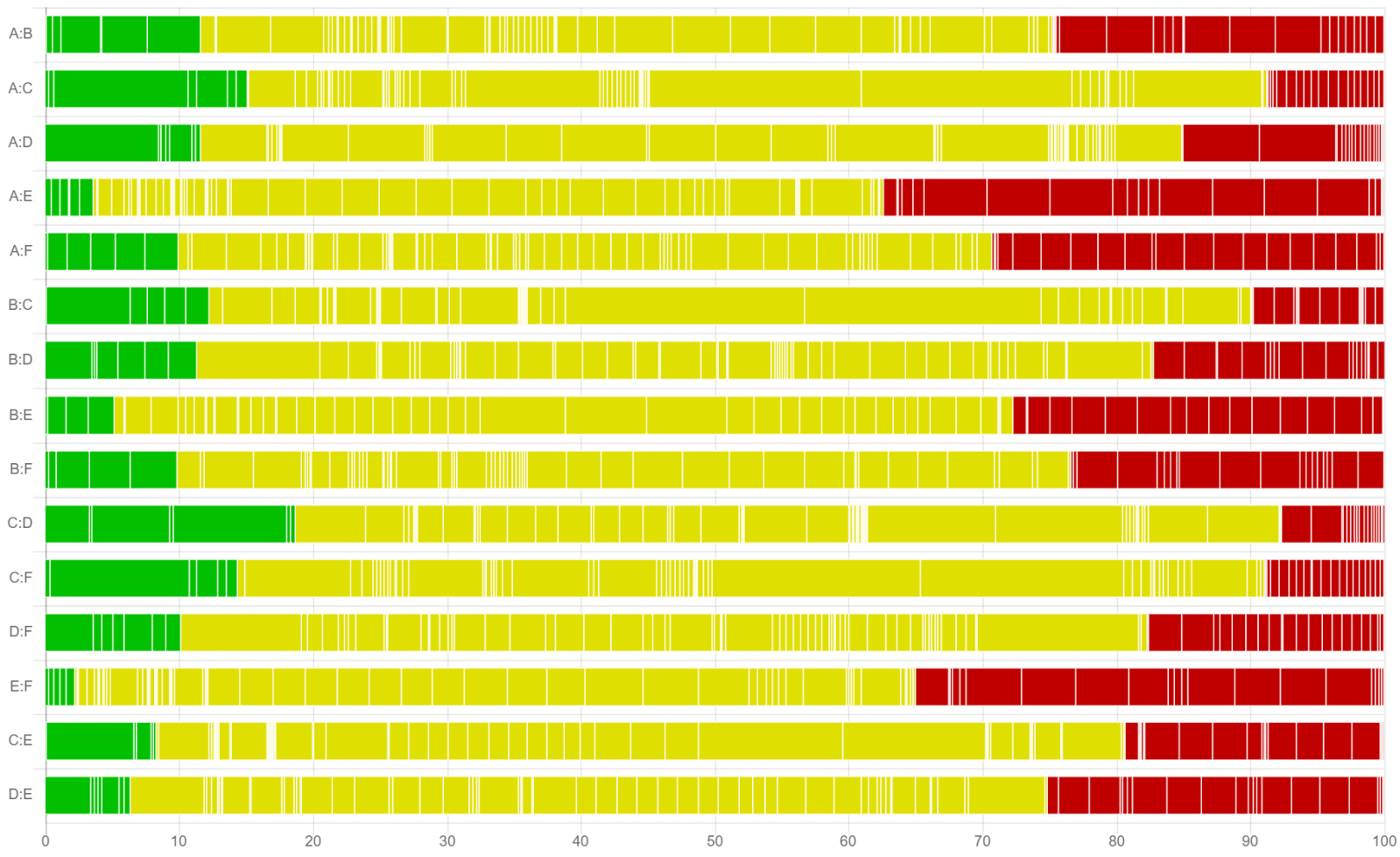


**Figure S9.5**: Risk of bias contribution by intervention group in 2hPG


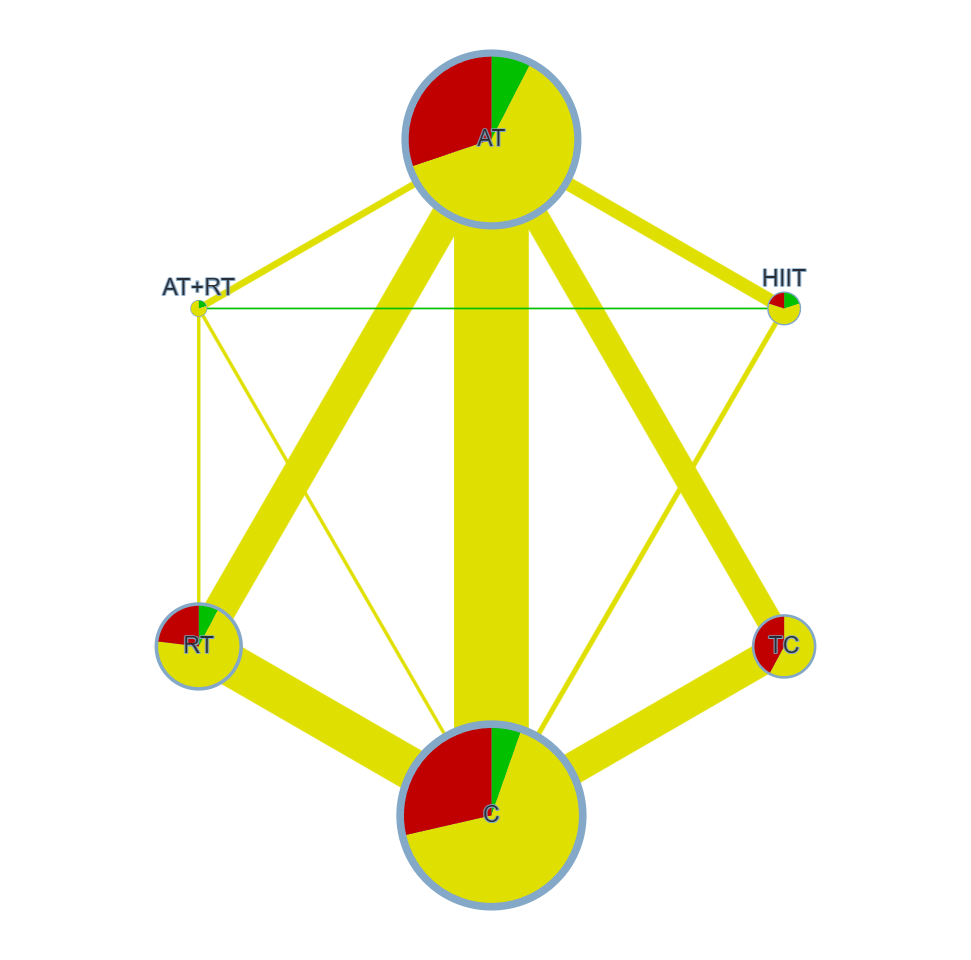


**Figure S9.6:** Overall risk of bias by treatment comparison in 2hPG


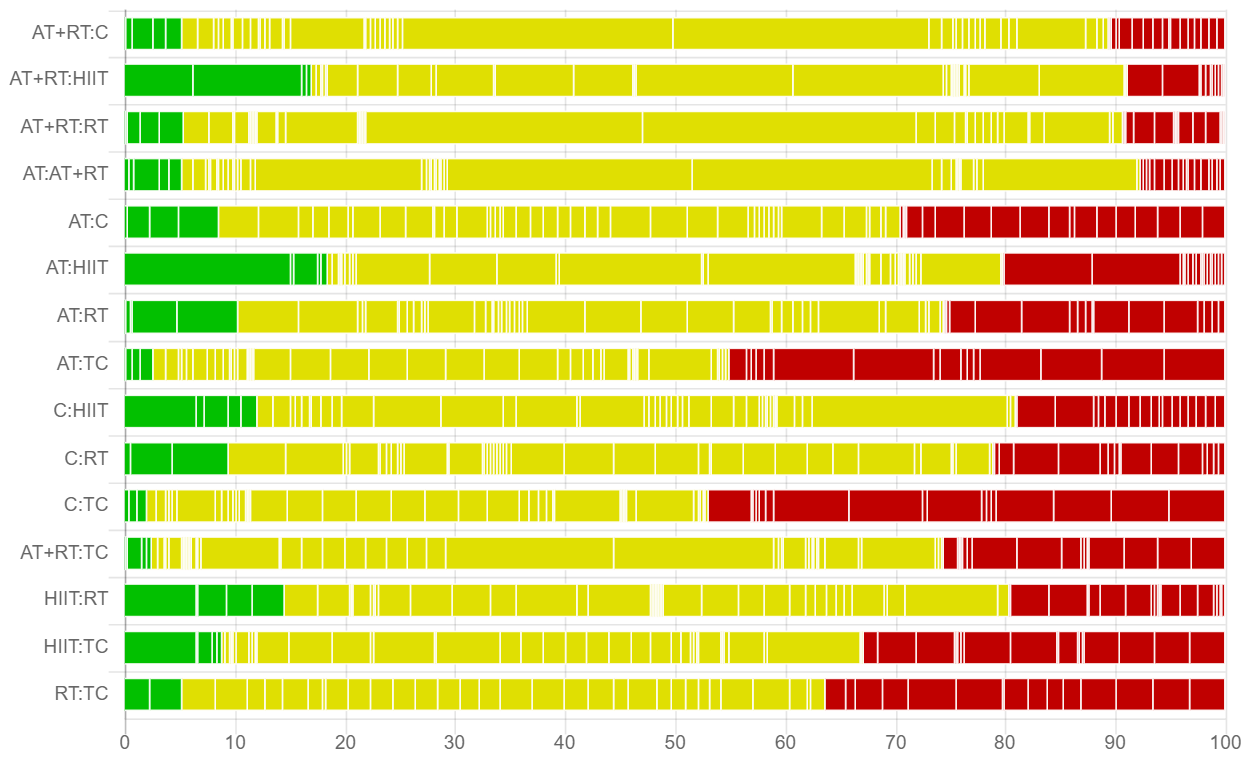


**Figure S9.7:** Risk of bias contribution by intervention group in TC


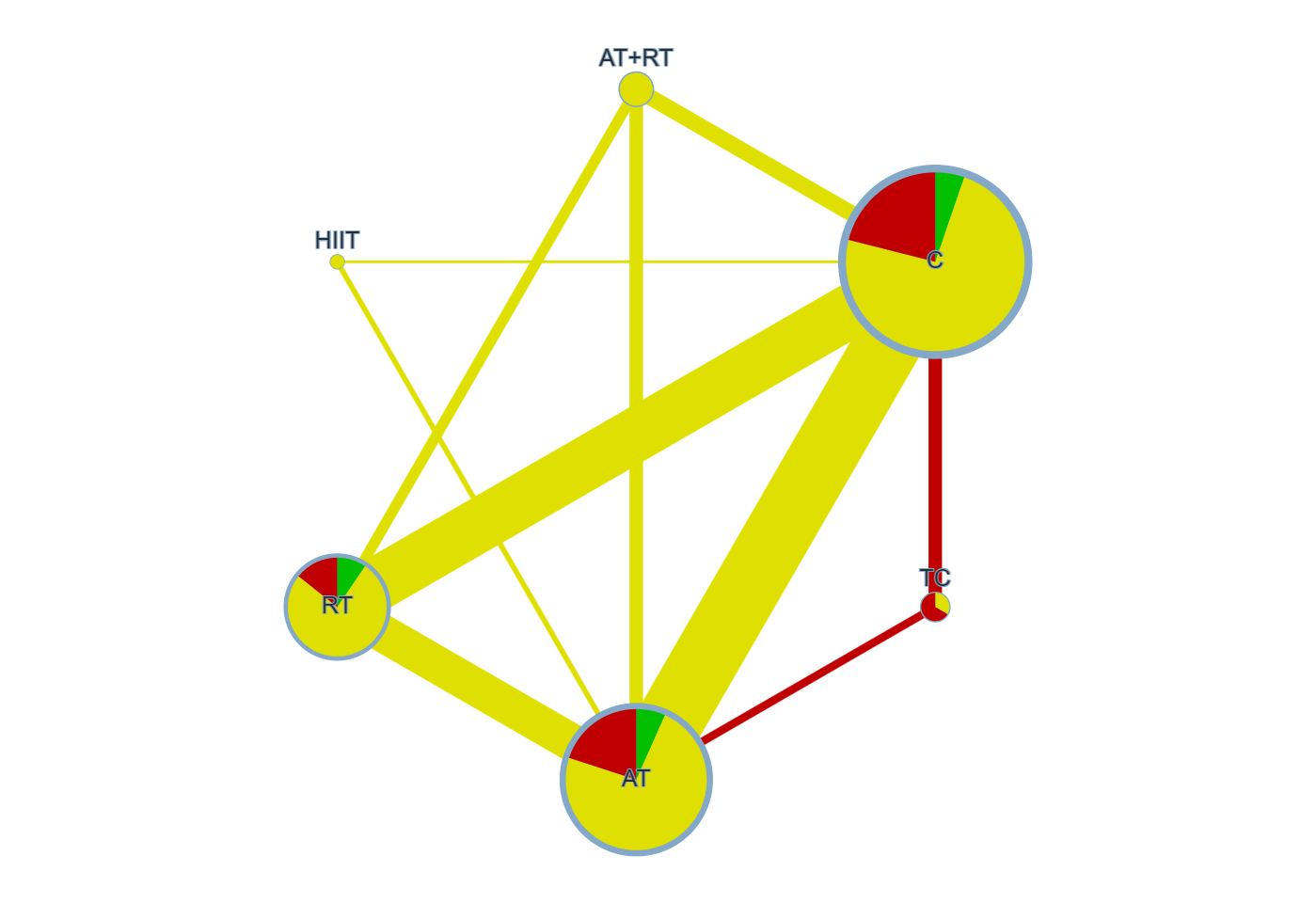


**Figure S9.8:** Overall risk of bias by treatment comparison in TC


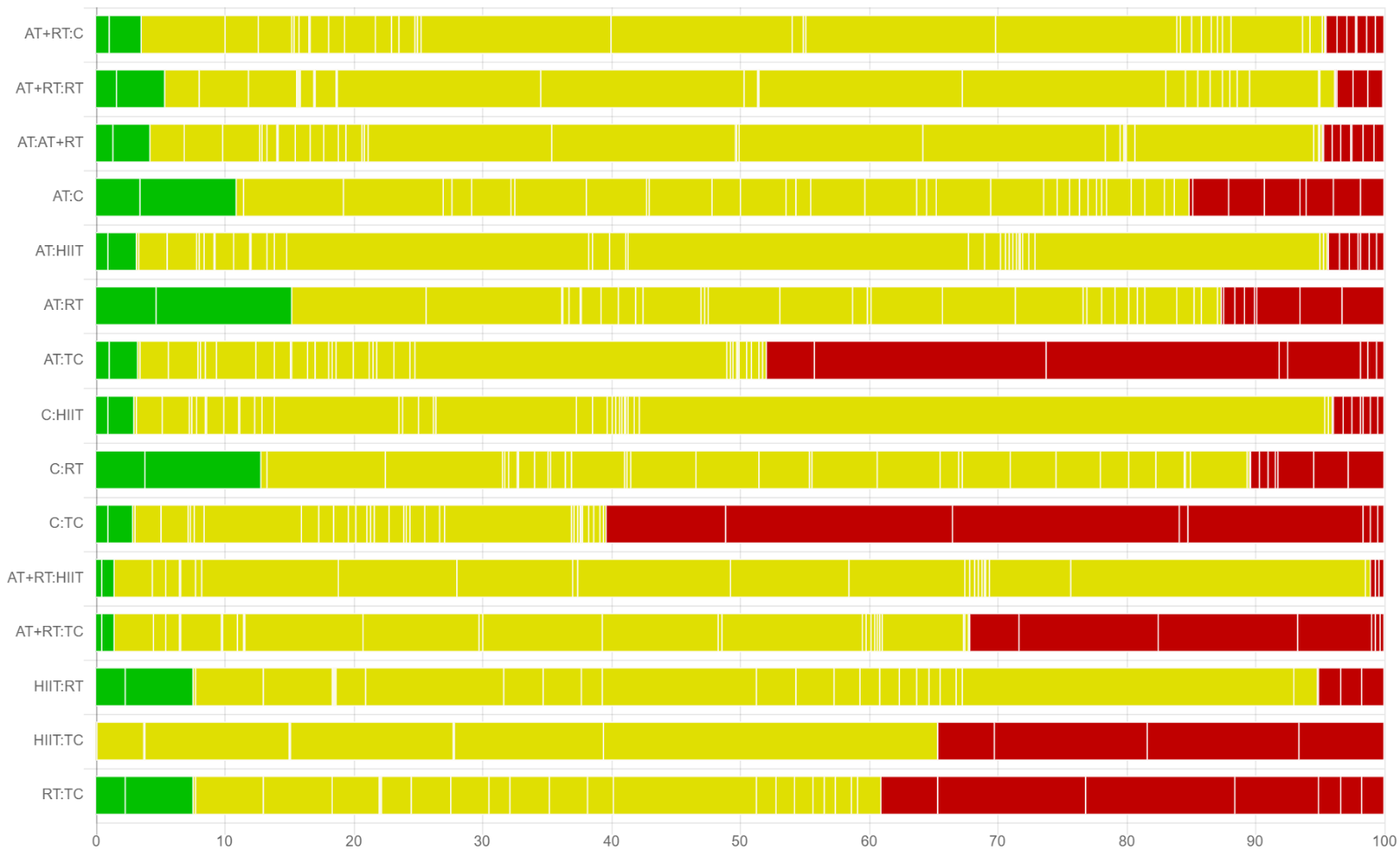


**Figure S9.9:** Risk of bias contribution by intervention group in TG


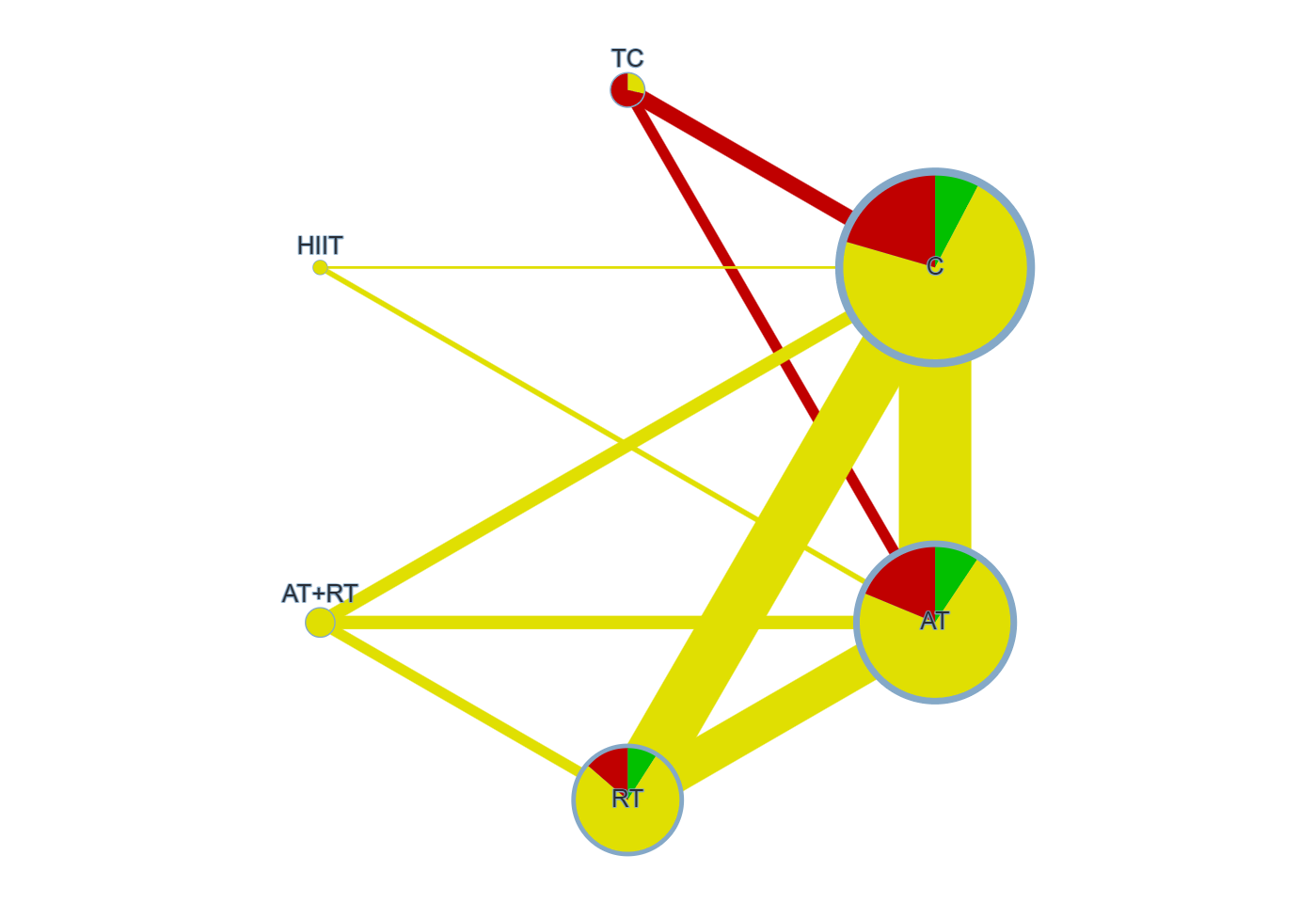


**Figure S9.10:** Overall risk of bias by treatment comparison in TG


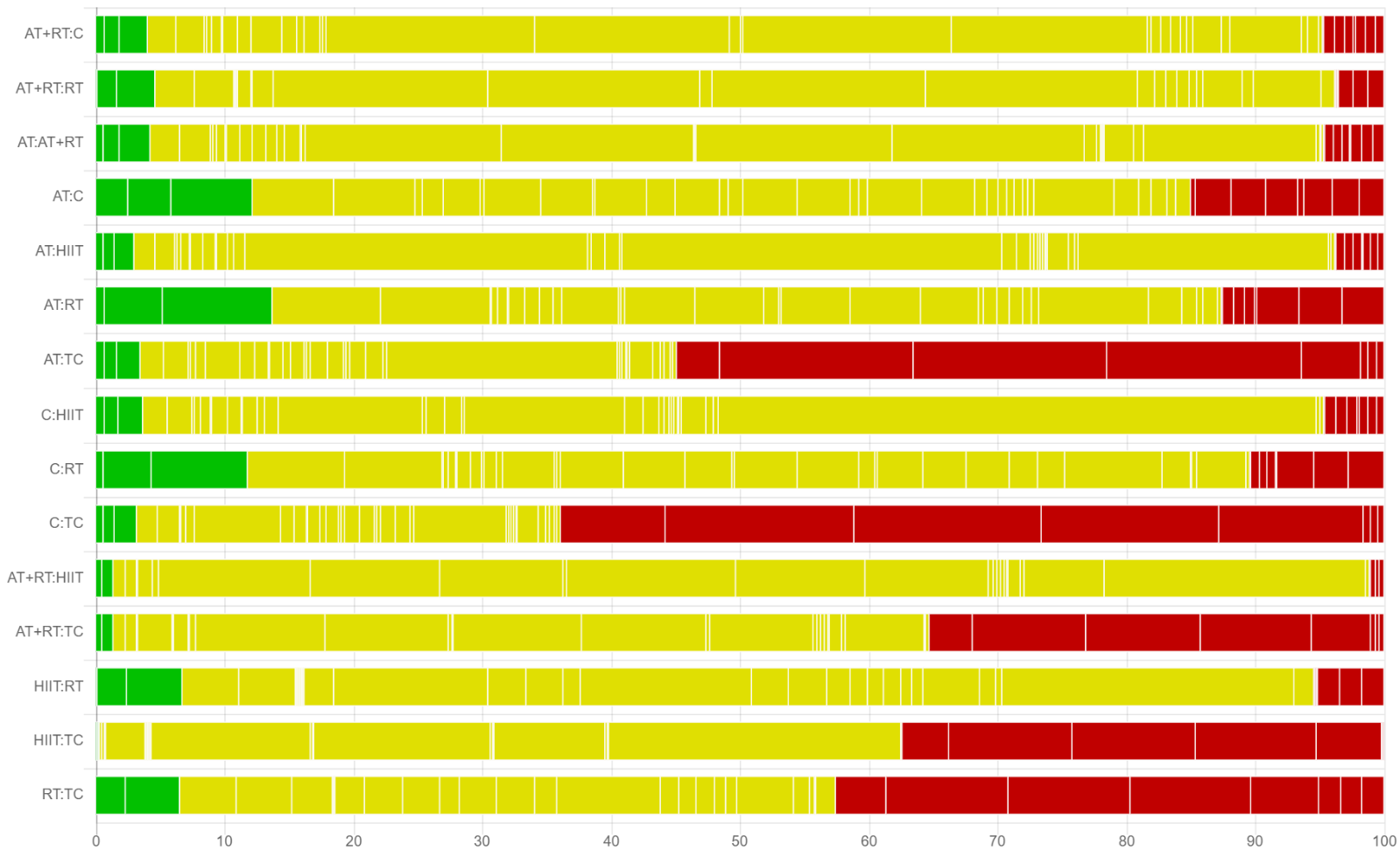


**Figure S9.11:** Risk of bias contribution by intervention group in HDL


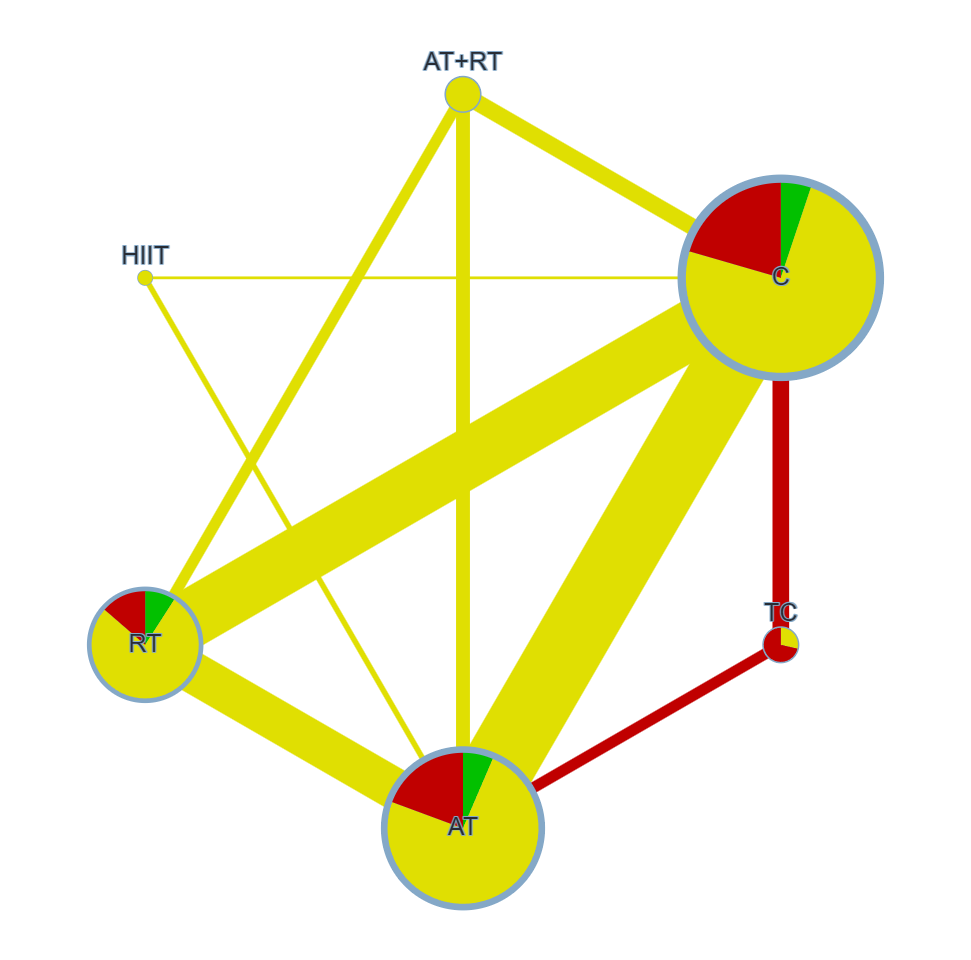


**Figure S9.12:** Overall risk of bias by treatment comparison in HDL


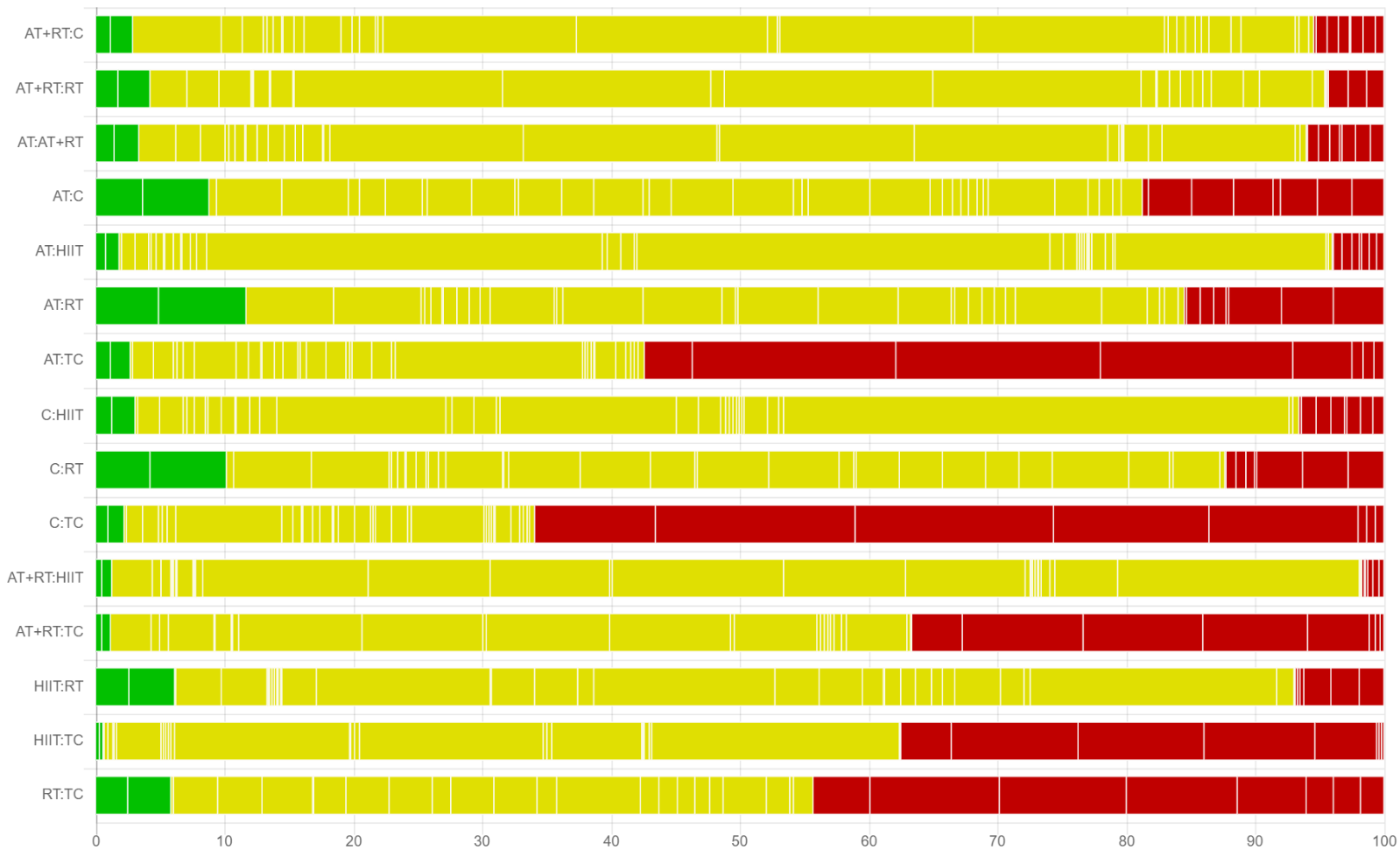


**Figure S9.13:** Risk of bias contribution by intervention group in LDL


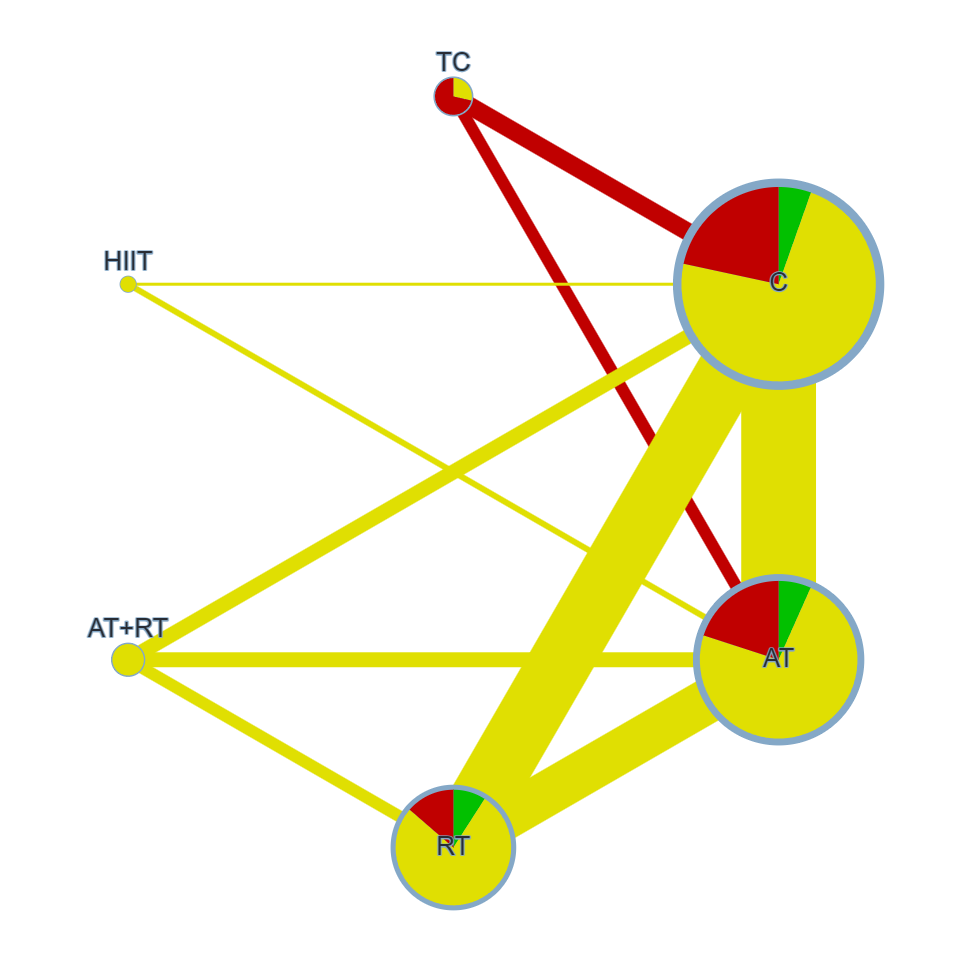


**Figure S9.14:** Overall risk of bias by treatment comparison in LDL


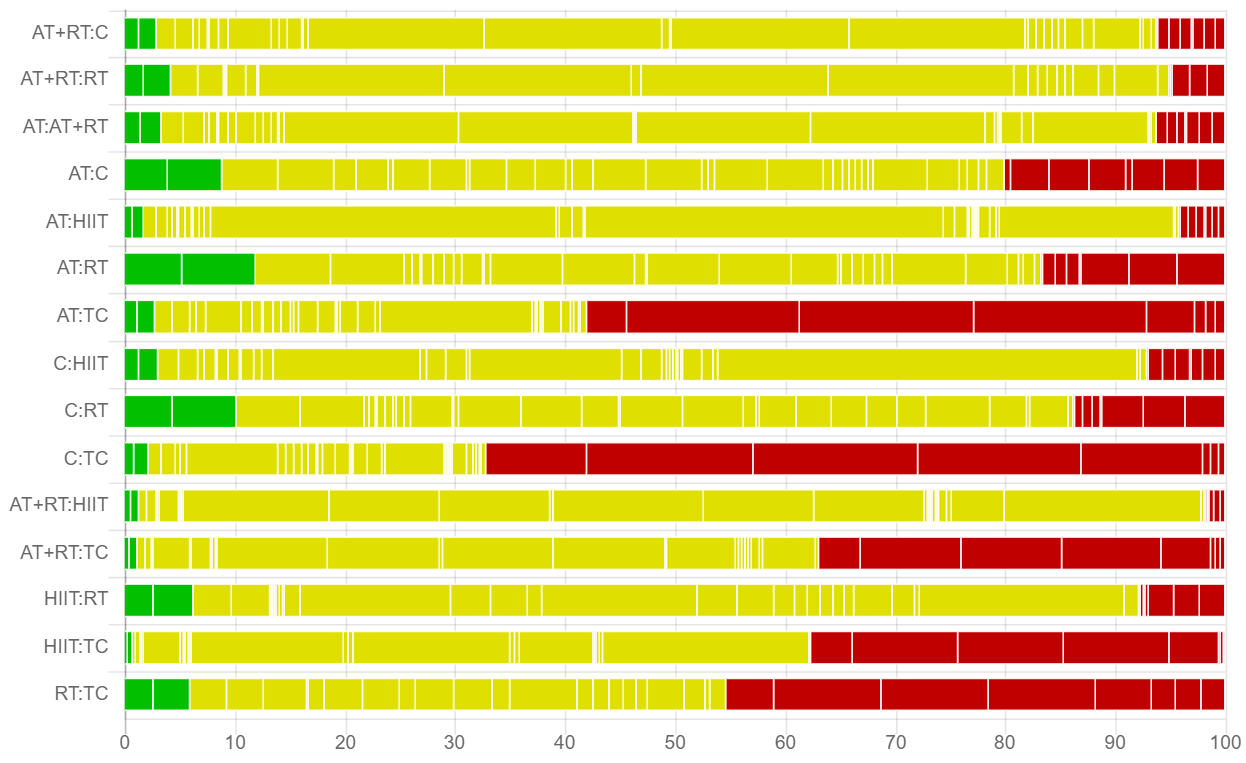


**Figure S9.15:** Risk of bias contribution by intervention group in BMI


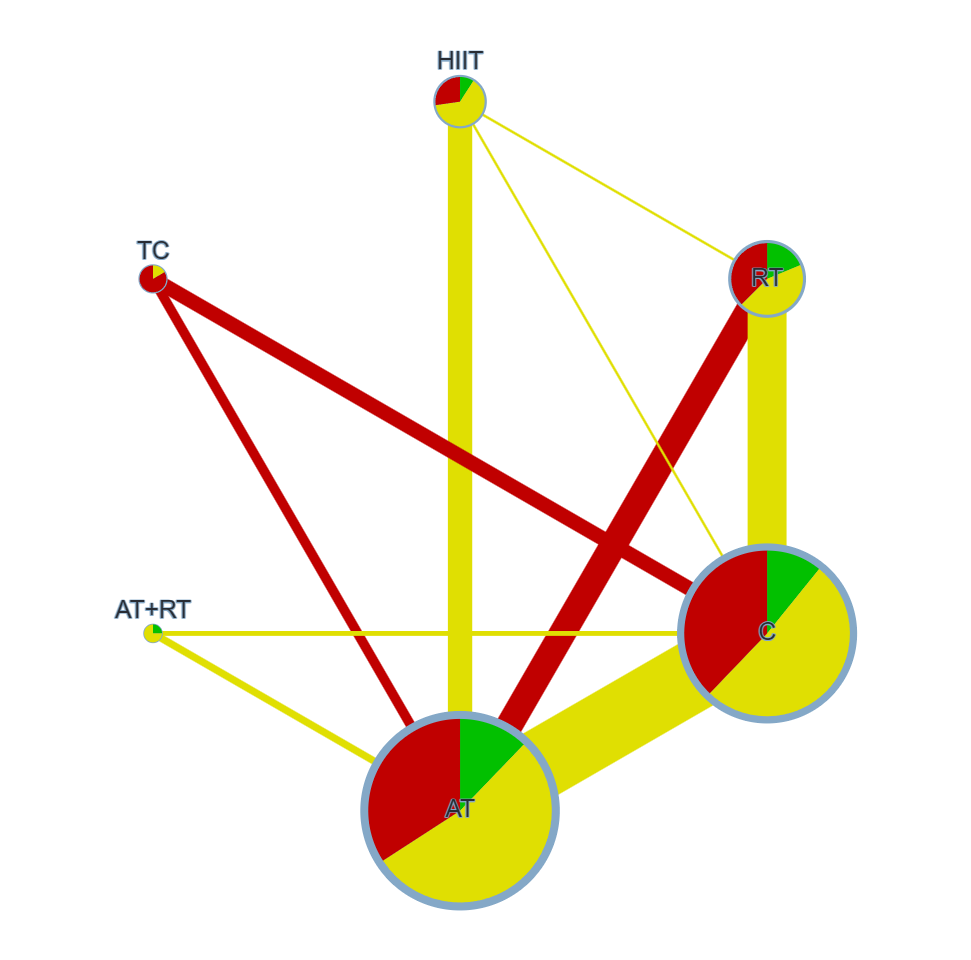


**Figure S9.16:** Overall risk of bias by treatment comparison in BMI


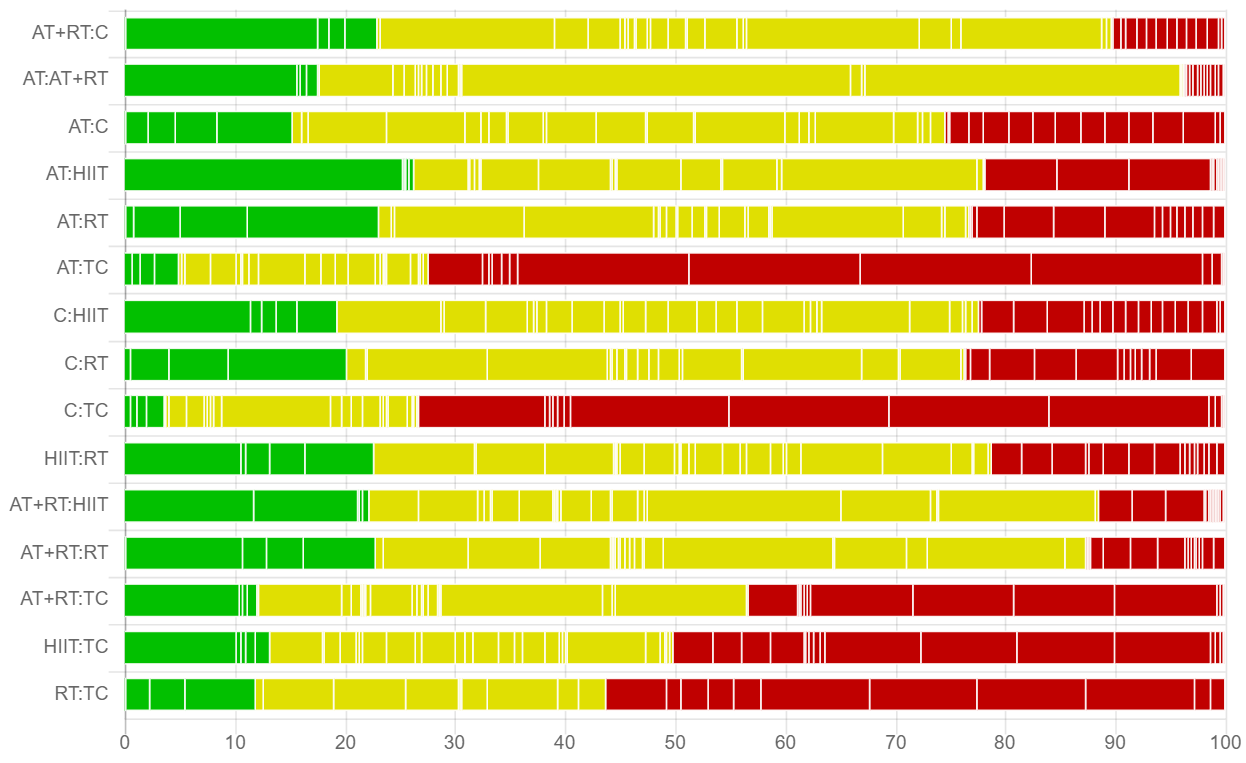


**Figure S9.17:** Risk of bias contribution by intervention group in BW


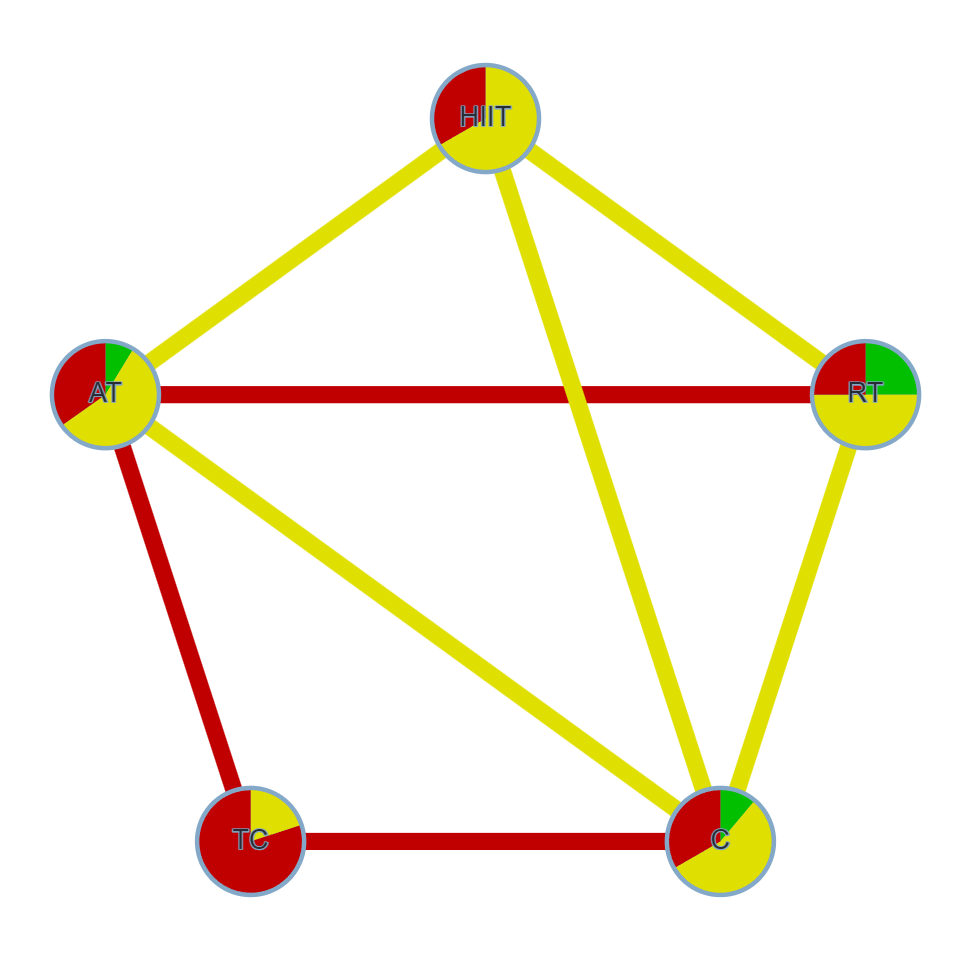


**Figure S9.18:** Overall risk of bias by treatment comparison in BW


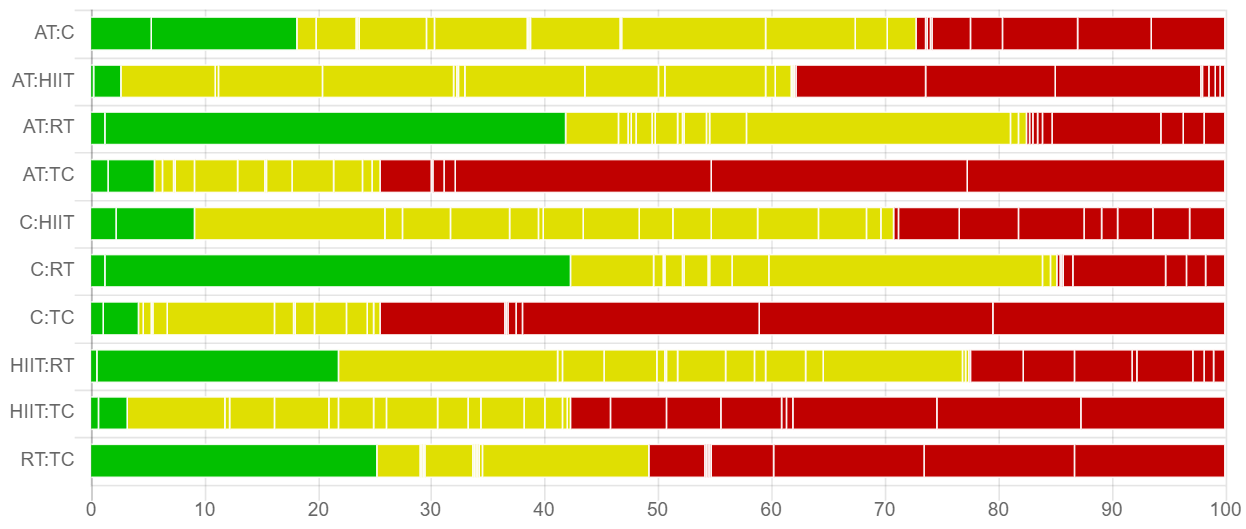


**Figure S9.19:** Risk of bias contribution by intervention group in WC


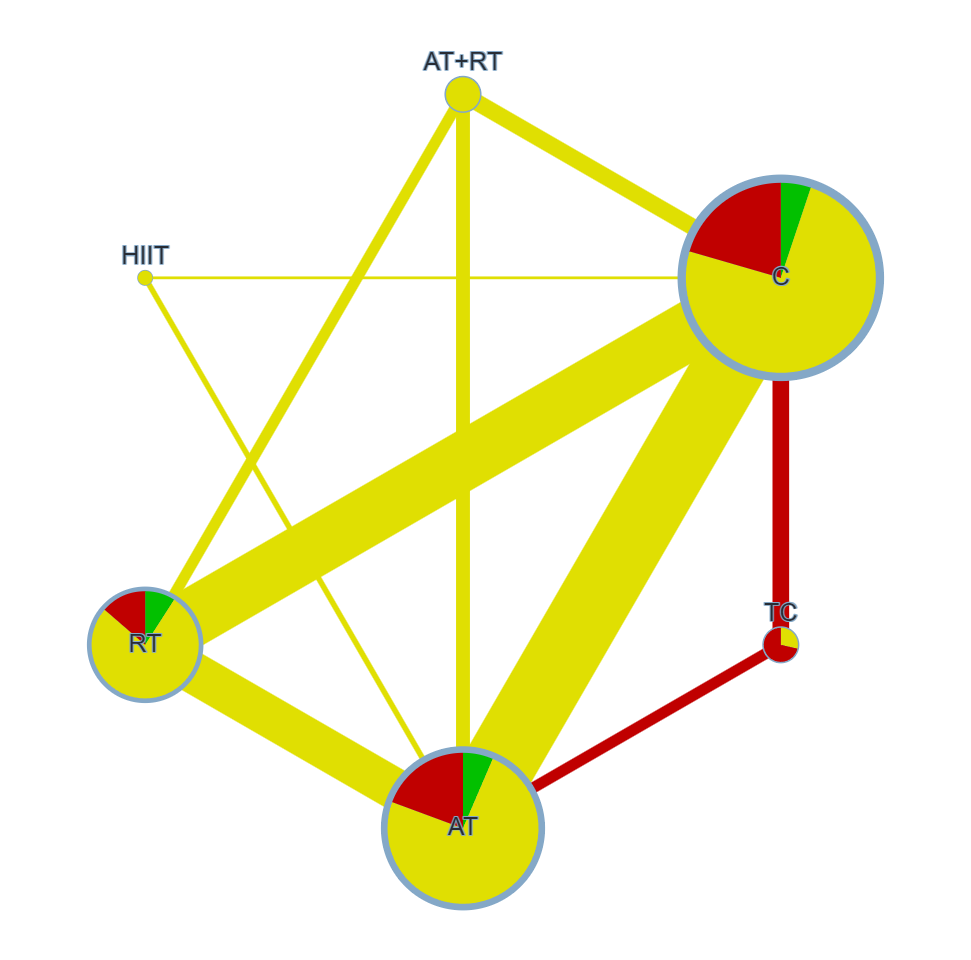


**Figure S9.20:** Overall risk of bias by treatment comparison in WC
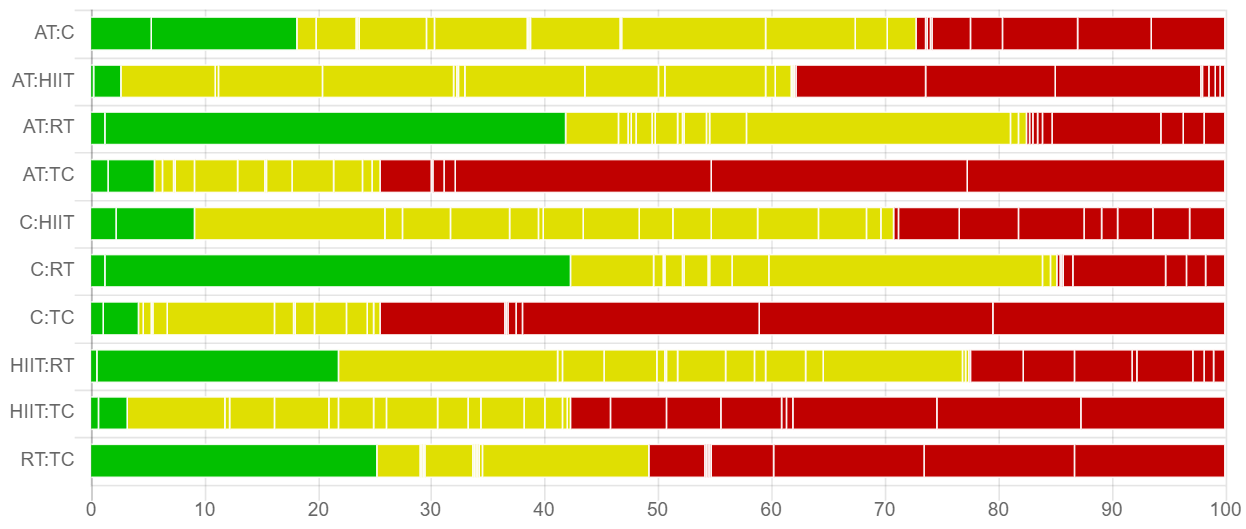


**Table S9.1:** Transitivity (Indirectness) Assessment

|  | Baseline variable (Mean ± SD) | |
| --- | --- | --- |
| Intervention | Age(year) | HbA1c（%） |
| AT | 53.62±7.26 | 5.95±0.48 |
| RT | 58.15±6.42 | 5.90±0.42 |
| AT+RT | 53.14±6.69 | 5.96±0.30 |
| HIIT | 50.83±9.4 | 5.99±0.37 |
| TCEs | 58.53±3.81 | 6.29±0.45 |
| C | 56.40±6.22 | 5.90±0.49 |

**Table S9.1:** CINeMA Results of HbA1c

| Comparison | Within-study  bias | Reporting bias | Indirectness | Imprecision | Heterogeneity | Incoherence | Confidence rating |
| --- | --- | --- | --- | --- | --- | --- | --- |
| AT:AT+RT | Some concerns | Low risk | No concerns | Major concerns | No concerns | No concerns | Very low |
| AT:C | Some concerns | Low risk | No concerns | No concerns | No concerns | No concerns | Moderate |
| AT:HIIT | Some concerns | Low risk | No concerns | No concerns | Major concerns | Major concerns | Very low |
| AT:RT | Some concerns | Low risk | No concerns | Major concerns | No concerns | No concerns | Very low |
| AT:TCEs | Some concerns | Low risk | No concerns | Major concerns | No concerns | No concerns | Very low |
| AT+RT:C | Some concerns | Low risk | No concerns | No concerns | No concerns | No concerns | Moderate |
| AT+RT:HIIT | Some concerns | Low risk | No concerns | Major concerns | No concerns | No concerns | Very low |
| AT+RT:RT | Some concerns | Low risk | No concerns | Major concerns | No concerns | No concerns | Very low |
| C:HIIT | Some concerns | Low risk | No concerns | No concerns | No concerns | No concerns | Moderate |
| C:RT | Some concerns | Low risk | No concerns | No concerns | No concerns | No concerns | Moderate |
| C:TCEs | Some concerns | Low risk | No concerns | No concerns | Major concerns | No concerns | Low |
| AT+RT:TCEs | Some concerns | Low risk | No concerns | No concerns | Major concerns | No concerns | Low |
| HIIT:RT | Some concerns | Low risk | No concerns | No concerns | Major concerns | No concerns | Low |
| HIIT:TCEs | Some concerns | Low risk | No concerns | No concerns | Major concerns | Major concerns | Low |
| RT:TCEs | Some concerns | Low risk | No concerns | Major concerns | No concerns | No concerns | Very low |

**Table S9.2:** CINeMA Results of FBG

| Comparison | Within-study  bias | Reporting bias | Indirectness | Imprecision | Heterogeneity | Incoherence | Confidence rating |
| --- | --- | --- | --- | --- | --- | --- | --- |
| AT:RT | Some concerns | Low risk | No concerns | No concerns | Major concerns | No concerns | Low |
| AT:AT+RT | Some concerns | Low risk | No concerns | Major concerns | No concerns | No concerns | Very low |
| AT:HIIT | Some concerns | Low risk | No concerns | Major concerns | No concerns | Major concerns | Very low |
| AT:TCEs | Some concerns | Low risk | No concerns | No concerns | Major concerns | No concerns | Low |
| AT:C | Some concerns | Low risk | No concerns | No concerns | Major concerns | No concerns | Low |
| RT:AT+RT | Some concerns | Low risk | No concerns | Major concerns | No concerns | No concerns | Very low |
| RT:HIIT | Some concerns | Low risk | No concerns | Major concerns | No concerns | No concerns | Very low |
| RT:TCEs | Some concerns | Low risk | No concerns | Major concerns | No concerns | No concerns | Low |
| RT:C | Some concerns | Low risk | No concerns | No concerns | Major concerns | No concerns | Low |
| AT+RT:HIIT | Some concerns | Low risk | No concerns | Major concerns | No concerns | No concerns | Very low |
| AT+RT:C | Some concerns | Low risk | No concerns | No concerns | Major concerns | No concerns | Low |
| HIIT:C | Some concerns | Low risk | No concerns | No concerns | Major concerns | Major concerns | Very low |
| TCEs:C | Some concerns | Low risk | No concerns | No concerns | Major concerns | No concerns | Low |
| AT+RT:TCEs | Some concerns | Low risk | No concerns | Major concerns | No concerns | No concerns | Very low |
| HIIT:TCEs | Some concerns | Low risk | No concerns | Major concerns | No concerns | No concerns | Very low |

**Table S9.3:** CINeMA Results of 2hPG

| Comparison | Within-study bias | Reporting bias | Indirectness | Imprecision | Heterogeneity | Incoherence | Confidence rating |
| --- | --- | --- | --- | --- | --- | --- | --- |
| AT:AT+RT | Some concerns | Low risk | No concerns | Major concerns | No concerns | No concerns | Very low |
| AT:C | Some concerns | Low risk | No concerns | No concerns | No concerns | No concerns | Moderate |
| AT:HIIT | Some concerns | Low risk | No concerns | No concerns | Major concerns | Major concerns | Very low |
| AT:RT | Some concerns | Low risk | No concerns | Major concerns | No concerns | No concerns | Low |
| AT:TCEs | Some concerns | Low risk | No concerns | Major concerns | No concerns | No concerns | Low |
| AT+RT:C | Some concerns | Low risk | No concerns | No concerns | No concerns | No concerns | Moderate |
| AT+RT:RT | Some concerns | Low risk | No concerns | Major concerns | No concerns | No concerns | Low |
| C:HIIT | Some concerns | Low risk | No concerns | No concerns | No concerns | No concerns | Moderate |
| C:RT | Some concerns | Low risk | No concerns | No concerns | Major concerns | No concerns | Low |
| C:TCEs | Some concerns | Low risk | No concerns | No concerns | No concerns | No concerns | Low |
| AT+RT:HIIT | Some concerns | Low risk | No concerns | Major concerns | No concerns | No concerns | Very low |
| AT+RT:TCEs | Some concerns | Low risk | No concerns | Major concerns | No concerns | No concerns | Very low |
| HIIT:RT | Some concerns | Low risk | No concerns | No concerns | Major concerns | No concerns | Low |
| HIIT:TCEs | Some concerns | Low risk | No concerns | Major concerns | No concerns | No concerns | Very low |
| RT:TCEs | Some concerns | Low risk | No concerns | Major concerns | No concerns | No concerns | Very low |

**Table S9.4:** CINeMA Results of TC

| Comparison | Within-study bias | Reporting bias | Indirectness | Imprecision | Heterogeneity | Incoherence | Confidence rating |
| --- | --- | --- | --- | --- | --- | --- | --- |
| AT:AT+RT | Some concerns | Low risk | No concerns | No concerns | No concerns | No concerns | Moderate |
| AT:C | Some concerns | Low risk | No concerns | No concerns | No concerns | No concerns | Moderate |
| AT:HIIT | Some concerns | Low risk | No concerns | No concerns | No concerns | No concerns | Moderate |
| AT:RT | Some concerns | Low risk | No concerns | Major concerns | No concerns | No concerns | Very low |
| AT:TCEs | Some concerns | Low risk | No concerns | Major concerns | No concerns | No concerns | Very low |
| AT+RT:C | Some concerns | Low risk | No concerns | No concerns | No concerns | No concerns | Moderate |
| AT+RT:RT | Some concerns | Low risk | No concerns | No concerns | No concerns | No concerns | Moderate |
| C:HIIT | Some concerns | Low risk | No concerns | No concerns | No concerns | No concerns | Moderate |
| C:RT | Some concerns | Low risk | No concerns | No concerns | No concerns | No concerns | Moderate |
| C:TCEs | Major concerns | Low risk | No concerns | No concerns | No concerns | No concerns | Low |
| AT+RT:HIIT | Some concerns | Low risk | No concerns | Major concerns | No concerns | No concerns | Very low |
| AT+RT:TCEs | Some concerns | Low risk | No concerns | No concerns | No concerns | No concerns | Moderate |
| HIIT:RT | Some concerns | Low risk | No concerns | No concerns | No concerns | No concerns | Moderate |
| HIIT:TCEs | Some concerns | Low risk | No concerns | No concerns | No concerns | No concerns | Moderate |
| RT:TCEs | Some concerns | Low risk | No concerns | Major concerns | No concerns | No concerns | Very low |

**Table S9.5:** CINeMA Results of TG

| Comparison | Within-study bias | Reporting bias | Indirectness | Imprecision | Heterogeneity | Incoherence | Confidence rating |
| --- | --- | --- | --- | --- | --- | --- | --- |
| AT:AT+RT | Some concerns | Low risk | No concerns | No concerns | No concerns | No concerns | Moderate |
| AT:C | Some concerns | Low risk | No concerns | No concerns | No concerns | No concerns | Moderate |
| AT:HIIT | Some concerns | Low risk | No concerns | Major concerns | No concerns | No concerns | Very low |
| AT:RT | Some concerns | Low risk | No concerns | Major concerns | No concerns | No concerns | Very low |
| AT:TCEs | Major concerns | Low risk | No concerns | Major concerns | No concerns | No concerns | Very low |
| AT+RT:C | Some concerns | Low risk | No concerns | No concerns | No concerns | No concerns | Moderate |
| AT+RT:RT | Some concerns | Low risk | No concerns | No concerns | No concerns | Major concerns | Very low |
| C:HIIT | Some concerns | Low risk | No concerns | No concerns | No concerns | No concerns | Moderate |
| C:RT | Some concerns | Low risk | No concerns | No concerns | No concerns | No concerns | Moderate |
| C:TCEs | Major concerns | Low risk | No concerns | No concerns | No concerns | No concerns | Low |
| AT+RT:HIIT | Some concerns | Low risk | No concerns | Major concerns | No concerns | No concerns | Very low |
| AT+RT:TCEs | Some concerns | Low risk | No concerns | No concerns | No concerns | No concerns | Moderate |
| HIIT:RT | Some concerns | Low risk | No concerns | Major concerns | No concerns | No concerns | Very low |
| HIIT:TCEs | Some concerns | Low risk | No concerns | Major concerns | No concerns | No concerns | Very low |
| RT:TCEs | Some concerns | Low risk | No concerns | Major concerns | No concerns | No concerns | Very low |

**Table S9.6:** CINeMA Results of HDL

| Comparison | Within-study bias | Reporting bias | Indirectness | Imprecision | Heterogeneity | Incoherence | Confidence rating |
| --- | --- | --- | --- | --- | --- | --- | --- |
| AT:AT+RT | Some concerns | Low risk | No concerns | Major concerns | No concerns | No concerns | Very low |
| AT:C | Some concerns | Low risk | No concerns | No concerns | Major concerns | No concerns | Low |
| AT:HIIT | Some concerns | Low risk | No concerns | No concerns | Major concerns | No concerns | Low |
| AT:RT | Some concerns | Low risk | No concerns | Major concerns | No concerns | No concerns | Very low |
| AT:TCEs | Major concerns | Low risk | No concerns | Major concerns | No concerns | No concerns | Very low |
| AT+RT:C | Some concerns | Low risk | No concerns | No concerns | Major concerns | No concerns | Low |
| AT+RT:RT | Some concerns | Low risk | No concerns | Major concerns | No concerns | No concerns | Very low |
| C:HIIT | Some concerns | Low risk | No concerns | No concerns | No concerns | No concerns | Moderate |
| C:RT | Some concerns | Low risk | No concerns | No concerns | Major concerns | No concerns | Low |
| C:TCEs | Major concerns | Low risk | No concerns | No concerns | Major concerns | No concerns | Very low |
| AT+RT:HIIT | Some concerns | Low risk | No concerns | Major concerns | No concerns | No concerns | Very low |
| AT+RT:TCEs | Some concerns | Low risk | No concerns | Major concerns | No concerns | No concerns | Very low |
| HIIT:RT | Some concerns | Low risk | No concerns | No concerns | Major concerns | No concerns | Low |
| HIIT:TCEs | Some concerns | Low risk | No concerns | Major concerns | No concerns | No concerns | Very low |
| RT:TCEs | Some concerns | Low risk | No concerns | Major concerns | No concerns | No concerns | Very low |

**Table S9.7:** CINeMA Results of LDL

| Comparison | Within-study bias | Reporting bias | Indirectness | Imprecision | Heterogeneity | Incoherence | Confidence rating |
| --- | --- | --- | --- | --- | --- | --- | --- |
| AT:AT+RT | Some concerns | Low risk | No concerns | Major concerns | No concerns | No concerns | Very low |
| AT:C | Some concerns | Low risk | No concerns | No concerns | Major concerns | No concerns | Low |
| AT:HIIT | Some concerns | Low risk | No concerns | Major concerns | No concerns | No concerns | Very low |
| AT:RT | Some concerns | Low risk | No concerns | Major concerns | No concerns | No concerns | Very low |
| AT:TCEs | Some concerns | Low risk | No concerns | Major concerns | No concerns | No concerns | Very low |
| AT+RT:C | Some concerns | Low risk | No concerns | No concerns | Major concerns | No concerns | Low |
| AT+RT:RT | Some concerns | Low risk | No concerns | Major concerns | No concerns | No concerns | Very low |
| C:HIIT | Some concerns | Low risk | No concerns | No concerns | No concerns | No concerns | Moderate |
| C:RT | Some concerns | Low risk | No concerns | No concerns | Major concerns | No concerns | Low |
| C:TCEs | Some concerns | Low risk | No concerns | Major concerns | No concerns | No concerns | Very low |
| AT+RT:HIIT | Some concerns | Low risk | No concerns | Major concerns | No concerns | No concerns | Very low |
| AT+RT:TCEs | Some concerns | Low risk | No concerns | Major concerns | No concerns | No concerns | Very low |
| HIIT:RT | Some concerns | Low risk | No concerns | No concerns | Major concerns | No concerns | Low |
| HIIT:TCEs | Some concerns | Low risk | No concerns | Major concerns | No concerns | No concerns | Very low |
| RT:TCEs | Some concerns | Low risk | No concerns | Major concerns | No concerns | No concerns | Very low |

**Table S9.8:** CINeMA Results of BMI

| Comparison | Within-study bias | Reporting bias | Indirectness | Imprecision | Heterogeneity | Incoherence | Confidence rating |
| --- | --- | --- | --- | --- | --- | --- | --- |
| AT:AT+RT | Some concerns | Low risk | No concerns | Major concerns | No concerns | No concerns | Very low |
| AT:C | Some concerns | Low risk | No concerns | No concerns | No concerns | No concerns | Moderate |
| AT:HIIT | Some concerns | Low risk | No concerns | Major concerns | No concerns | No concerns | Very low |
| AT:RT | Some concerns | Low risk | No concerns | Major concerns | No concerns | No concerns | Very low |
| AT:TCEs | Major concerns | Low risk | No concerns | Major concerns | No concerns | No concerns | Very low |
| AT+RT:C | Some concerns | Low risk | No concerns | No concerns | No concerns | No concerns | Very low |
| C:HIIT | Some concerns | Low risk | No concerns | No concerns | Major concerns | No concerns | Low |
| C:RT | Some concerns | Low risk | No concerns | No concerns | No concerns | No concerns | Moderate |
| C:TCEs | Major concerns | Low risk | No concerns | Major concerns | No concerns | No concerns | Very low |
| HIIT:RT | Some concerns | Low risk | No concerns | Major concerns | No concerns | No concerns | Very low |
| AT+RT:HIIT | Some concerns | Low risk | No concerns | Major concerns | No concerns | No concerns | Very low |
| AT+RT:RT | Some concerns | Low risk | No concerns | Major concerns | No concerns | No concerns | Very low |
| AT+RT:TCEs | Some concerns | Low risk | No concerns | Major concerns | No concerns | No concerns | Low |
| HIIT:TCEs | Major concerns | Low risk | No concerns | Major concerns | No concerns | No concerns | Very low |
| RT:TCEs | Major concerns | Low risk | No concerns | Major concerns | No concerns | No concerns | Very low |

**Table S9.9:** Results of BW

| Comparison | Within-study bias | Reporting bias | Indirectness | Imprecision | Heterogeneity | Incoherence | Confidence rating |
| --- | --- | --- | --- | --- | --- | --- | --- |
| AT:C | Some concerns | Low risk | No concerns | No concerns | Major concerns | No concerns | Low |
| AT:HIIT | Some concerns | Low risk | No concerns | Major concerns | No concerns | No concerns | Very low |
| AT:RT | No concerns | Low risk | No concerns | Major concerns | No concerns | No concerns | Low |
| AT:TCEs | Major concerns | Low risk | No concerns | Major concerns | No concerns | No concerns | Very low |
| C:HIIT | Some concerns | Low risk | No concerns | No concerns | Major concerns | No concerns | Low |
| C:RT | Some concerns | Low risk | No concerns | Major concerns | No concerns | No concerns | Very low |
| C:TCEs | Major concerns | Low risk | No concerns | No concerns | Major concerns | No concerns | Very low |
| HIIT:RT | Some concerns | Low risk | No concerns | Major concerns | No concerns | No concerns | Very low |
| HIIT:TCEs | Some concerns | Low risk | No concerns | Major concerns | No concerns | No concerns | Very low |
| RT:TCEs | Some concerns | Low risk | No concerns | Major concerns | No concerns | No concerns | Very low |

**Table S9.10:** CINeMA Results of WC

| Comparison | Within-study bias | Reporting bias | Indirectness | Imprecision | Heterogeneity | Incoherence | Confidence rating |
| --- | --- | --- | --- | --- | --- | --- | --- |
| AT:C | Some concerns | Low risk | No concerns | No concerns | No concerns | No concerns | Moderate |
| AT:HIIT | Some concerns | Low risk | No concerns | Major concerns | No concerns | No concerns | Very low |
| AT:RT | Some concerns | Low risk | No concerns | No concerns | Major concerns | No concerns | Low |
| AT:TCEs | Major concerns | Low risk | No concerns | Major concerns | No concerns | No concerns | Very low |
| C:HIIT | Some concerns | Low risk | No concerns | Major concerns | No concerns | No concerns | Very low |
| C:RT | Some concerns | Low risk | No concerns | Major concerns | No concerns | No concerns | Very low |
| C:TCEs | Major concerns | Low risk | No concerns | No concerns | Major concerns | No concerns | Low |
| HIIT:RT | Some concerns | Low risk | No concerns | Major concerns | No concerns | No concerns | Very low |
| HIIT:TCEs | Some concerns | Low risk | No concerns | Major concerns | No concerns | No concerns | Very low |
| RT:TCEs | Some concerns | Low risk | No concerns | Major concerns | No concerns | No concerns | Very low |

#

# Appendix 10: Funnel plots

**Figure S10.1:** Funnel plot of HbA1c

**
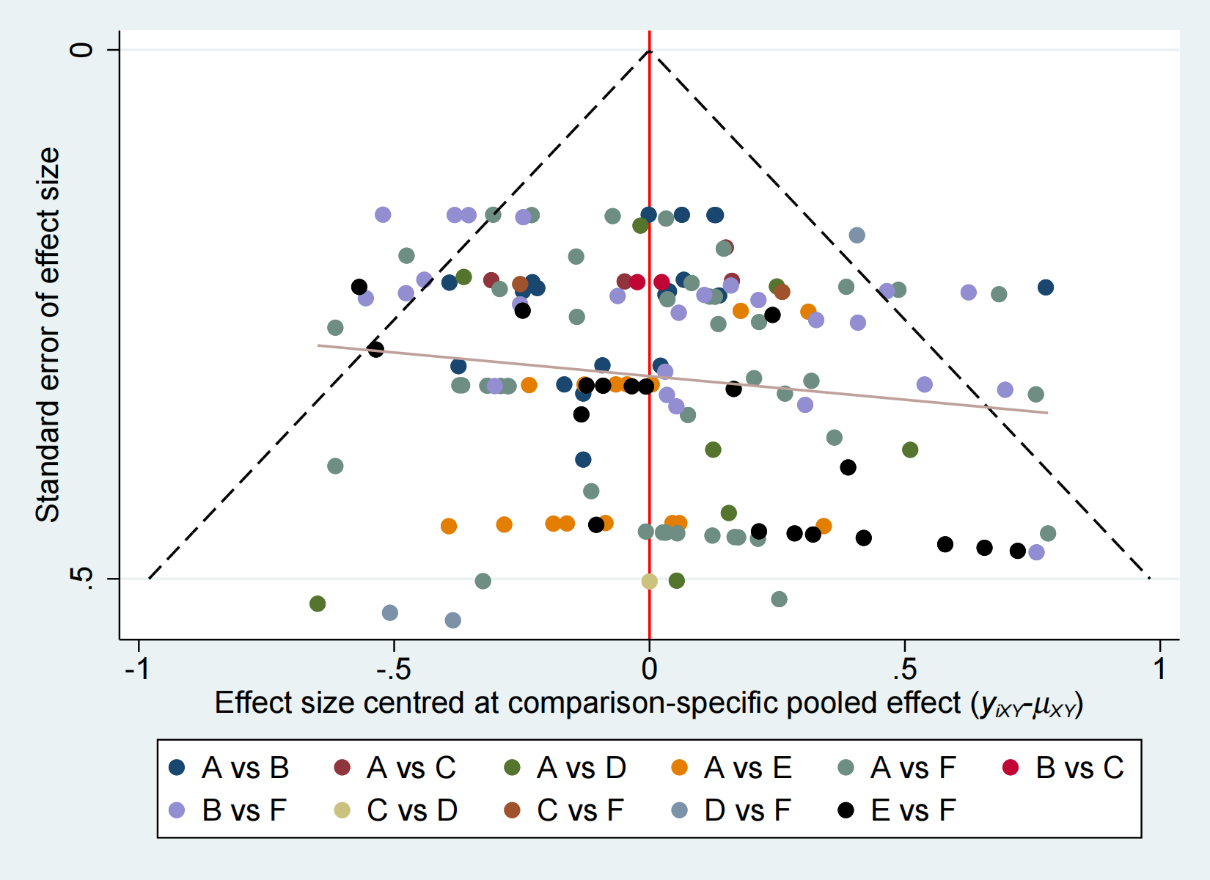
**

**Figure S10.2:** Funnel plot of FBG


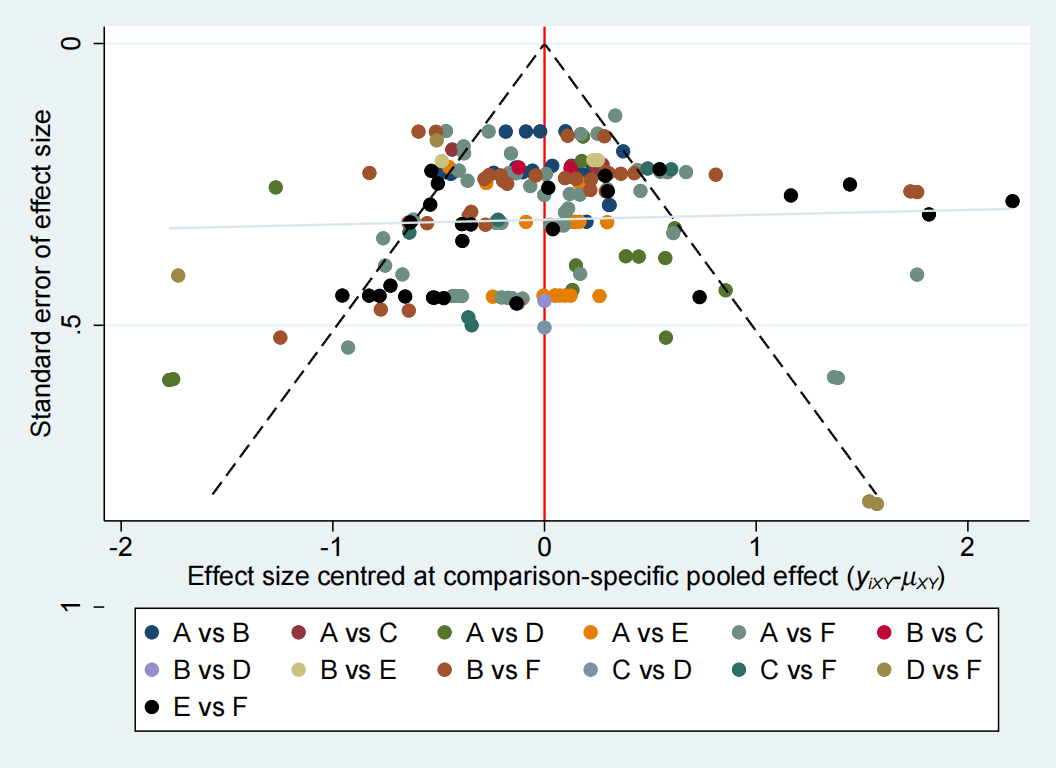


**Figure S10.3:** Funnel plot of 2hPG


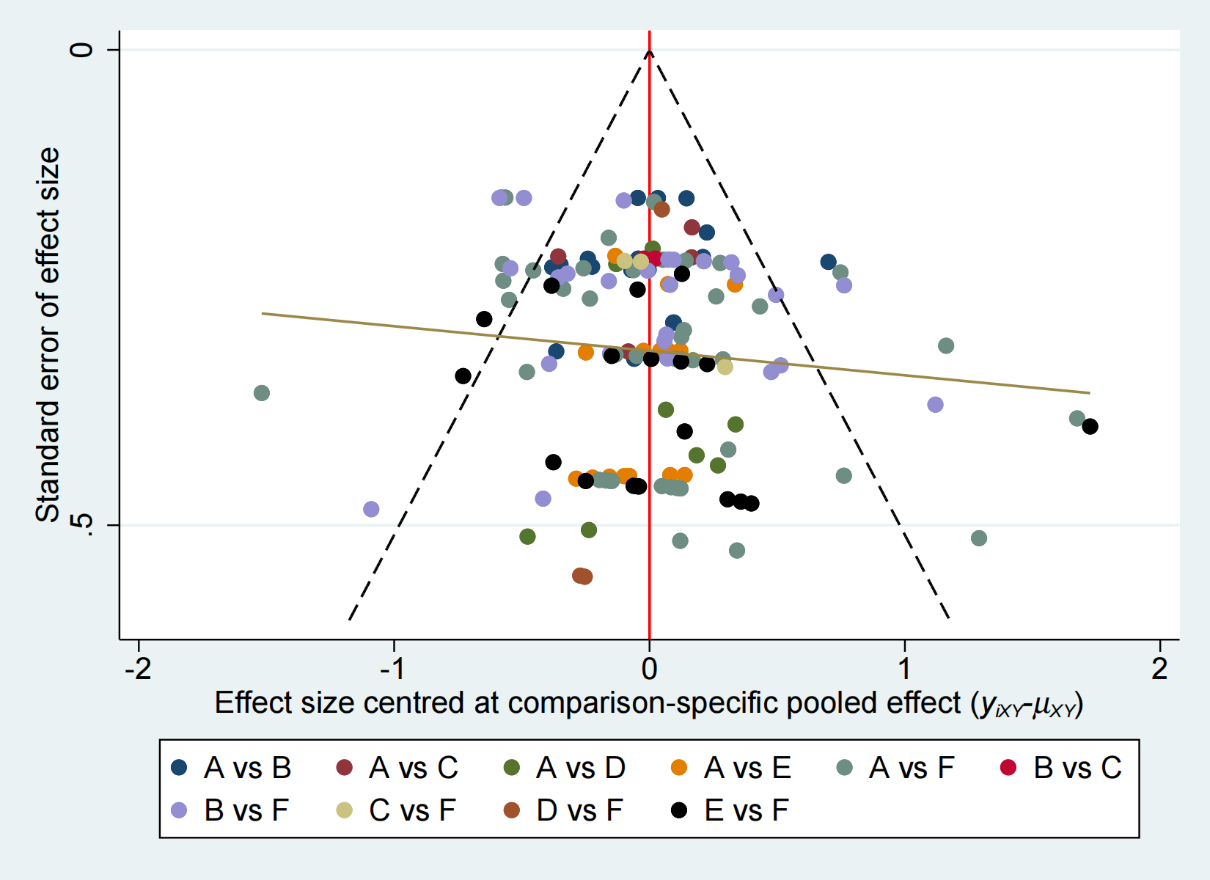


**Figure S10.4:** Funnel plot of HDL


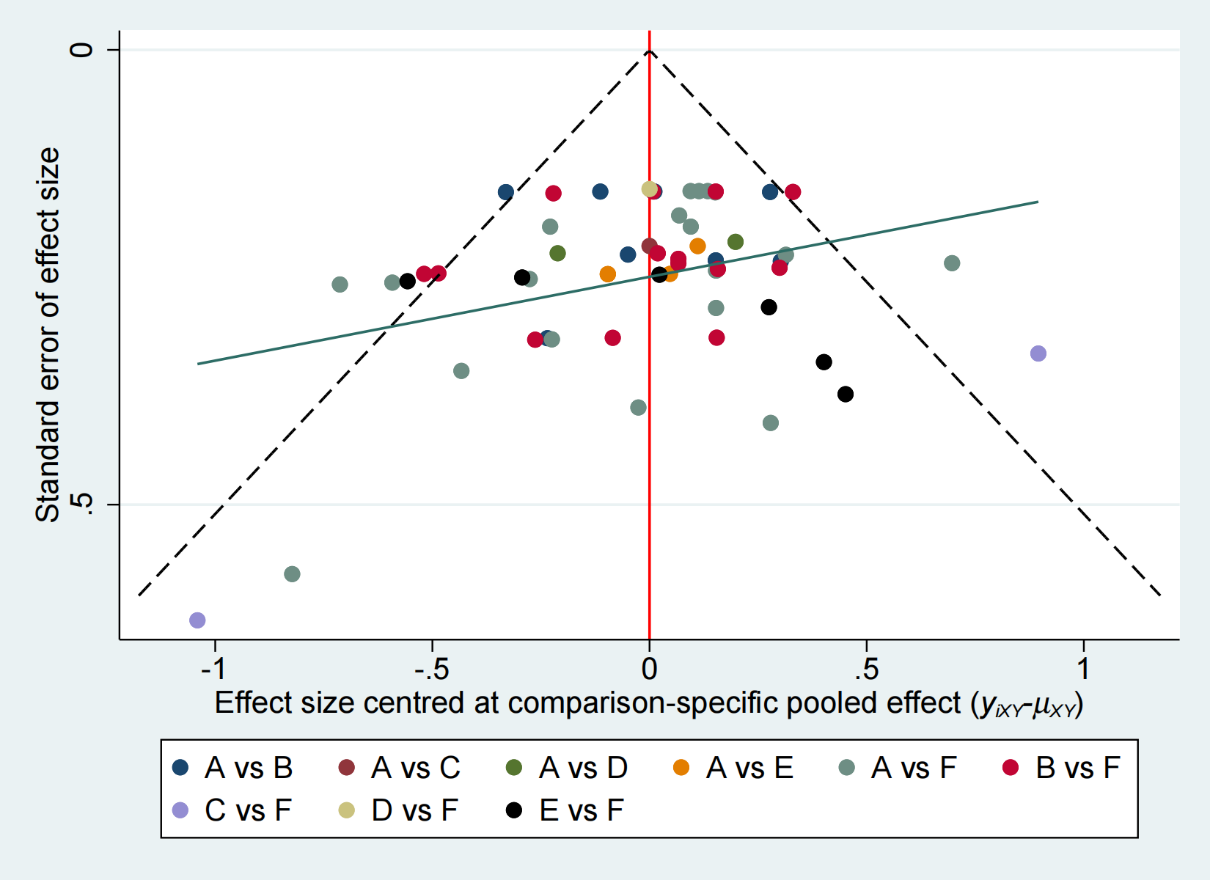


**Figure S10.5:** Funnel plot of LDL


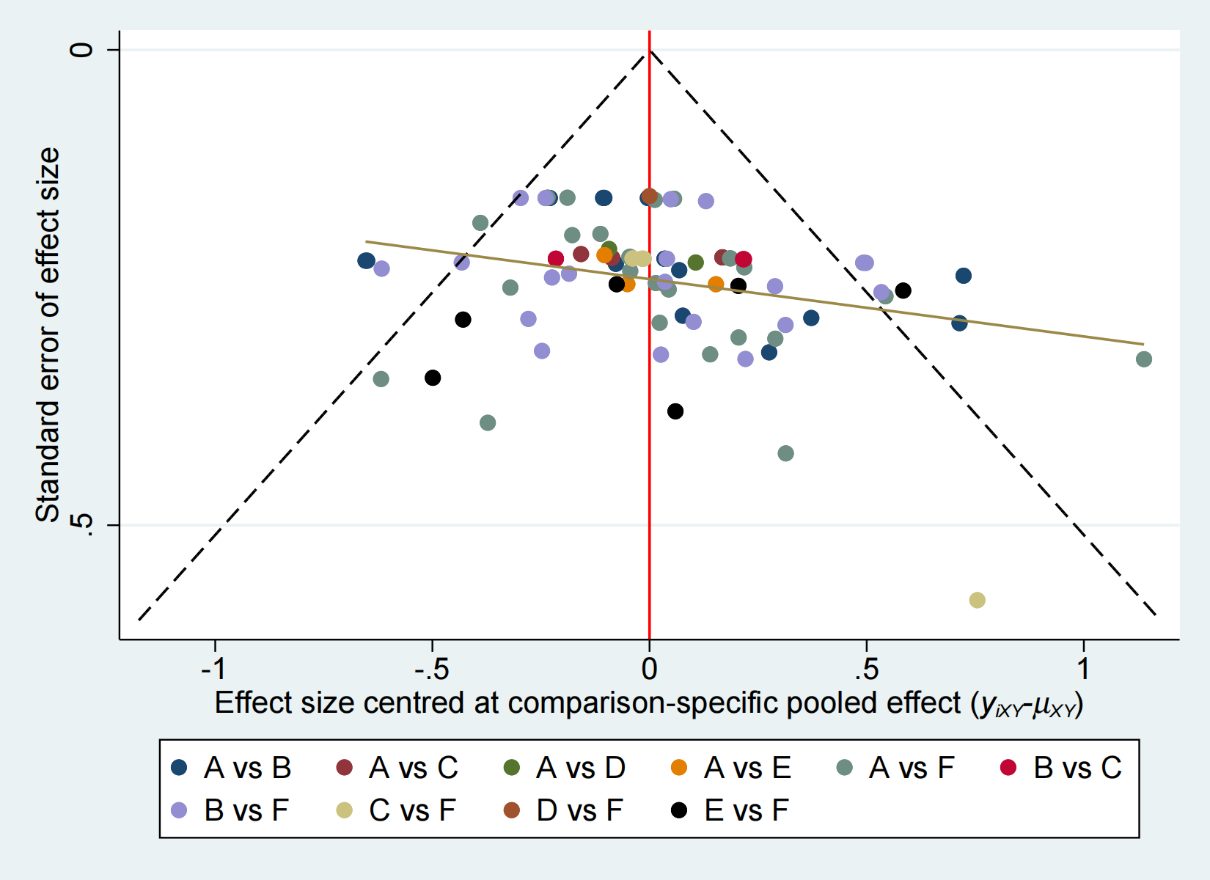


**Figure S10.6:** Funnel plot of TC


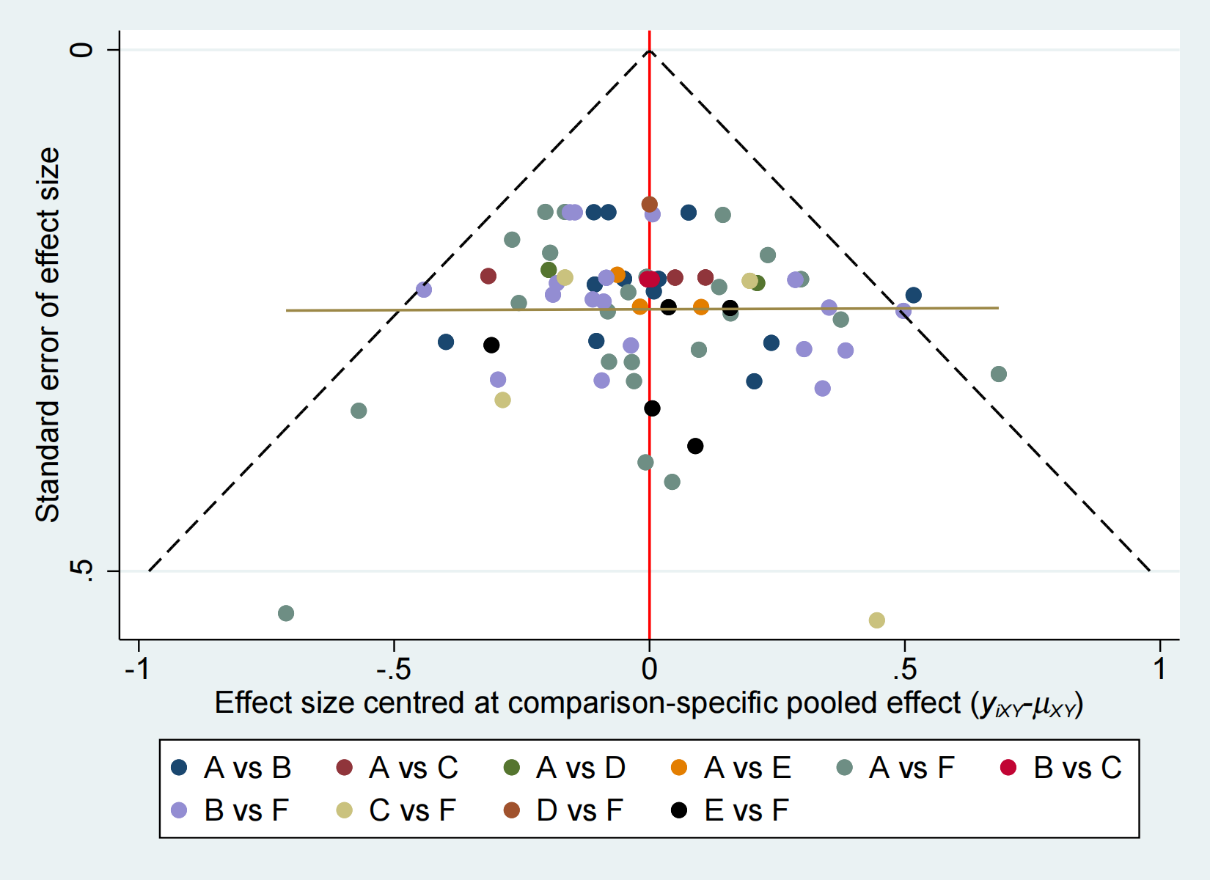


**Figure S10.7:** Funnel plot of TC


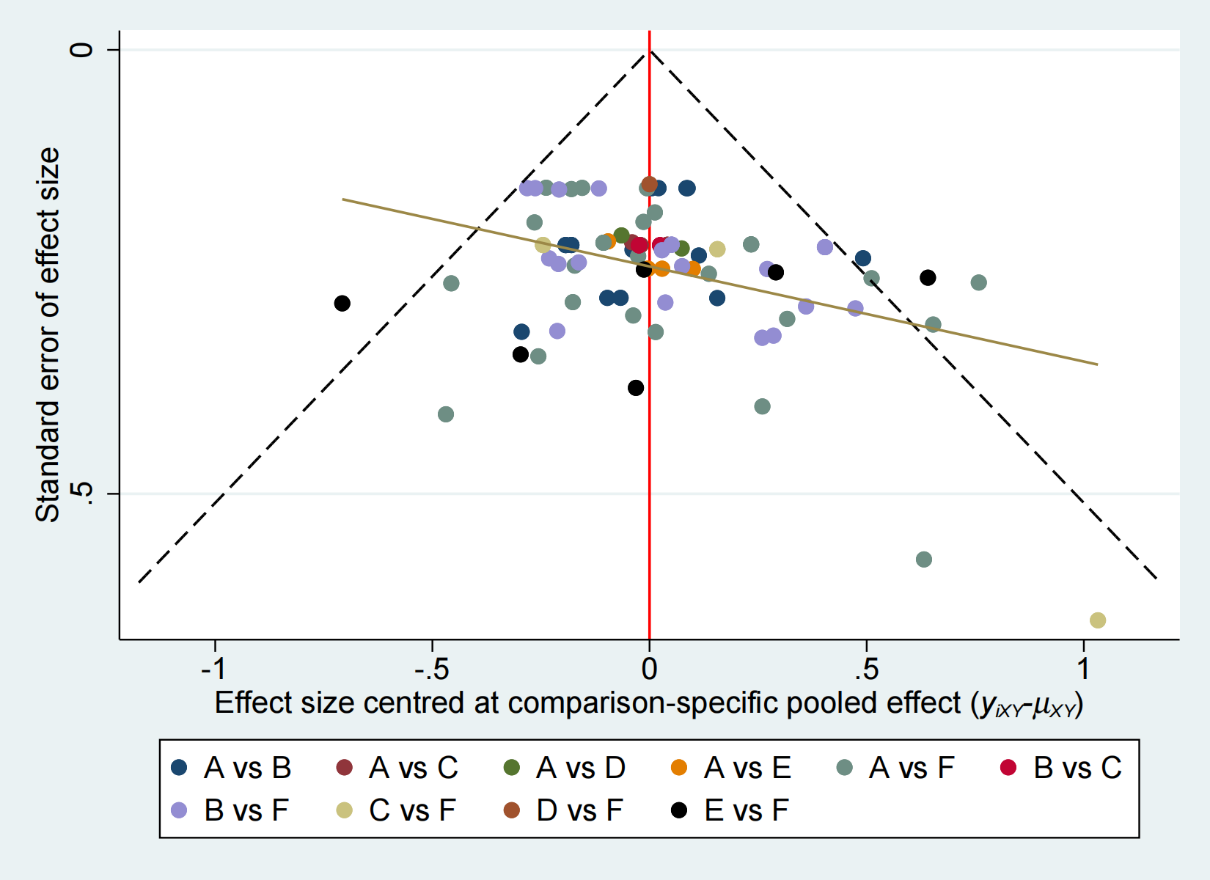


**Figure S10.8:** Funnel plot of BMI


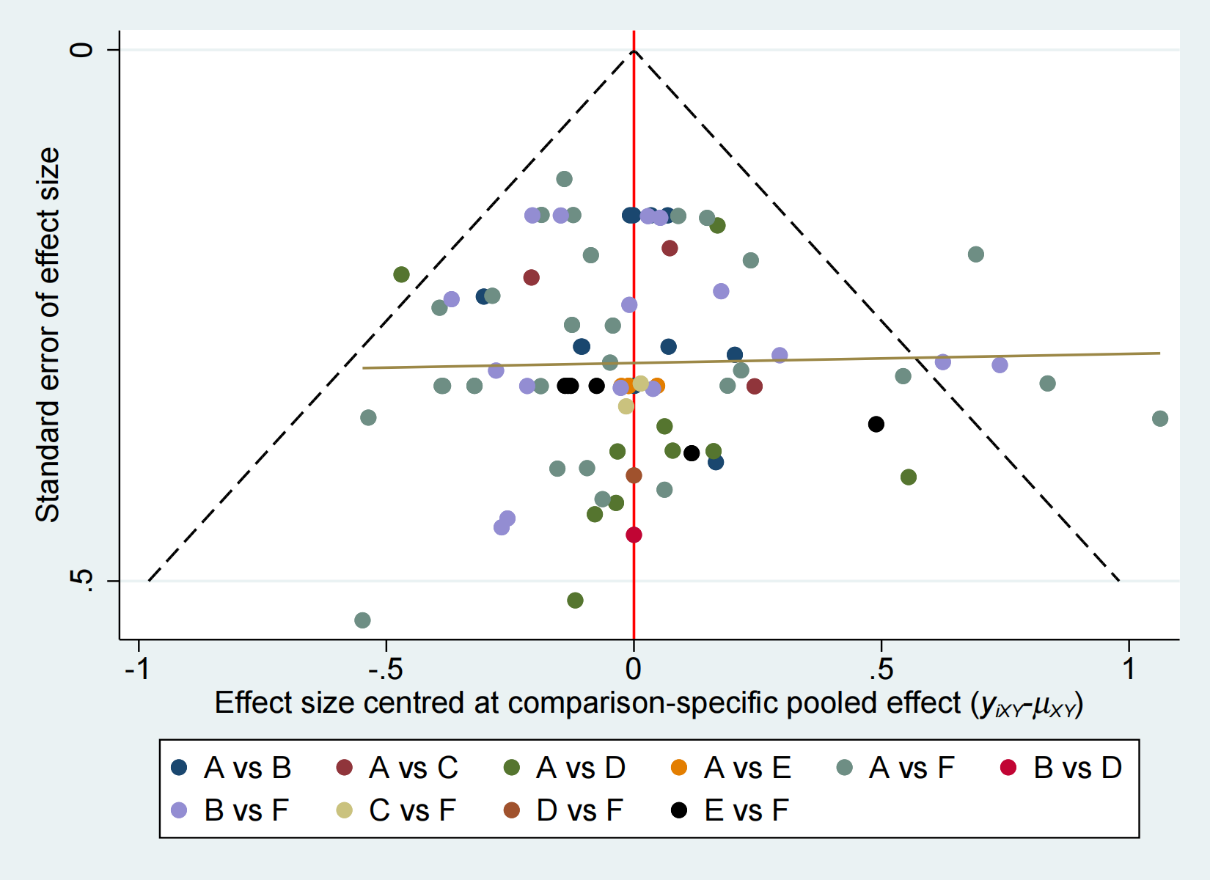


**Figure S10.9:** Funnel plot of BW


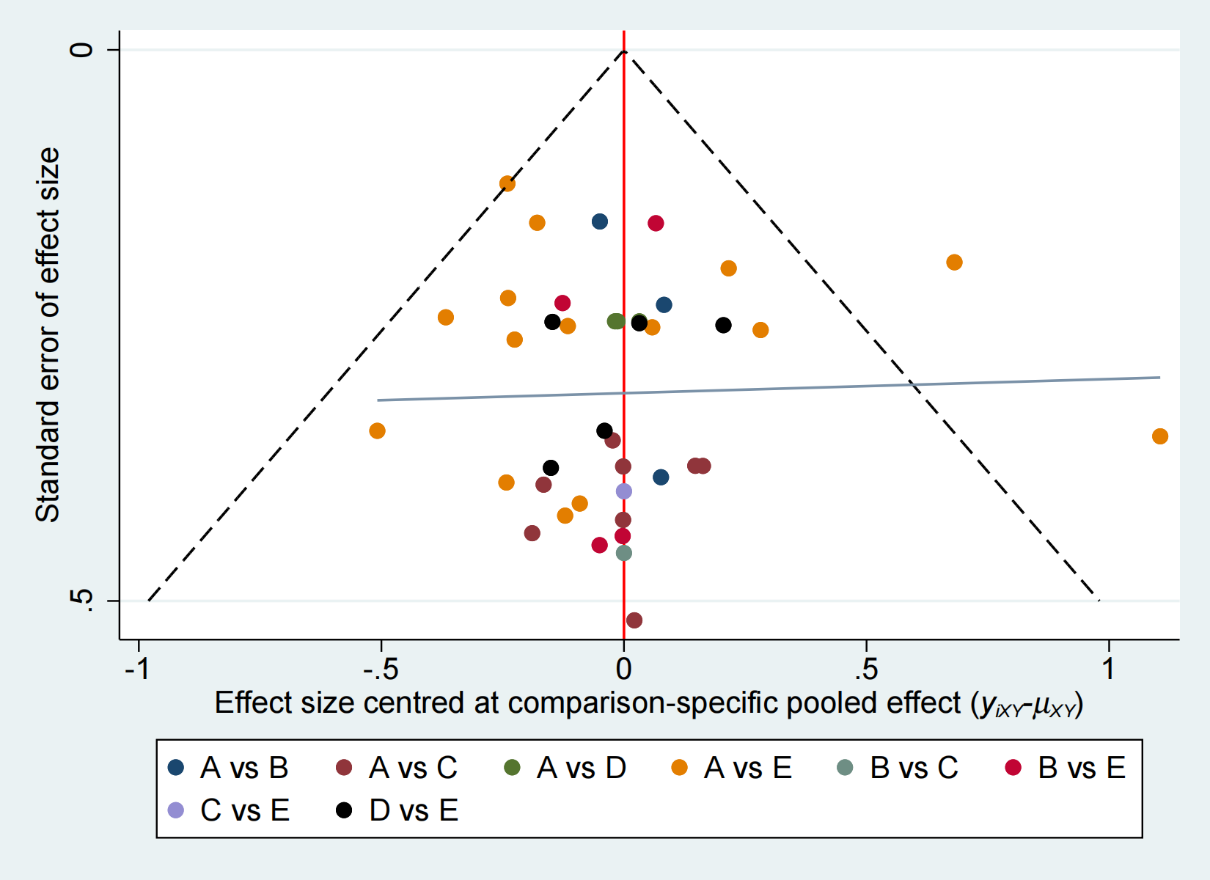


**Figure S10.10:** Funnel plot of BW


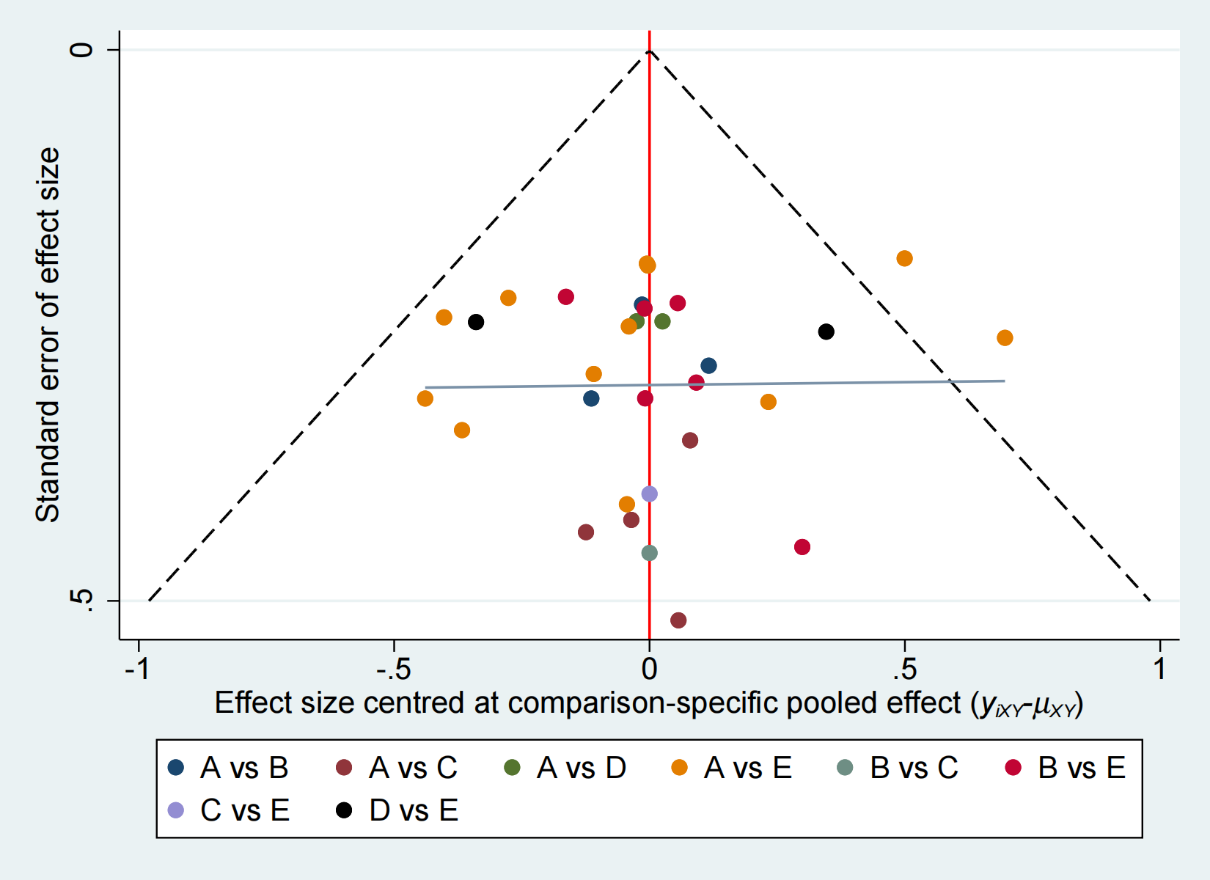


# Appendix 11: Subgroup analysis of different intensities of exercise interventions on prediabetes

**Figure S11: Effects of different intensities of exercise on various indicators of prediabetes.**

Network meta-analysis maps of the studies examining the efficacy of different exercise intensities on various indicators of prediabetes: (A) HbA1c, (B) 2hPG, (C)TC, (D) TG, (E) HDL, (F) LDL, (G) BMI, (H) BW,(i) WC. The size of the nodes relates to the number of participants in each intervention type, and the thickness of lines between the interventions relates to the number of studies for that comparison.

(A1) Network map of HbA1c


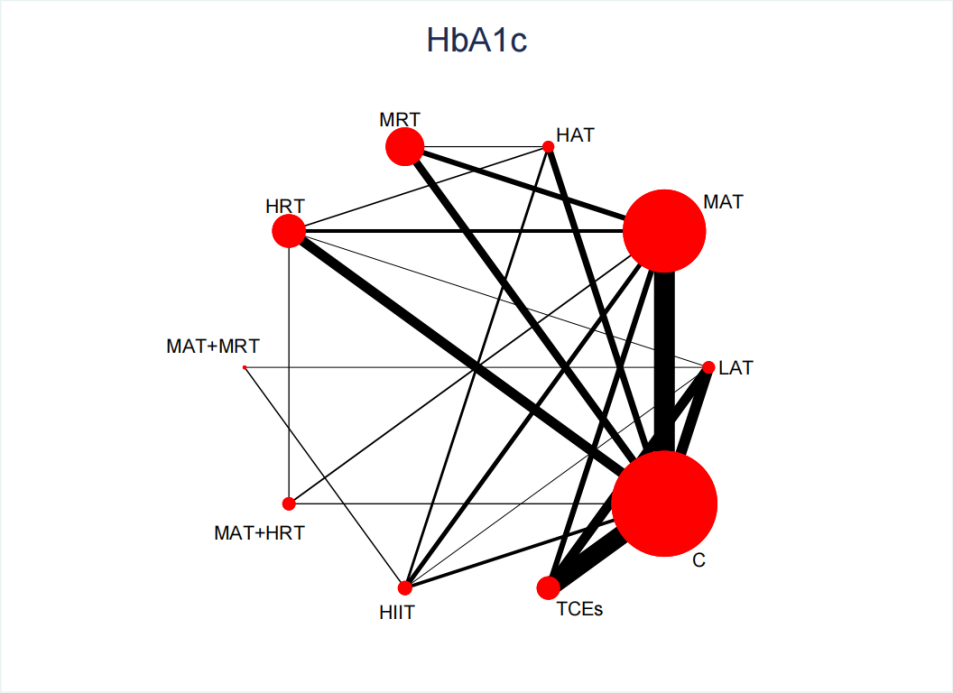


(A2) Forest plot of HbA1c


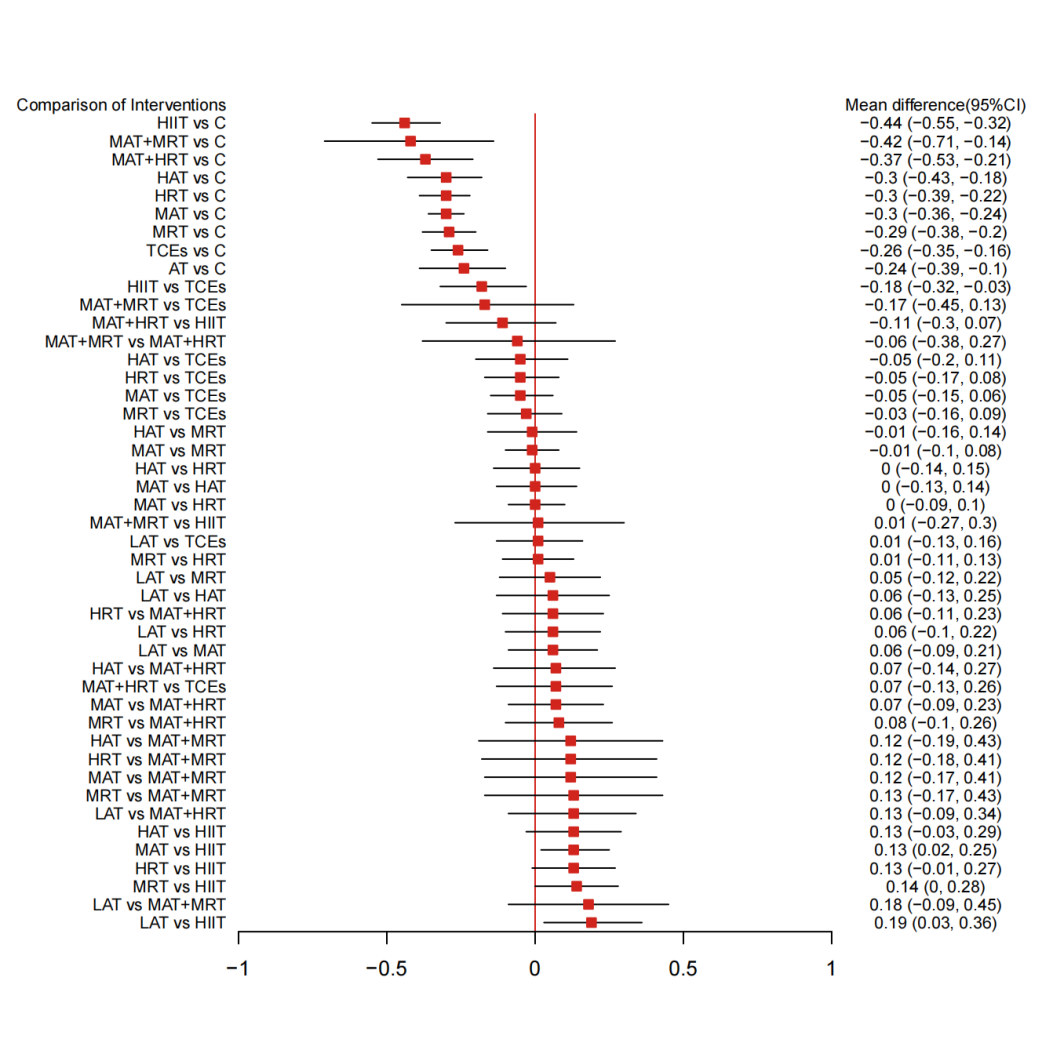


(A3) League table of HbA1c

| LAT |  |  |  |  |  |  |  |  |  |
| --- | --- | --- | --- | --- | --- | --- | --- | --- | --- |
| 0.03  (-0.13, 0.18) | MAT |  |  |  |  |  |  |  |  |
| -0.01  (-0.19, 0.17) | -0.04  (-0.16, 0.08) | HAT |  |  |  |  |  |  |  |
| 0.03  (-0.15, 0.2) | 0  (-0.09, 0.08) | 0.04  (-0.09, 0.17) | MRT |  |  |  |  |  |  |
| 0.05  (-0.12, 0.21) | 0.02  (-0.06, 0.1) | 0.06  (-0.07, 0.18) | 0.02  (-0.09, 0.13) | HRT |  |  |  |  |  |
| 0.17  (-0.12, 0.46) | 0.14  (-0.17, 0.45) | 0.18  (-0.14, 0.5) | 0.14  (-0.17, 0.46) | 0.12  (-0.19, 0.44) | MAT+MRT |  |  |  |  |
| 0.03  (-0.16, 0.22) | 0  (-0.11, 0.12) | 0.04  (-0.11, 0.2) | 0.01  (-0.13, 0.15) | -0.02  (-0.14, 0.11) | -0.14  (-0.46, 0.18) | MAT+HRT |  |  |  |
| 0.17  (0, 0.34) | 0.14  (0.03, 0.25) | 0.18  (0.03, 0.33) | 0.14  (0.01, 0.28) | 0.12  (-0.01, 0.25) | 0  (-0.31, 0.3) | 0.14  (-0.02, 0.29) | HIIT |  |  |
| 0.02  (-0.13, 0.18) | 0  (-0.11, 0.1) | 0.04  (-0.11, 0.18) | 0  (-0.13, 0.13) | -0.02  (-0.15, 0.1) | -0.14  (-0.46, 0.17) | -0.01  (-0.16, 0.14) | -0.14  (-0.28, 0) | TCEs |  |
| -0.25  (-0.4, -0.09) | -0.27  (-0.33, -0.22) | -0.23  (-0.34, -0.13) | -0.27  (-0.36, -0.18) | -0.29  (-0.37, -0.22) | -0.41  (-0.72, -0.11) | -0.28  (-0.39, -0.16) | -0.41  (-0.53, -0.3) | -0.27  (-0.37, -0.17) | C |

(B1) Network map of FBG


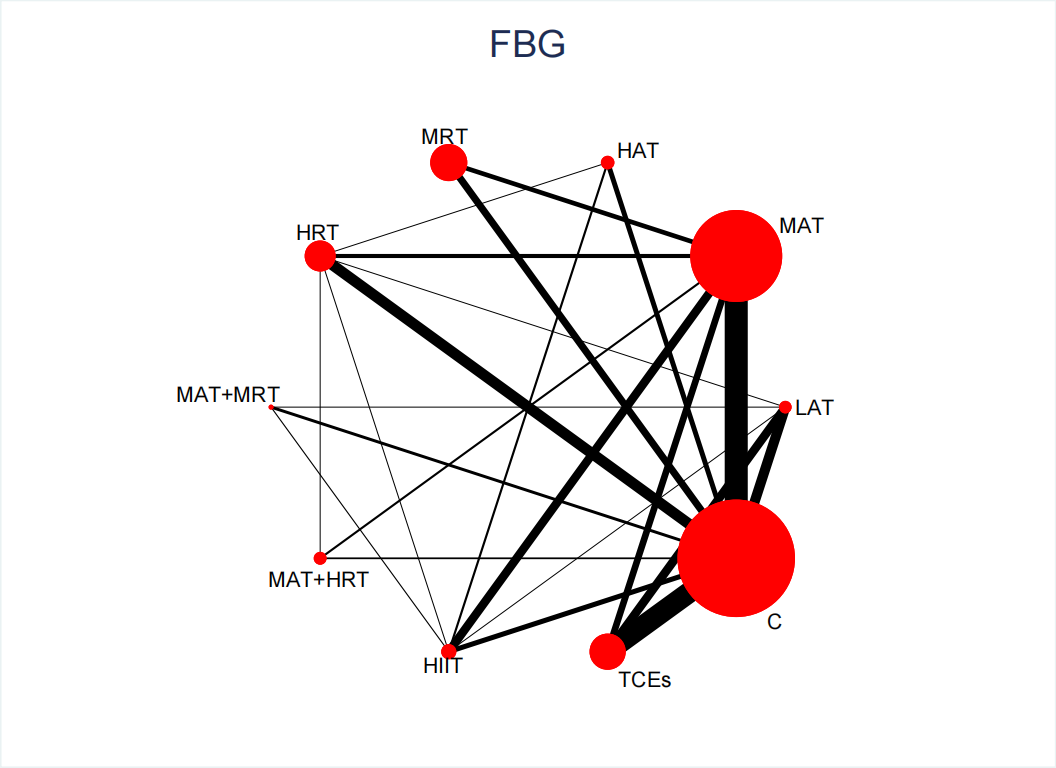


(B2) Forest plot of FBG


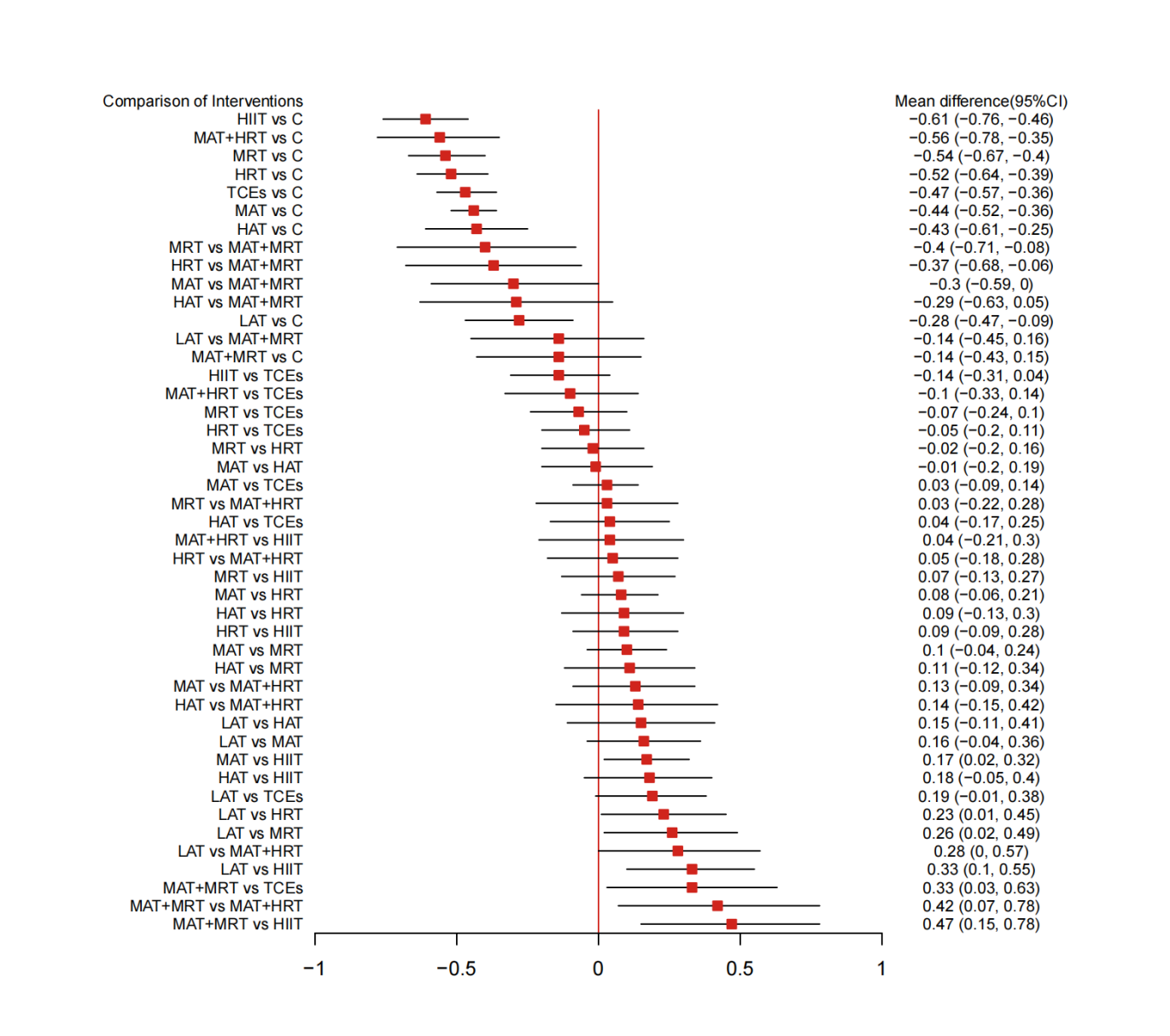


(B3) League table of FBG

| LAT |  |  |  |  |  |  |  |  |  |
| --- | --- | --- | --- | --- | --- | --- | --- | --- | --- |
| 0.16  (-0.04, 0.36) | MAT |  |  |  |  |  |  |  |  |
| 0.15  (-0.11, 0.41) | -0.01  (-0.2, 0.19) | HAT |  |  |  |  |  |  |  |
| 0.26  (0.02, 0.49) | 0.1  (-0.04, 0.24) | 0.11  (-0.12, 0.34) | MRT |  |  |  |  |  |  |
| 0.23  (0.01, 0.45) | 0.08  (-0.06, 0.21) | 0.09  (-0.13, 0.3) | -0.02  (-0.2, 0.16) | HRT |  |  |  |  |  |
| -0.14  (-0.45, 0.16) | -0.3  (-0.59, 0) | -0.29  (-0.63, 0.05) | -0.4  (-0.71, -0.08) | -0.37  (-0.68, -0.06) | MAT+MRT |  |  |  |  |
| 0.28  (0, 0.57) | 0.13  (-0.09, 0.34) | 0.14  (-0.15, 0.42) | 0.03  (-0.22, 0.28) | 0.05  (-0.18, 0.28) | 0.42  (0.07, 0.78) | MAT+HRT |  |  |  |
| 0.33  (0.1, 0.55) | 0.17 (0.02, 0.32) | 0.18  (-0.05, 0.4) | 0.07  (-0.13, 0.27) | 0.09  (-0.09, 0.28) | 0.47  (0.15, 0.78) | 0.04  (-0.21, 0.3) | HIIT |  |  |
| 0.19  (-0.01, 0.38) | 0.03  (-0.09, 0.14) | 0.04  (-0.17, 0.25) | -0.07  (-0.24, 0.1) | -0.05  (-0.2, 0.11) | 0.33  (0.03, 0.63) | -0.1  (-0.33, 0.14) | -0.14  (-0.31, 0.04) | TCEs |  |
| -0.28  (-0.47, -0.09) | -0.44  (-0.52, -0.36) | -0.43  (-0.61, -0.25) | -0.54  (-0.67, -0.4) | -0.52  (-0.64, -0.39) | -0.14  (-0.43, 0.15) | -0.56  (-0.78, -0.35) | -0.61  (-0.76, -0.46) | -0.47  (-0.57, -0.36) | C |

(C1) Network map of 2hPG


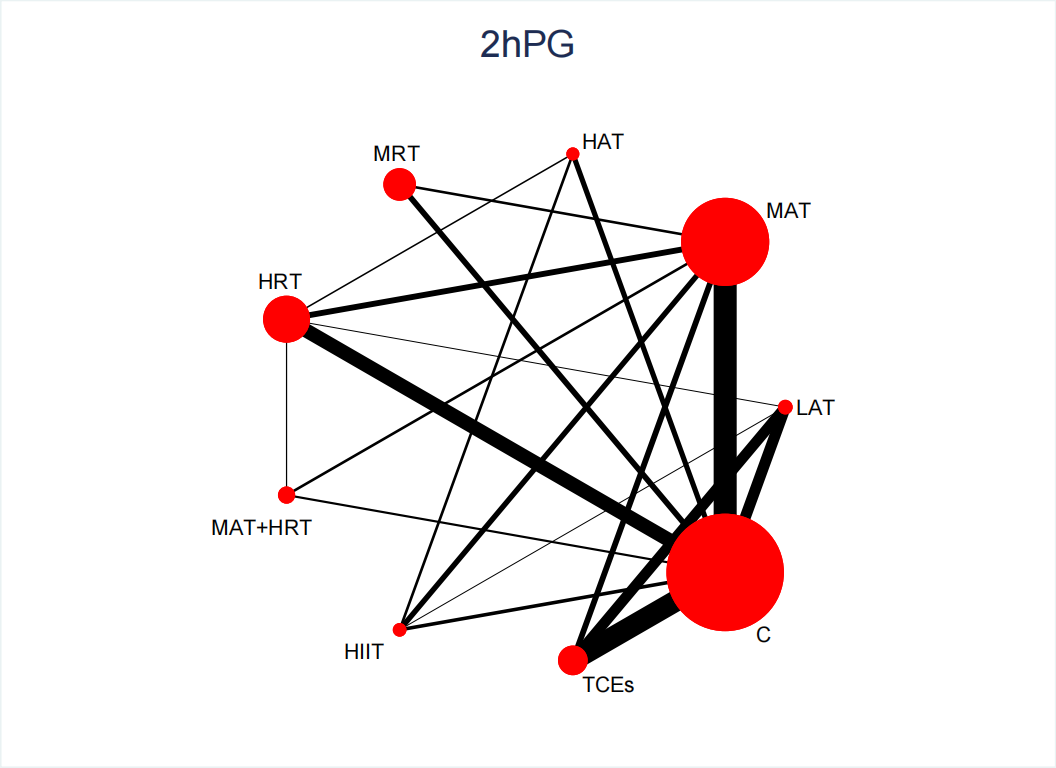


(C2) Forest plot of 2hPG


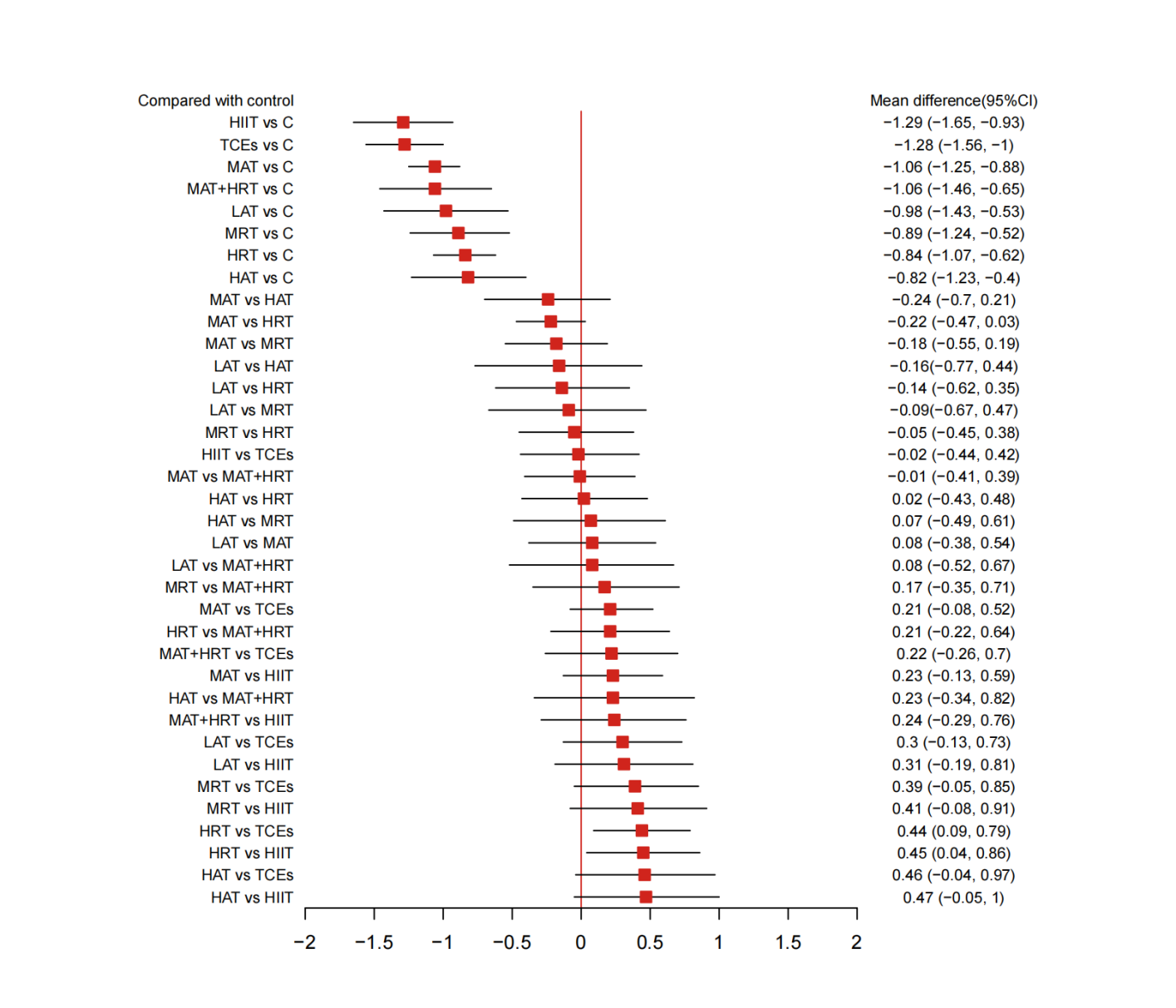


(C3) League table of 2hPG

| LAT |  |  |  |  |  |  |  |  |
| --- | --- | --- | --- | --- | --- | --- | --- | --- |
| 0.08  (-0.38, 0.54) | MAT |  |  |  |  |  |  |  |
| -0.16  (-0.77, 0.44) | -0.24  (-0.7, 0.21) | HAT |  |  |  |  |  |  |
| -0.09  (-0.67, 0.47) | -0.18  (-0.55, 0.19) | 0.07  (-0.49, 0.61) | MRT |  |  |  |  |  |
| -0.14  (-0.62, 0.35) | -0.22  (-0.47, 0.03) | 0.02  (-0.43, 0.48) | -0.05  (-0.45, 0.38) | HRT |  |  |  |  |
| 0.08  (-0.52, 0.67) | -0.01  (-0.41, 0.39) | 0.23  (-0.34, 0.82) | 0.17  (-0.35, 0.71) | 0.21  (-0.22, 0.64) | MAT+HRT |  |  |  |
| 0.31  (-0.19, 0.81) | 0.23  (-0.13, 0.59) | 0.47  (-0.05, 1) | 0.41  (-0.08, 0.91) | 0.45  (0.04, 0.86) | 0.24  (-0.29, 0.76) | HIIT |  |  |
| 0.3  (-0.13, 0.73) | 0.21  (-0.08, 0.52) | 0.46  (-0.04, 0.97) | 0.39  (-0.05, 0.85) | 0.44  (0.09, 0.79) | 0.22  (-0.26, 0.7) | -0.02  (-0.44, 0.42) | TCEs |  |
| -0.98  (-1.43, -0.53) | -1.06  (-1.25, -0.88) | -0.82  (-1.23, -0.4) | -0.89  (-1.24, -0.52) | -0.84  (-1.07, -0.62) | -1.06  (-1.46, -0.65) | -1.29  (-1.65, -0.93) | -1.28  (-1.56, -1) | C |

(D1) Network map of TC


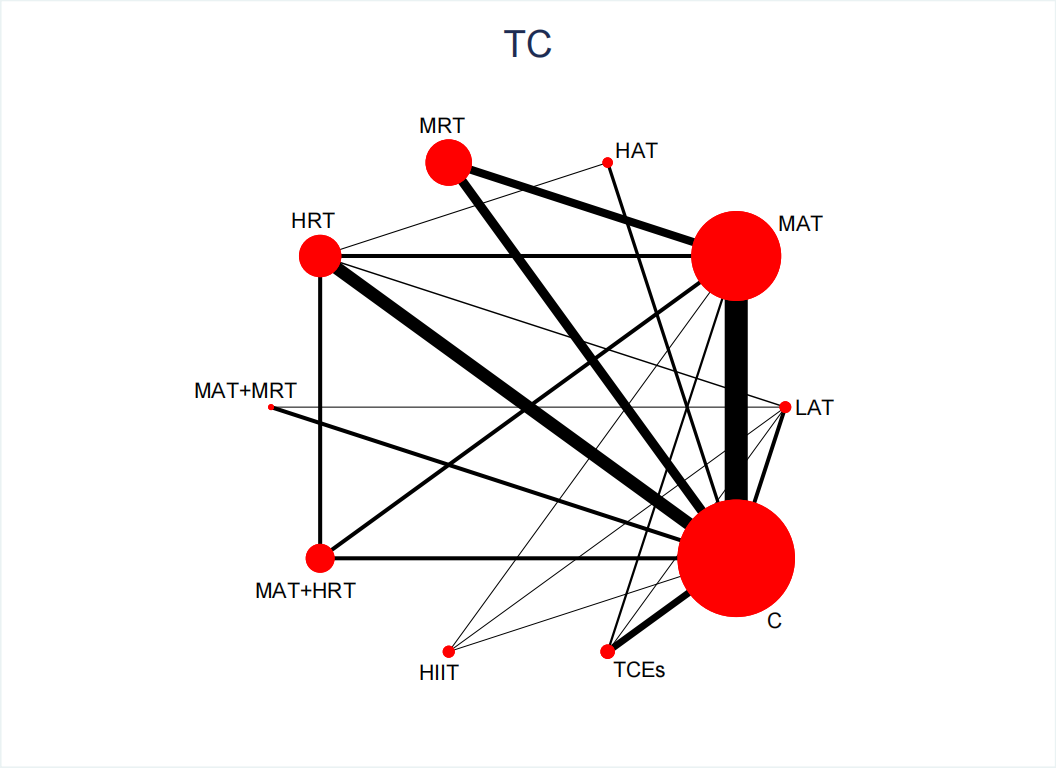


(D2) Forest plot of TC


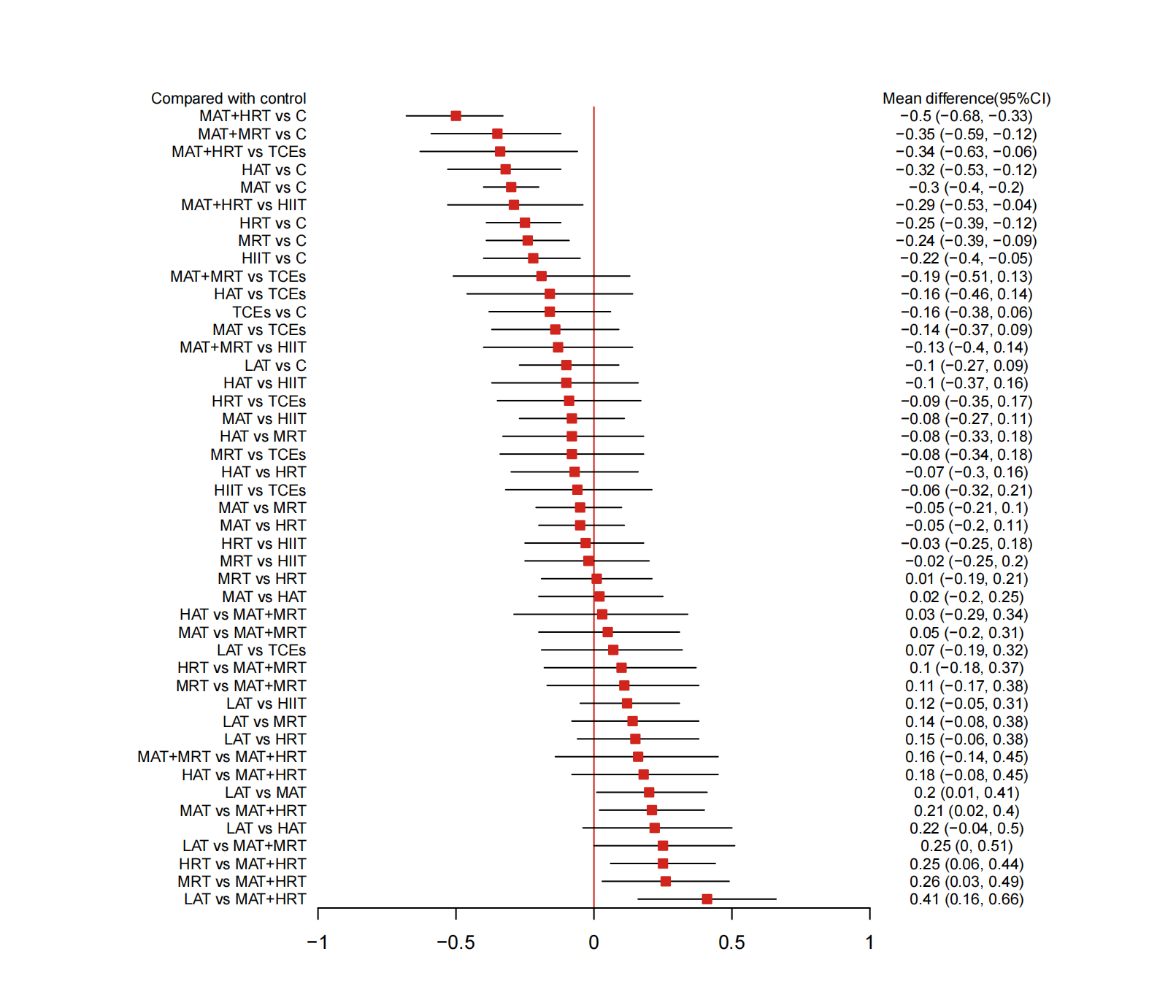


(D3) League table of TC

| LAT |  |  |  |  |  |  |  |  |  |
| --- | --- | --- | --- | --- | --- | --- | --- | --- | --- |
| 0.2  (0.01, 0.41) | MAT |  |  |  |  |  |  |  |  |
| 0.22  (-0.04, 0.5) | 0.02  (-0.2, 0.25) | HAT |  |  |  |  |  |  |  |
| 0.14  (-0.08, 0.38) | -0.05  (-0.21, 0.1) | -0.08  (-0.33, 0.18) | MRT |  |  |  |  |  |  |
| 0.15  (-0.06, 0.38) | -0.05  (-0.2, 0.11) | -0.07  (-0.3, 0.16) | 0.01  (-0.19, 0.21) | HRT |  |  |  |  |  |
| 0.25  (0, 0.51) | 0.05  (-0.2, 0.31) | 0.03  (-0.29, 0.34) | 0.11  (-0.17, 0.38) | 0.1  (-0.18, 0.37) | MAT+MRT |  |  |  |  |
| 0.41  (0.16, 0.66) | 0.21  (0.02, 0.4) | 0.18  (-0.08, 0.45) | 0.26  (0.03, 0.49) | 0.25  (0.06, 0.44) | 0.16  (-0.14, 0.45) | MAT+HRT |  |  |  |
| 0.12  (-0.05, 0.31) | -0.08  (-0.27, 0.11) | -0.1  (-0.37, 0.16) | -0.02  (-0.25, 0.2) | -0.03  (-0.25, 0.18) | -0.13  (-0.4, 0.14) | -0.29  (-0.53, -0.04) | HIIT |  |  |
| 0.07  (-0.19, 0.32) | -0.14  (-0.37, 0.09) | -0.16  (-0.46, 0.14) | -0.08  (-0.34, 0.18) | -0.09  (-0.35, 0.17) | -0.19  (-0.51, 0.13) | -0.34  (-0.63, -0.06) | -0.06  (-0.32, 0.21) | TCEs |  |
| -0.1  (-0.27, 0.09) | -0.3  (-0.4, -0.2) | -0.32  (-0.53, -0.12) | -0.24  (-0.39, -0.09) | -0.25  (-0.39, -0.12) | -0.35  (-0.59, -0.12) | -0.5  (-0.68, -0.33) | -0.22  (-0.4, -0.05) | -0.16  (-0.38, 0.06) | C |

(E1) Network map of TG


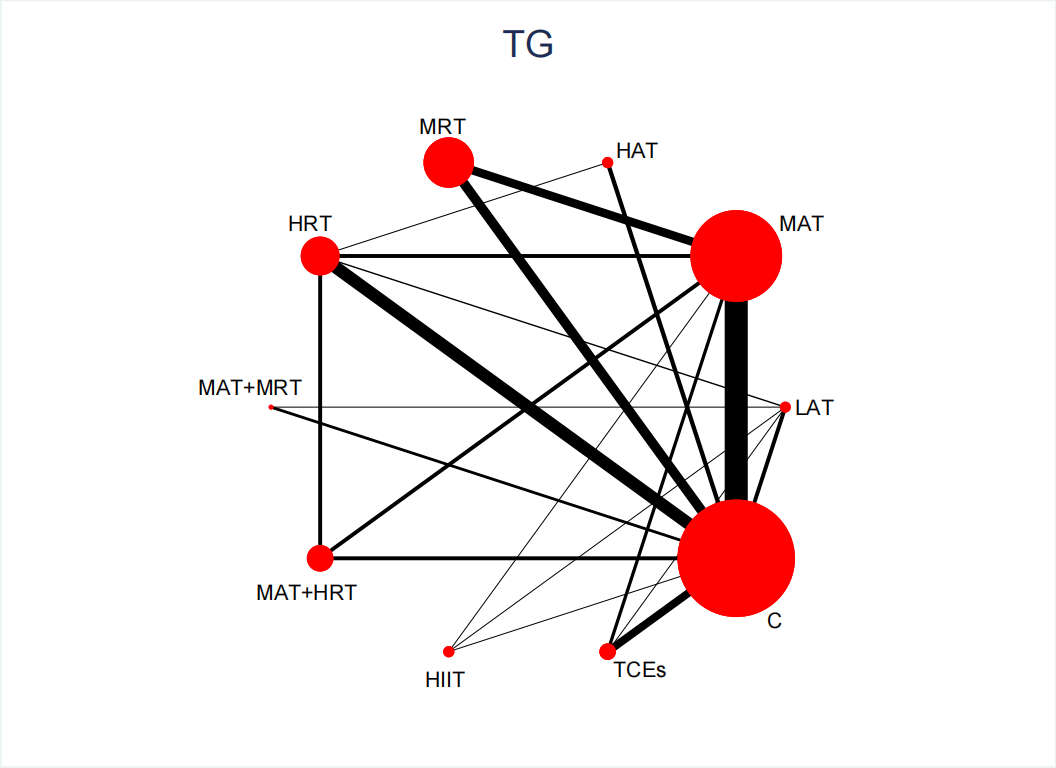


(E2) Forest plot of TG


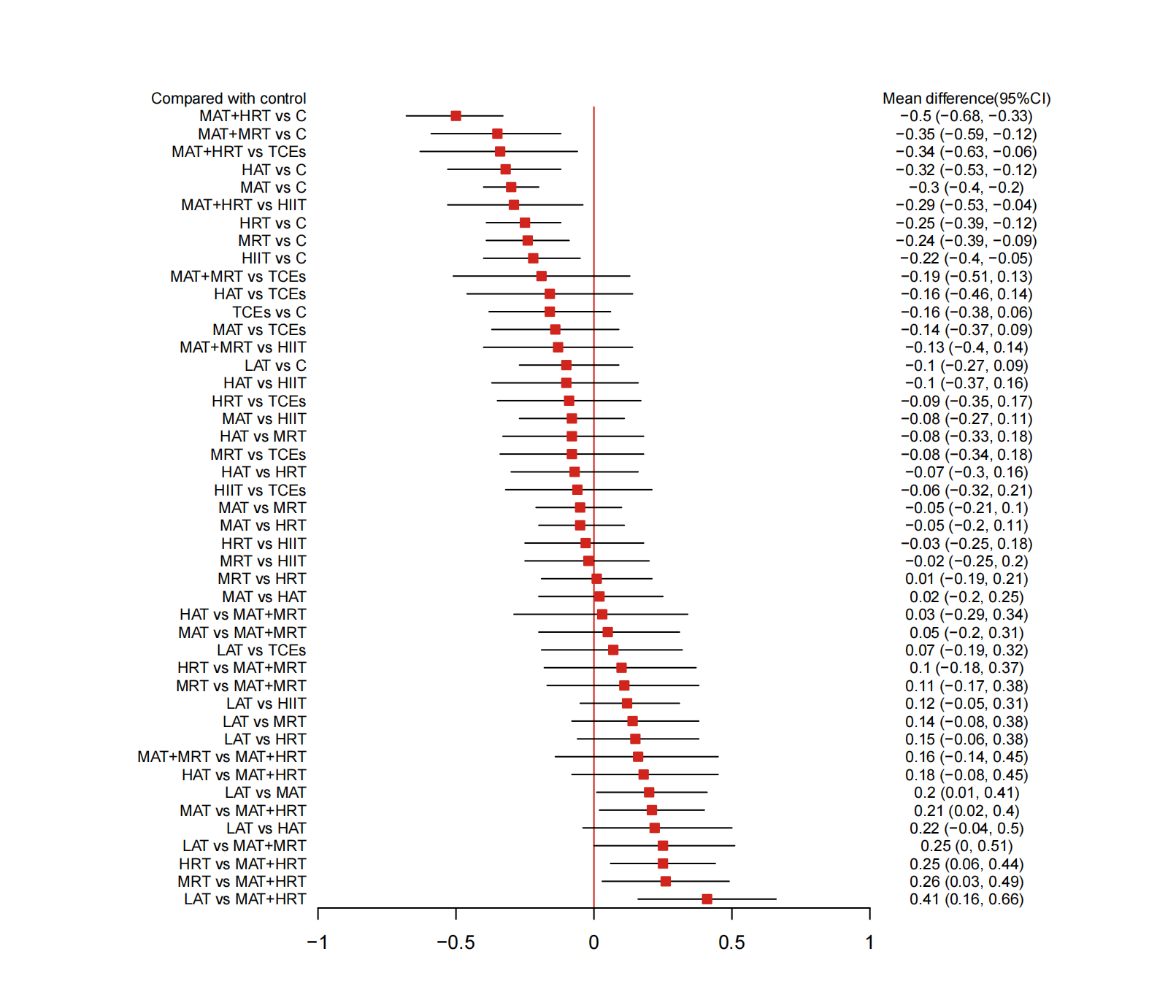


(E3) League table of TG

| LAT |  |  |  |  |  |  |  |  |  |
| --- | --- | --- | --- | --- | --- | --- | --- | --- | --- |
| -0.04  (-0.19, 0.15) | MAT |  |  |  |  |  |  |  |  |
| -0.1  (-0.31, 0.14) | -0.06  (-0.23, 0.11) | HAT |  |  |  |  |  |  |  |
| -0.06  (-0.23, 0.14) | -0.02  (-0.12, 0.08) | 0.04  (-0.15, 0.23) | MRT |  |  |  |  |  |  |
| 0.07  (-0.12, 0.28) | 0.1  (-0.03, 0.24) | 0.16  (-0.02, 0.36) | 0.12  (-0.03, 0.28) | HRT |  |  |  |  |  |
| 0.28  (0.05, 0.51) | 0.32  (0.07, 0.54) | 0.38  (0.09, 0.64) | 0.34  (0.08, 0.57) | 0.21  (-0.05, 0.46) | MAT+MRT |  |  |  |  |
| 0.38  (0.16, 0.62) | 0.41  (0.24, 0.58) | 0.47  (0.24, 0.7) | 0.43  (0.24, 0.62) | 0.31  (0.13, 0.49) | 0.1  (-0.17, 0.38) | MAT+HRT |  |  |  |
| 0.06  (-0.09, 0.22) | 0.1  (-0.08, 0.24) | 0.16  (-0.07, 0.36) | 0.12  (-0.07, 0.28) | 0  (-0.21, 0.17) | -0.22  (-0.47, 0.03) | -0.31  (-0.55, -0.1) | HIIT |  |  |
| -0.02  (-0.21, 0.18) | 0.02  (-0.14, 0.17) | 0.08  (-0.15, 0.29) | 0.04  (-0.14, 0.21) | -0.08  (-0.28, 0.1) | -0.3  (-0.56, -0.04) | -0.39  (-0.62, -0.17) | -0.08  (-0.27, 0.12) | TCEs |  |
| -0.23  (-0.38, -0.07) | -0.2  (-0.28, -0.13) | -0.14  (-0.31, 0.02) | -0.18  (-0.28, -0.08) | -0.3  (-0.43, -0.18) | -0.51  (-0.73, -0.29) | -0.61  (-0.79, -0.44) | -0.29  (-0.44, -0.14) | -0.22  (-0.36, -0.07) | C |

(F1) Network map of HDL


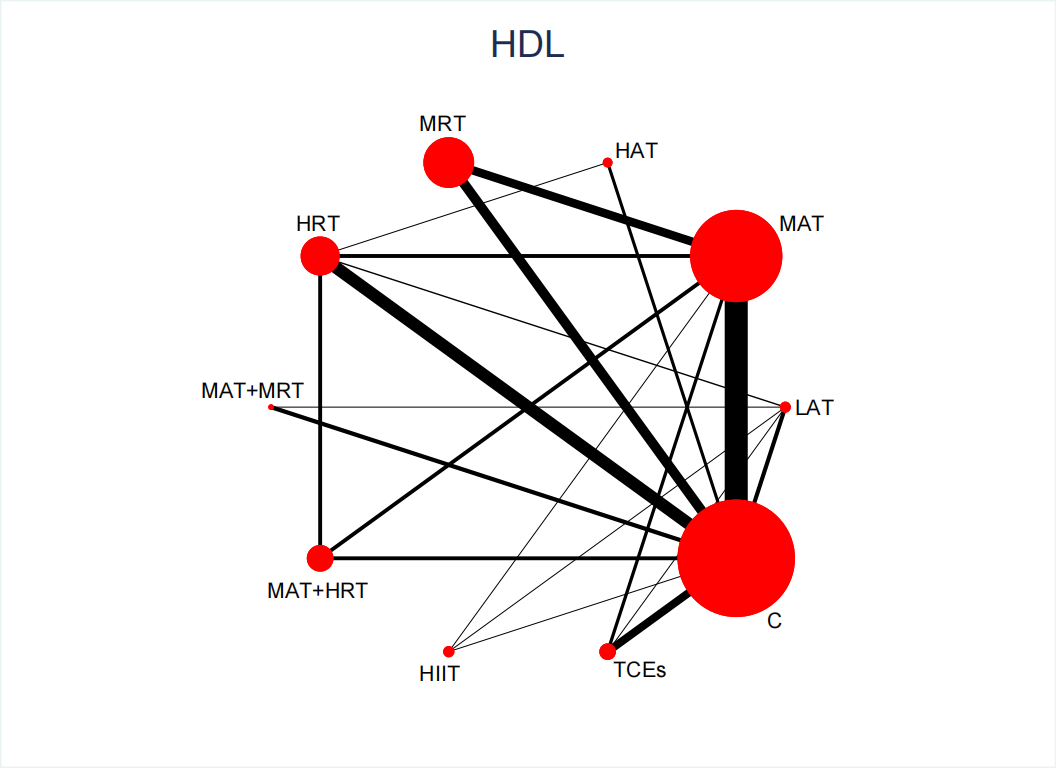


(F2) Forest plot of HDL


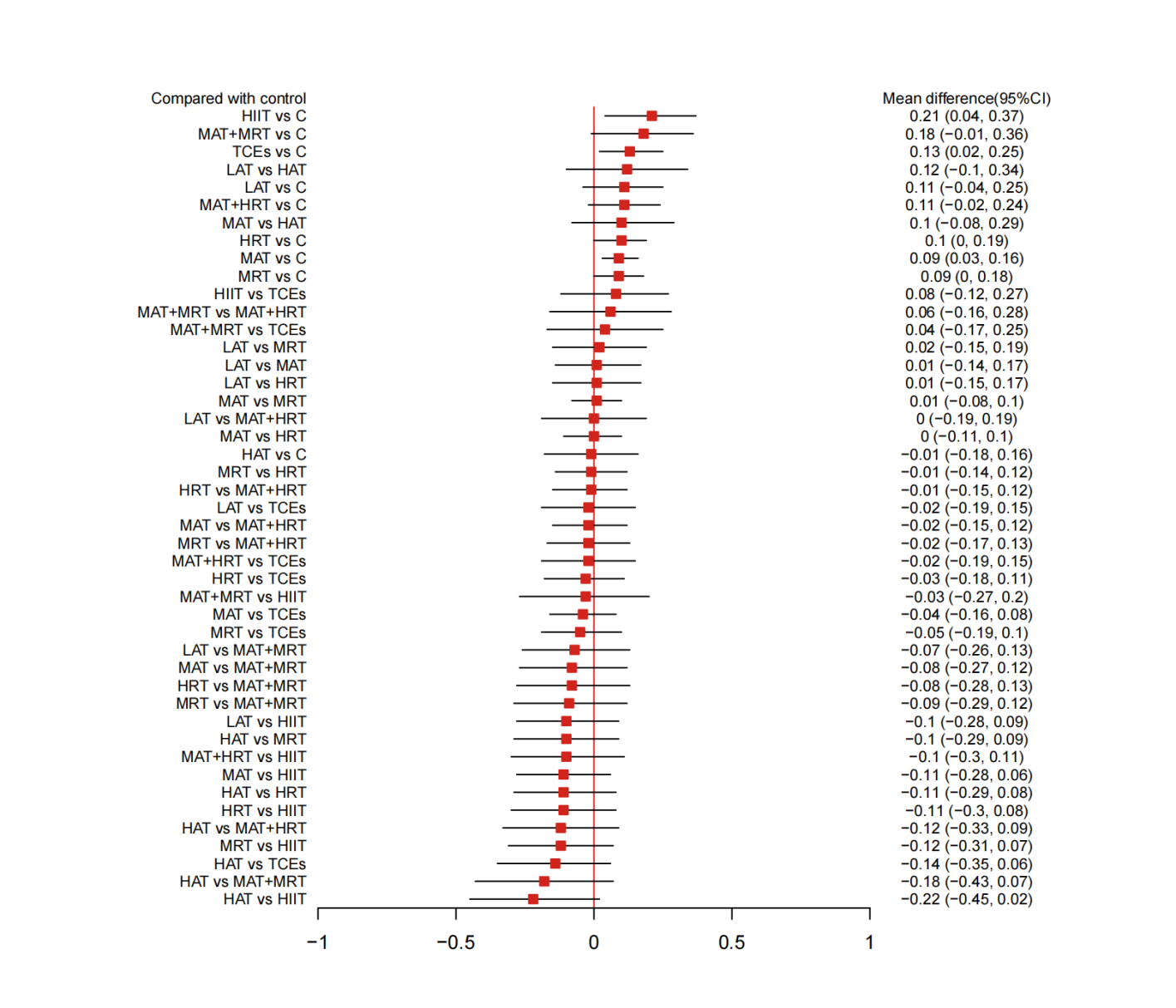


(F3) League table of HDL

| LAT |  |  |  |  |  |  |  |  |  |
| --- | --- | --- | --- | --- | --- | --- | --- | --- | --- |
| 0.01  (-0.14, 0.17) | MAT |  |  |  |  |  |  |  |  |
| 0.12  (-0.1, 0.34) | 0.1  (-0.08, 0.29) | HAT |  |  |  |  |  |  |  |
| 0.02  (-0.15, 0.19) | 0.01  (-0.08, 0.1) | -0.1  (-0.29, 0.09) | MRT |  |  |  |  |  |  |
| 0.01  (-0.15, 0.17) | 0  (-0.11, 0.1) | -0.11  (-0.29, 0.08) | -0.01  (-0.14, 0.12) | HRT |  |  |  |  |  |
| -0.07  (-0.26, 0.13) | -0.08  (-0.27, 0.12) | -0.18  (-0.43, 0.07) | -0.09  (-0.29, 0.12) | -0.08  (-0.28, 0.13) | MAT+MRT |  |  |  |  |
| 0  (-0.19, 0.19) | -0.02  (-0.15, 0.12) | -0.12  (-0.33, 0.09) | -0.02  (-0.17, 0.13) | -0.01  (-0.15, 0.12) | 0.06  (-0.16, 0.28) | MAT+HRT |  |  |  |
| -0.1  (-0.28, 0.09) | -0.11  (-0.28, 0.06) | -0.22  (-0.45, 0.02) | -0.12  (-0.31, 0.07) | -0.11  (-0.3, 0.08) | -0.03  (-0.27, 0.2) | -0.1  (-0.3, 0.11) | HIIT |  |  |
| -0.02  (-0.19, 0.15) | -0.04  (-0.16, 0.08) | -0.14  (-0.35, 0.06) | -0.05  (-0.19, 0.1) | -0.03  (-0.18, 0.11) | 0.04  (-0.17, 0.25) | -0.02  (-0.19, 0.15) | 0.08  (-0.12, 0.27) | TCEs |  |
| 0.11  (-0.04, 0.25) | 0.09  (0.03, 0.16) | -0.01  (-0.18, 0.16) | 0.09  (0, 0.18) | 0.1  (0, 0.19) | 0.18  (-0.01, 0.36) | 0.11  (-0.02, 0.24) | 0.21  (0.04, 0.37) | 0.13  (0.02, 0.25) | C |

(G1) Network map of LDL


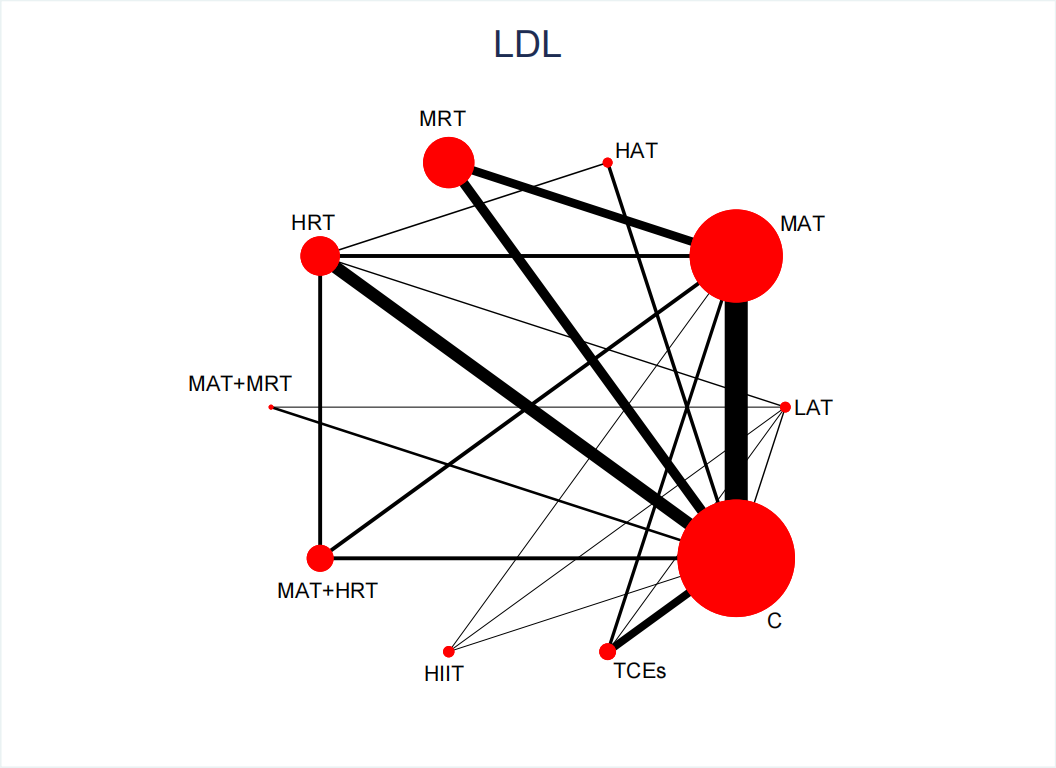


(G2) Forest plot of LDL


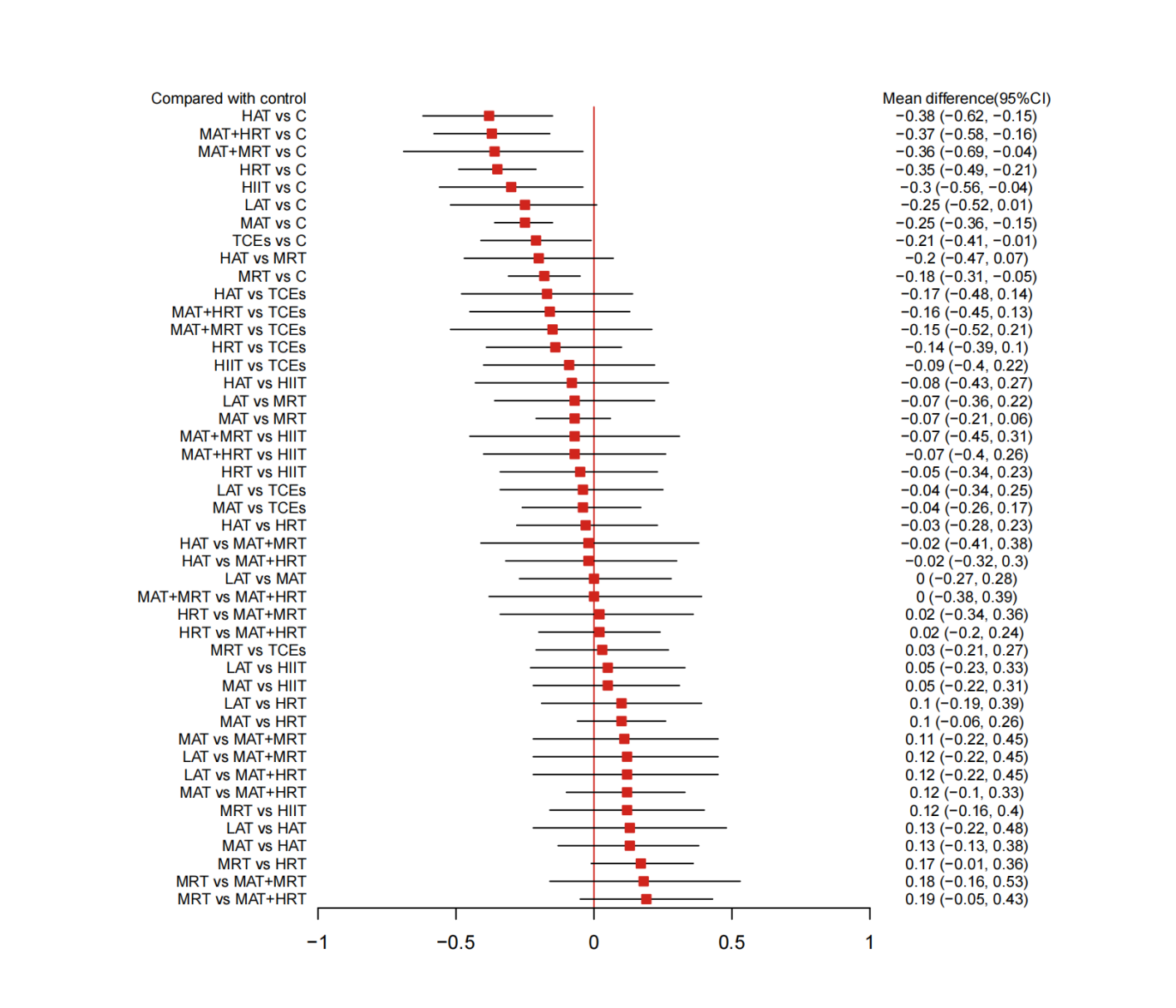


(G3) League table of LDL

| LAT |  |  |  |  |  |  |  |  |  |
| --- | --- | --- | --- | --- | --- | --- | --- | --- | --- |
| 0  (-0.27, 0.28) | MAT |  |  |  |  |  |  |  |  |
| 0.13  (-0.22, 0.48) | 0.13  (-0.13, 0.38) | HAT |  |  |  |  |  |  |  |
| -0.07  (-0.36, 0.22) | -0.07  (-0.21, 0.06) | -0.2  (-0.47, 0.07) | MRT |  |  |  |  |  |  |
| 0.1  (-0.19, 0.39) | 0.1  (-0.06, 0.26) | -0.03  (-0.28, 0.23) | 0.17  (-0.01, 0.36) | HRT |  |  |  |  |  |
| 0.12  (-0.22, 0.45) | 0.11  (-0.22, 0.45) | -0.02  (-0.41, 0.38) | 0.18  (-0.16, 0.53) | 0.02  (-0.34, 0.36) | MAT+MRT |  |  |  |  |
| 0.12  (-0.22, 0.45) | 0.12  (-0.1, 0.33) | -0.02  (-0.32, 0.3) | 0.19  (-0.05, 0.43) | 0.02  (-0.2, 0.24) | 0  (-0.38, 0.39) | MAT+HRT |  |  |  |
| 0.05  (-0.23, 0.33) | 0.05  (-0.22, 0.31) | -0.08  (-0.43, 0.27) | 0.12  (-0.16, 0.4) | -0.05  (-0.34, 0.23) | -0.07  (-0.45, 0.31) | -0.07  (-0.4, 0.26) | HIIT |  |  |
| -0.04  (-0.34, 0.25) | -0.04  (-0.26, 0.17) | -0.17  (-0.48, 0.14) | 0.03  (-0.21, 0.27) | -0.14  (-0.39, 0.1) | -0.15  (-0.52, 0.21) | -0.16  (-0.45, 0.13) | -0.09  (-0.4, 0.22) | TCEs |  |
| -0.25  (-0.52, 0.01) | -0.25  (-0.36, -0.15) | -0.38  (-0.62, -0.15) | -0.18  (-0.31, -0.05) | -0.35  (-0.49, -0.21) | -0.36  (-0.69, -0.04) | -0.37  (-0.58, -0.16) | -0.3  (-0.56, -0.04) | -0.21  (-0.41, -0.01) | C |

(H1) Network map of BMI


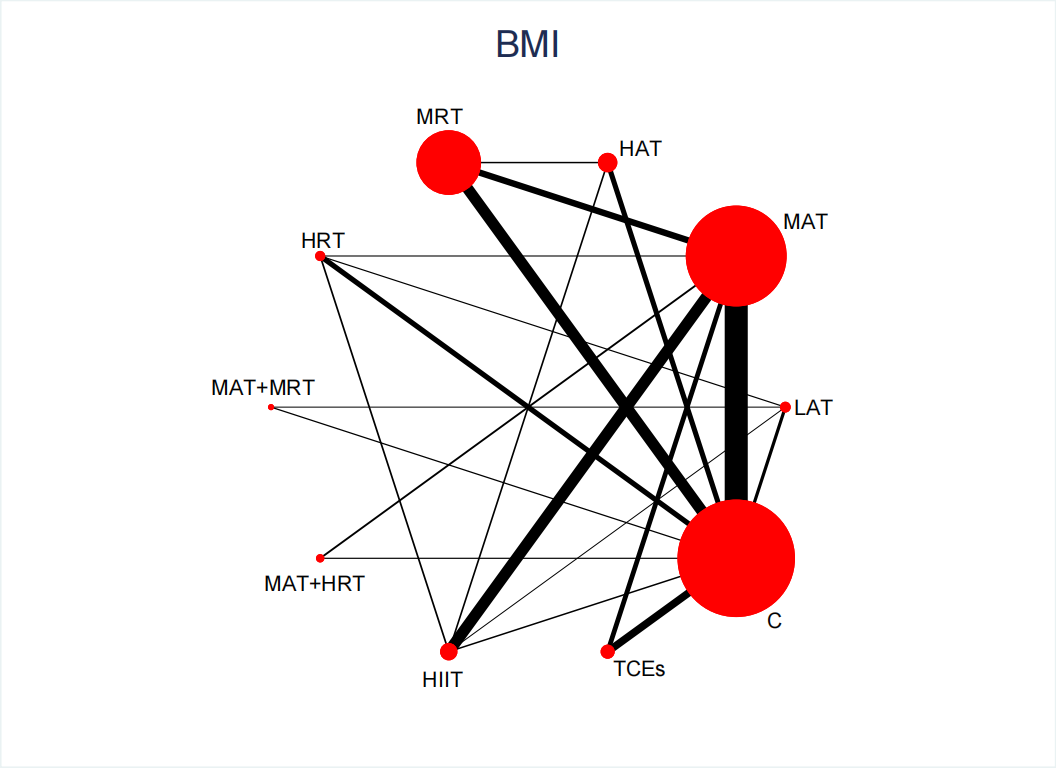


(H2) Forest plot of BMI


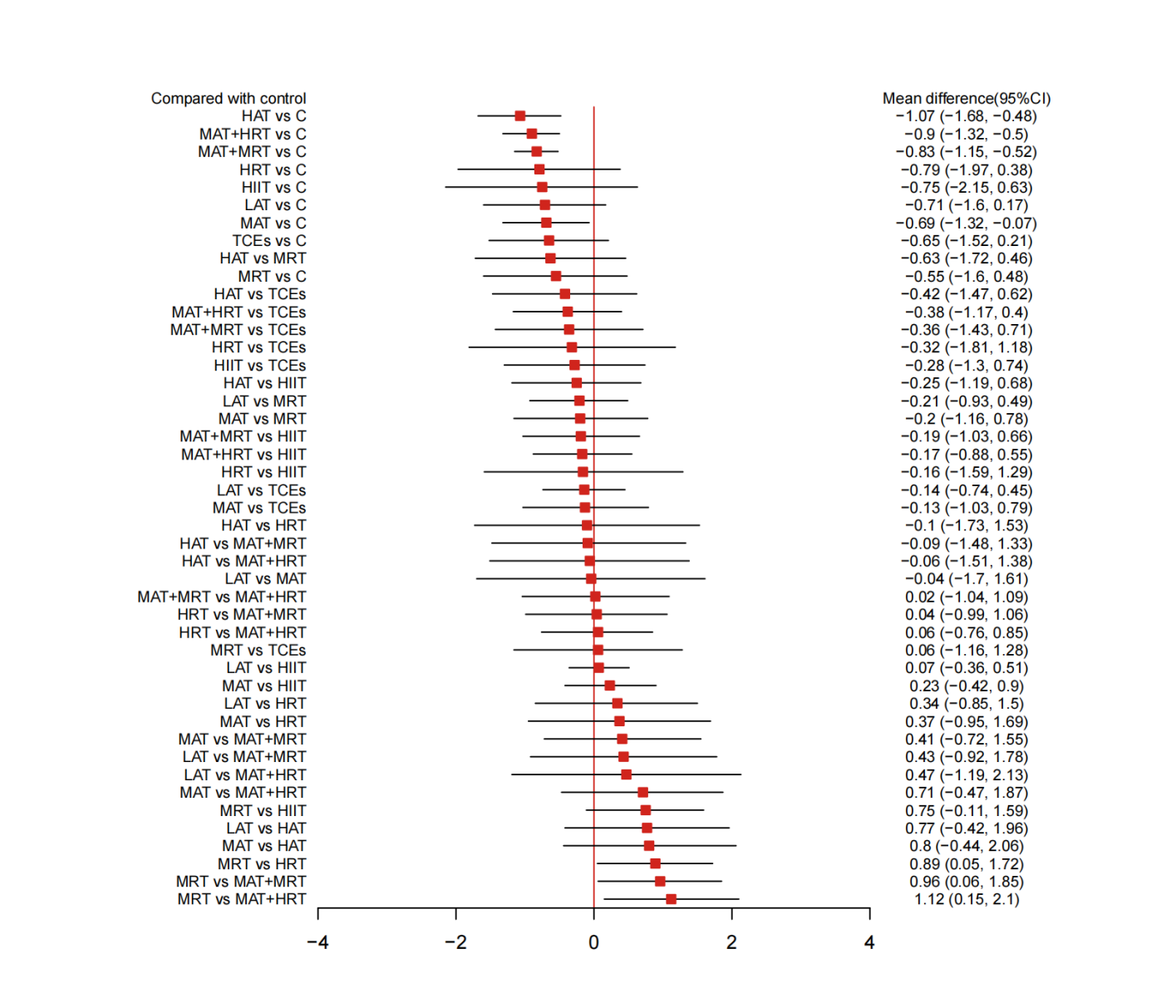


(H3) League table of BMI

| LAT |  |  |  |  |  |  |  |  |  |
| --- | --- | --- | --- | --- | --- | --- | --- | --- | --- |
| 0.89  (0.05, 1.72) | MAT |  |  |  |  |  |  |  |  |
| 1.12  (0.15, 2.1) | 0.23  (-0.42, 0.9) | HAT |  |  |  |  |  |  |  |
| 0.96  (0.06, 1.85) | 0.07  (-0.36, 0.51) | -0.17  (-0.88, 0.55) | MRT |  |  |  |  |  |  |
| 0.34  (-0.85, 1.5) | -0.55  (-1.6, 0.48) | -0.79  (-1.97, 0.38) | -0.63  (-1.72, 0.46) | HRT |  |  |  |  |  |
| 0.8  (-0.44, 2.06) | -0.09  (-1.48, 1.33) | -0.32  (-1.81, 1.18) | -0.16  (-1.59, 1.29) | 0.47  (-1.19, 2.13) | MAT+MRT |  |  |  |  |
| 0.71  (-0.47, 1.87) | -0.19  (-1.03, 0.66) | -0.42  (-1.47, 0.62) | -0.25  (-1.19, 0.68) | 0.37  (-0.95, 1.69) | -0.1  (-1.73, 1.53) | MAT+HRT |  |  |  |
| 0.75  (-0.11, 1.59) | -0.14  (-0.74, 0.45) | -0.38  (-1.17, 0.4) | -0.21  (-0.93, 0.49) | 0.41  (-0.72, 1.55) | -0.06  (-1.51, 1.38) | 0.04  (-0.99, 1.06) | HIIT |  |  |
| 0.77  (-0.42, 1.96) | -0.13  (-1.03, 0.79) | -0.36  (-1.43, 0.71) | -0.2  (-1.16, 0.78) | 0.43  (-0.92, 1.78) | -0.04  (-1.7, 1.61) | 0.06  (-1.16, 1.28) | 0.02  (-1.04, 1.09) | TCEs |  |
| 0.06  (-0.76, 0.85) | -0.83  (-1.15, -0.52) | -1.07  (-1.68, -0.48) | -0.9  (-1.32, -0.5) | -0.28  (-1.3, 0.74) | -0.75  (-2.15, 0.63) | -0.65  (-1.52, 0.21) | -0.69  (-1.32, -0.07) | -0.71  (-1.6, 0.17) | C |

(I1) Network map of BW


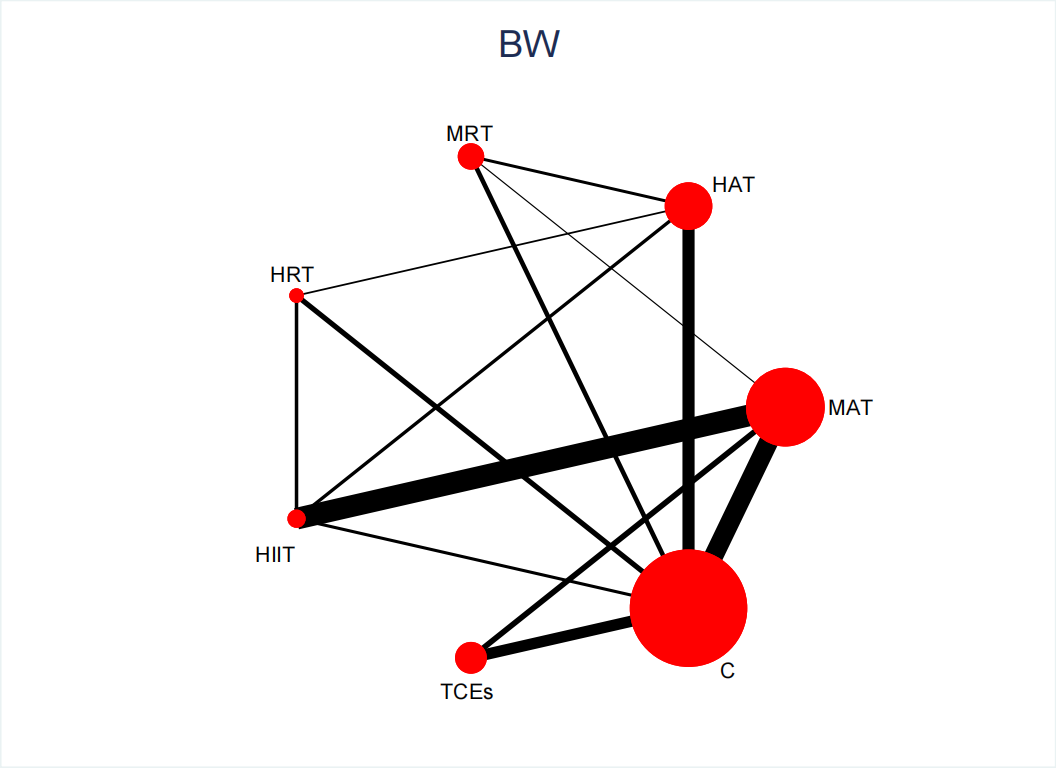


(I2) Forest plot of BW


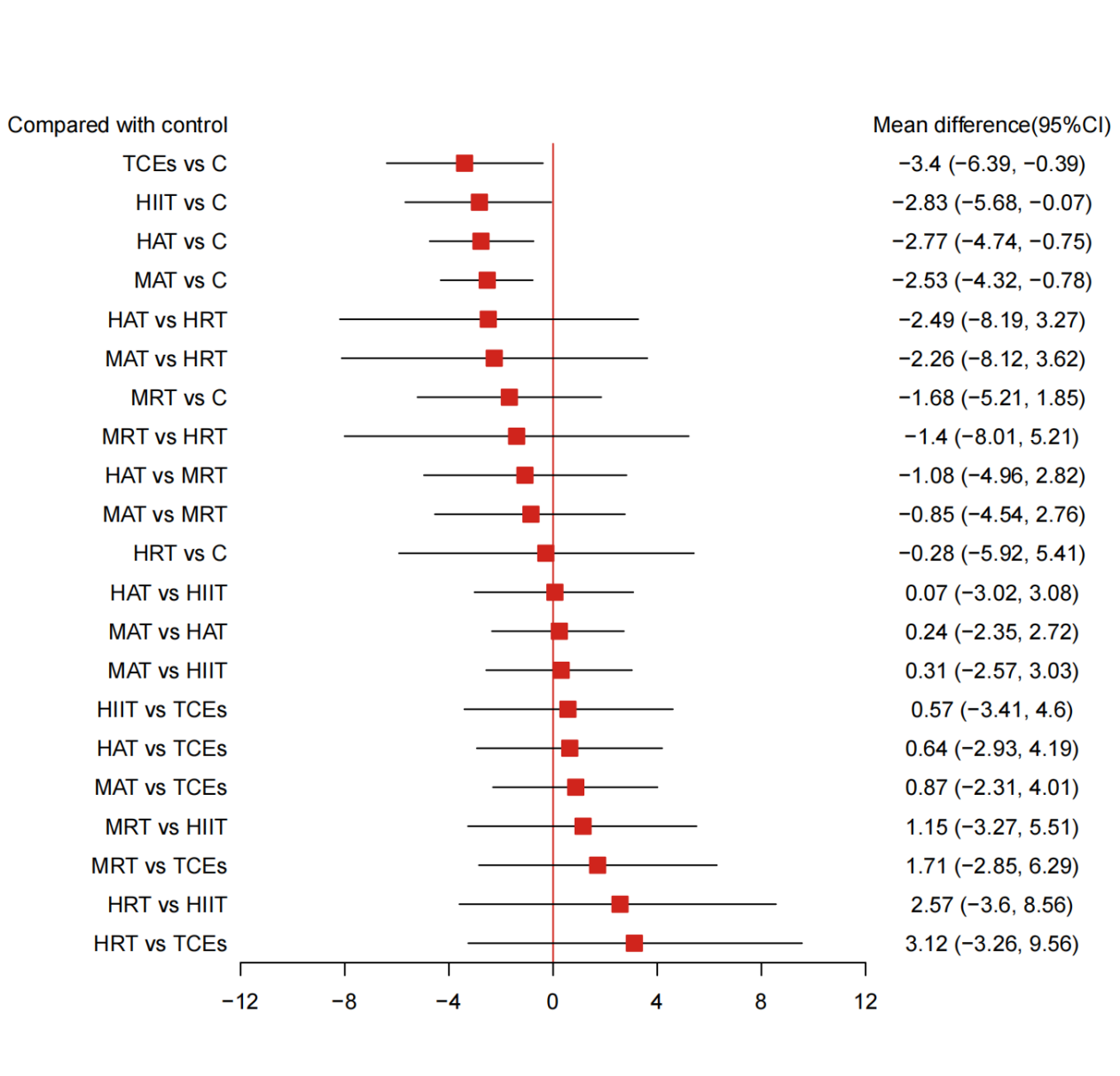


(I3) League table of BW

| MAT |  |  |  |  |  |  |
| --- | --- | --- | --- | --- | --- | --- |
| 0.24  (-2.35, 2.72) | HAT |  |  |  |  |  |
| -0.85  (-4.54, 2.76) | -1.08  (-4.96, 2.82) | MRT |  |  |  |  |
| -2.26  (-8.12, 3.62) | -2.49  (-8.19, 3.27) | -1.4  (-8.01, 5.21) | HRT |  |  |  |
| 0.31  (-2.57, 3.03) | 0.07  (-3.02, 3.08) | 1.15  (-3.27, 5.51) | 2.57  (-3.6, 8.56) | HIIT |  |  |
| 0.87  (-2.31, 4.01) | 0.64  (-2.93, 4.19) | 1.71  (-2.85, 6.29) | 3.12  (-3.26, 9.56) | 0.57  (-3.41, 4.6) | TCEs |  |
| -2.53  (-4.32, -0.78) | -2.77  (-4.74, -0.75) | -1.68  (-5.21, 1.85) | -0.28  (-5.92, 5.41) | -2.83  (-5.68, -0.07) | -3.4  (-6.39, -0.39) | C |

(J1) Network map of WC


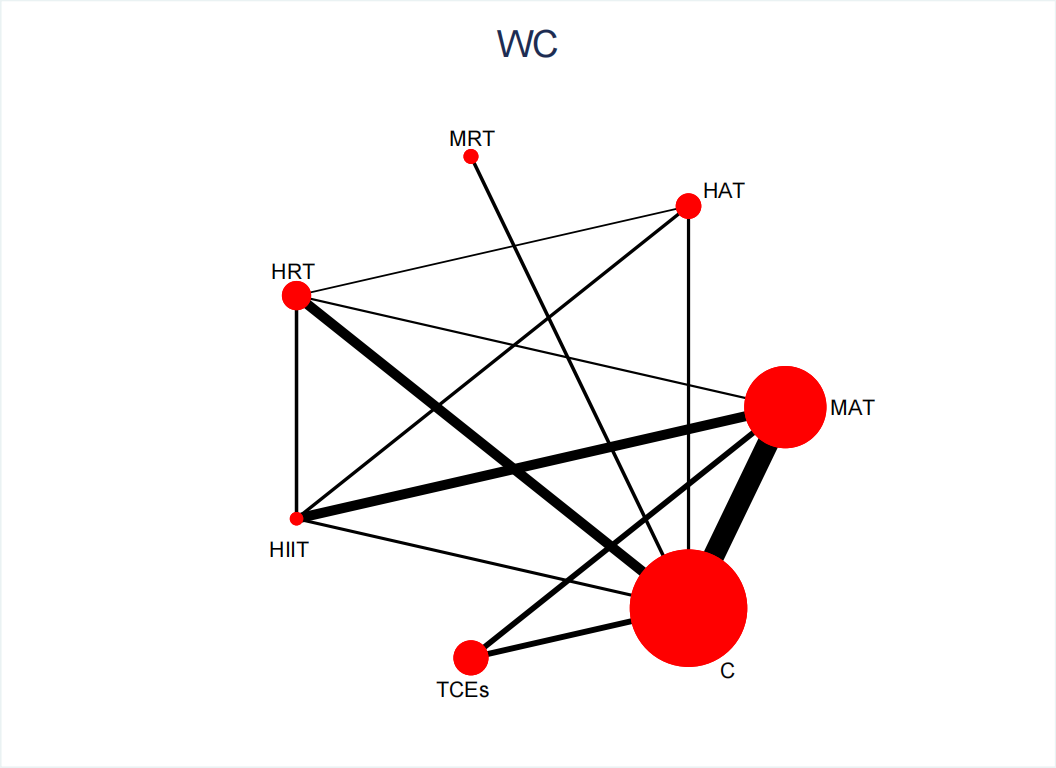


(J2) Forest plot of WC


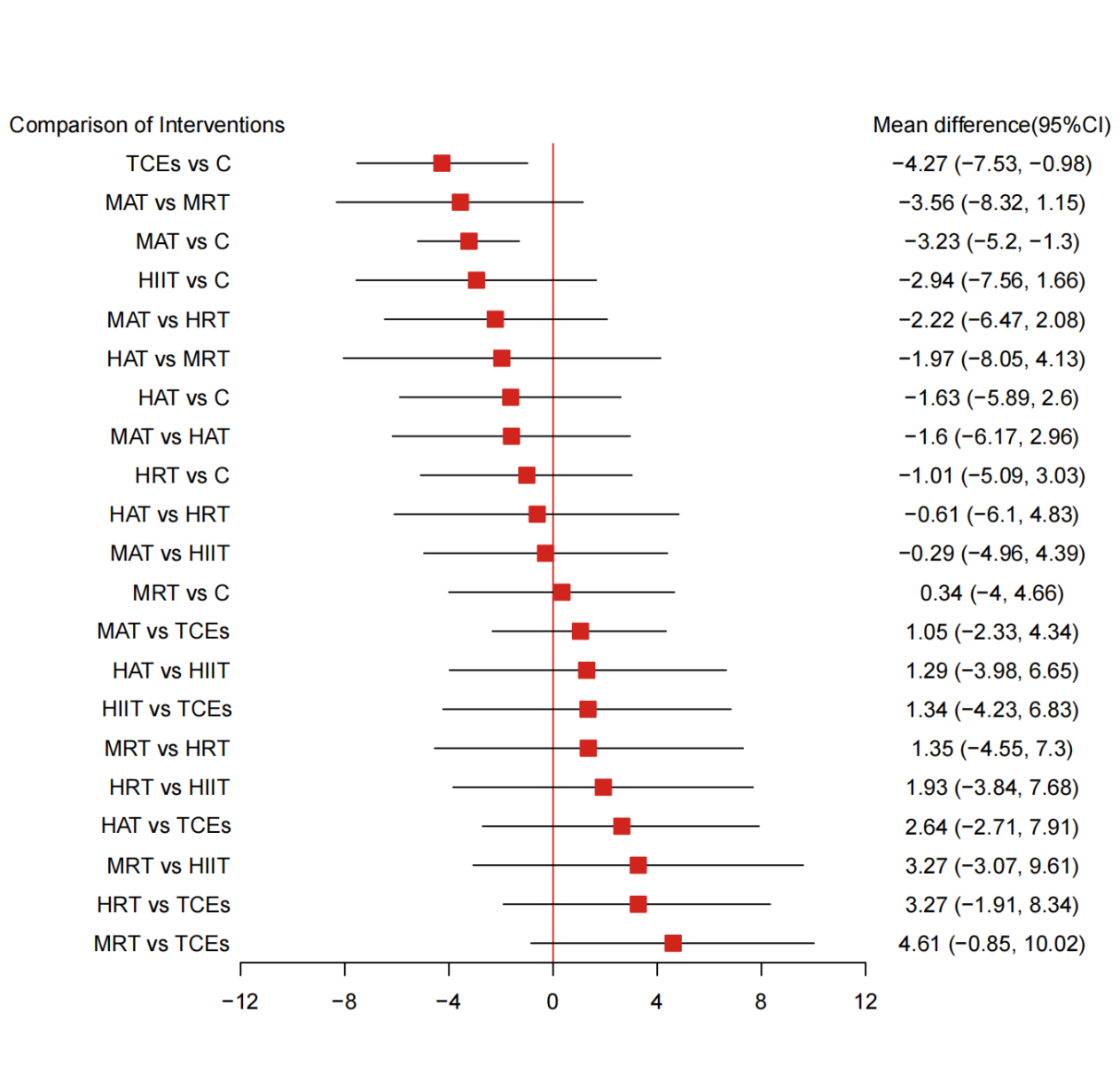


(J3) League table of BW

| MAT |  |  |  |  |  |  |
| --- | --- | --- | --- | --- | --- | --- |
| -1.6  (-6.17, 2.96) | HAT |  |  |  |  |  |
| -3.56  (-8.32, 1.15) | -1.97  (-8.05, 4.13) | MRT |  |  |  |  |
| -2.22  (-6.47, 2.08) | -0.61  (-6.1, 4.83) | 1.35  (-4.55, 7.3) | HRT |  |  |  |
| -0.29  (-4.96, 4.39) | 1.29  (-3.98, 6.65) | 3.27  (-3.07, 9.61) | 1.93  (-3.84, 7.68) | HIIT |  |  |
| 1.05  (-2.33, 4.34) | 2.64  (-2.71, 7.91) | 4.61  (-0.85, 10.02) | 3.27  (-1.91, 8.34) | 1.34  (-4.23, 6.83) | TCEs |  |
| -3.23  (-5.2, -1.3) | -1.63  (-5.89, 2.6) | 0.34  (-4, 4.66) | -1.01  (-5.09, 3.03) | -2.94  (-7.56, 1.66) | -4.27  (-7.53, -0.98) | C |

# Appendix12: Network Meta-Regression--changes in heterogeneity

**Table S12.1 Outcome of Network Meta-Regression**

| Covariate | HbA1c | FBG | 2hPG | TC | TG |
| --- | --- | --- | --- | --- | --- |
| Publish Year | -0.079546  (-0.16913,0.008694) | -0.28917*  (-0.4081,-0.17121) | -0.03629 (-0.31222,0.24131) | -0.184865 (-0.32821,0.04846) | -0.042202 (-0.149264,0.07050) |
| Sample Size | 0.004241 (-0.08262,0.09201) | -0.09899  (-0.2112,0.0113) | 0.1116 (-0.15543,0.38595) | 0.01204 (-0.11072,0.13374) | 0.07143 (-0.02373,0.17160) |
| Mean Age | -0.048357 (-0.15165,0.05621) | 0.087051  (-0.03756,0.21076) | 0.0755 (-0.33990,0.19195) | 0.0143 (-0.11936,0.15158) | -0.032219 (-0.130303,0.06964) |
| Percentage of Male | 0.062313 (-0.03288,0.15629) | -0.08811  (-0.2179,0.03993) | 0.07168 (-0.22840,0.37126) | 0.09097 (-0.05329,0.232340) | 0.111603 (-0.001496,0.22674) |
| BMI | -0.007046 (-0.10810,0.09065) | 0.24153*  (0.07313,0.412875) | 0.59671* (0.2158,0.98278) | 0.15377* (0.033182,0.27467) | 0.03981 (-0.051205,0.13597) |
| Exercise period | -0.0406365 (-0.13890,0.05701) | -0.07736  (-0.1995,0.04623) | 0.1979 (-0.08782,0.48326) | -0.15754 (-0.33149,0.01399) | 0.037721 (-0.049069,0.12079) |
| Exercise Frequency | 0.056157 (-0.04146,0.15314) | 0.0187  (-0.1164,0.1521) | -0.3635* (-0.63434,0.09096) | 0.05768 (-0.09275,0.21146) | 0.074113 (-0.032814,0.17997) |
| Time per Session | -0.0207151 (-0.12080,0.07828) | 0.10325  (-0.02084,0.22724) | 0.0171 (-0.28892,0.32387) | -0.06569 (-0.18530,0.05509) | -0.035548 (-0.13827,0.06324) |
| Time per Week | 0.055027 (-0.04449,0.15436) | 0.09499  (-0.03563,0.22541) | -0.1746 (-0.47334,0.12642) | 0.02134 (-0.14023,0.19283) | 0.081182 (-0.052965,0.21281) |

| Covariate | HDL | LDL | BMI | BW | WC |
| --- | --- | --- | --- | --- | --- |
| Publish Year | 0.01931 (-0.07971,0.11927) | -0.04636 (-0.21174,0.12132) | -0.45626 (-0.9059,0.009151) | -2.00929 (-4.0858,0.01289) | -1.1200 (-3.03815,0.6108) |
| Sample Size | -0.036153 (-0.12974,0.06025) | 0.07157 (-0.06707,0.2121) | -0.13355 (-0.5850,0.3239) | -0.2018 (-2.2283,1.956) | 0.02722 (-1.9834,1.965) |
| Mean Age | -0.05427 (-0.15071,0.04300) | 0.06284 (-0.08481,0.2139) | 0.240285 (-0.4376,0.9150) | 0.4775 (-2.2440,3.174) | 0.7674 (-1.19361,2.784) |
| Percentage of Male | -0.040139 (-0.15772,0.07660) | 0.14349* (0.01041,0.2805) | -0.566 (-1.2236,0.09592） | -3.2008* (-5.45506,-0.6757) | -0.2405 (-3.75998,3.263) |
| BMI | -0.434674 (-0.9560,0.1040) | 0.07898 (-0.07427,0.2313) | -0.5637 (-1.2109,0.09897) | -0.1877 (-3.0310,2.836) | 1.0354 (-0.89382,3.156) |
| Exercise period | 0.053189 (-0.04415,0.15063) | -0.002909 (-0.13112,0.12234) | -0.17674 (-0.7336,0.3706) | -0.9068 (-3.4055,1.450) | -0.4646 (-2.51760,1.455) |
| Exercise Frequency | -0.054751 (-0.15054,0.04152) | 0.13225 (-0.01261,0.2842) | 0.335911 (-0.2826,0.9461) | 1.9602 (-1.0527,4.737) | 1.3225 (-0.66935,3.359) |
| Time per Session | -0.028766 (-0.12949,0.07407) | -0.10869 (-0.23390,0.01584) | -0.08649 (-0.8101,0.6060) | -0.3815 (-3.2404,2.453) | 0.6489 (-2.01107,3.372) |
| Time per Week | -0.083679 (-0.18334,0.01755) | 0.08465 (-0.08402,0.26510) | 0.294821 (-0.5349,1.0872) | 2.2138 (-1.2600,5.446) | 2.2765 (-0.22112,4.799) |

**12.2** HbA1c

**12.2.1** Publication year

When the model was adjusted for centering value of publish year 2018, the hierarchy from the unadjusted model retained.

**Figure S12.2.1** presents the impact of various exercise interventions on overall symptom changes after adjusting for the publication year 2018. It compares and ranks different exercise types against the control group (CON) based on the mean difference (MD). The interventions crossing the y-axis show no significant difference compared to the control group.


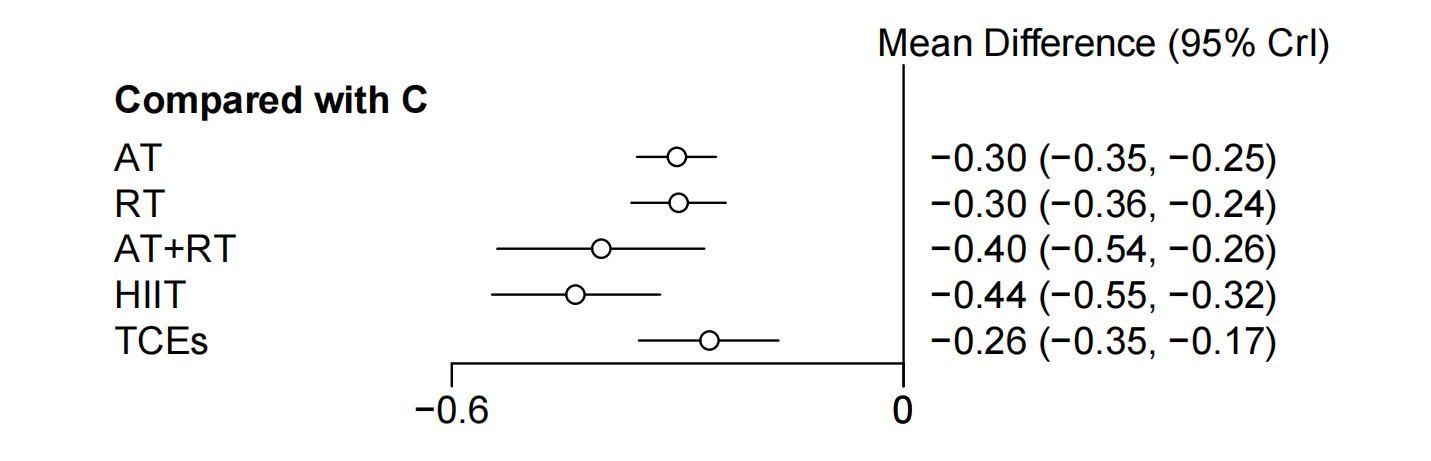


**12.2.2** Sample size
When the model was adjusted for centering value of sample size 32, the hierarchy from the unadjusted model retained.

**Figure S12.2.2** presents the impact of various exercise interventions on overall symptom changes after adjusting for the sample size 32. It compares and ranks different exercise types against the control group (CON) based on the mean difference (MD). The interventions crossing the y-axis show no significant difference compared to the control group.


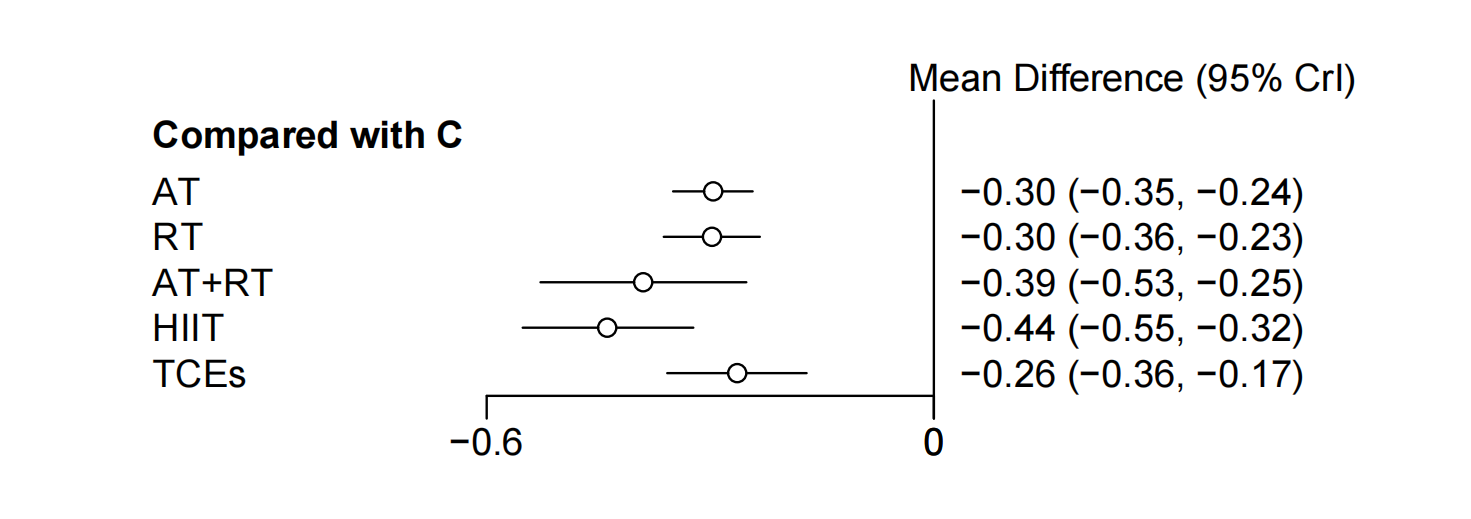


**12.2.3** Mean age
When the model was adjusted for centering value of mean age 57, the hierarchy from the unadjusted model retained.

**Figure S12.2.3** presents the impact of various exercise interventions on overall symptom changes after adjusting for the mean age of 57. It compares and ranks different exercise types against the control group (CON) based on the mean difference (MD). The interventions crossing the y-axis show no significant difference compared to the control group.


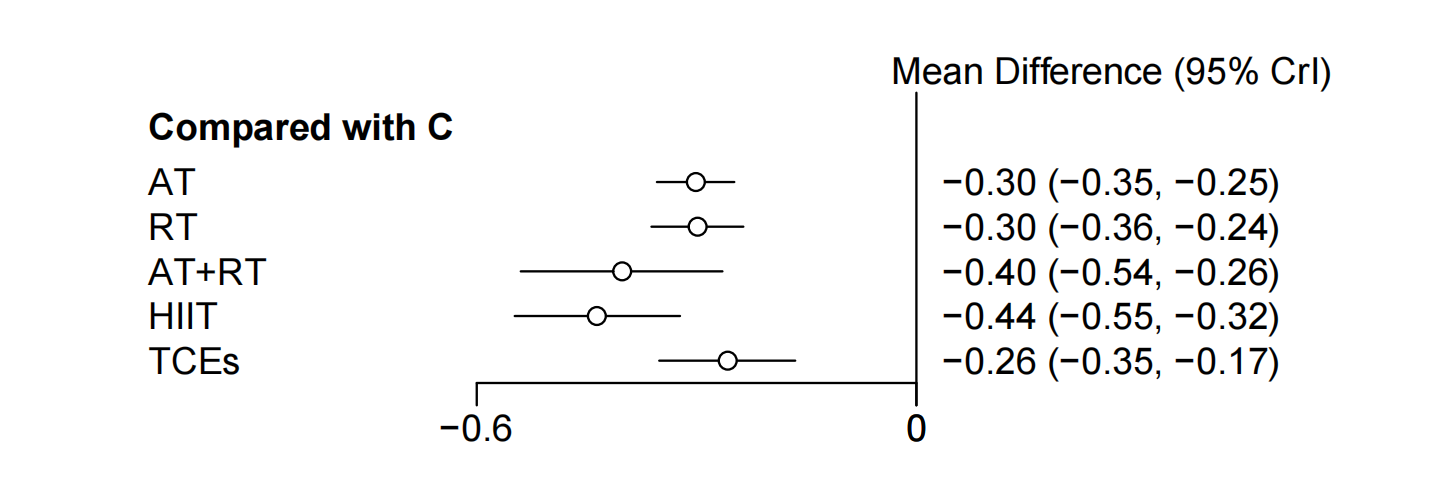


**12.2.4** Percentage of Male

When the model was adjusted for centering value of male’s percentage 46%, the hierarchy from the unadjusted model retained.

**Figure S12.2.4** presents the impact of various exercise interventions on overall symptom changes after adjusting for the male’s percentage of 46%. It compares and ranks different exercise types against the control group (CON) based on the mean difference (MD). The interventions crossing the y-axis show no significant difference compared to the control group.


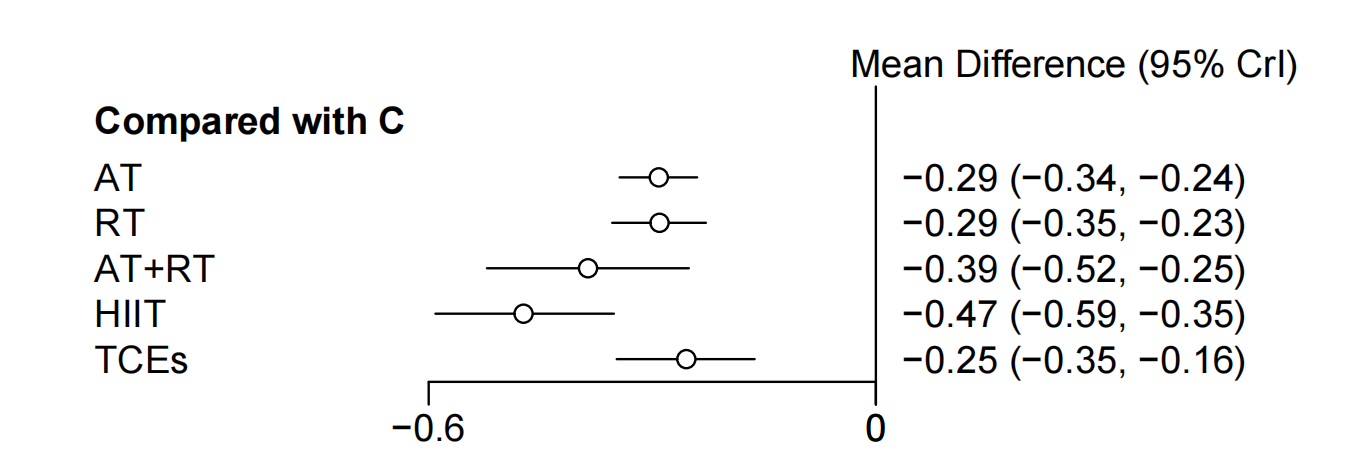


**12.2.5** BMI
When the model was adjusted for centering value of BMI 25, the hierarchy from the unadjusted model retained.

**Figure S12.2.5** presents the impact of various exercise interventions on overall symptom changes after adjusting for BMI 25. It compares and ranks different exercise types against the control group (CON) based on the mean difference (MD). The interventions crossing the y-axis show no significant difference compared to the control group.


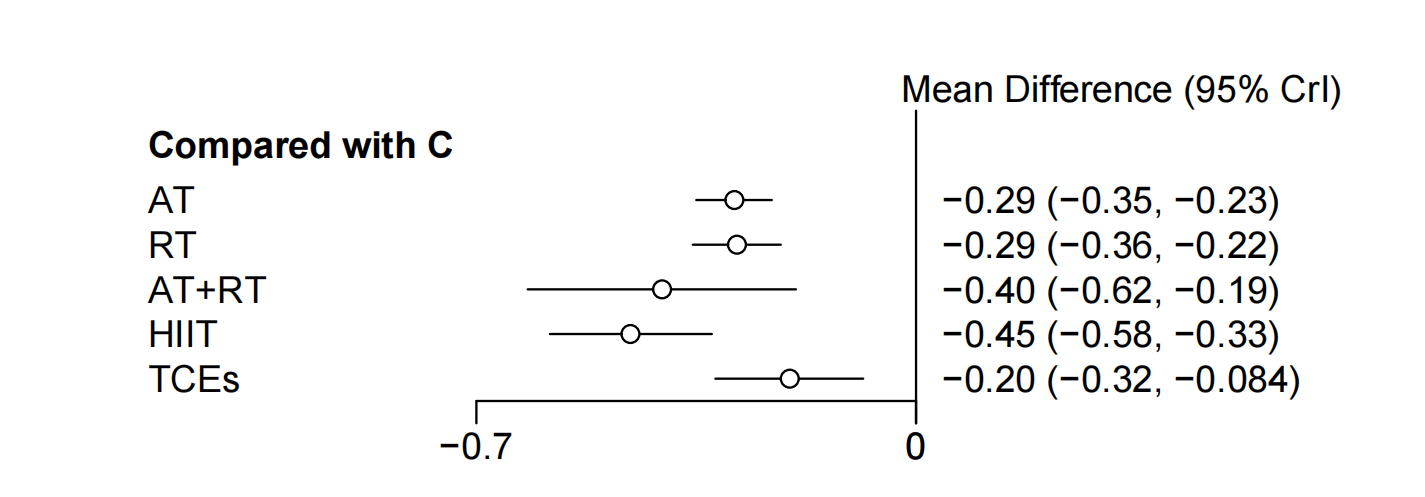


**12.2.6** Exercise period
When the model was adjusted for centering value of exercise period 23 weeks, the hierarchy from the unadjusted model retained.

**Figure S12.2.6** presents the impact of various exercise interventions on overall symptom changes after adjusting for the exercise period of 23 weeks. It compares and ranks different exercise types against the control group (CON) based on the mean difference (MD). The interventions crossing the y-axis show no significant difference compared to the control group.


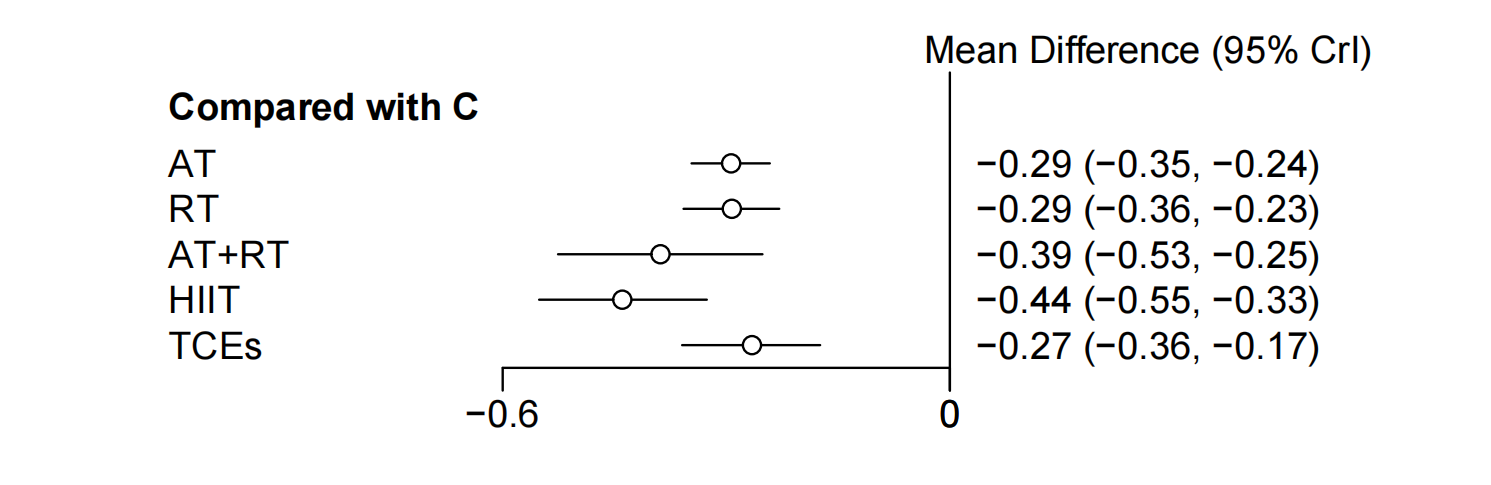


**12.2.7** Exercise frequency
When the model was adjusted for centering value of exercise frequency (3 times per week), the hierarchy from the unadjusted model retained.
**Figure S12.2.7** presents the impact of various exercise interventions on overall symptom changes after adjusting for the exercise frequency of 3 times per week. It compares and ranks different exercise types against the control group (CON) based on the mean difference (MD). The interventions crossing the y-axis show no significant difference compared to the control group.


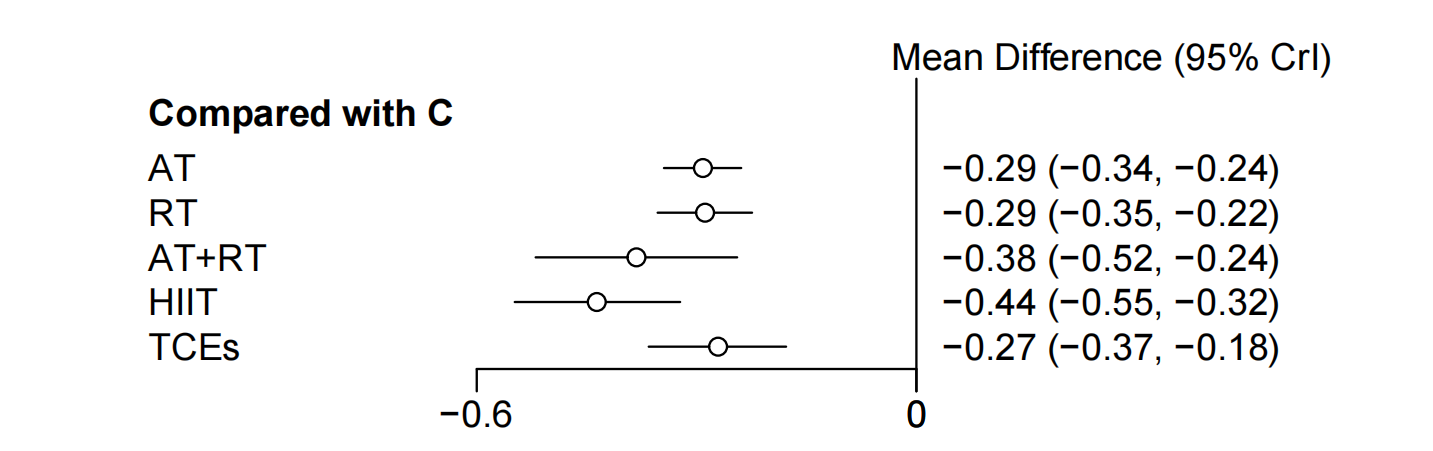


**12.2.8** Time per session
When the model was adjusted for centering value of time per session (44 minutes), the hierarchy from the unadjusted model retained.
**Figure S12.2.8** presents the impact of various exercise interventions on overall symptom changes after adjusting for the time per session of 44 minutes. It compares and ranks different exercise types against the control group (CON) based on the mean difference (MD). The interventions crossing the y-axis show no significant difference compared to the control group.


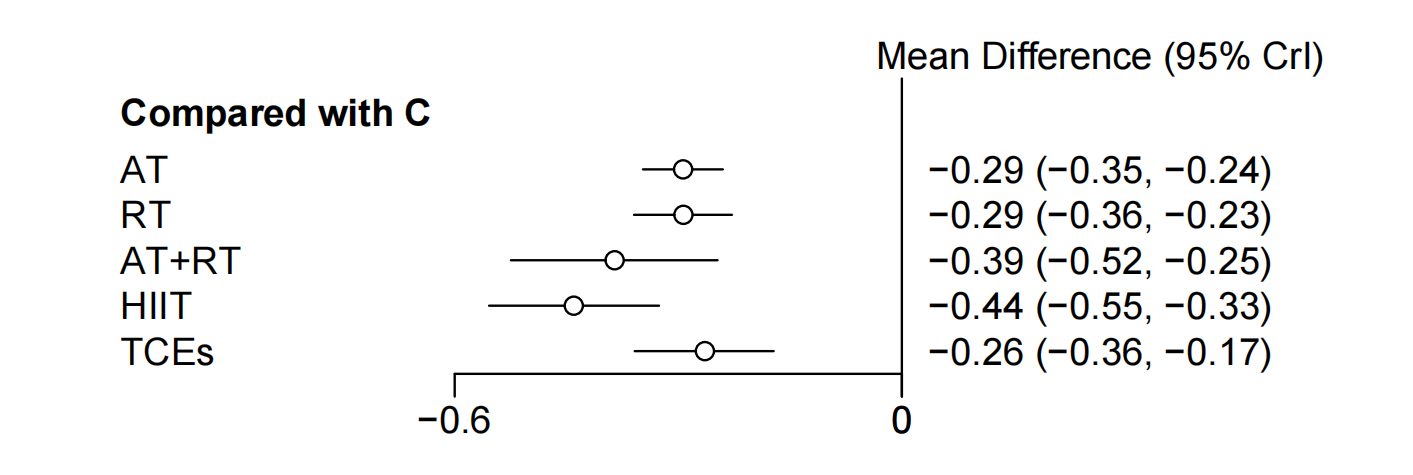


**12.2.9** Time per week
When the model was adjusted for centering value of time per week (160 minutes), the hierarchy from the unadjusted model retained.
**Figure S12.2.9** presents the impact of various exercise interventions on overall symptom changes after adjusting for the time per week of 160 minutes. It compares and ranks different exercise types against the control group (CON) based on the mean difference (MD). The interventions crossing the y-axis show no significant difference compared to the control group.


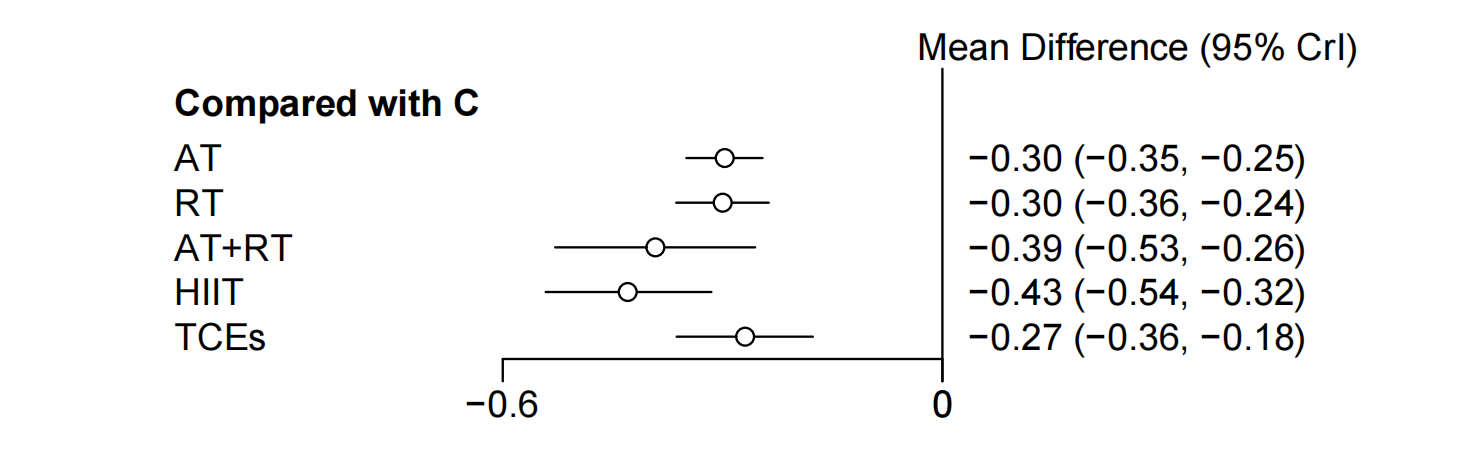


**12.3** FBG

**12.3.1** Publication year

When the model was adjusted for centering value of publish year 2017, the hierarchy from the unadjusted model retained.

**Figure S12.3.1** presents the impact of various exercise interventions on overall symptom changes after adjusting for the publication year 2017. It compares and ranks different exercise types against the control group (CON) based on the mean difference (MD). The interventions crossing the y-axis show no significant difference compared to the control group.


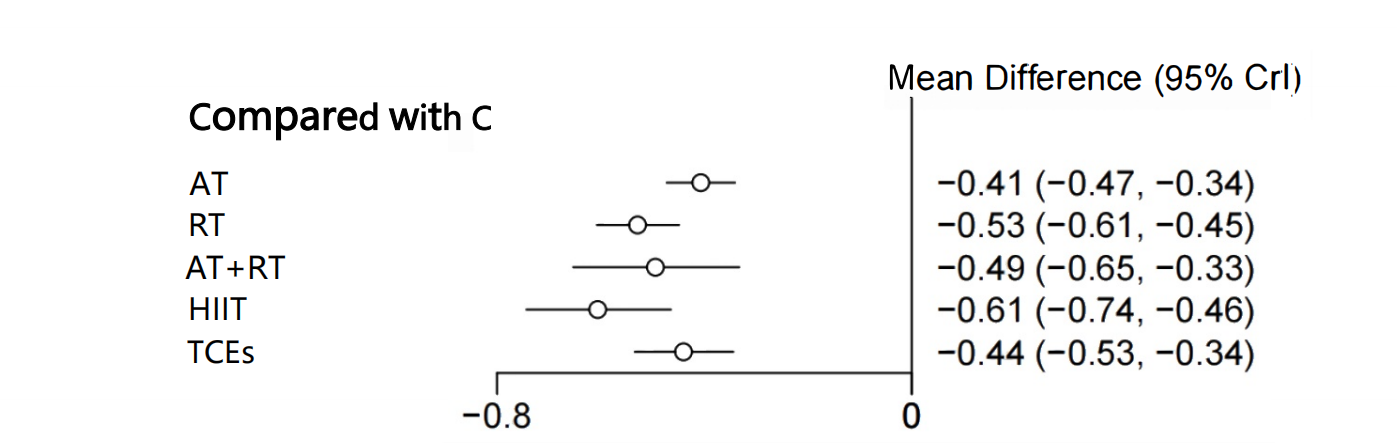


**12.3.2** Sample size
When the model was adjusted for centering value of sample size 32, the hierarchy from the unadjusted model retained.

**Figure S12.3.2** presents the impact of various exercise interventions on overall symptom changes after adjusting for the sample size 32. It compares and ranks different exercise types against the control group (CON) based on the mean difference (MD). The interventions crossing the y-axis show no significant difference compared to the control group.

**12.3.3** Mean age
When the model was adjusted for centering value of mean age 56, the hierarchy from the unadjusted model retained.

**Figure S12.3.3** presents the impact of various exercise interventions on overall symptom changes after adjusting for the mean age of 56. It compares and ranks different exercise types against the control group (CON) based on the mean difference (MD). The interventions crossing the y-axis show no significant difference compared to the control group.

**12.3.4** Percentage of Male

When the model was adjusted for centering value of male’s percentage 42%, the hierarchy from the unadjusted model retained.

**Figure S12.3.4** presents the impact of various exercise interventions on overall symptom changes after adjusting for the male’s percentage of 42%. It compares and ranks different exercise types against the control group (CON) based on the mean difference (MD). The interventions crossing the y-axis show no significant difference compared to the control group.

**12.3.5** BMI
When the model was adjusted for centering value of BMI 27, the hierarchy from the unadjusted model retained.

**Figure S12.3.5** presents the impact of various exercise interventions on overall symptom changes after adjusting for BMI 27. It compares and ranks different exercise types against the control group (CON) based on the mean difference (MD). The interventions crossing the y-axis show no significant difference compared to the control group.

**12.3.6** Exercise period
When the model was adjusted for centering value of exercise period 22 weeks, the hierarchy from the unadjusted model retained.

**Figure S12.3.6** presents the impact of various exercise interventions on overall symptom changes after adjusting for the exercise period of 22 weeks. It compares and ranks different exercise types against the control group (CON) based on the mean difference (MD). The interventions crossing the y-axis show no significant difference compared to the control group.

**12.3.7** Exercise frequency
When the model was adjusted for centering value of exercise frequency (4 times per week), the hierarchy from the unadjusted model retained.
**Figure S12.3.7** presents the impact of various exercise interventions on overall symptom changes after adjusting for the exercise frequency of 4 times per week. It compares and ranks different exercise types against the control group (CON) based on the mean difference (MD). The interventions crossing the y-axis show no significant difference compared to the control group.

**12.3.8** Time per session
When the model was adjusted for centering value of time per session (46 minutes), the hierarchy from the unadjusted model retained.
**Figure S12.3.8** presents the impact of various exercise interventions on overall symptom changes after adjusting for the time per session of 46 minutes. It compares and ranks different exercise types against the control group (CON) based on the mean difference (MD). The interventions crossing the y-axis show no significant difference compared to the control group.

**12.3.9** Time per week
When the model was adjusted for centering value of time per week (172 minutes), the hierarchy from the unadjusted model retained.
**Figure S12.3.9** presents the impact of various exercise interventions on overall symptom changes after adjusting for the time per week of 172 minutes. It compares and ranks different exercise types against the control group (CON) based on the mean difference (MD). The interventions crossing the y-axis show no significant difference compared to the control group.

**12.4** 2hPG

**12.4.1** Publication year

When the model was adjusted for centering value of publish year 2018, the hierarchy from the unadjusted model retained.

**Figure S12.4.1** presents the impact of various exercise interventions on overall symptom changes after adjusting for the publication year 2018. It compares and ranks different exercise types against the control group (CON) based on the mean difference (MD). The interventions crossing the y-axis show no significant difference compared to the control group.

**12.4.2** Sample size
When the model was adjusted for centering value of sample size 29, the hierarchy from the unadjusted model retained.

**Figure S12.4.2** presents the impact of various exercise interventions on overall symptom changes after adjusting for the sample size 29. It compares and ranks different exercise types against the control group (CON) based on the mean difference (MD). The interventions crossing the y-axis show no significant difference compared to the control group.

**12.4.3** Mean age
When the model was adjusted for centering value of mean age 57, the hierarchy from the unadjusted model retained.

**Figure S12.4.3** presents the impact of various exercise interventions on overall symptom changes after adjusting for the mean age of 57. It compares and ranks different exercise types against the control group (CON) based on the mean difference (MD). The interventions crossing the y-axis show no significant difference compared to the control group.

**12.4.4** Percentage of Male

When the model was adjusted for centering value of male’s percentage 45%, the hierarchy from the unadjusted model retained.

**Figure S12.4.4** presents the impact of various exercise interventions on overall symptom changes after adjusting for the male’s percentage of 45%. It compares and ranks different exercise types against the control group (CON) based on the mean difference (MD). The interventions crossing the y-axis show no significant difference compared to the control group.

**12.4.5** BMI
When the model was adjusted for centering value of BMI 27, the hierarchy from the unadjusted model retained.

**Figure S12.4.5** presents the impact of various exercise interventions on overall symptom changes after adjusting for BMI 27. It compares and ranks different exercise types against the control group (CON) based on the mean difference (MD). The interventions crossing the y-axis show no significant difference compared to the control group.

**12.4.6** Exercise period
When the model was adjusted for centering value of exercise period 23 weeks, the hierarchy from the unadjusted model retained.

**Figure S12.4.6** presents the impact of various exercise interventions on overall symptom changes after adjusting for the exercise period of 23 weeks. It compares and ranks different exercise types against the control group (CON) based on the mean difference (MD). The interventions crossing the y-axis show no significant difference compared to the control group.

**12.4.7** Exercise frequency
When the model was adjusted for centering value of exercise frequency (4 times per week), the hierarchy from the unadjusted model retained.
**Figure S12.4.7** presents the impact of various exercise interventions on overall symptom changes after adjusting for the exercise frequency of 4 times per week. It compares and ranks different exercise types against the control group (CON) based on the mean difference (MD). The interventions crossing the y-axis show no significant difference compared to the control group.

**12.4.8** Time per session
When the model was adjusted for centering value of time per session (47 minutes), the hierarchy from the unadjusted model retained.
**Figure S12.4.8** presents the impact of various exercise interventions on overall symptom changes after adjusting for the time per session of 47 minutes. It compares and ranks different exercise types against the control group (CON) based on the mean difference (MD). The interventions crossing the y-axis show no significant difference compared to the control group.

**12.4.9** Time per week
When the model was adjusted for centering value of time per week (182 minutes), the hierarchy from the unadjusted model retained.
**Figure S12.4.9** presents the impact of various exercise interventions on overall symptom changes after adjusting for the time per week of 182 minutes. It compares and ranks different exercise types against the control group (CON) based on the mean difference (MD). The interventions crossing the y-axis show no significant difference compared to the control group.

**12.5** TC

**12.5.1** Publication year

When the model was adjusted for centering value of publish year 2017, the hierarchy from the unadjusted model retained.

**Figure S12.5.1** presents the impact of various exercise interventions on overall symptom changes after adjusting for the publication year 2017. It compares and ranks different exercise types against the control group (CON) based on the mean difference (MD). The interventions crossing the y-axis show no significant difference compared to the control group.

**12.5.2** Sample size
When the model was adjusted for centering value of sample size 37, the hierarchy from the unadjusted model retained.

**Figure S12.5.2** presents the impact of various exercise interventions on overall symptom changes after adjusting for the sample size 37. It compares and ranks different exercise types against the control group (CON) based on the mean difference (MD). The interventions crossing the y-axis show no significant difference compared to the control group.

**12.5.3** Mean age
When the model was adjusted for centering value of mean age 57, the hierarchy from the unadjusted model retained.

**Figure S12.5.3** presents the impact of various exercise interventions on overall symptom changes after adjusting for the mean age of 57. It compares and ranks different exercise types against the control group (CON) based on the mean difference (MD). The interventions crossing the y-axis show no significant difference compared to the control group.

**12.5.4** Percentage of Male

When the model was adjusted for centering value of male’s percentage 48%, the hierarchy from the unadjusted model retained.

**Figure S12.5.4** presents the impact of various exercise interventions on overall symptom changes after adjusting for the male’s percentage of 48%. It compares and ranks different exercise types against the control group (CON) based on the mean difference (MD). The interventions crossing the y-axis show no significant difference compared to the control group.

**12.5.5** BMI
When the model was adjusted for centering value of BMI 27, the hierarchy from the unadjusted model retained.

**Figure S12.5.5** presents the impact of various exercise interventions on overall symptom changes after adjusting for BMI 27. It compares and ranks different exercise types against the control group (CON) based on the mean difference (MD). The interventions crossing the y-axis show no significant difference compared to the control group.

**12.5.6** Exercise period
When the model was adjusted for centering value of exercise period 27 weeks, the hierarchy from the unadjusted model retained.

**Figure S12.5.6** presents the impact of various exercise interventions on overall symptom changes after adjusting for the exercise period of 27 weeks. It compares and ranks different exercise types against the control group (CON) based on the mean difference (MD). The interventions crossing the y-axis show no significant difference compared to the control group.

**12.5.7** Exercise frequency
When the model was adjusted for centering value of exercise frequency (3 times per week), the hierarchy from the unadjusted model retained.
**Figure S12.5.7** presents the impact of various exercise interventions on overall symptom changes after adjusting for the exercise frequency of 3 times per week. It compares and ranks different exercise types against the control group (CON) based on the mean difference (MD). The interventions crossing the y-axis show no significant difference compared to the control group.

**12.5.8** Time per session
When the model was adjusted for centering value of time per session (50 minutes), the hierarchy from the unadjusted model retained.
**Figure S12.5.8** presents the impact of various exercise interventions on overall symptom changes after adjusting for the time per session of 50 minutes. It compares and ranks different exercise types against the control group (CON) based on the mean difference (MD). The interventions crossing the y-axis show no significant difference compared to the control group.

**12.5.9** Time per week
When the model was adjusted for centering value of time per week (170 minutes), the hierarchy from the unadjusted model retained.
**Figure S12.5.9** presents the impact of various exercise interventions on overall symptom changes after adjusting for the time per week of 170 minutes. It compares and ranks different exercise types against the control group (CON) based on the mean difference (MD). The interventions crossing the y-axis show no significant difference compared to the control group.

**12.6** TG

**12.6.1** Publication year

When the model was adjusted for centering value of publish year 2018, the hierarchy from the unadjusted model retained.

**Figure S12.6.1** presents the impact of various exercise interventions on overall symptom changes after adjusting for the publication year 2018. It compares and ranks different exercise types against the control group (CON) based on the mean difference (MD). The interventions crossing the y-axis show no significant difference compared to the control group.

**12.6.2** Sample size
When the model was adjusted for centering value of sample size 38, the hierarchy from the unadjusted model retained.

**Figure S12.6.2** presents the impact of various exercise interventions on overall symptom changes after adjusting for the sample size 38. It compares and ranks different exercise types against the control group (CON) based on the mean difference (MD). The interventions crossing the y-axis show no significant difference compared to the control group.

**12.6.3** Mean age
When the model was adjusted for centering value of mean age 57, the hierarchy from the unadjusted model retained.

**Figure S12.6.3** presents the impact of various exercise interventions on overall symptom changes after adjusting for the mean age of 57. It compares and ranks different exercise types against the control group (CON) based on the mean difference (MD). The interventions crossing the y-axis show no significant difference compared to the control group.

**12.6.4** Percentage of Male

When the model was adjusted for centering value of male’s percentage 48%, the hierarchy from the unadjusted model retained.

**Figure S12.6.4** presents the impact of various exercise interventions on overall symptom changes after adjusting for the male’s percentage of 48%. It compares and ranks different exercise types against the control group (CON) based on the mean difference (MD). The interventions crossing the y-axis show no significant difference compared to the control group.

**12.6.5** BMI
When the model was adjusted for centering value of BMI 27, the hierarchy from the unadjusted model retained.

**Figure S12.6.5** presents the impact of various exercise interventions on overall symptom changes after adjusting for BMI 27. It compares and ranks different exercise types against the control group (CON) based on the mean difference (MD). The interventions crossing the y-axis show no significant difference compared to the control group.

**12.6.6** Exercise period
When the model was adjusted for centering value of exercise period 27 weeks, the hierarchy from the unadjusted model retained.

**Figure S12.6.6** presents the impact of various exercise interventions on overall symptom changes after adjusting for the exercise period of 27 weeks. It compares and ranks different exercise types against the control group (CON) based on the mean difference (MD). The interventions crossing the y-axis show no significant difference compared to the control group.

**12.6.7** Exercise frequency
When the model was adjusted for centering value of exercise frequency (3 times per week), the hierarchy from the unadjusted model retained.
**Figure S12.6.7** presents the impact of various exercise interventions on overall symptom changes after adjusting for the exercise frequency of 3 times per week. It compares and ranks different exercise types against the control group (CON) based on the mean difference (MD). The interventions crossing the y-axis show no significant difference compared to the control group.

**12.6.8** Time per session
When the model was adjusted for centering value of time per session (50 minutes), the hierarchy from the unadjusted model retained.
**Figure S12.6.8** presents the impact of various exercise interventions on overall symptom changes after adjusting for the time per session of 50 minutes. It compares and ranks different exercise types against the control group (CON) based on the mean difference (MD). The interventions crossing the y-axis show no significant difference compared to the control group.

**12.6.9** Time per week
When the model was adjusted for centering value of time per week (171 minutes), the hierarchy from the unadjusted model retained.
**Figure S12.6.9** presents the impact of various exercise interventions on overall symptom changes after adjusting for the time per week of 171 minutes. It compares and ranks different exercise types against the control group (CON) based on the mean difference (MD). The interventions crossing the y-axis show no significant difference compared to the control group.

**12.7** HDL

**12.7.1** Publication year

When the model was adjusted for centering value of publish year 2017, the hierarchy from the unadjusted model retained.

**Figure S12.7.1** presents the impact of various exercise interventions on overall symptom changes after adjusting for the publication year 2017. It compares and ranks different exercise types against the control group (CON) based on the mean difference (MD). The interventions crossing the y-axis show no significant difference compared to the control group.

**12.7.2** Sample size
When the model was adjusted for centering value of sample size 38, the hierarchy from the unadjusted model retained.

**Figure S12.7.2** presents the impact of various exercise interventions on overall symptom changes after adjusting for the sample size 38. It compares and ranks different exercise types against the control group (CON) based on the mean difference (MD). The interventions crossing the y-axis show no significant difference compared to the control group.

**12.7.3** Mean age

When the model was adjusted for centering value of mean age 57, the hierarchy from the unadjusted model retained.

**Figure S12.7.3** presents the impact of various exercise interventions on overall symptom changes after adjusting for the mean age of 57. It compares and ranks different exercise types against the control group (CON) based on the mean difference (MD). The interventions crossing the y-axis show no significant difference compared to the control group.

**12.7.4** Percentage of Male

When the model was adjusted for centering value of male’s percentage 48%, the hierarchy from the unadjusted model retained.

**Figure S12.7.4** presents the impact of various exercise interventions on overall symptom changes after adjusting for the male’s percentage of 48%. It compares and ranks different exercise types against the control group (CON) based on the mean difference (MD). The interventions crossing the y-axis show no significant difference compared to the control group.

**12.7.5** BMI

When the model was adjusted for centering value of BMI 27, the hierarchy from the unadjusted model retained.

**Figure S12.7.6** presents the impact of various exercise interventions on overall symptom changes after adjusting for BMI 27. It compares and ranks different exercise types against the control group (CON) based on the mean difference (MD). The interventions crossing the y-axis show no significant difference compared to the control group.

**12.7.6** Exercise period
When the model was adjusted for centering value of exercise period 27 weeks, the hierarchy from the unadjusted model retained.

**Figure S12.7.6** presents the impact of various exercise interventions on overall symptom changes after adjusting for the exercise period of 27 weeks. It compares and ranks different exercise types against the control group (CON) based on the mean difference (MD). The interventions crossing the y-axis show no significant difference compared to the control group.

**12.7.7** Exercise frequency
When the model was adjusted for centering value of exercise frequency (3 times per week), the hierarchy from the unadjusted model retained.
**Figure S12.7.7** presents the impact of various exercise interventions on overall symptom changes after adjusting for the exercise frequency of 3 times per week. It compares and ranks different exercise types against the control group (CON) based on the mean difference (MD). The interventions crossing the y-axis show no significant difference compared to the control group.

**12.7.8** Time per session
When the model was adjusted for centering value of time per session (50 minutes), the hierarchy from the unadjusted model retained.
**Figure S12.7.8** presents the impact of various exercise interventions on overall symptom changes after adjusting for the time per session of 50 minutes. It compares and ranks different exercise types against the control group (CON) based on the mean difference (MD). The interventions crossing the y-axis show no significant difference compared to the control group.

**12.7.9** Time per week
When the model was adjusted for centering value of time per week (170 minutes), the hierarchy from the unadjusted model retained.
**Figure S12.7.9** presents the impact of various exercise interventions on overall symptom changes after adjusting for the time per week of 170 minutes. It compares and ranks different exercise types against the control group (CON) based on the mean difference (MD). The interventions crossing the y-axis show no significant difference compared to the control group.

**12.8** LDL

**12.8.1** Publication year

When the model was adjusted for centering value of publish year 2018, the hierarchy from the unadjusted model retained.

**Figure S12.8.1** presents the impact of various exercise interventions on overall symptom changes after adjusting for the publication year 2018. It compares and ranks different exercise types against the control group (CON) based on the mean difference (MD). The interventions crossing the y-axis show no significant difference compared to the control group.

**12.8.2** Sample size
When the model was adjusted for centering value of sample size 39, the hierarchy from the unadjusted model retained.

**Figure S12.8.2** presents the impact of various exercise interventions on overall symptom changes after adjusting for the sample size 39. It compares and ranks different exercise types against the control group (CON) based on the mean difference (MD). The interventions crossing the y-axis show no significant difference compared to the control group.

**12.8.3** Mean age
When the model was adjusted for centering value of mean age 57, the hierarchy from the unadjusted model retained.

**Figure S12.8.3** presents the impact of various exercise interventions on overall symptom changes after adjusting for the mean age of 57. It compares and ranks different exercise types against the control group (CON) based on the mean difference (MD). The interventions crossing the y-axis show no significant difference compared to the control group.

**12.8.4** Percentage of Male

When the model was adjusted for centering value of male’s percentage 48%, the hierarchy from the unadjusted model retained.

**Figure S12.8.4** presents the impact of various exercise interventions on overall symptom changes after adjusting for the male’s percentage of 48%. It compares and ranks different exercise types against the control group (CON) based on the mean difference (MD). The interventions crossing the y-axis show no significant difference compared to the control group.

**12.8.5** BMI
When the model was adjusted for centering value of BMI 27, the hierarchy from the unadjusted model retained.

**Figure S12.8.5** presents the impact of various exercise interventions on overall symptom changes after adjusting for BMI 27. It compares and ranks different exercise types against the control group (CON) based on the mean difference (MD). The interventions crossing the y-axis show no significant difference compared to the control group.

**12.8.6** Exercise period
When the model was adjusted for centering value of exercise period 27 weeks, the hierarchy from the unadjusted model retained.

**Figure S12.8.6** presents the impact of various exercise interventions on overall symptom changes after adjusting for the exercise period of 27 weeks. It compares and ranks different exercise types against the control group (CON) based on the mean difference (MD). The interventions crossing the y-axis show no significant difference compared to the control group.

**12.8.7** Exercise frequency
When the model was adjusted for centering value of exercise frequency (3 times per week), the hierarchy from the unadjusted model retained.
**Figure S12.8.7** presents the impact of various exercise interventions on overall symptom changes after adjusting for the exercise frequency of 3 times per week. It compares and ranks different exercise types against the control group (CON) based on the mean difference (MD). The interventions crossing the y-axis show no significant difference compared to the control group.

**12.8.8** Time per session
When the model was adjusted for centering value of time per session (50 minutes), the hierarchy from the unadjusted model retained.
**Figure S12.8.8** presents the impact of various exercise interventions on overall symptom changes after adjusting for the time per session of 50 minutes. It compares and ranks different exercise types against the control group (CON) based on the mean difference (MD). The interventions crossing the y-axis show no significant difference compared to the control group.

**12.8.9** Time per week
When the model was adjusted for centering value of time per week (171 minutes), the hierarchy from the unadjusted model retained.
**Figure S12.8.9** presents the impact of various exercise interventions on overall symptom changes after adjusting for the time per week of 171 minutes. It compares and ranks different exercise types against the control group (CON) based on the mean difference (MD). The interventions crossing the y-axis show no significant difference compared to the control group.

**12.9** BMI

**12.9.1** Publication year

When the model was adjusted for centering value of publish year 2017, the hierarchy from the unadjusted model retained.

**Figure S12.9.1** presents the impact of various exercise interventions on overall symptom changes after adjusting for the publication year 2017. It compares and ranks different exercise types against the control group (CON) based on the mean difference (MD). The interventions crossing the y-axis show no significant difference compared to the control group.

**12.9.2** Sample size
When the model was adjusted for centering value of sample size 33, the hierarchy from the unadjusted model retained.

**Figure S12.9.2** presents the impact of various exercise interventions on overall symptom changes after adjusting for the sample size 33. It compares and ranks different exercise types against the control group (CON) based on the mean difference (MD). The interventions crossing the y-axis show no significant difference compared to the control group.

**12.9.3** Mean age
When the model was adjusted for centering value of mean age 57, the hierarchy from the unadjusted model retained.

**Figure S12.9.3** presents the impact of various exercise interventions on overall symptom changes after adjusting for the mean age of 57. It compares and ranks different exercise types against the control group (CON) based on the mean difference (MD). The interventions crossing the y-axis show no significant difference compared to the control group.

**12.9.4** Percentage of Male

When the model was adjusted for centering value of male’s percentage 42%, the hierarchy from the unadjusted model retained.

**Figure S12.9.4** presents the impact of various exercise interventions on overall symptom changes after adjusting for the male’s percentage of 42%. It compares and ranks different exercise types against the control group (CON) based on the mean difference (MD). The interventions crossing the y-axis show no significant difference compared to the control group.

**12.9.5** BMI
When the model was adjusted for centering value of BMI 28, the hierarchy from the unadjusted model retained.

**Figure S12.9.5** presents the impact of various exercise interventions on overall symptom changes after adjusting for BMI 28. It compares and ranks different exercise types against the control group (CON) based on the mean difference (MD). The interventions crossing the y-axis show no significant difference compared to the control group.

**12.9.6** Exercise period
When the model was adjusted for centering value of exercise period 23 weeks, the hierarchy from the unadjusted model retained.

**Figure S12.9.6** presents the impact of various exercise interventions on overall symptom changes after adjusting for the exercise period of 23 weeks. It compares and ranks different exercise types against the control group (CON) based on the mean difference (MD). The interventions crossing the y-axis show no significant difference compared to the control group.

**12.9.7** Exercise frequency
When the model was adjusted for centering value of exercise frequency (4 times per week), the hierarchy from the unadjusted model retained.
**Figure S12.9.7** presents the impact of various exercise interventions on overall symptom changes after adjusting for the exercise frequency of 4 times per week. It compares and ranks different exercise types against the control group (CON) based on the mean difference (MD). The interventions crossing the y-axis show no significant difference compared to the control group.

**12.9.8** Time per session
When the model was adjusted for centering value of time per session (48 minutes), the hierarchy from the unadjusted model retained.
**Figure S12.9.8** presents the impact of various exercise interventions on overall symptom changes after adjusting for the time per session of 48 minutes. It compares and ranks different exercise types against the control group (CON) based on the mean difference (MD). The interventions crossing the y-axis show no significant difference compared to the control group.

**12.9.9** Time per week
When the model was adjusted for centering value of time per week (181 minutes), the hierarchy from the unadjusted model retained.
**Figure S12.9.9** presents the impact of various exercise interventions on overall symptom changes after adjusting for the time per week of 181 minutes. It compares and ranks different exercise types against the control group (CON) based on the mean difference (MD). The interventions crossing the y-axis show no significant difference compared to the control group.

**12.10** BW

**12.10.1** Publication year

When the model was adjusted for centering value of publish year 2018, the hierarchy from the unadjusted model retained.

**Figure S12.10.1** presents the impact of various exercise interventions on overall symptom changes after adjusting for the publication year 2018. It compares and ranks different exercise types against the control group (CON) based on the mean difference (MD). The interventions crossing the y-axis show no significant difference compared to the control group.

**12.10.2** Sample size
When the model was adjusted for centering value of sample size 30, the hierarchy from the unadjusted model retained.

**Figure S12.10.2** presents the impact of various exercise interventions on overall symptom changes after adjusting for the sample size 30. It compares and ranks different exercise types against the control group (CON) based on the mean difference (MD). The interventions crossing the y-axis show no significant difference compared to the control group.

**12.10.3** Mean age
When the model was adjusted for centering value of mean age 56, the hierarchy from the unadjusted model retained.

**Figure S12.10.3** presents the impact of various exercise interventions on overall symptom changes after adjusting for the mean age of 56. It compares and ranks different exercise types against the control group (CON) based on the mean difference (MD). The interventions crossing the y-axis show no significant difference compared to the control group.

**12.10.4** Percentage of Male

When the model was adjusted for centering value of male’s percentage 39%, the hierarchy from the unadjusted model retained.

**Figure S12.10.4** presents the impact of various exercise interventions on overall symptom changes after adjusting for the male’s percentage of 39%. It compares and ranks different exercise types against the control group (CON) based on the mean difference (MD). The interventions crossing the y-axis show no significant difference compared to the control group.

**12.10.5** BMI
When the model was adjusted for centering value of BMI 30, the hierarchy from the unadjusted model retained.

**Figure S12.10.5** presents the impact of various exercise interventions on overall symptom changes after adjusting for BMI 30. It compares and ranks different exercise types against the control group (CON) based on the mean difference (MD). The interventions crossing the y-axis show no significant difference compared to the control group.

**12.10.6** Exercise period
When the model was adjusted for centering value of exercise period 16 weeks, the hierarchy from the unadjusted model retained.

**Figure S12.10.6** presents the impact of various exercise interventions on overall symptom changes after adjusting for the exercise period of 16 weeks. It compares and ranks different exercise types against the control group (CON) based on the mean difference (MD). The interventions crossing the y-axis show no significant difference compared to the control group.

**12.10.7** Exercise frequency
When the model was adjusted for centering value of exercise frequency (4 times per week), the hierarchy from the unadjusted model retained.
**Figure S12.10.7** presents the impact of various exercise interventions on overall symptom changes after adjusting for the exercise frequency of 4 times per week. It compares and ranks different exercise types against the control group (CON) based on the mean difference (MD). The interventions crossing the y-axis show no significant difference compared to the control group.

**12.10.8** Time per session
When the model was adjusted for centering value of time per session (45 minutes), the hierarchy from the unadjusted model retained.
**Figure S12.10.8** presents the impact of various exercise interventions on overall symptom changes after adjusting for the time per session of 45 minutes. It compares and ranks different exercise types against the control group (CON) based on the mean difference (MD). The interventions crossing the y-axis show no significant difference compared to the control group.

**12.10.9** Time per week
When the model was adjusted for centering value of time per week (175 minutes), the hierarchy from the unadjusted model retained.
**Figure S12.10.9** presents the impact of various exercise interventions on overall symptom changes after adjusting for the time per week of 175 minutes. It compares and ranks different exercise types against the control group (CON) based on the mean difference (MD). The interventions crossing the y-axis show no significant difference compared to the control group.

**12.11** WC

**12.11.1** Publication year

When the model was adjusted for centering value of publish year 2016, the hierarchy from the unadjusted model retained.

**Figure S12.11.1** presents the impact of various exercise interventions on overall symptom changes after adjusting for the publication year 2016. It compares and ranks different exercise types against the control group (CON) based on the mean difference (MD). The interventions crossing the y-axis show no significant difference compared to the control group.

**12.11.2** Sample size
When the model was adjusted for centering value of sample size 29, the hierarchy from the unadjusted model retained.

**Figure S12.11.2** presents the impact of various exercise interventions on overall symptom changes after adjusting for the sample size 29. It compares and ranks different exercise types against the control group (CON) based on the mean difference (MD). The interventions crossing the y-axis show no significant difference compared to the control group.

**12.11.3** Mean age
When the model was adjusted for centering value of mean age 55, the hierarchy from the unadjusted model retained.

**Figure S12.11.3** presents the impact of various exercise interventions on overall symptom changes after adjusting for the mean age of 55. It compares and ranks different exercise types against the control group (CON) based on the mean difference (MD). The interventions crossing the y-axis show no significant difference compared to the control group.

**12.11.4** Percentage of Male

When the model was adjusted for centering value of male’s percentage 43%, the hierarchy from the unadjusted model retained.

**Figure S12.11.4** presents the impact of various exercise interventions on overall symptom changes after adjusting for the male’s percentage of 43%. It compares and ranks different exercise types against the control group (CON) based on the mean difference (MD). The interventions crossing the y-axis show no significant difference compared to the control group.

**12.11.5** BMI
When the model was adjusted for centering value of BMI 29, the hierarchy from the unadjusted model retained.

**Figure S12.11.5** presents the impact of various exercise interventions on overall symptom changes after adjusting for BMI 29. It compares and ranks different exercise types against the control group (CON) based on the mean difference (MD). The interventions crossing the y-axis show no significant difference compared to the control group.

**12.11.6** Exercise period
When the model was adjusted for centering value of exercise period 15 weeks, the hierarchy from the unadjusted model retained.

**Figure S12.11.6** presents the impact of various exercise interventions on overall symptom changes after adjusting for the exercise period of 15 weeks. It compares and ranks different exercise types against the control group (CON) based on the mean difference (MD). The interventions crossing the y-axis show no significant difference compared to the control group.

**12.11.7** Exercise frequency
When the model was adjusted for centering value of exercise frequency (4 times per week), the hierarchy from the unadjusted model retained.
**Figure S12.11.7** presents the impact of various exercise interventions on overall symptom changes after adjusting for the exercise frequency of 4 times per week. It compares and ranks different exercise types against the control group (CON) based on the mean difference (MD). The interventions crossing the y-axis show no significant difference compared to the control group.

**12.11.8** Time per session
When the model was adjusted for centering value of time per session (40 minutes), the hierarchy from the unadjusted model retained.
**Figure S12.11.8** presents the impact of various exercise interventions on overall symptom changes after adjusting for the time per session of 40 minutes. It compares and ranks different exercise types against the control group (CON) based on the mean difference (MD). The interventions crossing the y-axis show no significant difference compared to the control group.

**12.11.9** Time per week
When the model was adjusted for centering value of time per week (148 minutes), the hierarchy from the unadjusted model retained.
**Figure S12.11.9** presents the impact of various exercise interventions on overall symptom changes after adjusting for the time per week of 148 minutes. It compares and ranks different exercise types against the control group (CON) based on the mean difference (MD). The interventions crossing the y-axis show no significant difference compared to the control group.

# Appendix 13: Sensitivity Analysis

Sensitivity analyses were undertaken by excluding studies classified as having a high risk of bias, in order to assess the robustness of the results.

**Figure S13.1** Forest plot of HbA1c

**Figure S13.2** Forest plot of FBG

**Figure S13.3** Forest plot of 2hPG

**Figure S13.4** Forest plot of TG

**Figure S13.5** Forest plot of TC

**Figure S13.6** Forest plot of HDL

**Figure S13.7** Forest plot of LDL

**Figure S13.8** Forest plot of BMI

**Figure S13.9** Forest plot of BW

**Figure S13.10** Forest plot of WC

# Appendix 14: subgroups analysis

**Figure S14.1** Forest plot of FBG

**Figure S14.2** Forest plot of 2hPG

**Figure S14.3** Forest plot of TC

**Figure S14.4** Forest plot of LDL

**Figure S14.5** Forest plot of BW
